# Burden of anemia and its underlying causes in 204 countries and territories, 1990–2019: results from the Global Burden of Disease Study 2019

**DOI:** 10.1186/s13045-021-01202-2

**Published:** 2021-11-04

**Authors:** Saeid Safiri, Ali-Asghar Kolahi, Maryam Noori, Seyed Aria Nejadghaderi, Nahid Karamzad, Nicola Luigi Bragazzi, Mark J. M. Sullman, Morteza Abdollahi, Gary S. Collins, Jay S. Kaufman, Jessica A. Grieger

**Affiliations:** 1 Research Center for Integrative Medicine in Aging, Aging Research Institute, Tabriz University of Medical Sciences, Tabriz, Iran; 2 Social Determinants of Health Research Center, Department of Community Medicine, Faculty of Medicine, Tabriz University of Medical Sciences, Tabriz, Iran; 3 Social Determinants of Health Research Center, Shahid Beheshti University of Medical Sciences, Tehran, Iran; 4 Student Research Committee, School of Medicine, Iran University of Medical Sciences, Tehran, Iran; 5 School of Medicine, Shahid Beheshti University of Medical Sciences, Tehran, Iran; 6 Systematic Review and Meta-analysis Expert Group (SRMEG), Universal Scientific Education and Research Network (USERN), Tehran, Iran; 7 Nutrition Research Center, Department of Biochemistry and Diet Therapy, School of Nutrition and Food Sciences, Tabriz University of Medical Sciences, Tabriz, Iran; 8 Centre for Disease Modelling, York University, Toronto, ON Canada; 9 Department of Life and Health Sciences, University of Nicosia, Nicosia, Cyprus; 10 Department of Social Sciences, University of Nicosia, Nicosia, Cyprus; 11 Centre for Statistics in Medicine, NDORMS, Botnar Research Centre, University of Oxford, Oxford, UK; 12 NIHR Oxford Biomedical Research Centre, Oxford University Hospitals NHS Foundation Trust, Oxford, UK; 13 Department of Epidemiology, Biostatistics and Occupational Health, Faculty of Medicine, McGill University, Montreal, QC Canada; 14 Adelaide Medical School, University of Adelaide, Adelaide, SA Australia; 15 Robinson Research Institute, University of Adelaide, Adelaide, SA, Australia

**Keywords:** Anemia, Epidemiology, Global burden of disease, Prevalence, Years lived with disability

## Abstract

**Background:**

Anemia is a common disease which affects around 40% of children and 30% of reproductive age women and can have major health consequences. The present study reports the global, regional and national burden of anemia and its underlying causes between 1990 and 2019, by age, sex and socio-demographic index (SDI).

**Methods:**

Publicly available data on the point prevalence and years lived with disability (YLDs) were retrieved from the global burden of disease (GBD) 2019 study for 204 countries and territories between 1990 and 2019. The point prevalence, YLD counts and rates per 100,000 population were presented, along with their corresponding 95% uncertainty intervals.

**Results:**

In 2019, the global age-standardized point prevalence and YLD rates for anemia were 23,176.2 (22,943.5–23,418.6) and 672.4 (447.2–981.5) per 100,000 population, respectively. Moreover, the global age-standardized point prevalence and YLD rate decreased by 13.4% (12.1–14.5%) and 18.8% (16.9–20.8%), respectively, over the period 1990–2019. The highest national point prevalences of anemia were found in Zambia [49327.1 (95% UI: 46,838.5–51,700.1)], Mali [46890.1 (95% UI: 44,301.1–49,389.8)], and Burkina Faso [46117.2 (95% UI: 43,640.7–48,319.2)]. In 2019, the global point prevalence of anemia was highest in the 15–19 and 95+ age groups in females and males, respectively. Also, the burden of anemia was lower in regions with higher socio-economic development. Globally, most of the prevalent cases were attributable to dietary iron deficiency, as well as hemoglobinopathies and hemolytic anemias.

**Conclusions:**

Anemia remains a major health problem, especially among females in less developed countries. The implementation of preventive programs with a focus on improving access to iron supplements, early diagnosis and the treatment of hemoglobinopathies should be taken into consideration.

**Supplementary Information:**

The online version contains supplementary material available at 10.1186/s13045-021-01202-2.

## Introduction

Anemia is an important global health issue. According to the World Health Organization (WHO), anemia is defined as having hemoglobin (Hb) levels lower than 12.0 and 13.0 g/dL in females and males, respectively [1]. Although anemia has various correlates based on its underlying pathophysiology, nutritional deficiencies and chronic diseases are generally the most common etiologies of anemia in children and older adults, respectively [2, 3]. The clinical presentations and complications of anemia differ according to the type of anemia and its level of severity. The consequences of iron deficiency anemia may range from immune system dysfunction, disturbances in the gastrointestinal tract, impaired thermoregulation and neurocognitive function [4]. Moreover, anemia can be a risk or a prognostic factor for other diseases, such as tuberculosis and heart failure [5, 6].

The economic burden attributable to anemia differs according to the type and severity of the pre-existing comorbidities and has been found to range from $US29,511 in individuals with congestive heart failure to $US7,092 in those with comorbid rheumatoid arthritis [7]. Furthermore, the global prevalence of anemia decreased from 40.2% in 1990 to 32.9% in 2010 [8], with the number of prevalent cases of anemia being higher in females (than males) and among those under 5 years of age [8].

Previous articles have used different data sources to estimate the burden of anemia [8–10]. Kassebaum and colleagues used the global burden of disease (GBD) 2010 data to estimate the global burden of anemia and its contributing causes, although the association between anemia and socio-economic development was not reported [8]. Furthermore, another study used 257 population-representative data sources from 107 countries, from 1995 to 2011, to investigate the prevalence of anemia and Hb concentration in children and women aged 15–49 years old. However, the male population, women aged < 15 years and those aged > 49 years old (non-reproductive period) were not included in their study [9]. Kassebaum et al. updated their previous study using the GBD 2013 data [10]. Lastly, using GBD 2019 data, the overall burden of anemia and its contributing causes, by severity of disease, was reported as an *abstract supplement*, but a full paper was not produced [11]. Therefore, there has been no comprehensive study published on the global burden of anemia and its contributing causes, since GBD 2013. With this in mind, the present study used the most recently available data from the GBD project to report the point prevalence of anemia, years lived with disability (YLDs) and its attributable underlying causes by age, sex and socio-demographic index (SDI) in 204 countries and territories between 1990 and 2019.

## Methods

### Overview

The GBD project examines the levels and trends associated with diseases and injuries across the world. GBD 2019, the most recent iteration of this study, included data for 369 diseases and injuries and 87 risk factors in 204 countries and territories, 7 super-regions and 21 regions from 1990 to 2019 [12]. The methodology used in GBD 2019 to estimate the burden of diseases, injuries and risk factors can be found in detail elsewhere [12, 13]. Information on the fatal and non-fatal estimates can be found at https://vizhub.healthdata.org/gbd-compare/ and http://ghdx.healthdata.org/gbd-results-tool.

### Case definition and data sources

In GBD 2019, anemia was defined as decreased blood concentrations of hemoglobin (Hb), irrespective of the underlying cause, red blood cell morphology or red blood cell function. The thresholds used to define individuals as anemic, and the anemia severity thresholds, are based on WHO thresholds for hemoglobin in g/L [14]. Furthermore, a different threshold was used for neonates than for the rest of the < 5 years old age group. As there are currently no international guidelines on the thresholds for diagnosing anemia in neonates, the thresholds selected were a blend of the WHO recommendations (for 6–59 month olds) and the higher hemoglobin levels typically seen in newborns [12]. Additional file 1: Table S1 presents the definitions of mild, moderate and severe anemia, based upon blood hemoglobin concentration.

A variety of data sources was used to estimate total anemia (i.e., “the envelope”). The criteria for inclusion included the use of quantitative measurements of hemoglobin, in either a population-based sample or group judged to be adequately representative of the age, sex and location of the study. Furthermore, population-based surveys, such as the Demographic and Health Survey (DHS), Multiple Indicator Cluster Survey (MICS) series, national micronutrient surveys and other national and sub-national nutrition surveys provided the majority of the data included. These were supplemented with information collated by the WHO Vitamin and Mineral Nutrition Information System (VMNIS) [12].

The sources with individual-level data were collated and placed into GBD age groups, by sex, according to seven different criteria: mean Hb, standard deviation (SD) of Hb, prevalence of severe anemia, prevalence of moderate anemia, prevalence of moderate + severe anemia, prevalence of mild anemia and the total prevalence of anemia. The pregnancy status was also recorded, but no adjustments were made to their Hb values. Information on the mean (and SD) Hb levels were extracted from the VMNIS and other literature sources, where available. Those sources without mean (and SD) values of Hb were excluded from the modeling process, but their prevalence data were extracted to help establish the predictive accuracy of the resultant model. In total, there were 703 data sources from 153 countries used in the modeling process. The sources of data used to obtain epidemiological data on anemia included population-based surveys, such as the Demographic and Health Survey (DHS), Multiple Indicator Cluster Survey (MICS) series, national micronutrient surveys, sources collated in the WHO Vitamin and Mineral Nutrition Information System (VMNIS) and other national and subnational nutrition surveys. These sources can be found using the GBD 2019 Data Input Sources Tool (http://ghdx.healthdata.org/gbd-2019/data-input-sources). However, there were differences in the types of databases used in some areas [12].

### Data processing and disease model

In the majority of the surveys, a HemoCue test was used, which was adjusted for altitude, but those with terminal or acute medical conditions were excluded. However, hemoglobin was typically measured using a Coulter counter in articles published in the scientific literature and those from higher income locations. These two methods, which were treated as equivalent in this research, work by reacting hemoglobin with a specific reagent (Drabkin’s solution) and measuring the absorbance wavelengths. Furthermore, no distinction was made between studies that collected whole blood from participants and those which used capillary venous sampling [12]. As Hb concentration increases with altitude, the Hb concentration was adjusted for altitude using the formula recommended by WHO. The studies that reported altitude-adjusted Hb data were included directly, without any adjustment, while those that did not present altitude-adjusted Hb values, but did present altitude, were adjusted using the equation mentioned above [12, 14].

Age- and sex-splitting were conducted on all studies included in the research, in order to fit within the predetermined GBD age- and sex-groups. The processing of Hb data from pregnant women varied according to the source. All population-based studies that sampled pregnant women were processed according to the information presented in Additional file 1: Table S1 and no Hb adjustments were made. In these cases, the assumption was made that the pregnancy rates in the surveyed population were representative of the adult female population being surveyed. The studies that *only* included pregnant women were crosswalked to the general population using the meta-regression–Bayesian, regularized, trimmed (MR-BRT) method [12].

The estimation of the overall prevalence of anemia was undertaken in four steps: (1) Spatiotemporal Gaussian Process Regression (ST-GPR) models of the mean (and SD) of the Hb concentration, (2) calculation of the ensemble weights, (3) generation of the ensemble distributions and (4) identifying the area under the curve (AUC). Further information on each of these step can be found in a previous article [12].

### Anemia causal attribution

There are many different diseases that can cause anemia. As scientific evidence improves, additional causes are added to the GBD Anemia Causal Attribution analysis. In GBD 2019, the following level 3 causes were included for anemia: chronic kidney disease; dietary iron deficiency; endocrine, metabolic, blood and immune disorders; gynecological diseases; hemoglobinopathies and hemolytic anemias; HIV/AIDS; inflammatory bowel disease; intestinal nematode infections; malaria; maternal disorders; other neglected tropical diseases; other unspecified infectious diseases; schistosomiasis; upper digestive system diseases; and vitamin A deficiency. A detailed definition of each can be found in a previous article [12].

### Compilation of results

The GBD 2013 European Disability Weights Measurement Study was used to determine the disability weights (DWs) for mild [0.004 (0.001–0.008)], moderate [0.052 (0.034–0.076)] and severe [0.149 (0.101–0.209)] anemia [15]. The prevalence of each severity category was then multiplied by severity-specific DWs in order to produce the years lived with disability (YLDs).

Years of life lost (YLLs) could not be calculated, since no mortality could be attributed to anemia; thus, YLDs were also equal to the disability adjusted life years (DALYs). Using the GBD standard population, the age-standardized rates were estimated and reported per 100,000 population, in terms of the age-standardized point prevalence and age-standardized YLD rates. Uncertainty was propagated by sampling 1000 draws at each computational step and combining uncertainty from multiple sources, including input data, corrections for measurement error and estimated residual non-sampling error. The uncertainty intervals (UIs) were defined as the 25th and 975th values of the 1000 ordered draws. The association between the burden of anemia, in terms of YLDs, and the Socio-demographic Index (SDI) for the 21 regions and 204 countries and territories were examined using smoothing splines models. [16]. SDI is an indicator of a country’s development level and is comprised of the lag-dependent income per capita, the gross domestic product per capita smoothed over the previous 10 years, education level among the population aged ≥ 15 years old and the total fertility rate < 25 years old. The SDI ranges from 0 (less developed) to 1 (most developed). R software, version 3.5.2, was used to map the age-standardized point prevalence and YLD rates.

## Results

### Global level

In 2019, there were 1.8 billion (95% UI: 1.7–1.8) prevalent cases of anemia across the world, with an age-standardized point prevalence of 23,176.2 per 100,000 population (95% UI: 22,943.5–23,418.6), which is 13.4% lower than in 1990 (95% UI: 12.1–14.5) (the world population was estimated to be 5.35 billion in 1990 and 7.74 billion in 2019). Anemia accounted for 50.3 million (95% UI: 33.4–73.4) YLDs in 2019, with an age-standardized rate of 672.4 (95% UI: 447.2–981.5), which is 18.8% lower than in 1990 (95% UI: 16.9–20.8) (Table 1).

**Table 1:** Prevalent cases and years lived with disability (YLDs) for anemia in 2019 and the percentage change in the age-standardized rates (ASRs) per 100,000, by GBD region, from 1990 to 2019 (Generated from data available from http://ghdx.healthdata.org/gbd-results-tool)

	Prevalence					YLD				
	1990		2019		% change in ASRs between 1990 and 2019	1990		2019		% change in ASRs between 1990 and 2019
	No (95% UI)	ASRs per 100,000 (95% UI)	No (95% UI)	ASRs per 100,000 (95% UI)		No (95% UI)	ASRs per 100,000 (95% UI)	No (95% UI)	ASRs per 100,000 (95% UI)	
Global	1,441,197,733 (1,427,406,375, 1,454,392,253)	26,752 (26,508.1, 26,978.6)	1,761,561,953 (1,744,013,909, 1,779,850,189)	23,176.2 (22,943.5, 23,418.6)	− 13.4 (− 14.5, − 12.1)	45,434,224 (30,161,499, 65,937,847)	828 (550.5, 1200)	50,296,485 (33,399,077, 73,432,754)	672.4 (447.2, 981.5)	− 18.8 (− 20.8, − 16.9)
High-income Asia Pacific	31,294,014 (29,228,325, 33,382,953)	17,659.1 (16,428.4, 18,908.4)	24,772,126 (22,617,793, 27,016,500)	11,037.4 (9947, 12,361.4)	− 37.5 (− 45.1, − 28.6)	682,181 (436,230, 1,029,532)	392.2 (252, 590.5)	413,741 (259,883, 642,035)	194.6 (120.4, 301)	− 50.4 (− 58.1, − 41.9)
High-income North America	22,403,849 (20,595,793, 24,319,566)	7684.7 (7039.3, 8370.9)	27,820,834 (24,886,580, 31,222,390)	6920 (6162.3, 7806.5)	− 10 (− 22, 4.8)	359,204 (222,789, 554,376)	124.5 (77.3, 191.1)	443,997 (273,887, 695,503)	112.9 (68.4, 177.5)	− 9.3 (− 24.4, 10.6)
Western Europe	28,812,625 (27,043,936, 30,737,146)	7575.6 (7082, 8110.3)	22,120,972 (20,605,893, 23,792,370)	4880.1 (4491.3, 5308.1)	− 35.6 (− 41.6, − 28.7)	443,251 (274,294, 697,421)	120.9 (74.7, 189.9)	306,588 (191,681, 481,932)	70.8 (44.3, 112.5)	− 41.5 (− 49.1, − 33.7)
Australasia	1,845,288 (1,591,653, 2,114,956)	9418.5 (8055.6, 10,884.9)	1,942,195 (1,649,693, 2,271,715)	6765.7 (5571.5, 8157.6)	− 28.2 (− 43.2, − 8.9)	30,897 (19,046, 49,770)	159.7 (98.7, 256.2)	29,143 (17,242, 47,395)	102.8 (59.1, 166.9)	− 35.6 (− 52.5, − 13.1)
Andean Latin America	9,993,482 (9,411,505, 10,614,309)	25,521.7 (24,229.8, 26,833.5)	9,988,961 (9,351,246, 10,696,617)	15,746.5 (14,742.1, 16,875.9)	− 38.3 (− 43.3, − 32.5)	305,793 (197,376, 455,780)	761.7 (497.5, 1123.7)	219,766 (139,238, 329,651)	347.6 (220.7, 520.1)	− 54.4 (− 59.8, − 48.1)
Tropical Latin America	38,404,299 (35,162,828, 41,567,727)	25,892 (23,851.1, 27,868.6)	42,933,173 (38,993,225, 46,967,298)	19,063.1 (17,259.7, 20,779.5)	− 26.4 (− 34.6, − 17)	1,135,088 (723,760, 1,694,993)	744.1 (475.3, 1122)	1,069,215 (681,813, 1,668,724)	482.7 (308.4, 738.4)	− 35.1 (− 45, − 23.8)
Central Latin America	24,052,521 (23,217,633, 24,971,481)	15,061 (14,582.2, 15,567.2)	24,973,205 (24,051,149, 26,013,867)	10,286.9 (9918.3, 10,716.6)	− 31.7 (− 34.8, − 28.4)	598,408 (390,003, 894,761)	364.5 (238.7, 543.8)	518,615 (338,670, 765,747)	215.7 (141, 318.5)	− 40.8 (− 44.5, − 37.1)
Southern Latin America	8,302,071 (7,686,993, 8,952,093)	16,834.1 (15,617.3, 18,146.1)	7,667,450 (6,832,474, 8,619,405)	11,667.6 (10,346.7, 13,149)	− 30.7 (− 39.9, − 20.8)	174,290 (110,593, 261,928)	351.6 (224.7, 526.2)	131,795 (81,751, 202,574)	205.9 (126.8, 316.7)	− 41.5 (− 52.1, − 29.7)
Caribbean	9,621,664 (9,189,487, 10,054,721)	27,387.8 (26,198.1, 28,535.7)	11,886,493 (11,346,858, 12,405,308)	25,484.8 (24,316.8, 26,618.2)	− 6.9 (− 12.4, − 1.5)	247,026 (161,827, 361,514)	690.7 (454, 1014.3)	288,251 (187,090, 427,217)	629.5 (409.4, 931.5)	− 8.9 (− 16.3, − 1.8)
Central Europe	20,716,435 (19,619,911, 21,863,324)	17,256.4 (16,308, 18,224.2)	13,595,798 (12,724,085, 14,551,566)	12,637.2 (11,788, 13,577.9)	− 26.8 (− 32.3, − 21)	428,624 (273,625, 636,875)	364.5 (232.6, 538.7)	232,928 (148,386, 358,829)	226.6 (141.9, 347.2)	− 37.8 (− 43.9, − 30.8)
Eastern Europe	33,426,887 (30,713,405, 36,352,568)	14,023.4 (12,907.2, 15,251.6)	27,467,447 (24,847,685, 30,416,012)	11,042.4 (9962.7, 12,230.3)	− 21.3 (− 30, − 11)	678,143 (439,672, 1,016,172)	285.8 (184.9, 427.1)	491,571 (311,459, 755,427)	196.6 (122.7, 301.5)	− 31.2 (− 41.9, − 19.8)
Central Asia	20,996,220 (20,320,923, 21,720,706)	30,174.8 (29,283.3, 31,152.6)	24,438,402 (23,313,742, 25,576,821)	26,151.5 (24,981.8, 27,309.4)	− 13.3 (− 17.7, − 8.8)	623,987 (412,777, 915,967)	884.1 (584.9, 1297.1)	640,516 (415,160, 948,555)	681.9 (441.4, 1011.3)	− 22.9 (− 28.6, − 16.8)
North Africa and Middle East	88,326,772 (85,497,085, 91,386,150)	25,504.3 (24,785.9, 26,262.7)	107,540,095 (103,637,597, 111,903,977)	18,019.7 (17,398.5, 18,738.9)	− 29.3 (− 32.4, − 26.3)	2,477,794 (1,642,022, 3,658,563)	682.4 (455.1, 1005.7)	2,613,567 (1,704,030, 3,884,951)	434.8 (283.2, 648.5)	− 36.3 (− 40.2, − 32.5)
South Asia	517,489,379 (510,460,894, 524,063,402)	47,570.2 (47,039.5, 48,077.6)	731,143,119 (720,084,143, 741,777,108)	41,646.1 (41,034.3, 42,208.3)	− 12.5 (− 14, − 11)	19,440,166 (13,075,762, 27,962,135)	1755.7 (1179, 2513.3)	23,719,569 (15,826,969, 34,432,493)	1358.2 (907.3, 1972.1)	− 22.6 (− 25.3, − 20.1)
Southeast Asia	134,462,388 (129,750,492, 139,282,802)	29,935.5 (29,012.9, 30,855)	133,070,367 (127,670,127, 138,618,485)	20,313 (19,510.6, 21,123.8)	− 32.1 (− 35.7, − 28.9)	3,768,376 (2,454,042, 5,553,513)	819.2 (533.8, 1208.5)	2,933,216 (1,906,247, 4,391,293)	454 (295.7, 679.7)	− 44.6 (− 48.8, − 40.1)
East Asia	242,317,203 (234,616,647, 249,609,434)	20,520.1 (19,942.1, 21,077)	134,607,357 (126,648,483, 143,183,464)	8358.4 (7840.8, 8888.8)	− 59.3 (− 62.1, − 56.2)	6,291,117 (4,163,036, 9,124,713)	536.3 (355.5, 778.6)	2,421,967 (1,515,435, 3,828,239)	156.8 (97.3, 242.3)	− 70.8 (− 74.8, − 66.6)
Oceania	2,159,756 (2,057,867, 2,269,190)	33,535.8 (32,159.4, 34,922.9)	4,117,576 (3,867,196, 4,392,826)	31,291.1 (29,606.7, 33,061)	− 6.7 (− 12.8, − 0.2)	70,323 (46,259, 101,768)	1043.5 (682.1, 1506)	123,409 (80,514, 181,276)	907.2 (594.6, 1321.3)	− 13.1 (− 21.7, − 4.1)
Western Sub-Saharan Africa	89,221,652 (86,947,344, 91,425,721)	40,960.7 (39,903.4, 42,019.9)	205,607,201 (199,087,872, 212,195,550)	40,977 (39,789.3, 42,154.8)	0 (− 3.3, 3.6)	3,436,819 (2,293,131, 4,937,123)	1405.9 (936.9, 2045.7)	7,272,620 (4,861,319, 10,527,853)	1317.8 (879.9, 1912.7)	− 6.3 (− 11, − 1.6)
Eastern Sub-Saharan Africa	78,781,361 (77,456,951, 80,153,343)	38,935.4 (38,313.5, 39,524.3)	138,926,032 (135,954,864, 142,247,277)	32,699.5 (32,069.9, 33,345.9)	− 16 (− 17.9, − 14)	2,923,146 (1,940,080, 4,253,163)	1334.3 (885.1, 1933)	4,380,697 (2,907,670, 6,427,045)	970.8 (646.2, 1419.7)	− 27.2 (− 30.4, − 24)
Central Sub-Saharan Africa	25,665,245 (24,472,259, 26,801,865)	44,195.3 (42,505.8, 45,846.3)	50,757,787 (48,119,655, 53,363,924)	36,861.4 (35,218.3, 38,434.2)	− 16.6 (− 21.1, − 11.8)	942,777 (628,865, 1,376,172)	1521.5 (1013.6, 2211.5)	1,599,655 (1,049,715, 2,328,750)	1089.1 (721.3, 1588.2)	− 28.4 (− 35, − 22)
Southern Sub-Saharan Africa	12,904,624 (12,018,565, 13,785,907)	24,631.4 (23,248.8, 26,109.9)	16,185,363 (15,329,927, 17,101,430)	20,967.7 (19,936.3, 22,072.8)	− 14.9 (− 21.3, − 7.8)	376,815 (247,060, 561,368)	705.3 (460.1, 1044.1)	445,660 (293,732, 652,913)	572.1 (379.6, 834.9)	− 18.9 (− 27.1, − 10)

Globally, the number of prevalent cases [mild: 954.3 million (95% UI: 945.0–964.0; moderate: 747.8 million (95% UI: 737.5–758.2); severe: 59.5 million (95% UI: 57.8–61.2)], age-standardized prevalence (per 100,000 population) [mild: 12,349.8 (95% UI: 12,230.7–12,472.9; moderate: 10,035.3 (95% UI: 9891.2–10,173.5); severe: 791.1 (95% UI: 768.0–815.0)], and the percentage change in the age-standardized prevalence from 1990 to 2019 [mild: − 10.0% (95% UI: − 11.0 to − 8.8; moderate: − 15.2 (95% UI: − 16.6 to − 13.5); severe: − 34.1(95% UI: − 36.6 to − 31.4)] differed by severity level (Additional file 2: Table S2). In addition, Additional file 3: Table S3 presents the global YLD numbers and age-standardized rates due to anemia in 2019, by severity level and their changes from 1990 to 2019.

### Regional level

In 2019, the age-standardized point prevalence of anemia (per 100,000 population) was highest in South Asia [41646.1 (95% UI: 41,034.3–42,208.3)], Western Sub-Saharan Africa [40977.0 (95% UI: 39,789.3–42,154.8)] and Central Sub-Saharan Africa [36861.4 (95% UI: 35,218.3–38,434.2)]. Western Europe [4880.1 (95% UI: 4491.3–5308.1)], Australasia [6765.7 (95% UI: 5571.5–8157.6)] and High-income North America [6920.0 (95% UI: 6162.3–7806.5)] had the lowest age-standardized rates (Table 1).

South Asia [1358.2 (95% UI: 907.3–1972.1)], Western Sub-Saharan Africa [1317.8 (95% UI: 897.9–1912.7)] and Central Sub-Saharan Africa [1089.1 (95% UI: 721.3–1588.2)] had the highest age-standardized YLD rates from anemia. The rates were lowest for Western Europe [70.8 (95% UI: 44.3–112.5)], Australasia [102.8 (95% UI: 59.1–166.9)] and High-income North America [112.9 (95% UI: 68.4–177.5)] (Table 1). The age-standardized point prevalence and YLD rates of anemia, for all GBD regions in 2019, are reported in Additional file 4: Fig. S1 and Additional file 5: Fig. S2, respectively.

All GBD regions showed a decrease in the age-standardized point prevalence of anemia from 1990 to 2019, with the largest decreases being in East Asia [− 59.3% (95% UI: − 62.1 to − 56.2)], Andean Latin America [− 38.3% (95% UI: − 43.3 to − 32.5)] and High-income Asia Pacific [− 37.5% (95% UI: − 45.1 to − 28.6)] (Table 1). In the same period, all regions showed a decrease in the age-standardized YLD rates of anemia, with the largest decreases being found in East Asia [− 70.8% (95% UI: − 74.8 to − 66.6)], Andean Latin America [− 54.4% (95% UI: − 59.8 to − 48.1)] and High-income Asia Pacific [− 50.4% (95% UI: − 58.1 to − 41.9)] (Table 1). The percentage change, during the period 1990–2019, in the age-standardized point prevalence and YLD rates for anemia are reported in Additional file 6: Fig. S3 and Additional file 7: Fig. S4, respectively.

The global number of prevalent cases of anemia increased from 1.4 billion (95% UI: 1.4–1.5) in 1990 to 1.8 billion (95% UI: 1.7–1.8) in 2019. South Asia, East Asia and Southeast Asia experienced the largest number of prevalent cases in 1990, while the highest in 2019 were found in South Asia, Western Sub-Saharan Africa and Eastern Sub-Saharan Africa (Table 1). The global number of YLDs due to anemia increased from 45.4 million (95% UI: 30.2–65.9) in 1990 to 50.3 million (95% UI: 33.4–73.4) in 2019. South Asia, East Asia and Southeast Asia had the highest numbers of YLDs due to anemia in 1990, while in 2019 the highest numbers were found in South Asia, Western Sub-Saharan Africa and Eastern Sub-Saharan Africa (Table 1).

### National level

**Fig. 1:**
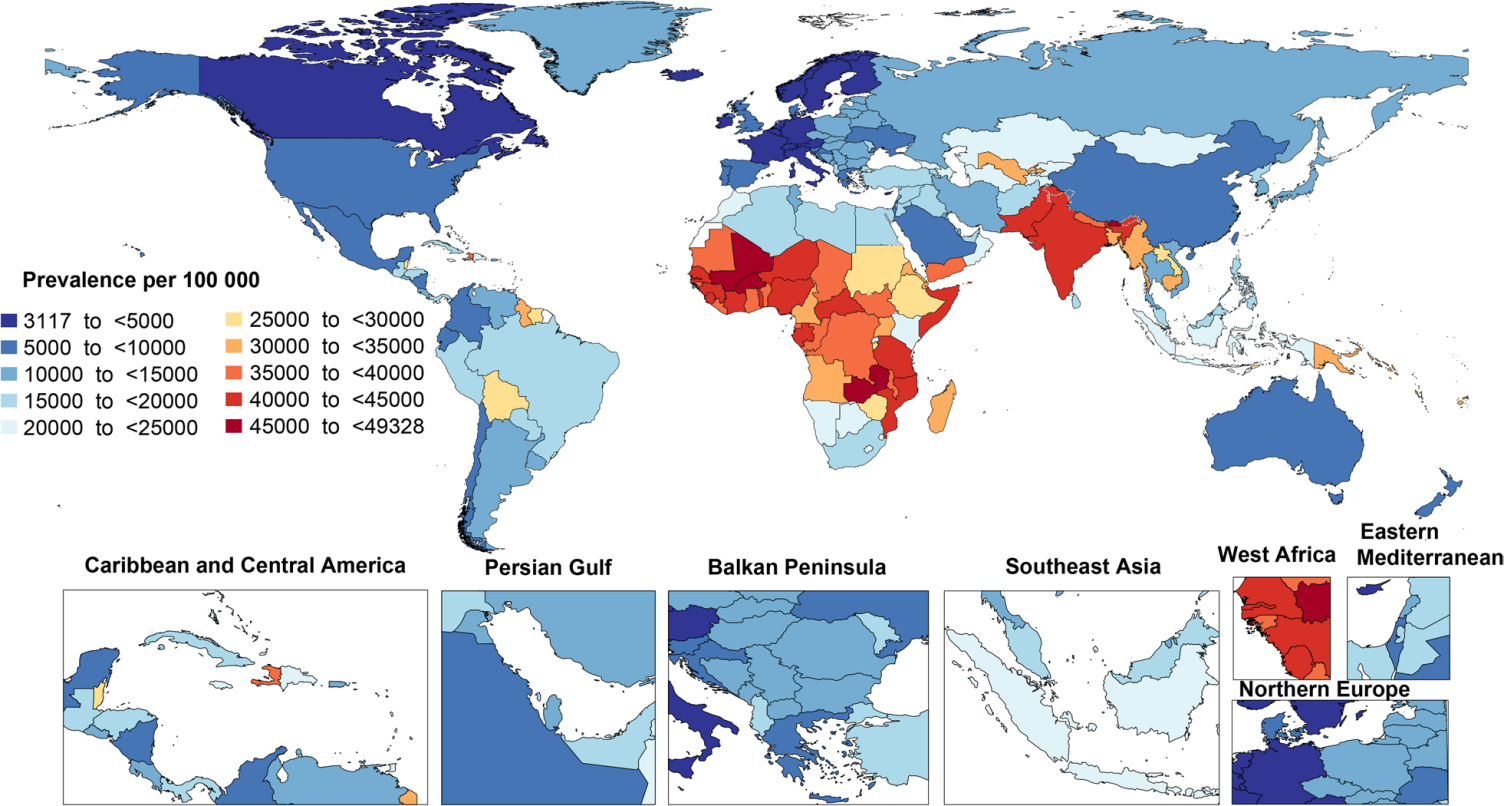
Age-standardized point prevalence of anemia per 100,000 population in 2019, by country. (Generated from data available from http://ghdx.healthdata.org/gbd-results-tool)

In 2019, the national age-standardized point prevalence of anemia ranged from 3118.0 to 49,327.1 cases per 100,000 population. Zambia [49327.1 (95% UI: 46,838.5–51,700.1)], Mali [46890.1 (95% UI: 44,301.1–49,389.8)], and Burkina Faso [46117.2 (95% UI: 43,640.7–48,319.2)] had the highest age-standardized point prevalence rates of anemia in 2019. In contrast, France [3118.0 (95% UI: 2341.9–4261.1)], Iceland [3622.1 (95% UI: 2747.8–4656.3)] and Belgium [3778.9 (95% UI: 2898.6–4873.0)] had the lowest rates (Fig. 1 and Additional file 8: Table S4).

**Fig. 2:**
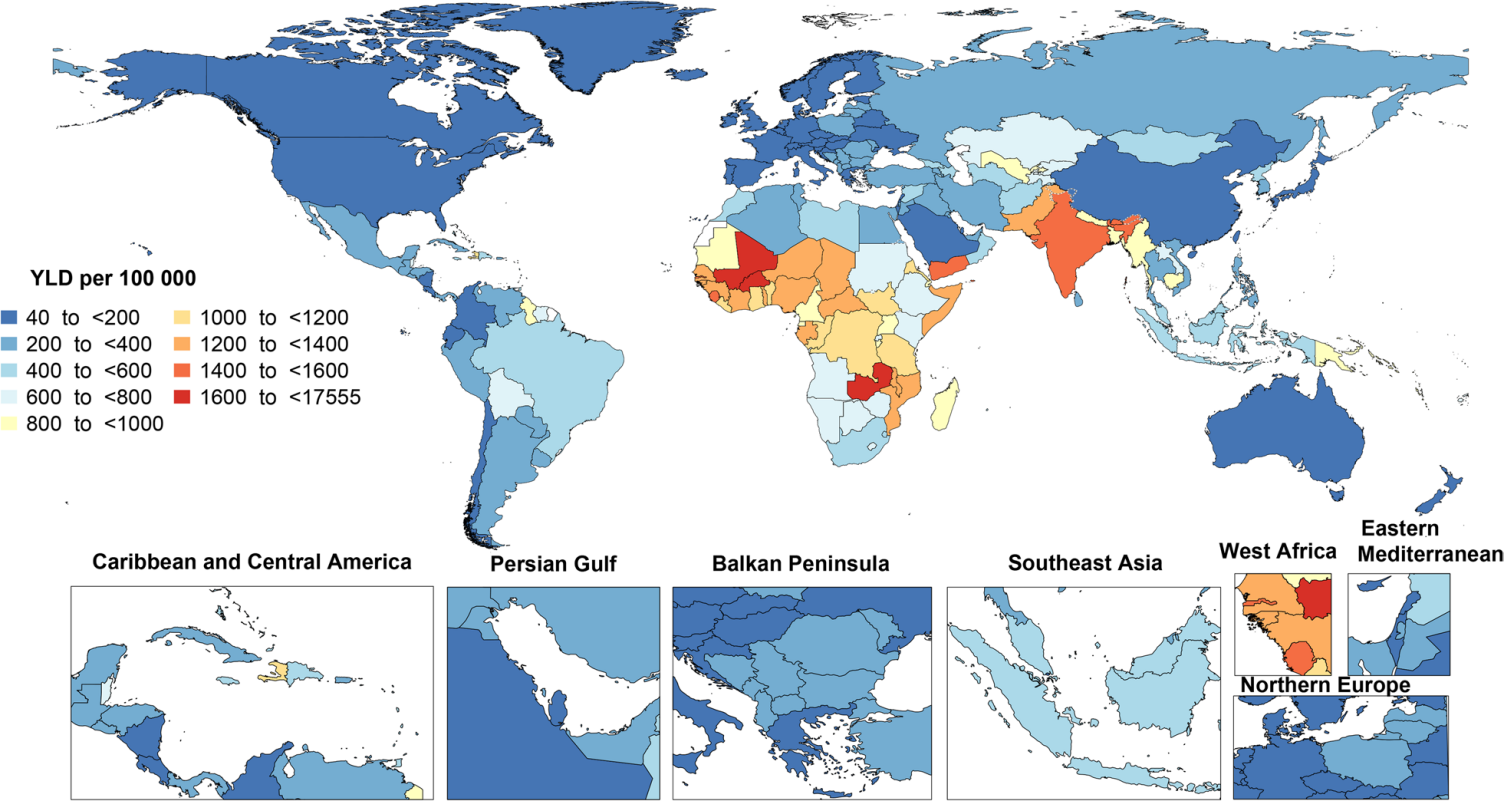
Age-standardized years lived with disability (YLDs) rate of anemia (per 100,000 population) in 2019, by country. (Generated from data available from http://ghdx.healthdata.org/gbd-results-tool)

The national age-standardized YLD rates of anemia varied in 2019 from 40.5 to 1754.3 cases per 100,000 population. The highest rates were observed in Zambia [1754.3 (95% UI: 1176.7–2517.7)], Mali [1681.7 (95% UI: 1110.1–2438.7)] and Burkina Faso [1620.0 (95% UI: 1077.6–2362.7)], while the lowest rate was found in France [40.5 (95% UI: 21.9–70.3)], Iceland [45.7 (95% UI: 24.6–77.2)] and the Netherlands [47.7 (95% UI: 25.6–82.2)] (Fig. 2 and Additional file 9: Table S5).

The percentage change in the age-standardized point prevalence, from 1990 to 2019, differed substantially between countries, but there was no country which demonstrated an increase during the measurement period. China [− 60.5% (95% UI: − 63.4 to − 57.4)], the Republic of Korea [− 55.8% (95% UI: − 62.4 to − 48.1)] and Ecuador [− 53.4% (95% UI: − 60.3 to − 45.8)] showed the largest decreases over the period 1990–2019 (Additional file 8: Table S4).

China [− 72.1% (95% UI: − 76.1 to − 67.8)], the Republic of Korea [− 68.2% (95% UI: − 74.4 to − 61.1)] and the Maldives [− 64.6% (95% UI: − 71.1 to − 57.6)] showed the largest decreases in the age-standardized YLD rates of anemia from 1990 to 2019, while there was no country whose YLD rate increased during this period (Additional file 9: Table S5). The prevalent cases and YLDs, due to anemia in 2019, and the percentage change in the age-standardized rates (ASRs) from 1990 to 2019, are reported by severity level for all countries and territories in Additional file 2: Table S2 and Additional file 3: Table S3.

### Age and sex patterns

**Fig. 3:**
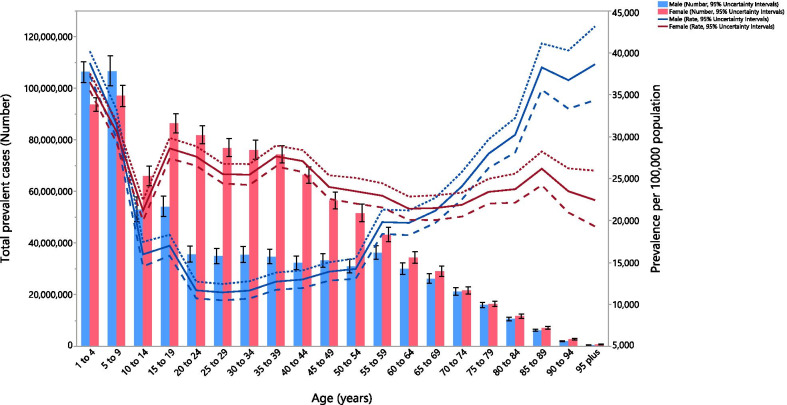
Global number of prevalent cases and the point prevalence of anemia per 100,000 population, by age and sex in 2019; Dotted and dashed lines indicate 95% upper and lower uncertainty intervals, respectively. (Generated from data available from http://ghdx.healthdata.org/gbd-results-tool)

In 2019, the global point prevalence of anemia was high among individuals aged less than 10 years old, for both males and females. Following this, there was a decrease in the point prevalence up to the 25–29 age group in males, before increasing again with advancing age. For females, the point prevalence reached its lowest level in the 10–14 age group, before peaking in the 15–19 age group and generally decreasing with increasing age. The global point prevalence was higher in females, than among males, in the 10–69 age groups. The global number of prevalent cases peaked in the 5–9 years age group and was higher in females in all age groups, except those aged 1–9 years (Fig. 3).

In males, the global YLD rate reached its highest level in the 5–9 age group and then decreased up to the 25–29 age group, before broadly increasing with advancing age. In females, the global YLD rate peaked in the 5–9 age group and then generally decreased with advancing age. The YLD rate was higher in females than among males in the 10–84 age groups. The global number of YLDs peaked in the 5–9 age group and was higher in females across all age groups, except for the 1–9 age groups (Additional file 10: Fig. S5).

### Association with the socio-demographic index (SDI)

**Fig. 4:**
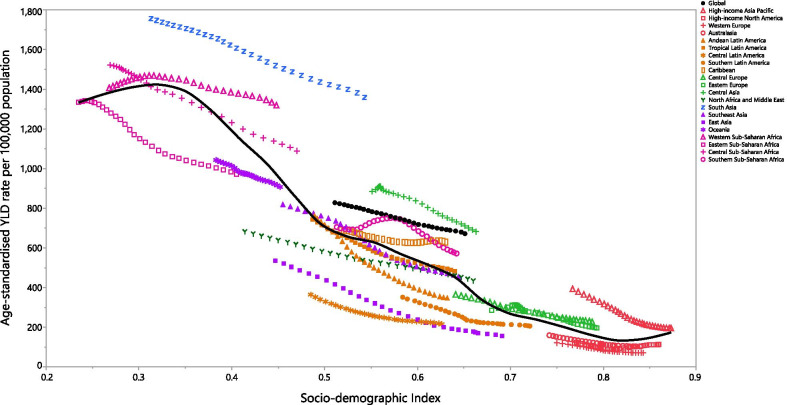
Age-standardized YLD rates of anemia for the 21 Global Burden of Disease regions by Socio-demographic Index, 1990–2019; Expected values based on Socio-demographic Index and disease rates in all locations are shown as the black line. Thirty points are plotted for each GBD region and show the observed age-standardized YLD rates from 1990 to 2019 for that region. YLD = years lived with disability. (Generated from data available from http://ghdx.healthdata.org/gbd-results-tool)

At the regional level, there was a negative association between SDI and the age-standardized YLD rate of anemia, suggesting that the burden of anemia was lower in regions with higher socio-economic development. South Asia, Western Sub-Saharan Africa, Central Asia, Southern Sub-Saharan Africa, the Caribbean and High-income Asia Pacific all had higher than expected YLD rates from 1990 to 2019, based upon their level of socio-demographic development (as measured by the SDI). In contrast, Eastern Sub-Saharan Africa, Oceania, Southern Latin America, East Asia, Central Latin America, Australasia, Western Europe and High-income North America all had lower than expected burdens across the measurement period (Fig. 4).

At the country-level, in 2019 the burden of anemia generally decreased with increasing socio-economic development (Fig. 4). Countries and territories, such as Zambia, Mali, Burkina Faso, Bhutan, India, Yemen and Gambia had much higher than expected burdens, whereas countries and territories, such as France, Italy, Greece, Colombia, El Salvador, Honduras and Rwanda all had much lower than expected burdens (Additional file 11: Fig. S6).

### Underlying causes

**Fig. 5:**
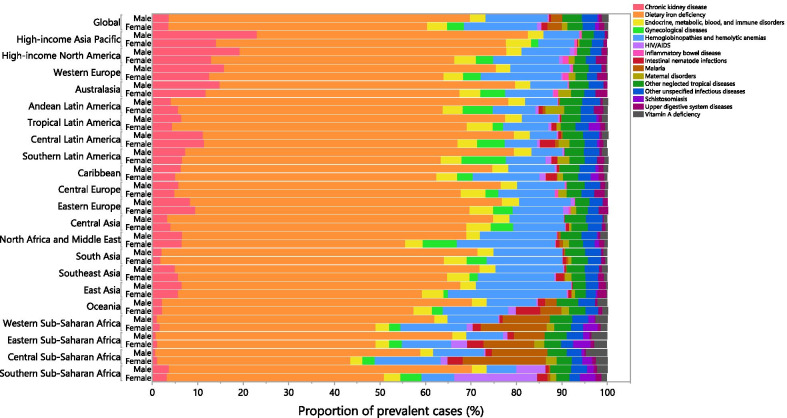
Proportion of prevalent cases of anemia attributable to each underlying cause by sex and Global Burden of Disease region in 2019. (Generated from data available from http://ghdx.healthdata.org/gbd-results-tool)

Although there were sex and country differences in the proportion of prevalent cases of anemia attributable to the underlying causes, globally the most prevalent cases were accounted for by dietary iron deficiency (males: 66.1%, females: 56.8%), as well as hemoglobinopathies and hemolytic anemias (male: 13.6%, female:16.1%). The lowest and highest proportion of prevalent cases attributable to dietary iron deficiency were seen in Western Sub-Saharan Africa (male: 61.0%, female: 47.5%) and Andean latin America (male: 74.1%, female: 58.1%), respectively. In addition, the lowest and highest proportion of the prevalent cases attributable to hemoglobinopathies and hemolytic anemias were seen in East Asia (20.9%) and Central Latin America (5.9%) for males and in East Asia (26.1%) and Southern Sub-Saharan Africa (7.2%) for females (Fig. 5).

**Fig. 6:**
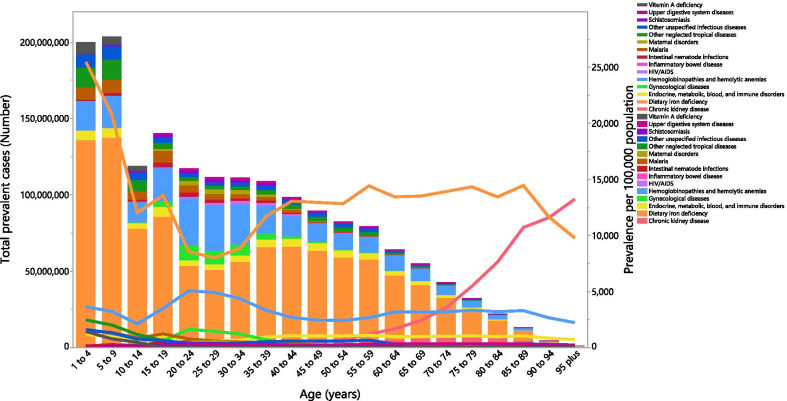
Global number of prevalent cases and point prevalence due to anemia per 100,000 population attributable to each underlying cause by age in 2019. (Generated from data available from http://ghdx.healthdata.org/gbd-results-tool)

In 2019, the global point prevalence of anemia that was attributable to dietary iron deficiency was higher than all other causes, in all age groups except the 95+ age group. The peak was reached in the 1–4 age group and then generally decreased up to the 25–29 age group, then broadly increased up to the 85–89 age group, before decreasing over the remaining age groups. In addition, the global point prevalence attributable to hemoglobinopathies and hemolytic anemias accounted for the second largest proportion, which peaked in the 20–24 age group and then decreased up to the 50–54 age group, increased up to the 85–89 age group, before decreasing in the last two age groups. The global point prevalence attributable to chronic kidney disease started to increase in the 50–54 age group and reached its peak in the oldest age group. The number of prevalent cases attributable to dietary iron deficiency and hemoglobinopathies and hemolytic anemias accounted for the largest proportions in almost all age groups (Fig. 6).

At the regional level, the association between SDI and the age-standardized YLD rates of anemia showed similar patterns for all underlying causes (a decrease with increasing SDI), except for inflammatory bowel disease, which showed the opposite trend (Additional file 12: Fig. S7, Additional file 13: Fig. S8, Additional file 14: Fig. S9, Additional file 15: Fig. S10, Additional file 16: Fig. S11, Additional file 17: Fig. S12, Additional file 18: Fig. S13, Additional file 19: Fig. S14, Additional file 20: Fig. S15, Additional file 21: Fig. S16, Additional file 22: Fig. S17, Additional file 23: Fig. S18, Additional file 24: Fig. S19, Additional file 25: Fig. S20, Additional file 26: Fig. S21).

## Discussion

The present study reported the burden of anemia and its attributable underlying causes at the global, regional and national levels using GBD 2019 data. In 2019, approximately 1.8 billion individuals lived with anemia and this illness accounted for nearly 50.3 million YLDs across the world. The age-standardized point prevalence and YLD rates have decreased over the past three decades, which might be as a result of better access to healthcare services, in terms of improvements in screening, prevention and the treatment of anemia. However, the absolute number of prevalent cases and YLDs have increased, which may be primarily due to population aging and population growth, as well as improvements in the survival of patients with long-term conditions known to cause anemia, such as chronic kidney disease and hemoglobinopathies. These trends demonstrate that anemia still contributes a substantial health burden globally, and that its overall burden may continue to rise.

Although a direct comparison to the GBD 2010 [8] and GBD 2013 [10] is not possible, due to variations in data sources and methodological differences, several comparable results were observed. The decrease observed in the age-standardized point prevalence of anemia (7.3%) from 1990 to 2010 was relatively consistent with our finding, where a 13.4% reduction was observed from 1990 to 2019. In addition, South Asia and the Western, Eastern and Central Sub-Saharan Africa regions had the highest point prevalence of anemia, which was also in accordance with the GBD 2010 findings [8]. However, while South Asia represented the highest point prevalence of anemia in 2019, this region ranked fourth highest in 2010 [8].

Our results showed that children under 10 years of age endure the largest burden of anemia across the world. Similar findings were also reported by the 2005 WHO Vitamin and Mineral Nutrition Information System study, where pre-school children had the highest prevalence of anemia globally [17]. As with other age groups, iron deficiency constitutes the leading cause of anemia in children under 10 years old [18, 19]. In young children, iron deficiency is associated with adverse psychomotor, cognitive and socioemotional development [20]; with longitudinal studies demonstrating long-lasting adverse effects on behavior and development in adolescence, even if the deficiency has been corrected [21–23]. Rich sources of dietary iron include lean meat and seafood, with nonheme dietary sources including nuts, beans, vegetables and fortified grain products. However, it is evident that low dietary intakes and a high prevalences of deficiency occur in both low and higher income countries [24, 25]. One of the guidelines from The World Health Organization is the use of multiple micronutrient powders for the point-of-use fortification of foods consumed by infants and young children aged 6–23 months and children aged 2–12 years [26]. Accordingly, the introduction of iron fortified infant formulas by the American Academy of Pediatrics, for use during the first year of infancy, was a major public health success, with the prevalence of iron deficiency anemia dropping from more than 20% to less than 3% in about 2 decades [27]. Anemia prevention and control strategies have been described, and include: improved dietary intake and increased food diversity with increased iron bioavailability; targeted fortification of foods for high-risk groups (e.g., in infant formula); and iron (and folic acid) supplementation (tablets, powders, spreads) for high-risk groups, such as children and adolescents [25]. However, barriers exist regarding the dissemination and uptake of these programs, including insufficient priority; lack of knowledge and education around anemia prevention; and the difficulty in meeting the needs of high-risk groups at specific times in their lives [25]. Given that the early years of life are a critical period for mental and psychomotor development, the prevention of anemia should continue to be supported, encouraged and adopted.

The present study found that that the prevalence of anemia was higher among male children younger than 10 years old, compared to females in the same age group. In line with our findings, several studies have reported that male infants and young children have lower iron stores, which may put them at greater risk of iron deficiency anemia [8, 28–30]. Although there is no definitive explanation for this phenomena, it may be that the hormonal effects on erythropoietin activity and the higher pre- and postnatal growth rate may play a role in the increased susceptibility of male children to anemia [28, 29]. Conversely, other studies have demonstrated girls to have higher rates of anemia than boys [31, 32]. Reverse causation may partly explain the relationship between anemia in boys and girls. That is, public health messages consistently highlight girls as a high risk group, thus mothers of young girls may prioritize their dietary iron needs over that of boys, leading to higher rates of iron sufficiency in girls but higher rates of deficiency in boys.

As documented here, and in previous studies [8, 10], sex differences in the burden of anemia were expected, with a higher prevalence of anemia in women, than among men of the same age, throughout adulthood. Regular blood loss, due to menstrual bleeding, along with pregnancy-related complications over the childbearing ages, deplete iron stores and therefore iron requirements are increased. Globally, anemia in women of reproductive age remains the most challenging nutritional problem. One of the World Health Organization guidelines recommend a daily 30–60 mg elemental iron supplement for adult women and adolescent girls [33], given their high risk of deficiency. In 2012, the World Health Assembly endorsed the goal of reducing the prevalence of anemia by 50% in women of reproductive age by 2025 [34]. The prevalence of anemia was found to have decreased by 4% (33% in 1995 to 29% in 2011) for non-pregnant women and by 5% (43% in 1995 to 38% in 2011) for pregnant women across the globe [9]. Consequently, reaching the World Health Assembly goal by 2025 will require more integrated efforts in the coming years, particularly in the areas of the world where anemia is more prevalent.

The present research showed that the point prevalence and YLD rates increased gradually with age for both sexes, albeit to a more pronounced degree among men. This finding is comparable with previous research, which also found that the prevalence of anemia increased with advancing age in adults aged over 50 years, with males more likely than females to develop anemia at an older age [35]. Furthermore, chronic kidney disease, which impairs the kidneys’ ability to produce sufficient erythropoietin, was found to be one of the leading causes of anemia from the sixth decade of life onwards. It has also been shown that at reduced levels of renal function, men had a greater decrease in Hb concentration than women [36]. Moreover, it is believed that the declining Hb concentration among the elderly could be due to the reduced number of bone marrow erythroid progenitors, which is also more evident in elderly men than among elderly women [37].

Inherited Hb disorders, such as thalassemia, sickle cell trait or hemolytic anemia were the second most common cause of anemia across the globe, particularly in East Asia. Improvements in the treatment of Hb disorders have resulted in falling mortality rates among affected children under 5 years of age [38]. Therefore, many children with thalassemia or sickle cell trait disease that would have otherwise died during infancy, are currently alive due to improved diagnosis and disease management. This pattern is now evident in many Asian countries [39]. The more treatment measures that are improved, the more patients will reach adulthood, manifesting increased complications, such as anemia [40]. In parallel with treating those affected by hemoglobonopathies, it is important to educate the population about the likelihood of having children at increased risk of the disease. In this regard, carrier screening and genetic counselling are necessary for at risk couples [41].

According to our findings, malaria was among the leading causes of anemia in malaria endemic areas, especially in Western, Eastern and Central sub-Saharan Africa, where there is also a high prevalence of anemia among males and females. A previous review revealed that in the malaria endemic areas of Africa, malaria control for children increased Hb concentrations by a mean 7.6 g/L and reduced the risk of mild and severe anemia by 27% and 60%, respectively [42]. Iron supplementation programs in malaria endemic areas must be implemented with caution, because while iron supplementation for children may reduce clinical malaria in areas where prevention and management services are provided, it may subsequently increase the incidence of malaria in areas where such services are unavailable [43]. The WHO recommends the provision of iron supplementation for children in malaria endemic areas to be paired with measures for the prevention, diagnosis and treatment of malaria [44]. The combination of these approaches could provide synergistic benefits in reducing the burden of anemia [44].

A negative relationship was observed between the degree of development and the burden of anemia, at both the regional and national levels. Low SDI is an important indicator for a range of socio demographic and lifestyle variables, including poor nutritional status, inadequate access to health services, lack of education and low gender equality. In this regard, anemia is potentially linked to poor nutritional behaviors, food insecurity and poor quality food products. A recent review revealed that an inadequate intake of micronutrients, especially iron, folate and zinc, is a common problem among women from low and middle income countries [45]. Vitamin A deficiency is a common cause of anemia in many poor countries [29, 46–48]. In 2013, it was estimated that sub-Saharan Africa and South Asia had the highest prevalence of vitamin A deficiency, which were 48% and 44%, respectively [46, 47]. Furthermore, limited access to healthcare services may also contribute to the increased risk of developing anemia. Resource deprived settings, where access to anemia prevention, screening and treatment services are low, together with poor access to iron supplementation and food fortification technology are all likely to be associated with a high anemia burden and to be found in countries with low SDIs. Additionally, educational status is another important determinant of health, with research suggesting that women with no education have an 8% higher risk of anemia and their children are 9% more likely to become affected by anemia, in comparison with those having secondary or higher education [25]. Finally, another important variable is gender inequality, which minimizes the empowerment of women, reduces their access to education, reduces nutritional literacy, restricts household income, leads to early marriage, a high frequency of pregnancies and having children during adolescence, which are all potential explanations for the observed negative association between the level of development and the odds of having anemia [49].

Anemia is still a clear public health burden and a public health issue. Multiple factors contribute to anemia, highlighting the need for an integrated approach to address anemia; descriptions of interventions and programs have been described elsewhere [18, 26]. Briefly, micronutrient supplementation programs have so far been successful in reducing rates of anemia [50]. A review of randomized clinical trials in children, non-pregnant and pregnant women showed that iron supplementation significantly improved Hb concentrations [51–53]. Children consuming iron-containing multiple micronutrient powders, that also included vitamin A and zinc, were also less likely to have iron deficiency, albeit there is weaker evidence for reduced likelihood of anemia [54]. The rationale for additional micronutrients like vitamin A, zinc and folic acid are due to their general low rates of sufficiency, particularly in children of developing countries [46, 55], but also due to their contributing relationship to anemia. Despite the general positive benefits of these interventions, the uptake is low [48], there is low adherence by the targeted population [47], and higher quality evidence is needed. Fortification of commonly consumed food products, such as staple foods and condiments which are both inexpensive and readily available, has also been shown to successful [56]. A systematic review of randomized controlled trials revealed that iron-fortified wheat flour, with or without other micronutrients, reduced the risk of anemia by 27% [57]; however, its effect on other outcomes requires further investigation. For pregnant women, the WHO recommends a daily 300 µg iron and 400 µg folic acid supplement [58]. Iron supplementation studies have demonstrated a reduced risk of maternal anemia at term by 70% and iron deficiency at term by 57%; and women receiving iron also had higher levels of Hb per litre at or near term than women not receiving supplements [59]. Iron and folic acid fortification studies have also demonstrated increased serum ferritin and Hb levels in women of reproductive age and in pregnant women and reduced neural tube defects [60]. Bridging the gap between higher and lower income countries; improved understanding on cost and resource implications; careful identification of the strategy to reach the target populations; and further engagement with key public health stakeholders may contribute to reduced rates of anemia.

### Strengths and limitations

We acknowledge that our study has some limitations that should be taken into consideration when interpretating the results. Firstly, there was a sparcity of data, particularly in countries with lower socioeconomic status and with minimal data sources. This can greatly affect the estimated burden. Improvements in the collection of data and in data sharing policies will help to increase the precision of the estimates. Secondly, the burden of disease associated with each type of anemia (e.g., iron deficiency anemia, sickle cell anemia, aplastic anemia and megaloblastic anemia) could not be reported in the present study. As each type may not have a similar etiology or risk factors, it may be difficult to provide specific preventive recommendations at a global level. Therefore, future GBD studies should report the burden of anemia by type. Thirdly, a lack of data on a number of other potential risk factors for anemia, which are categorized as “other neglected tropical diseases” and “other unspecified infectious diseases,” hinders the explanation of the associations. Nevertheless, the present study presents the most recent estimates on the burden of anemia and its causes, which will be useful for public health policy makers.

## Conclusions

Anemia is a major public health issue across the world, but varies considerably by country. Although the age standardized point prevalence and YLD rates due to anemia decreased from 1990 to 2019, its burden remains high, particularly in less developed countries. Given the increasing aging of the global population, there is likely to be increasing numbers of individuals living with anemia. Controlling the burden of anemia necessitates the formulation of integrated interventions which prioritize the highest risk groups, including young children and women of reproductive age. Despite the multifactorial nature of the disease, dietary iron deficiency remains the leading cause of anemia in all regions. Therefore, more attention must be paid to nutritional interventions and then to controlling the burden of hemoglobinopathies, parasitic infections and chronic diseases.

## Supplementary Information

**Additional file 1: Table S1.** Definitions of mild, moderate, and severe anemia, based on blood hemoglobin concentration.

**Additional file 2: Table S2.** Prevalent cases of anemia in 2019 and the percentage change in the age-standardized rates (ASRs) from 1990 to 2019, by severity and location (Generated from data available from http://ghdx.healthdata.org/gbd-results-tool).

**Additional file 3: Table S3.** Years lived with disability (YLDs) due to anemia in 2019 and the percentage change in the age-standardized rates (ASRs) from 1990 to 2019, by severity and location (Generated from data available from http://ghdx.healthdata.org/gbd-results-tool).

**Additional file 4: Figure S1.** The age-standardized point prevalence of anemia in 2019 for the 21 Global Burden of Disease regions, by sex. (Generated from data available from http://ghdx.healthdata.org/gbd-results-tool).

**Additional file 5: Figure S2.** The age-standardized years lived with disability (YLDs) rates of anemia in 2019 for the 21 Global Burden of Disease regions, by sex. (Generated from data available from http://ghdx.healthdata.org/gbd-results-tool).

**Additional file 6: Figure S3.** The percentage change in the age-standardized point prevalence of anemia from 1990 to 2019 for the 21 Global Burden of Disease regions, by sex. (Generated from data available from http://ghdx.healthdata.org/gbd-results-tool).

**Additional file 7: Figure S4.** The percentage change in the age-standardized years lived with disability (YLDs) rates of anemia from 1990 to 2019 for the 21 Global Burden of Disease regions, by sex. (Generated from data available from http://ghdx.healthdata.org/gbd-results-tool).

**Additional file 8: Table S4.** Prevalent cases of anemia in 1990 and 2019 and the percentage change in the age-standardized rates (ASRs) per 100,000, by location (Generated from data available from http://ghdx.healthdata.org/gbd-results-tool).

**Additional file 9: Table S5.** Years lived with disability (YLDs) due to anemia in 1990 and 2019 and the percentage change in the age-standardized rates (ASRs) per 100,000, by location (Generated from data available from http://ghdx.healthdata.org/gbd-results-tool).

**Additional file 10: Figure S5.** Global number of years lived with disability (YLDs) cases and years lived with disability (YLDs) of anemia per 100,000 population, by age and sex in 2019; Dotted and dashed lines indicate 95% upper and lower uncertainty intervals, respectively. (Generated from data available from http://ghdx.healthdata.org/gbd-results-tool).

**Additional file 11: Figure S6.** Age-standardized YLD rates of anemia for 204 countries and territories by Socio-demographic Index, in 2019; Expected values based on the Socio-demographic Index and disease rates in all locations are shown as the black line. Each point shows the observed age-standardized YLD rate for each country in 2019. YLD = years lived with disability. (Generated from data available from http://ghdx.healthdata.org/gbd-results-tool).

**Additional file 12: Figure S7.** Age-standardized YLD rates of anemia attributable to chronic kidney disease for the 21 Global Burden of Disease regions by Socio-demographic Index, 1990–2019; Expected values based on Socio-demographic Index and disease rates in all locations are shown as the black line. Thirty points are plotted for each GBD region and show the observed age-standardized YLD rates from 1990 to 2019 for that region. YLD = years lived with disability. (Generated from data available from http://ghdx.healthdata.org/gbd-results-tool).

**Additional file 13: Figure S8.** Age-standardized YLD rates of anemia attributable to dietary iron deficiency for the 21 Global Burden of Disease regions by Socio-demographic Index, 1990–2019; Expected values based on Socio-demographic Index and disease rates in all locations are shown as the black line. Thirty points are plotted for each GBD region and show the observed age-standardized YLD rates from 1990 to 2019 for that region. YLD = years lived with disability. (Generated from data available from http://ghdx.healthdata.org/gbd-results-tool).

**Additional file 14: Figure S9.** Age-standardized YLD rates of anemia attributable to endocrine, metabolic, blood, and immune disorders for the 21 Global Burden of Disease regions by Socio-demographic Index, 1990–2019; Expected values based on Socio-demographic Index and disease rates in all locations are shown as the black line. Thirty points are plotted for each GBD region and show the observed age-standardized YLD rates from 1990 to 2019 for that region. YLD = years lived with disability. (Generated from data available from http://ghdx.healthdata.org/gbd-results-tool).

**Additional file 15: Figure S10.** Age-standardized YLD rates of anemia attributable to gynecological diseases for the 21 Global Burden of Disease regions by Socio-demographic Index, 1990–2019; Expected values based on Socio-demographic Index and disease rates in all locations are shown as the black line. Thirty points are plotted for each GBD region and show the observed age-standardized YLD rates from 1990 to 2019 for that region. YLD = years lived with disability. (Generated from data available from http://ghdx.healthdata.org/gbd-results-tool).

**Additional file 16: Figure S11.** Age-standardized YLD rates of anemia attributable to hemoglobinopathies and hemolytic anemias for the 21 Global Burden of Disease regions by Socio-demographic Index, 1990–2019; Expected values based on Socio-demographic Index and disease rates in all locations are shown as the black line. Thirty points are plotted for each GBD region and show the observed age-standardized YLD rates from 1990 to 2019 for that region. YLD = years lived with disability. (Generated from data available from http://ghdx.healthdata.org/gbd-results-tool).

**Additional file 17: Figure S12.** Age-standardized YLD rates of anemia attributable to HIV/AIDS for the 21 Global Burden of Disease regions by Socio-demographic Index, 1990–2019; Expected values based on Socio-demographic Index and disease rates in all locations are shown as the black line. Thirty points are plotted for each GBD region and show the observed age-standardized YLD rates from 1990 to 2019 for that region. YLD = years lived with disability, HIV= human immunodeficiency virus, AIDS: acquired immunodeficiency syndrome (Generated from data available from http://ghdx.healthdata.org/gbd-results-tool).

**Additional file 18: Figure S13.** Age-standardized YLD rates of anemia attributable to inflammatory bowel disease for the 21 Global Burden of Disease regions by Socio-demographic Index, 1990–2019; Expected values based on Socio-demographic Index and disease rates in all locations are shown as the black line. Thirty points are plotted for each GBD region and show the observed age-standardized YLD rates from 1990 to 2019 for that region. YLD = years lived with disability. (Generated from data available from http://ghdx.healthdata.org/gbd-results-tool).

**Additional file 19: Figure S14.** Age-standardized YLD rates of anemia attributable to intestinal nematode infections for the 21 Global Burden of Disease regions by Socio-demographic Index, 1990–2019; Expected values based on Socio-demographic Index and disease rates in all locations are shown as the black line. Thirty points are plotted for each GBD region and show the observed age-standardized YLD rates from 1990 to 2019 for that region. YLD = years lived with disability. (Generated from data available from http://ghdx.healthdata.org/gbd-results-tool).

**Additional file 20: Figure S15.** Age-standardized YLD rates of anemia attributable to malaria for the 21 Global Burden of Disease regions by Socio-demographic Index, 1990–2019; Expected values based on Socio-demographic Index and disease rates in all locations are shown as the black line. Thirty points are plotted for each GBD region and show the observed age-standardized YLD rates from 1990 to 2019 for that region. YLD = years lived with disability. (Generated from data available from http://ghdx.healthdata.org/gbd-results-tool).

**Additional file 21: Figure S16.** Age-standardized YLD rates of anemia attributable to maternal disorders for the 21 Global Burden of Disease regions by Socio-demographic Index, 1990–2019; Expected values based on Socio-demographic Index and disease rates in all locations are shown as the black line. Thirty points are plotted for each GBD region and show the observed age-standardized YLD rates from 1990 to 2019 for that region. YLD = years lived with disability. (Generated from data available from http://ghdx.healthdata.org/gbd-results-tool).

**Additional file 22: Figure S17.** Age-standardized YLD rates of anemia attributable to “other neglected tropical diseases” for the 21 Global Burden of Disease regions by Socio-demographic Index, 1990–2019; Expected values based on Socio-demographic Index and disease rates in all locations are shown as the black line. Thirty points are plotted for each GBD region and show the observed age-standardized YLD rates from 1990 to 2019 for that region. YLD = years lived with disability. (Generated from data available from http://ghdx.healthdata.org/gbd-results-tool).

**Additional file 23: Figure S18.** Age-standardized YLD rates of anemia attributable to “other unspecified infectious diseases” for the 21 Global Burden of Disease regions by Socio-demographic Index, 1990–2019; Expected values based on Socio-demographic Index and disease rates in all locations are shown as the black line. Thirty points are plotted for each GBD region and show the observed age-standardized YLD rates from 1990 to 2019 for that region. YLD = years lived with disability. (Generated from data available from http://ghdx.healthdata.org/gbd-results-tool).

**Additional file 24: Figure S19.** Age-standardized YLD rates of anemia attributable to schistosomiasis for the 21 Global Burden of Disease regions by Socio-demographic Index, 1990–2019; Expected values based on Socio-demographic Index and disease rates in all locations are shown as the black line. Thirty points are plotted for each GBD region and show the observed age-standardized YLD rates from 1990 to 2019 for that region. YLD = years lived with disability. (Generated from data available from http://ghdx.healthdata.org/gbd-results-tool).

**Additional file 25: Figure S20.** Age-standardized YLD rates of anemia attributable to upper digestive system diseases for the 21 Global Burden of Disease regions by Socio-demographic Index, 1990–2019; Expected values based on Socio-demographic Index and disease rates in all locations are shown as the black line. Thirty points are plotted for each GBD region and show the observed age-standardized YLD rates from 1990 to 2019 for that region. YLD = years lived with disability. (Generated from data available from http://ghdx.healthdata.org/gbd-results-tool).

**Additional file 26: Figure S21.** Age-standardized YLD rates of anemia attributable to vitamin A deficiency for the 21 Global Burden of Disease regions by Socio-demographic Index, 1990–2019; Expected values based on Socio-demographic Index and disease rates in all locations are shown as the black line. Thirty points are plotted for each GBD region and show the observed age-standardized YLD rates from 1990 to 2019 for that region. YLD = years lived with disability. (Generated from data available from http://ghdx.healthdata.org/gbd-results-tool).

## Data Availability

The data used for these analyses are all publicly available at http://ghdx.healthdata.org/gbd-results-tool.
