# Supplementary material for: Burden of anemia and its underlying causes in 204 countries and territories, 1990–2019: results from the Global Burden of Disease Study 2019
Source: J Hematol Oncol. 2021 Nov 4;14:185. doi: 10.1186/s13045-021-01202-2 (PMC8567696; doi:10.1186/s13045-021-01202-2)
Supplement: Supplementary file 1 — Additional file 1: Table S1. Definitions of mild, moderate, and severe anemia, based on blood hemoglobin concentration. [file 13045_2021_1202_MOESM1_ESM.docx]

| **Additional file 1: Table S1. Definitions of mild, moderate, and severe anaemia, based on blood haemoglobin concentration** | | | | |
| --- | --- | --- | --- | --- |
| **Sex** | **Age** | **Mild (g/L)** | **Moderate (g/L)** | **Severe (g/L)** |
| Both | <28 days | 130 to 149 | 90 to 129 | < 90 |
| Both | 1 month to 4 years | 100 to 109 | 70 to 99 | < 70 |
| Both | 5 to 14 years | 110 to 114 | 80 to 109 | < 80 |
| Male | 15^+^ years | 110 to 129 | 80 to 109 | < 80 |
| Female, non-pregnant | 15^+^ years | 110 to 119 | 80 to 109 | < 80 |
| Female, pregnant | 15^+^ years | 100 to 109 | 70 to 99 | < 70 |
