# Supplementary material for: Burden of anemia and its underlying causes in 204 countries and territories, 1990–2019: results from the Global Burden of Disease Study 2019
Source: J Hematol Oncol. 2021 Nov 4;14:185. doi: 10.1186/s13045-021-01202-2 (PMC8567696; doi:10.1186/s13045-021-01202-2)
Supplement: Supplementary file 2 — Additional file 2: Table S2. Prevalent cases of anemia in 2019 and the percentage change in the age-standardized rates (ASRs) from 1990 to 2019, by severity and location (Generated from data available from http://ghdx.healthdata.org/gbd-results-tool). [file 13045_2021_1202_MOESM2_ESM.doc]

| **Additional file 2: Table S2. Prevalent cases of anemia in 1990 and 2019 and the percentage change in the age-standardised rates (ASRs) per 100,000 by severity and location**  **(Generated from data available from http://ghdx.healthdata.org/gbd-results-tool)** | | | | | | | | | |
| --- | --- | --- | --- | --- | --- | --- | --- | --- | --- |
|  | **Mild** | | | **Moderate** | | | **Severe** | | |
|  | **No (95% UI)**  **2019** | **ASRs per 100,000 (95% UI)**  **2019** | **Percentage change in ASRs per 100,000**  **(1990 - 2019)** | **No (95% UI)**  **2019** | **ASRs per 100,000 (95% UI)**  **2019** | **Percentage change in ASRs per 100,000**  **(1990 - 2019)** | **No (95% UI)**  **2019** | **ASRs per 100,000 (95% UI)**  **2019** | **Percentage change in ASRs per 100,000**  **(1990 - 2019)** |
| **Global** | **954311494 (944960116 , 963999903)** | **12349.8 (12230.7 , 12472.9)** | **-10 (-11 , -8.8)** | **747799875 (737546863 , 758151574)** | **10035.3 (9891.2 , 10173.5)** | **-15.2 (-16.6 , -13.5)** | **59450583 (57816053 , 61200498)** | **791.1 (768 , 815)** | **-34.1 (-36.6 , -31.4)** |
| **High-income North America** | **21123354 (18996232 , 23431959)** | **5195.6 (4636.2 , 5798.6)** | **-10 (-20.9 , 4.4)** | **6448197 (5533857 , 7578685)** | **1667.8 (1419 , 1984.9)** | **-10.4 (-26.2 , 10.5)** | **249283 (198066 , 312575)** | **56.6 (44.5 , 72.7)** | **6.5 (-20.8 , 41.1)** |
| **Canada** | **1353186 (1104280 , 1637104)** | **3258.9 (2621.1 , 3939.5)** | **-25.9 (-42.6 , -5.2)** | **358585 (272437 , 454901)** | **931.1 (692.5 , 1206.3)** | **-33.5 (-52.6 , -8.2)** | **11446 (8025 , 15933)** | **26.5 (17.9 , 37.4)** | **-42.3 (-63.6 , -8.8)** |
| **Greenland** | **4239 (3644 , 4879)** | **7359.9 (6296.6 , 8499.8)** | **-20.8 (-34.5 , -5.5)** | **1589 (1290 , 1952)** | **2894.9 (2319.1 , 3592)** | **-32.2 (-47.2 , -11.2)** | **70 (52 , 94)** | **121.3 (90.9 , 160.4)** | **-39.8 (-57 , -13.2)** |
| **United States of America** | **19765593 (17650802 , 22117911)** | **5410.2 (4792.9 , 6069.5)** | **-8.5 (-20.3 , 6.2)** | **6087921 (5180560 , 7187465)** | **1749.4 (1473.7 , 2104)** | **-8.4 (-25.9 , 14.2)** | **237763 (187961 , 301003)** | **60.1 (46.9 , 78.2)** | **11.5 (-18.9 , 50)** |
| **Australasia** | **1503843 (1280607 , 1746995)** | **5201.7 (4334.4 , 6207.5)** | **-24.9 (-39.9 , -6.9)** | **426197 (344177 , 531472)** | **1528.7 (1198.8 , 2000.7)** | **-36.8 (-54.5 , -12.3)** | **12155 (9254 , 16107)** | **35.4 (26.2 , 47.5)** | **-47.6 (-63.6 , -24.4)** |
| **Australia** | **1259404 (1044193 , 1503156)** | **5115.7 (4130.6 , 6241.9)** | **-26 (-42.8 , -4.9)** | **348063 (268745 , 451945)** | **1455.9 (1076.5 , 1992.4)** | **-38.1 (-57.5 , -10.5)** | **9900 (7100 , 13548)** | **33.7 (23.8 , 47.7)** | **-48.6 (-67 , -21)** |
| **New Zealand** | **244439 (204581 , 284931)** | **5615.5 (4591.5 , 6698.9)** | **-19.6 (-35.5 , -0.1)** | **78134 (59470 , 103794)** | **1897.9 (1357.8 , 2666.7)** | **-30.5 (-54.1 , 3.3)** | **2255 (1633 , 3045)** | **44 (30.9 , 61.7)** | **-42.9 (-62.8 , -14.5)** |
| **High-income Asia Pacific** | **18455473 (16913813 , 20144406)** | **8030.6 (7280.7 , 8913.3)** | **-30.1 (-38.3 , -20.5)** | **6076188 (5345983 , 6886078)** | **2895.5 (2513 , 3385.8)** | **-50.3 (-57.8 , -41.2)** | **240465 (201809 , 285159)** | **111.4 (92.5 , 134.1)** | **-67.9 (-74 , -59.9)** |
| **Brunei Darussalam** | **41093 (35942 , 47126)** | **9894.2 (8722.9 , 11219.7)** | **-19.1 (-29.7 , -7)** | **17896 (14593 , 21595)** | **4410.2 (3624.2 , 5304.5)** | **-35.1 (-47.1 , -20.4)** | **882 (661 , 1152)** | **211 (163.1 , 265.5)** | **-50.9 (-62.8 , -36.2)** |
| **Japan** | **13510360 (12093090 , 15035015)** | **8065.7 (7036 , 9209.3)** | **-21.9 (-34 , -6.3)** | **4403489 (3715661 , 5155960)** | **2815.5 (2317 , 3444.9)** | **-37.6 (-51.1 , -20.4)** | **150118 (116746 , 188295)** | **84.4 (64.2 , 109.9)** | **-50.8 (-64.3 , -30.7)** |
| **Singapore** | **452442 (387982 , 523662)** | **7289.1 (6246.5 , 8450.1)** | **-36 (-46.1 , -24.1)** | **137684 (108621 , 171150)** | **2356.7 (1877.7 , 2945.7)** | **-54.9 (-65 , -42.6)** | **5305 (3900 , 7273)** | **84.1 (62.8 , 114)** | **-68.9 (-77.5 , -58.2)** |
| **Republic of Korea** | **4451578 (3863836 , 5078092)** | **7782.1 (6724.3 , 8939.6)** | **-46.5 (-54.5 , -37.8)** | **1517119 (1263736 , 1821540)** | **3018.7 (2486.4 , 3665.3)** | **-67.9 (-74 , -60.6)** | **84160 (66202 , 108300)** | **166.8 (127.4 , 217.7)** | **-79.5 (-84.5 , -73.1)** |
| **Western Europe** | **17654852 (16502826 , 18948501)** | **3831.5 (3553.7 , 4135.2)** | **-33.2 (-39.1 , -26.5)** | **4330552 (3952990 , 4744678)** | **1018.9 (902.5 , 1150.7)** | **-42.8 (-50.8 , -33.8)** | **135568 (118297 , 156291)** | **29.7 (24.2 , 36.5)** | **-47.8 (-58.1 , -34.6)** |
| **Andorra** | **2874 (2249 , 3629)** | **3335.7 (2598.1 , 4254.8)** | **-29.3 (-47.8 , -3.6)** | **622 (456 , 844)** | **771.2 (532.7 , 1088.8)** | **-37 (-59.1 , -2.3)** | **15 (10 , 22)** | **13.7 (8.9 , 20.3)** | **-47.2 (-67.5 , -11.5)** |
| **Austria** | **339467 (267431 , 429315)** | **3511.3 (2676.4 , 4480.6)** | **-31.5 (-49.2 , -8.8)** | **76712 (58043 , 99773)** | **837.3 (591.9 , 1178)** | **-42.2 (-62.8 , -12.6)** | **1993 (1354 , 2908)** | **15.9 (10.6 , 22.9)** | **-52.5 (-70.3 , -26)** |
| **Belgium** | **361263 (285695 , 448047)** | **3049.2 (2361.7 , 3878.9)** | **-35.6 (-53 , -13.7)** | **80931 (61307 , 107259)** | **716.7 (502.1 , 1045.7)** | **-45.5 (-64.9 , -14)** | **1997 (1337 , 2895)** | **13 (8.6 , 19.4)** | **-56.9 (-73.4 , -30.5)** |
| **Cyprus** | **53014 (41207 , 66138)** | **3858.4 (3005.5 , 4834.4)** | **-48 (-60.7 , -33.2)** | **11911 (8785 , 16024)** | **930.2 (662.1 , 1349.4)** | **-60.7 (-73.6 , -40.3)** | **272 (182 , 400)** | **18.1 (12.2 , 26.2)** | **-72.5 (-82.5 , -58)** |
| **Denmark** | **249772 (192057 , 318743)** | **4184.9 (3173.9 , 5460.2)** | **-35.1 (-52.2 , -13)** | **45727 (33897 , 61708)** | **826.6 (570.6 , 1227.3)** | **-46.2 (-65 , -13.4)** | **824 (547 , 1182)** | **10.8 (7.1 , 16.2)** | **-54.8 (-73.6 , -23.7)** |
| **Finland** | **210699 (166951 , 263326)** | **3733.3 (2809.1 , 4975.3)** | **-39.2 (-56.3 , -15.9)** | **48811 (36663 , 63642)** | **881.6 (620.6 , 1241.6)** | **-51 (-67.2 , -26.7)** | **1357 (905 , 1949)** | **19.5 (12.1 , 29.8)** | **-62 (-77.8 , -36.2)** |
| **France** | **1661242 (1291520 , 2109335)** | **2525.8 (1910.5 , 3360.3)** | **-38 (-55.6 , -14.8)** | **355415 (257400 , 510977)** | **583.6 (391.4 , 938)** | **-47.9 (-67.6 , -12.4)** | **6529 (4294 , 9485)** | **8.6 (5.4 , 13.2)** | **-61.1 (-78.2 , -34.5)** |
| **Germany** | **3388264 (2740910 , 4149803)** | **3665.1 (2931.9 , 4545.6)** | **-35.4 (-51.7 , -14.8)** | **773030 (606997 , 983393)** | **892.1 (652.9 , 1197.1)** | **-46.1 (-63.8 , -21.1)** | **21138 (14459 , 29953)** | **17 (11.8 , 24.2)** | **-57.6 (-73.1 , -34.2)** |
| **Greece** | **531235 (437668 , 643048)** | **4490 (3528.6 , 5646)** | **-24.3 (-41.4 , 0.1)** | **136704 (106147 , 172759)** | **1164.9 (842.2 , 1590.5)** | **-32.7 (-54.4 , -1.6)** | **4997 (3516 , 7029)** | **28.7 (20.5 , 40.8)** | **-42.2 (-61.6 , -12.4)** |
| **Iceland** | **11287 (8747 , 14116)** | **2966 (2266.1 , 3777.1)** | **-26.3 (-46.7 , -1.8)** | **2290 (1708 , 3102)** | **644.7 (458.6 , 929)** | **-36.4 (-59.7 , 0)** | **51 (35 , 74)** | **11.4 (7.5 , 16.9)** | **-47.2 (-67 , -14)** |
| **Ireland** | **169591 (131405 , 210859)** | **3344.5 (2539.8 , 4261)** | **-43.6 (-58.5 , -26.4)** | **36768 (27205 , 50246)** | **736.3 (530.5 , 1036.8)** | **-54.8 (-69.8 , -32.6)** | **804 (536 , 1189)** | **13.7 (9.1 , 20.3)** | **-66.5 (-79.6 , -46.2)** |
| **Israel** | **467205 (373260 , 570631)** | **4875.5 (3862.1 , 5967)** | **-33.7 (-49.2 , -15.8)** | **129163 (91514 , 177553)** | **1355.8 (948.2 , 1878.7)** | **-45.6 (-63.9 , -21)** | **2921 (2020 , 4116)** | **29 (19.6 , 41.3)** | **-56.5 (-72.4 , -34.6)** |
| **Italy** | **2515254 (2036749 , 3037661)** | **3828.3 (2953 , 4798.7)** | **-33.3 (-48.5 , -12.2)** | **609922 (466967 , 798455)** | **961.2 (684.1 , 1384.3)** | **-43.7 (-61.6 , -15.6)** | **19390 (13295 , 26720)** | **20.1 (13.7 , 28.4)** | **-55.4 (-70.8 , -32.5)** |
| **Luxembourg** | **22241 (17745 , 27478)** | **3378.1 (2612.8 , 4274.2)** | **-40.6 (-55.1 , -20)** | **4987 (3673 , 6629)** | **796.2 (568.2 , 1122.6)** | **-51.7 (-67.4 , -24.8)** | **120 (78 , 176)** | **15.1 (10 , 22)** | **-63.3 (-77.7 , -41.6)** |
| **Malta** | **20485 (16594 , 24748)** | **4296.6 (3422 , 5334)** | **-35 (-50.5 , -15.3)** | **4736 (3663 , 6078)** | **1063 (763.6 , 1496)** | **-46.5 (-63.4 , -20.1)** | **127 (90 , 180)** | **21.2 (14.7 , 30.4)** | **-57.4 (-72.7 , -32.7)** |
| **Monaco** | **1343 (1063 , 1653)** | **3188.3 (2409 , 4104)** | **-24.3 (-45.9 , 1.2)** | **296 (223 , 390)** | **742.2 (520.1 , 1094)** | **-31.9 (-55.6 , 3.7)** | **8 (5 , 11)** | **13.4 (9 , 20.2)** | **-40.6 (-63.8 , -2.6)** |
| **Netherlands** | **558600 (436148 , 706952)** | **3160.7 (2402.7 , 4193.8)** | **-30.6 (-51 , -3.3)** | **118149 (87198 , 155662)** | **664.8 (478.7 , 931.6)** | **-40.1 (-60.5 , -11.7)** | **2851 (1825 , 4219)** | **12.4 (7.7 , 18.6)** | **-50.5 (-70.6 , -18.3)** |
| **Norway** | **217981 (177406 , 271974)** | **4017.8 (3179.7 , 5203.4)** | **-33.2 (-49 , -12.4)** | **44407 (33294 , 60805)** | **879.1 (619.1 , 1302.7)** | **-44.3 (-64.1 , -14.5)** | **893 (640 , 1242)** | **14 (9.6 , 20.5)** | **-53.2 (-69.8 , -26.3)** |
| **Portugal** | **443423 (353907 , 545882)** | **4076.3 (3170.2 , 5051.7)** | **-45 (-58.1 , -27.4)** | **107228 (79447 , 147268)** | **1046.3 (728.7 , 1524.8)** | **-58.1 (-71.9 , -33.4)** | **2851 (1912 , 4172)** | **20 (13 , 30.2)** | **-70.4 (-81.5 , -50.6)** |
| **San Marino** | **1195 (947 , 1499)** | **3374.7 (2601.7 , 4315.2)** | **-26.8 (-45.3 , -0.7)** | **269 (206 , 356)** | **794.3 (562.7 , 1119.8)** | **-34.3 (-56.1 , 2.5)** | **7 (5 , 10)** | **14.5 (9.7 , 21.6)** | **-44.6 (-66.7 , -9.2)** |
| **Spain** | **2155499 (1748854 , 2598503)** | **4806.3 (3778 , 5945.3)** | **-37.6 (-53.1 , -19.3)** | **526496 (391098 , 699390)** | **1298.5 (897.2 , 1899.6)** | **-50.7 (-68.4 , -21.8)** | **10692 (7226 , 15576)** | **20.6 (13.4 , 31.2)** | **-65.1 (-79.4 , -42.4)** |
| **Sweden** | **402530 (316247 , 496258)** | **3651.9 (2762.1 , 4647.3)** | **-25 (-44.3 , 0.1)** | **86671 (64107 , 116337)** | **833.3 (573.6 , 1201.1)** | **-32.8 (-56.3 , 2.3)** | **2130 (1472 , 3108)** | **15.9 (10.4 , 23.9)** | **-41.6 (-63.5 , -7.5)** |
| **Switzerland** | **290916 (231867 , 358572)** | **3165.4 (2455.5 , 4030.7)** | **-27.9 (-47.2 , -1.8)** | **63253 (46465 , 85032)** | **733.1 (518.2 , 1056.3)** | **-36.6 (-60.4 , 0.9)** | **1380 (906 , 2064)** | **12.3 (7.9 , 18.5)** | **-45.7 (-68.9 , -5.4)** |
| **United Kingdom** | **3564082 (3135408 , 4073634)** | **4877.8 (4213.5 , 5692.8)** | **-25 (-38.8 , -8.5)** | **1062281 (880451 , 1301165)** | **1652.8 (1320.4 , 2102.4)** | **-32 (-48.5 , -8.6)** | **52103 (40008 , 68248)** | **93 (65.5 , 129.2)** | **-36.3 (-58.3 , -7.1)** |
| **Southern Latin America** | **5643502 (5082629 , 6300672)** | **8466.6 (7635.6 , 9459.5)** | **-24.7 (-33.7 , -15)** | **1947135 (1635745 , 2292795)** | **3090.1 (2570.1 , 3690.5)** | **-42.2 (-53.6 , -29)** | **76813 (63257 , 93049)** | **110.9 (90.6 , 134.2)** | **-55.1 (-64.8 , -44.3)** |
| **Argentina** | **4496009 (3955021 , 5120105)** | **10054.5 (8872.8 , 11467.5)** | **-21.1 (-32.4 , -9.3)** | **1652100 (1348781 , 1998143)** | **3864.3 (3140.6 , 4713.2)** | **-41.3 (-54.3 , -26.2)** | **63234 (49981 , 78980)** | **137.2 (107.8 , 172.1)** | **-53.5 (-65 , -40.2)** |
| **Chile** | **847862 (712766 , 1009791)** | **4186.3 (3495.2 , 4992)** | **-45.2 (-55.6 , -32.5)** | **196617 (157079 , 240138)** | **983.7 (779.9 , 1214.7)** | **-59.6 (-69.4 , -47.5)** | **9813 (7352 , 12716)** | **45.2 (33.7 , 58.6)** | **-67.9 (-76.8 , -54.7)** |
| **Uruguay** | **299348 (257088 , 344357)** | **8787.6 (7510.6 , 10209.1)** | **-23.5 (-35 , -9.3)** | **98320 (77901 , 122065)** | **3071.4 (2360.3 , 3963.7)** | **-38.7 (-53.9 , -18.6)** | **3763 (2920 , 4756)** | **98.4 (74.3 , 126.8)** | **-50.8 (-64.4 , -32.4)** |
| **Eastern Europe** | **19977162 (18187134 , 21835545)** | **8036.8 (7275.9 , 8828.1)** | **-16.2 (-24.8 , -5.8)** | **7109903 (6191846 , 8228960)** | **2865.2 (2488.8 , 3319.8)** | **-31.5 (-42.1 , -19.3)** | **380382 (313920 , 470836)** | **140.4 (114.9 , 171.7)** | **-43.8 (-55.3 , -29.6)** |
| **Belarus** | **899351 (778284 , 1016290)** | **8327.2 (7102.2 , 9562.5)** | **-22.8 (-35.3 , -8.6)** | **290718 (233189 , 351023)** | **2709.9 (2165.9 , 3325)** | **-41.1 (-54.5 , -24.3)** | **13095 (9889 , 17147)** | **109 (83.1 , 139.4)** | **-55.4 (-67.6 , -37.5)** |
| **Estonia** | **120387 (104743 , 139754)** | **7866.5 (6645.7 , 9238.6)** | **-25.8 (-38.6 , -11.9)** | **36788 (29964 , 44367)** | **2391.9 (1925.5 , 2981.3)** | **-46 (-58.7 , -29.8)** | **1645 (1248 , 2133)** | **93.6 (71.1 , 121.7)** | **-59.6 (-70.6 , -44.7)** |
| **Latvia** | **198969 (172599 , 225976)** | **8930.2 (7716.4 , 10261.1)** | **-16.3 (-29.6 , -1)** | **67889 (55798 , 80646)** | **3031.8 (2454 , 3689.6)** | **-33.1 (-48 , -13.3)** | **3284 (2519 , 4239)** | **128.3 (98.3 , 164.1)** | **-46.3 (-60.3 , -25.6)** |
| **Lithuania** | **292238 (257166 , 329375)** | **8991.8 (7736.3 , 10359.2)** | **-12.7 (-25.7 , 1.5)** | **100582 (82166 , 120002)** | **3086.8 (2474.4 , 3764.5)** | **-25.9 (-41.2 , -5.5)** | **4894 (3731 , 6295)** | **130.9 (100.9 , 166.2)** | **-38.7 (-54.3 , -17.8)** |
| **Republic of Moldova** | **474573 (429647 , 522543)** | **11649.7 (10529.7 , 12728.4)** | **-17.4 (-27.2 , -6.7)** | **206928 (177498 , 236838)** | **5208.7 (4452.4 , 6054.3)** | **-36.2 (-47.7 , -22.8)** | **10423 (8286 , 12909)** | **231.1 (185.2 , 283)** | **-47.5 (-60.2 , -31)** |
| **Russian Federation** | **14548086 (12795386 , 16512174)** | **8329.7 (7314.4 , 9441)** | **-15.7 (-27.4 , -1.2)** | **5404028 (4496362 , 6459376)** | **3080.6 (2554.8 , 3692.1)** | **-31.6 (-45.2 , -15.6)** | **291211 (226570 , 378132)** | **154 (119.6 , 197.8)** | **-44.6 (-59.2 , -26)** |
| **Ukraine** | **3443559 (2966720 , 3997977)** | **6686.9 (5724.3 , 7804.4)** | **-16.4 (-30.3 , 1.5)** | **1002970 (826942 , 1206141)** | **2027.2 (1657.6 , 2505.7)** | **-29.2 (-45.4 , -7)** | **55830 (42949 , 70476)** | **98.8 (78 , 125.1)** | **-39 (-55 , -17.8)** |
| **Central Europe** | **10058285 (9425435 , 10721339)** | **9130.8 (8540.5 , 9775.9)** | **-20.8 (-26.4 , -14.8)** | **3386964 (3112209 , 3699949)** | **3372 (3076.1 , 3728.9)** | **-38 (-44.2 , -30.7)** | **150549 (134440 , 171824)** | **134.3 (120.3 , 151.7)** | **-53.7 (-59.7 , -46.7)** |
| **Albania** | **319975 (297395 , 342174)** | **11760 (10974.5 , 12558.9)** | **-24.3 (-32.9 , -14.7)** | **126721 (111234 , 143049)** | **5070.8 (4379.1 , 5903.8)** | **-38.9 (-49.9 , -25.8)** | **5229 (4323 , 6359)** | **189.6 (157.4 , 226.5)** | **-57.8 (-68 , -44.1)** |
| **Bosnia and Herzegovina** | **337879 (288257 , 390426)** | **10366.1 (8870.4 , 12012)** | **-17.9 (-30.6 , -3.2)** | **121307 (98834 , 144747)** | **4078.7 (3269.8 , 4986.2)** | **-35.6 (-49.2 , -18.2)** | **5824 (4399 , 7544)** | **174 (133.5 , 223.2)** | **-51.6 (-64.4 , -34.3)** |
| **Bulgaria** | **692758 (595818 , 791988)** | **10460.1 (8974.9 , 12005.9)** | **-8.3 (-23.2 , 8.7)** | **247765 (200015 , 297802)** | **4101.1 (3309.1 , 5063)** | **-18.5 (-37.5 , 3.9)** | **11932 (8907 , 15756)** | **173.9 (133.8 , 223.8)** | **-30.7 (-50 , -4.2)** |
| **Croatia** | **305699 (254385 , 364825)** | **7343.1 (6057.2 , 8796.9)** | **-18.3 (-32.9 , 2.1)** | **84734 (65752 , 106038)** | **2153.1 (1670.7 , 2724.8)** | **-31.9 (-49.5 , -6.9)** | **3328 (2320 , 4561)** | **72.8 (52.2 , 99.9)** | **-43.5 (-61.8 , -17)** |
| **Czechia** | **798964 (669729 , 930721)** | **7838.8 (6514.1 , 9220.7)** | **-22.4 (-36.6 , -4.8)** | **235615 (188118 , 288261)** | **2491.2 (1975.6 , 3112.2)** | **-38.5 (-53.4 , -18)** | **9232 (6868 , 12037)** | **87.2 (64.5 , 112.2)** | **-52.9 (-66.9 , -33.6)** |
| **Hungary** | **678537 (561198 , 807264)** | **7654.2 (6361 , 9006.1)** | **-19.2 (-34.7 , -2.1)** | **220209 (170492 , 275750)** | **2770.3 (2149.1 , 3485.1)** | **-36 (-52.2 , -12.3)** | **9218 (6611 , 12608)** | **102.5 (74.9 , 138.1)** | **-50.1 (-65.1 , -27.9)** |
| **Montenegro** | **56458 (48662 , 65665)** | **9184.4 (7834.5 , 10726)** | **-9.7 (-24.7 , 8.4)** | **18549 (15015 , 22735)** | **3241.6 (2620.5 , 4046.5)** | **-19.6 (-38 , 5.2)** | **787 (592 , 1034)** | **125.5 (93.8 , 161.7)** | **-31.1 (-51.2 , -4.4)** |
| **North Macedonia** | **201081 (172662 , 231151)** | **9250.4 (8082 , 10463.4)** | **-17.3 (-29.8 , -1.9)** | **68491 (57346 , 80631)** | **3747.4 (3110.9 , 4496.1)** | **-34 (-46.5 , -15.9)** | **3660 (2890 , 4585)** | **197.7 (153.3 , 256.6)** | **-49.6 (-63 , -30.1)** |
| **Poland** | **3465219 (2963656 , 4044364)** | **9228.2 (7884.1 , 10809.7)** | **-23.5 (-35.7 , -9.8)** | **1171119 (948884 , 1419627)** | **3419.7 (2722.8 , 4250.4)** | **-42.2 (-54.3 , -26)** | **53530 (40786 , 70681)** | **141.1 (107.9 , 183.6)** | **-58.6 (-70.2 , -43.6)** |
| **Romania** | **1783469 (1532344 , 2046733)** | **9827.3 (8462 , 11172)** | **-19.4 (-32.4 , -5.5)** | **631183 (510938 , 753923)** | **3851.3 (3079.2 , 4700.5)** | **-36 (-50.4 , -17.8)** | **28018 (21224 , 36160)** | **151.2 (115.2 , 194.3)** | **-52.4 (-65 , -34.6)** |
| **Serbia** | **817524 (690254 , 949298)** | **9727 (8273.8 , 11302)** | **-20.3 (-33.5 , -5.4)** | **277730 (224545 , 340813)** | **3562.2 (2840.5 , 4417.9)** | **-37.8 (-52.6 , -21.6)** | **12371 (9223 , 16502)** | **143.6 (108.1 , 188.3)** | **-53.5 (-66.5 , -35.9)** |
| **Slovakia** | **458514 (388572 , 539809)** | **8525.5 (7187.2 , 10085.2)** | **-19.7 (-33.7 , -3.2)** | **144122 (114275 , 176708)** | **2915.4 (2278.8 , 3574.7)** | **-34.6 (-50.2 , -14)** | **5954 (4361 , 7837)** | **108.2 (80.5 , 139.1)** | **-49.1 (-64.5 , -29.7)** |
| **Slovenia** | **142208 (119219 , 169115)** | **7085.2 (5954.2 , 8443.4)** | **-24.4 (-38.3 , -6.7)** | **39421 (30956 , 48696)** | **2135.3 (1663.8 , 2698.9)** | **-39.6 (-54.7 , -17.8)** | **1466 (1053 , 1993)** | **69.7 (50.8 , 93.9)** | **-53.8 (-67.3 , -31.9)** |
| **Central Asia** | **13991482 (13470570 , 14520395)** | **15035.8 (14503.5 , 15579.5)** | **-5 (-8.9 , -1.2)** | **9876268 (9220191 , 10558527)** | **10504.5 (9835.2 , 11236.2)** | **-21.4 (-27.3 , -15.3)** | **570652 (514376 , 632214)** | **611.2 (552.7 , 674.9)** | **-37.3 (-44 , -29.4)** |
| **Armenia** | **338875 (305419 , 373127)** | **10671.6 (9618.4 , 11766.2)** | **-8.9 (-18.8 , 2.6)** | **165288 (142786 , 191043)** | **5688.7 (4825.5 , 6677.5)** | **-20.5 (-33.8 , -4.4)** | **11261 (9312 , 13616)** | **371.8 (303.9 , 457.1)** | **-36.7 (-51 , -20)** |
| **Azerbaijan** | **1452049 (1333808 , 1573208)** | **13948.9 (12921.1 , 15078.8)** | **-7.7 (-15.6 , 1.3)** | **828126 (728976 , 937597)** | **8019.8 (7105.3 , 9073.2)** | **-28.9 (-39.1 , -17.2)** | **47055 (37661 , 58063)** | **441.4 (362 , 531.3)** | **-43.4 (-55.2 , -29.5)** |
| **Georgia** | **488396 (449678 , 526317)** | **12391.8 (11330.9 , 13481.3)** | **-5.7 (-15 , 4.7)** | **290068 (256329 , 328730)** | **7655.2 (6667.9 , 8768.5)** | **-14.4 (-28 , 1.1)** | **21629 (17949 , 25934)** | **527.1 (437.2 , 627.9)** | **-15.8 (-33.8 , 8)** |
| **Kazakhstan** | **2579817 (2387622 , 2781528)** | **13885 (12856 , 14981.1)** | **-11.8 (-19.7 , -4.1)** | **1732358 (1511920 , 1972264)** | **9287.4 (8130.1 , 10596.1)** | **-29.7 (-39.2 , -19)** | **104872 (84842 , 129206)** | **557.6 (453.8 , 681.9)** | **-43.8 (-55.3 , -29.2)** |
| **Kyrgyzstan** | **927522 (849482 , 1005775)** | **14365 (13230.6 , 15510.2)** | **-4.3 (-12.9 , 5)** | **659056 (574507 , 751148)** | **9947.3 (8769.5 , 11247.5)** | **-22.1 (-33 , -10.3)** | **39139 (32867 , 46667)** | **616.5 (519.9 , 736.2)** | **-42.7 (-53.8 , -28.8)** |
| **Mongolia** | **399696 (369970 , 429294)** | **11954.1 (11088.9 , 12808)** | **-14.1 (-21.4 , -6.8)** | **268168 (232286 , 305462)** | **8009 (7003.1 , 9047.3)** | **-38.9 (-47.4 , -29)** | **27027 (22426 , 31881)** | **810.8 (684.5 , 949.6)** | **-54.2 (-62.3 , -44.3)** |
| **Tajikistan** | **1269939 (1162821 , 1372516)** | **13996.4 (13009.3 , 15028.6)** | **-3.9 (-12.3 , 5.3)** | **851358 (734310 , 968922)** | **9105.6 (7951.5 , 10208.3)** | **-18 (-29 , -4.7)** | **57981 (48188 , 68324)** | **660.9 (559.3 , 768.3)** | **-29.2 (-42.9 , -12.4)** |
| **Turkmenistan** | **649817 (594980 , 709368)** | **12932.4 (11909.9 , 14046.4)** | **-10.2 (-18.7 , -0.7)** | **386961 (332902 , 449916)** | **7602.4 (6582.8 , 8794)** | **-30.1 (-40.7 , -16.9)** | **25291 (20735 , 30911)** | **502.7 (415 , 607.4)** | **-43.4 (-54.8 , -28.3)** |
| **Uzbekistan** | **5885371 (5416261 , 6327048)** | **17654.6 (16365.6 , 18839.7)** | **-4.7 (-12 , 3)** | **4694885 (4114251 , 5289588)** | **13681.6 (12006.2 , 15354.9)** | **-21.6 (-32.5 , -9.6)** | **236396 (188662 , 291296)** | **706.6 (567.7 , 857.5)** | **-36.4 (-50.4 , -18.3)** |
| **Central Latin America** | **16843734 (16239642 , 17513040)** | **6897.6 (6654.8 , 7159.3)** | **-25.9 (-29.1 , -22.3)** | **7701322 (7357622 , 8116801)** | **3211.1 (3069.5 , 3380.5)** | **-40.3 (-43.8 , -36.3)** | **428149 (404931 , 451295)** | **178.2 (168.6 , 187.5)** | **-53.1 (-56.3 , -49.8)** |
| **Colombia** | **3019381 (2596648 , 3496558)** | **6257.5 (5346.7 , 7230.3)** | **-43.4 (-52.1 , -33.6)** | **1043198 (840566 , 1266952)** | **2211.7 (1751.9 , 2726.4)** | **-61.1 (-69.5 , -50.5)** | **53019 (41748 , 66666)** | **105.6 (82.4 , 133.3)** | **-68.5 (-76.4 , -58.5)** |
| **Costa Rica** | **341089 (292579 , 392602)** | **7230.7 (6177.8 , 8309.2)** | **-27.7 (-39 , -15.1)** | **127294 (100262 , 157962)** | **2797.5 (2170.4 , 3499.4)** | **-39.3 (-53.4 , -20.2)** | **5652 (4386 , 7265)** | **117.7 (90.9 , 150.6)** | **-48.2 (-61 , -31.4)** |
| **El Salvador** | **494928 (433733 , 561963)** | **8095.5 (7089.5 , 9130.1)** | **-26.2 (-37.4 , -13.5)** | **225025 (180467 , 271988)** | **3690.4 (2960.6 , 4465.9)** | **-38.2 (-52.8 , -20.8)** | **10271 (8417 , 12644)** | **166.1 (135.7 , 204.9)** | **-59 (-68.6 , -46.7)** |
| **Guatemala** | **1837547 (1646325 , 2049387)** | **10660.1 (9628.3 , 11760.4)** | **-20.9 (-29.6 , -11.2)** | **1055429 (891022 , 1250041)** | **5931.6 (5065.3 , 6931.2)** | **-37.1 (-47.9 , -25.4)** | **51671 (42197 , 63463)** | **325.3 (271.1 , 391.4)** | **-57.8 (-65.9 , -47.7)** |
| **Honduras** | **1014686 (898905 , 1131643)** | **10784.2 (9658.5 , 11910.8)** | **-22.3 (-31.1 , -11.8)** | **511354 (425850 , 618270)** | **5323.4 (4544.6 , 6284.8)** | **-36.8 (-47.9 , -22.5)** | **23017 (18928 , 27955)** | **282.3 (236.8 , 335.7)** | **-48.4 (-58.1 , -35.6)** |
| **Mexico** | **7141962 (6967387 , 7335007)** | **5922.5 (5778.6 , 6073.3)** | **-19.7 (-22.2 , -17)** | **3584836 (3457093 , 3731575)** | **3006.1 (2899.8 , 3128.2)** | **-35.1 (-38.4 , -31.7)** | **225774 (214598 , 237983)** | **189.4 (180.4 , 199.3)** | **-47.7 (-50.9 , -44)** |
| **Nicaragua** | **424982 (361455 , 494434)** | **6980.9 (6000.9 , 8026.7)** | **-39.8 (-49.1 , -28.9)** | **143459 (115317 , 177170)** | **2411.5 (1971.1 , 2899.5)** | **-58.8 (-67.4 , -48.2)** | **6853 (5423 , 8618)** | **132.1 (105.3 , 162.9)** | **-66.8 (-74.4 , -57.6)** |
| **Panama** | **431863 (386052 , 483981)** | **10386.7 (9272 , 11667.4)** | **-18.9 (-28.6 , -6.9)** | **207596 (172737 , 246792)** | **4986.8 (4148.4 , 5922.6)** | **-34.6 (-47.2 , -19.7)** | **9280 (7196 , 11726)** | **221.6 (171.8 , 279.1)** | **-51.4 (-64 , -35.1)** |
| **Venezuela (Bolivarian Republic of)** | **2137296 (1843331 , 2457988)** | **7647.9 (6608.6 , 8787.6)** | **-27.7 (-37.9 , -15.1)** | **803131 (660768 , 972525)** | **2949.1 (2412.5 , 3588.5)** | **-42.7 (-54.5 , -27.1)** | **42613 (33711 , 53043)** | **153.6 (122.3 , 189.2)** | **-55.2 (-65.7 , -41.3)** |
| **Andean Latin America** | **6483512 (6081846 , 6904680)** | **10204.5 (9586.3 , 10850.6)** | **-23.9 (-29.6 , -17.6)** | **3333890 (3018138 , 3709558)** | **5266.1 (4762.4 , 5857.2)** | **-52.7 (-57.9 , -46.3)** | **171559 (154795 , 191663)** | **275.9 (249.6 , 307.5)** | **-71.9 (-75.8 , -67.2)** |
| **Bolivia (Plurinational State of)** | **1927702 (1763606 , 2102525)** | **15647.8 (14328.2 , 17024.5)** | **-10.5 (-19.1 , -1.6)** | **1150862 (1000659 , 1323549)** | **9057.3 (7907.2 , 10368.9)** | **-35.1 (-44.3 , -23.7)** | **73713 (61526 , 89542)** | **608.9 (513.6 , 731.9)** | **-59.2 (-67.5 , -49.3)** |
| **Ecuador** | **1012682 (901072 , 1126218)** | **5930.9 (5325.1 , 6577.2)** | **-44.1 (-51.1 , -35.6)** | **444858 (378426 , 516868)** | **2606.3 (2225.3 , 3009.3)** | **-65.5 (-72.3 , -57.6)** | **25324 (20918 , 30156)** | **154.4 (128.3 , 183.3)** | **-69 (-76.1 , -59.8)** |
| **Peru** | **3543128 (3195301 , 3905625)** | **10548.4 (9506 , 11598.6)** | **-22.4 (-31.2 , -11.7)** | **1738170 (1466864 , 2052056)** | **5227.3 (4393 , 6203.1)** | **-56.5 (-64.5 , -46.6)** | **72523 (62013 , 86071)** | **216.9 (184.4 , 258.1)** | **-79.5 (-83.6 , -73.9)** |
| **Caribbean** | **7204515 (6869783 , 7525231)** | **15209.6 (14509.5 , 15868.9)** | **-6.5 (-11.7 , -1)** | **4449223 (4175496 , 4747910)** | **9775.3 (9142.7 , 10458.7)** | **-6.6 (-14.4 , 1)** | **232756 (206274 , 262725)** | **499.9 (441.9 , 566.8)** | **-23.3 (-34.4 , -10.5)** |
| **Antigua and Barbuda** | **13286 (11758 , 14812)** | **14999.1 (13397.9 , 16643.7)** | **-10.7 (-21.8 , 1.1)** | **6047 (4997 , 7057)** | **7316.9 (6060.5 , 8684)** | **-25.3 (-39.4 , -8)** | **229 (174 , 293)** | **253.7 (192.6 , 323.2)** | **-38.8 (-55 , -15.9)** |
| **Barbados** | **36075 (31400 , 40893)** | **11570.1 (10096.2 , 13102.3)** | **-14.8 (-26.2 , -1.8)** | **13036 (10623 , 15532)** | **5107.4 (4073.3 , 6229.4)** | **-25.4 (-40.9 , -6.8)** | **498 (350 , 714)** | **211.4 (136 , 324.7)** | **-52.2 (-71.5 , -19.7)** |
| **Belize** | **67904 (61142 , 74803)** | **17181.5 (15544.7 , 18785.8)** | **-4.8 (-14.8 , 6.3)** | **40051 (33469 , 46720)** | **9853 (8287.3 , 11420.5)** | **-14.9 (-28.9 , 2.1)** | **1723 (1320 , 2195)** | **429.7 (333.2 , 542.5)** | **-28.9 (-46.4 , -5.9)** |
| **Bermuda** | **6566 (5695 , 7553)** | **9722.9 (8406.4 , 11137.8)** | **-27.6 (-38.3 , -14.6)** | **1971 (1581 , 2414)** | **3357 (2618.7 , 4234.4)** | **-45.8 (-59.2 , -28.3)** | **59 (43 , 80)** | **86.2 (63 , 116.5)** | **-61.5 (-73.5 , -45.1)** |
| **Bahamas** | **56880 (50739 , 62965)** | **15167.6 (13597.7 , 16746.4)** | **-7.3 (-18.5 , 4.6)** | **28115 (23759 , 32793)** | **7788.7 (6483.8 , 9118.8)** | **-16.2 (-31.3 , 2.3)** | **1169 (882 , 1513)** | **302.2 (233.7 , 385)** | **-27.7 (-46.4 , -1.7)** |
| **Cuba** | **1476625 (1292443 , 1662245)** | **12448.4 (10943.6 , 14096.2)** | **-15.4 (-27 , -2.8)** | **542663 (442816 , 645213)** | **5105.3 (4127.1 , 6220.5)** | **-28.3 (-45.1 , -9.6)** | **17979 (13329 , 23511)** | **150.5 (112.3 , 195.9)** | **-42.8 (-59 , -21.3)** |
| **Dominica** | **10581 (9494 , 11699)** | **14739.6 (13281.6 , 16264.3)** | **-5.3 (-16.4 , 7.7)** | **4914 (4279 , 5596)** | **7138.1 (6192.9 , 8213.1)** | **-13.7 (-28.3 , 4.7)** | **242 (190 , 302)** | **336.3 (266.3 , 420.1)** | **-16.9 (-37.3 , 9.5)** |
| **Dominican Republic** | **1512398 (1357225 , 1674261)** | **14011.3 (12659.2 , 15442.3)** | **-16.8 (-26 , -6.5)** | **712587 (605325 , 814680)** | **6577.9 (5608.9 , 7517.8)** | **-37.3 (-47.5 , -25.5)** | **32639 (25649 , 40845)** | **301.4 (237.9 , 376.9)** | **-52.4 (-64.5 , -38.2)** |
| **Grenada** | **14668 (13112 , 16435)** | **14373.5 (12921.3 , 16025.2)** | **-9 (-18 , 1.4)** | **7526 (6320 , 8720)** | **7989.9 (6625.1 , 9417)** | **-25.9 (-38.3 , -10.9)** | **303 (233 , 379)** | **309.1 (237.7 , 389.7)** | **-42.6 (-56.4 , -24.6)** |
| **Guyana** | **140683 (131068 , 150238)** | **18499.9 (17304.1 , 19668)** | **-13.7 (-20.6 , -6.6)** | **96632 (85756 , 108857)** | **12842.8 (11372.9 , 14459.4)** | **-27.8 (-37.5 , -17)** | **5689 (4716 , 6934)** | **754.4 (625.3 , 913.6)** | **-46.5 (-57.3 , -33.6)** |
| **Haiti** | **2398645 (2246240 , 2563032)** | **19833.4 (18678.7 , 21076.7)** | **1 (-7.2 , 10.4)** | **2310630 (2093850 , 2557911)** | **17929.1 (16371.9 , 19691.9)** | **-4.3 (-14.7 , 7.5)** | **143382 (120607 , 171923)** | **1175.1 (995.8 , 1381.5)** | **-34.9 (-48.6 , -17.8)** |
| **Jamaica** | **434663 (386791 , 482339)** | **15553.2 (13912 , 17172)** | **-6.4 (-18 , 4.7)** | **207216 (173333 , 242482)** | **7757.7 (6420 , 9131.5)** | **-16.5 (-32.9 , 2)** | **8238 (6282 , 10393)** | **292.7 (224.9 , 373)** | **-25.5 (-46 , 2)** |
| **Puerto Rico** | **402931 (349796 , 460066)** | **10868.9 (9305.8 , 12376.6)** | **-20.4 (-33.1 , -6.6)** | **131067 (104763 , 158109)** | **4007.4 (3115.7 , 5015.8)** | **-36.3 (-51.8 , -15.8)** | **4151 (3041 , 5501)** | **112.3 (80.6 , 150.2)** | **-50.6 (-65.2 , -30)** |
| **Saint Kitts and Nevis** | **8317 (7353 , 9357)** | **13854.4 (12278 , 15563.3)** | **-13.2 (-24.9 , -0.4)** | **3544 (2948 , 4279)** | **6249.4 (5166.6 , 7570.2)** | **-28.6 (-43.6 , -10)** | **130 (99 , 170)** | **210.1 (162.2 , 272.2)** | **-43.9 (-58.3 , -21.6)** |
| **Saint Lucia** | **28080 (25325 , 31331)** | **15792.7 (14256 , 17560.4)** | **-11.1 (-21 , 0.9)** | **13488 (11498 , 15674)** | **8161.7 (6855.5 , 9656.8)** | **-27.1 (-40.7 , -11.8)** | **567 (444 , 717)** | **314.1 (247.1 , 395.2)** | **-44 (-58.6 , -26)** |
| **Saint Vincent and the Grenadines** | **19208 (17440 , 21010)** | **16709.6 (15214.7 , 18299.3)** | **-4.1 (-14.5 , 8)** | **10120 (8742 , 11645)** | **9266.9 (7956.9 , 10790.8)** | **-13.1 (-28.1 , 4.6)** | **468 (368 , 590)** | **405.4 (320.4 , 510.7)** | **-22.5 (-41.2 , 2.3)** |
| **Suriname** | **97777 (89443 , 106658)** | **16951.9 (15509.3 , 18450.2)** | **-6 (-15.9 , 4.9)** | **56921 (49306 , 65713)** | **10032.2 (8663.8 , 11643.3)** | **-16.9 (-30.2 , -1.3)** | **2718 (2135 , 3382)** | **464.3 (365.9 , 576.1)** | **-31.4 (-48.4 , -10.6)** |
| **Trinidad and Tobago** | **220358 (197666 , 245363)** | **15537.9 (13890.5 , 17300.9)** | **-9.8 (-19.9 , 3.1)** | **105732 (90035 , 123954)** | **8010.5 (6643.2 , 9533.7)** | **-22.7 (-37.2 , -4.9)** | **4450 (3449 , 5730)** | **312.5 (242.3 , 396.7)** | **-37.6 (-54.7 , -16)** |
| **United States Virgin Islands** | **14818 (13222 , 16490)** | **13602.7 (12071.9 , 15229.9)** | **-11.5 (-22.7 , 1.3)** | **6247 (5210 , 7458)** | **6326 (5141.9 , 7663.6)** | **-23.2 (-38 , -2.5)** | **239 (183 , 307)** | **223.5 (169.4 , 289.5)** | **-36.2 (-54.1 , -12.6)** |
| **Tropical Latin America** | **25821324 (23679992 , 28212757)** | **11308.6 (10419.2 , 12290.3)** | **-19.8 (-28.4 , -10.5)** | **16077552 (14167408 , 18114829)** | **7291.4 (6387.5 , 8218.9)** | **-32.8 (-42.4 , -21.1)** | **1034297 (825618 , 1268584)** | **463.1 (368.5 , 565.1)** | **-50.9 (-62.6 , -36.3)** |
| **Brazil** | **25072131 (22934041 , 27470324)** | **11314.8 (10385.3 , 12331.8)** | **-19.8 (-28.6 , -10.1)** | **15664735 (13759238 , 17708970)** | **7331 (6402.4 , 8297.9)** | **-32.7 (-42.8 , -20.8)** | **1011430 (804664 , 1246955)** | **467.1 (370 , 572.1)** | **-50.9 (-62.9 , -35.8)** |
| **Paraguay** | **749193 (668595 , 843574)** | **11010.3 (9896.3 , 12324)** | **-20.5 (-29.4 , -9.8)** | **412817 (344807 , 488770)** | **6028.3 (5058.3 , 7134.6)** | **-32.6 (-43.9 , -19)** | **22867 (18096 , 29094)** | **339.8 (271.3 , 425.8)** | **-46.2 (-59.4 , -29.4)** |
| **East Asia** | **98064025 (93453919 , 102984399)** | **5961.1 (5670.4 , 6263.6)** | **-51 (-53.5 , -48.3)** | **34517658 (31068896 , 38265124)** | **2270.3 (2040.7 , 2525)** | **-70.4 (-73.8 , -66.6)** | **2025674 (1683710 , 2439816)** | **127 (106.3 , 151.7)** | **-81.4 (-85 , -76.8)** |
| **China** | **93004565 (88294259 , 97892897)** | **5827.5 (5529.5 , 6144.1)** | **-52.1 (-54.7 , -49.3)** | **32095733 (28470559 , 35812610)** | **2170.8 (1932.7 , 2422.5)** | **-71.8 (-75.3 , -67.9)** | **1881991 (1545445 , 2290635)** | **121.9 (100.4 , 147.8)** | **-82.4 (-86 , -77.8)** |
| **Democratic People's Republic of Korea** | **3308165 (3029806 , 3634639)** | **12304.4 (11300.7 , 13469.4)** | **-10 (-19.1 , 0.9)** | **1849316 (1628533 , 2078120)** | **7208.1 (6292.8 , 8265.6)** | **-21 (-34.2 , -5.3)** | **117102 (93144 , 144606)** | **417.8 (335.2 , 512)** | **-21.2 (-40.5 , 2.4)** |
| **Taiwan (Province of China)** | **1751295 (1496446 , 2021007)** | **6496.6 (5532.8 , 7464.4)** | **-33.3 (-43.5 , -20.6)** | **572609 (457840 , 699216)** | **2386.8 (1871.1 , 2993.4)** | **-49.8 (-61.7 , -34.9)** | **26581 (20337 , 35015)** | **101.4 (75.4 , 133.5)** | **-63 (-73.3 , -48.8)** |
| **Southeast Asia** | **85960385 (82670123 , 89136131)** | **12986 (12524.1 , 13453.3)** | **-22 (-25.6 , -18.4)** | **44891576 (42343458 , 47489602)** | **6984.4 (6598.7 , 7390.6)** | **-43.8 (-47.8 , -39.6)** | **2218406 (2043741 , 2403058)** | **342.6 (316.5 , 370.6)** | **-60.1 (-64.3 , -55.6)** |
| **Cambodia** | **2936104 (2738957 , 3172865)** | **18025.5 (16861.1 , 19384.7)** | **-6.7 (-14.6 , 2.2)** | **2144030 (1868807 , 2416507)** | **13032.8 (11457.9 , 14598.3)** | **-33.3 (-42.4 , -22.6)** | **79236 (65603 , 94918)** | **511.5 (426.6 , 614.4)** | **-65.7 (-72.7 , -56.7)** |
| **Indonesia** | **34989872 (32434830 , 37485646)** | **13748.3 (12809.8 , 14655.6)** | **-22.3 (-29 , -15.3)** | **18586400 (16559737 , 20626436)** | **7529.1 (6732.7 , 8307.8)** | **-45.7 (-52.8 , -37.6)** | **1030543 (878547 , 1185522)** | **428.7 (369.3 , 487)** | **-56.4 (-64.1 , -47.6)** |
| **Lao People's Democratic Republic** | **1140040 (1049013 , 1248208)** | **16605.4 (15393.5 , 17952.4)** | **-8.2 (-15.5 , 0.4)** | **737014 (647737 , 841644)** | **10679.1 (9453.1 , 12064.2)** | **-33.4 (-42 , -22.9)** | **36497 (30455 , 43156)** | **574.1 (485.3 , 672.6)** | **-58.3 (-66 , -48.2)** |
| **Malaysia** | **3837780 (3443251 , 4226147)** | **12475.3 (11264.8 , 13727)** | **-6.4 (-16 , 3.6)** | **1979783 (1679934 , 2299798)** | **6574 (5596.3 , 7668.9)** | **-39.5 (-49 , -28.9)** | **134514 (109541 , 163881)** | **434.6 (355.7 , 529.1)** | **-61.7 (-69.3 , -52.6)** |
| **Maldives** | **67464 (59963 , 75530)** | **14398.5 (12884.5 , 15983.3)** | **-25.3 (-34.2 , -16)** | **34681 (28744 , 40858)** | **7550.7 (6201.7 , 8975)** | **-62.9 (-69.8 , -55.7)** | **979 (755 , 1244)** | **211.4 (167.8 , 262.4)** | **-85.8 (-89.5 , -81.3)** |
| **Mauritius** | **177026 (157380 , 195985)** | **13525.3 (12097 , 14885.7)** | **-12.7 (-23 , -1.8)** | **79913 (67437 , 93179)** | **7155.4 (5940.7 , 8521.8)** | **-33.5 (-45.6 , -19.9)** | **3360 (2701 , 4184)** | **285.1 (225.1 , 365.4)** | **-50.7 (-63.5 , -35.5)** |
| **Myanmar** | **9729070 (9075975 , 10410952)** | **18107.4 (16978.1 , 19335.5)** | **-3 (-10.6 , 4.9)** | **7598288 (6879467 , 8429316)** | **14312.2 (12951.6 , 15867)** | **-20.3 (-30 , -9.5)** | **345635 (293852 , 405272)** | **656.4 (557.5 , 772)** | **-53.2 (-62.7 , -41.2)** |
| **Philippines** | **12072637 (11015300 , 13219484)** | **11222.8 (10311.8 , 12184.1)** | **-27.2 (-34.5 , -19.3)** | **6102900 (5227593 , 7077338)** | **5625.8 (4874.7 , 6431.5)** | **-44.2 (-53.3 , -33.7)** | **301386 (241783 , 369559)** | **294.7 (239.9 , 358.7)** | **-55.9 (-65.2 , -43.5)** |
| **Sri Lanka** | **2895135 (2590854 , 3199853)** | **12854.4 (11530 , 14200.8)** | **-25.8 (-34.4 , -16.8)** | **1229968 (1011709 , 1445690)** | **5588.2 (4607.1 , 6547.9)** | **-56.9 (-65.1 , -47.3)** | **41905 (32402 , 53194)** | **185.2 (144.4 , 233.4)** | **-76.2 (-82.1 , -68.3)** |
| **Seychelles** | **13048 (11691 , 14671)** | **12673.4 (11308.4 , 14183.4)** | **-19 (-28.8 , -8)** | **5492 (4656 , 6509)** | **5665.3 (4674.5 , 6816.7)** | **-41.3 (-52.8 , -27.2)** | **210 (165 , 259)** | **205.5 (161.9 , 252.4)** | **-59.6 (-69.6 , -47.3)** |
| **Thailand** | **8541374 (7514572 , 9720323)** | **11142.3 (9726.3 , 12607.3)** | **-28.4 (-38.1 , -18)** | **2555505 (2101066 , 3046965)** | **3669.7 (2968.3 , 4513.2)** | **-51.5 (-61.5 , -39.5)** | **80174 (61008 , 102150)** | **102.4 (76.6 , 128.3)** | **-68.6 (-77.2 , -58.4)** |
| **Timor-Leste** | **206536 (189584 , 224241)** | **15583.8 (14428.5 , 16796.8)** | **-12.8 (-21.1 , -3)** | **132729 (111608 , 154086)** | **9333 (8088.9 , 10615)** | **-34.4 (-44.4 , -22.7)** | **4395 (3653 , 5239)** | **365.5 (311.2 , 428.9)** | **-60.8 (-68.9 , -50.5)** |
| **Viet Nam** | **9241682 (8218973 , 10305968)** | **9647.2 (8659.7 , 10720.6)** | **-35 (-42.8 , -25.7)** | **3646061 (3047737 , 4334771)** | **4067.6 (3392 , 4836.2)** | **-60.3 (-67.3 , -51.4)** | **156666 (124979 , 199302)** | **174.7 (139 , 222.4)** | **-73.4 (-80.1 , -64.5)** |
| **Oceania** | **2133506 (2025829 , 2246745)** | **16822.8 (16043 , 17660.7)** | **-1.6 (-8 , 5.1)** | **1836510 (1676590 , 2002971)** | **13344.5 (12312 , 14428.7)** | **-10.8 (-18.8 , -2.4)** | **147560 (124195 , 173080)** | **1123.8 (962.5 , 1301.1)** | **-24.6 (-37.7 , -9.3)** |
| **American Samoa** | **7774 (7013 , 8537)** | **14841.4 (13476.1 , 16219.8)** | **-3.3 (-13.8 , 8.2)** | **4014 (3446 , 4636)** | **7658.7 (6562.7 , 8840)** | **-13.8 (-27.7 , 1.7)** | **211 (167 , 263)** | **406.8 (323.3 , 501.5)** | **-24.6 (-42.1 , -1.5)** |
| **Cook Islands** | **2567 (2292 , 2882)** | **13640.3 (12190.5 , 15320.1)** | **-9.6 (-20.8 , 4.1)** | **1073 (889 , 1276)** | **6022.4 (4947.2 , 7298.6)** | **-25.7 (-40.3 , -7.4)** | **41 (31 , 52)** | **219.8 (166.5 , 280.4)** | **-40.9 (-56.5 , -18.5)** |
| **Micronesia (Federated States of)** | **16183 (14778 , 17665)** | **16972.4 (15588 , 18444.9)** | **-4.9 (-13.4 , 4.7)** | **10581 (9211 , 12063)** | **10964.5 (9624.2 , 12394.9)** | **-24.5 (-34.7 , -13.6)** | **617 (499 , 763)** | **663.7 (543.9 , 810.7)** | **-42.9 (-54.4 , -28.1)** |
| **Fiji** | **185294 (171999 , 199334)** | **20794.3 (19318.8 , 22312.6)** | **12.5 (3.1 , 22.3)** | **103809 (92105 , 116777)** | **11655.9 (10404.7 , 13022.1)** | **2.1 (-11.5 , 18.3)** | **5078 (4194 , 6141)** | **591.8 (492.9 , 709.1)** | **-9.1 (-28 , 14.7)** |
| **Guam** | **25170 (22456 , 27942)** | **14526 (12971 , 16077.6)** | **-0.2 (-13.1 , 13.8)** | **11959 (10185 , 14054)** | **7030.8 (5931.6 , 8278.9)** | **-8.4 (-25.2 , 11.5)** | **511 (403 , 635)** | **295.7 (233 , 369.8)** | **-14.1 (-35.8 , 13.5)** |
| **Kiribati** | **20301 (18782 , 21801)** | **18231.9 (16994.3 , 19473.3)** | **0.4 (-7.6 , 9.9)** | **16737 (14812 , 18721)** | **14333.8 (12865.1 , 15803.2)** | **-9.3 (-19.6 , 3.3)** | **1258 (1018 , 1561)** | **1151.9 (951.7 , 1389.7)** | **-22.8 (-37.7 , -2.2)** |
| **Marshall Islands** | **9150 (8382 , 9950)** | **17367.1 (16001.8 , 18835.6)** | **-0.5 (-10 , 9.4)** | **6321 (5495 , 7200)** | **11354 (10008.8 , 12774.1)** | **-16.3 (-27.2 , -4.2)** | **390 (312 , 482)** | **729.3 (593.1 , 887.8)** | **-29 (-43.5 , -10.7)** |
| **Nauru** | **1565 (1415 , 1725)** | **16336.5 (14946.5 , 17827.2)** | **-2.7 (-12.6 , 8.3)** | **961 (808 , 1139)** | **9281.9 (8008.2 , 10659.6)** | **-14.4 (-27.6 , 1)** | **45 (35 , 57)** | **467.2 (369.6 , 580.2)** | **-27.9 (-44.7 , -6.2)** |
| **Niue** | **249 (222 , 277)** | **14403.9 (12937.7 , 15969.6)** | **-9.5 (-19.9 , 2.6)** | **114 (97 , 132)** | **6902 (5829.6 , 8065.5)** | **-29.9 (-42.2 , -15.6)** | **5 (4 , 6)** | **283 (222.8 , 355)** | **-46.8 (-59.4 , -29.6)** |
| **Northern Mariana Islands** | **6027 (5227 , 6869)** | **13770.1 (12236.9 , 15403.8)** | **-6.5 (-18.4 , 7.1)** | **2394 (1975 , 2863)** | **6031.2 (5000.8 , 7190.2)** | **-18.2 (-34.6 , 1.9)** | **97 (73 , 126)** | **229 (178.3 , 289.5)** | **-29.1 (-47.9 , -3.9)** |
| **Palau** | **2623 (2313 , 2956)** | **14251 (12721.4 , 15878.6)** | **-8.1 (-18.7 , 4.4)** | **1111 (941 , 1308)** | **6560.7 (5560.1 , 7822.4)** | **-25.9 (-39.7 , -9.9)** | **48 (37 , 61)** | **267.6 (205.5 , 337.5)** | **-38.1 (-53.7 , -18.4)** |
| **Papua New Guinea** | **1555374 (1451899 , 1663840)** | **16299.7 (15253.9 , 17403.8)** | **-3.8 (-12.2 , 5.2)** | **1443007 (1290846 , 1604839)** | **13931.4 (12643.2 , 15291.9)** | **-15.6 (-25.2 , -5.8)** | **123254 (101183 , 148052)** | **1265 (1053.3 , 1484.7)** | **-31.2 (-45.7 , -14.7)** |
| **Samoa** | **24907 (21970 , 27806)** | **12910.1 (11593.1 , 14303)** | **-5.1 (-16.6 , 6.9)** | **14485 (11916 , 17438)** | **6984.9 (5942.5 , 8187.2)** | **-16.6 (-31.7 , 1.3)** | **700 (547 , 891)** | **356.4 (285.4 , 443.6)** | **-28.5 (-45.4 , -6)** |
| **Solomon Islands** | **110152 (101260 , 119650)** | **18163.7 (16791.2 , 19653.6)** | **-1.2 (-9.8 , 8.7)** | **85848 (74111 , 99583)** | **12618.8 (11192.6 , 14300.2)** | **-14.1 (-25 , 0)** | **5098 (4007 , 6614)** | **800.3 (653.6 , 993.1)** | **-32.3 (-46.7 , -11.9)** |
| **Tokelau** | **224 (203 , 247)** | **16018.3 (14496.4 , 17677.8)** | **-7.7 (-17.6 , 2.9)** | **125 (106 , 145)** | **8617 (7398 , 9952)** | **-32.2 (-43.3 , -19.2)** | **6 (5 , 7)** | **415.8 (333.9 , 519.9)** | **-52.4 (-64 , -37.5)** |
| **Tonga** | **15100 (13704 , 16538)** | **15799 (14397.9 , 17239.9)** | **-3.4 (-13.2 , 8.1)** | **8814 (7494 , 10240)** | **8911.3 (7701.9 , 10245.1)** | **-16.1 (-29.8 , 0.2)** | **479 (386 , 598)** | **503.4 (403.5 , 620.5)** | **-28.7 (-45.7 , -6.4)** |
| **Tuvalu** | **1955 (1767 , 2140)** | **17108.3 (15537.4 , 18593)** | **-3.7 (-13.6 , 6.1)** | **1196 (1039 , 1363)** | **10443 (9108.4 , 11830.3)** | **-26.6 (-37.1 , -15.1)** | **67 (54 , 83)** | **594.5 (483.9 , 724.3)** | **-45 (-57.4 , -30.1)** |
| **Vanuatu** | **48124 (44502 , 51931)** | **18003.6 (16761.2 , 19366.2)** | **1 (-8.2 , 10.9)** | **37221 (32957 , 41752)** | **12928.1 (11633.6 , 14293.5)** | **-7 (-18.1 , 5.2)** | **2687 (2175 , 3267)** | **981 (806.9 , 1182.8)** | **-19 (-35.8 , 2.7)** |
| **North Africa and Middle East** | **65541607 (63269515 , 67963640)** | **11039.3 (10682.1 , 11435.2)** | **-23.9 (-26.9 , -20.5)** | **39664787 (37553421 , 41947824)** | **6589.5 (6254 , 6952.5)** | **-36 (-39.6 , -32)** | **2333700 (2157396 , 2519779)** | **390.8 (362.4 , 420.4)** | **-45 (-50 , -39.6)** |
| **Afghanistan** | **3569773 (3285677 , 3874751)** | **9795.6 (9052.3 , 10551.6)** | **-16.7 (-24.9 , -8)** | **3301560 (2823734 , 3867684)** | **8078.6 (7077.2 , 9075.6)** | **-25.6 (-36.2 , -13.2)** | **236420 (188183 , 296397)** | **657.5 (536.9 , 794.2)** | **-37 (-50.5 , -19.7)** |
| **Algeria** | **4607136 (4078702 , 5166072)** | **11066 (9846.7 , 12398.5)** | **-22.3 (-31.5 , -11.7)** | **2357800 (1908320 , 2866653)** | **5649 (4599.9 , 6841.3)** | **-40.3 (-52.5 , -24.8)** | **86225 (65187 , 110981)** | **210.4 (161.7 , 269.9)** | **-59.1 (-70.3 , -45.3)** |
| **Bahrain** | **169814 (147457 , 192724)** | **12003.8 (10567.9 , 13496.6)** | **-25.9 (-35.8 , -14.9)** | **68322 (54895 , 82607)** | **5338.6 (4278.6 , 6511.3)** | **-46.5 (-58.5 , -31)** | **1691 (1246 , 2253)** | **133.1 (100 , 172.6)** | **-66.9 (-76.5 , -54.4)** |
| **Egypt** | **12746023 (11418847 , 14219319)** | **13120.9 (11841.2 , 14612.3)** | **-19.2 (-28.7 , -8.2)** | **6289472 (5169502 , 7624051)** | **6208.2 (5202.4 , 7388.4)** | **-46.2 (-56.7 , -34.6)** | **202116 (153881 , 272640)** | **217.1 (169.9 , 283.3)** | **-68.9 (-77 , -58.5)** |
| **Iran (Islamic Republic of)** | **6951187 (6031031 , 7919370)** | **8446.6 (7419.1 , 9555.4)** | **-37.7 (-46.5 , -28.5)** | **2611288 (2143855 , 3157186)** | **3320.5 (2737.2 , 3981.9)** | **-56.1 (-65 , -45.6)** | **105637 (81914 , 136109)** | **134.9 (105.9 , 171.1)** | **-68.3 (-76.4 , -58.4)** |
| **Iraq** | **4518532 (3989848 , 5067578)** | **11304.3 (10103.6 , 12587.8)** | **-20.3 (-29.5 , -10)** | **2407123 (1935244 , 2897080)** | **5864.8 (4855 , 6927.8)** | **-37.6 (-48.5 , -23.9)** | **91146 (69652 , 117717)** | **241 (188 , 306.7)** | **-57.5 (-67.7 , -43.6)** |
| **Jordan** | **1188097 (1080807 , 1316025)** | **10642.6 (9686.3 , 11737.9)** | **-21.5 (-30.8 , -11.8)** | **702988 (603677 , 809975)** | **6078.7 (5289.8 , 6949.7)** | **-38.2 (-48 , -26.3)** | **31743 (26222 , 38475)** | **283.3 (233.5 , 342.4)** | **-59.9 (-69 , -47.7)** |
| **Kuwait** | **389856 (346160 , 442167)** | **8913.6 (7952.2 , 9930.3)** | **-19.5 (-29.9 , -7.3)** | **187559 (152625 , 224525)** | **4372.1 (3610.8 , 5197.6)** | **-33.2 (-46 , -16.8)** | **7707 (5793 , 10264)** | **170.8 (133.9 , 219)** | **-52.4 (-64.5 , -36.5)** |
| **Lebanon** | **415529 (352371 , 486586)** | **7980.6 (6737.5 , 9336.5)** | **-43 (-51.9 , -32.3)** | **143723 (111024 , 186325)** | **2790.9 (2141.2 , 3638.1)** | **-63 (-72.1 , -50)** | **3525 (2630 , 4611)** | **67.4 (50.3 , 87.3)** | **-77.8 (-83.8 , -69.9)** |
| **Libya** | **778502 (687636 , 871985)** | **11957.3 (10636.6 , 13248.2)** | **-14.5 (-25.4 , -2.7)** | **400533 (328663 , 480539)** | **6395.5 (5239.9 , 7681.7)** | **-27.6 (-41.4 , -10.1)** | **16112 (12113 , 21456)** | **254.6 (196.3 , 327.2)** | **-45 (-59.5 , -26.3)** |
| **Morocco** | **4553041 (4139437 , 5047054)** | **12850.1 (11744.6 , 14204)** | **-17.1 (-26.1 , -7.2)** | **2702521 (2275742 , 3189403)** | **7765 (6527.7 , 9097.6)** | **-35.8 (-46.8 , -22.8)** | **122748 (95204 , 155699)** | **356.2 (279.5 , 451.1)** | **-57.2 (-67.4 , -44.2)** |
| **Palestine** | **545255 (490513 , 610551)** | **11504.6 (10411.7 , 12767.8)** | **-18.4 (-27.8 , -7)** | **268194 (223968 , 315083)** | **5421 (4643.8 , 6237.8)** | **-40.5 (-51.7 , -27)** | **8061 (6463 , 9832)** | **189.1 (152.9 , 228)** | **-60.2 (-69.9 , -48.1)** |
| **Oman** | **624695 (533249 , 723492)** | **15379.2 (13706.5 , 17200.4)** | **-24.1 (-34.4 , -13.8)** | **239003 (189988 , 296423)** | **6783.9 (5486.9 , 8365.8)** | **-53.4 (-62.7 , -41.7)** | **5530 (4306 , 7026)** | **197.5 (155.9 , 248)** | **-69.3 (-76.8 , -59.5)** |
| **Qatar** | **193291 (156260 , 239943)** | **7708.6 (6380.7 , 9142.7)** | **-42.8 (-53.2 , -31.5)** | **55159 (42666 , 70498)** | **2330.4 (1804.2 , 3020)** | **-62.9 (-71.8 , -51.9)** | **1613 (1083 , 2320)** | **63 (46.7 , 82.7)** | **-79.5 (-85 , -72.2)** |
| **Saudi Arabia** | **2231078 (1837900 , 2695098)** | **6991.4 (5925.6 , 8096.8)** | **-35.4 (-46 , -23.7)** | **719751 (552917 , 918179)** | **2493.6 (1961.9 , 3130.8)** | **-51.4 (-62.3 , -38.5)** | **24605 (17562 , 32885)** | **87.7 (66.2 , 111.5)** | **-70.9 (-78.8 , -61.2)** |
| **Sudan** | **5808310 (5330124 , 6358168)** | **14713.1 (13567.4 , 16089.1)** | **-10.3 (-18.3 , -1)** | **4891300 (4267749 , 5557131)** | **11013.9 (9676.5 , 12374.9)** | **-27.3 (-37 , -15.8)** | **275609 (220030 , 343360)** | **643.6 (525.4 , 778.1)** | **-52.7 (-62.7 , -39.4)** |
| **Syrian Arab Republic** | **1682965 (1488907 , 1887693)** | **12128.8 (10814.8 , 13526.4)** | **-18.9 (-28.6 , -7.5)** | **877693 (714838 , 1053311)** | **6402.7 (5231.4 , 7715.7)** | **-38 (-51.1 , -23.7)** | **32051 (24555 , 41852)** | **235.8 (182.7 , 299.6)** | **-60.1 (-70.5 , -46.4)** |
| **Tunisia** | **963684 (839074 , 1097859)** | **8348.8 (7284.9 , 9502.5)** | **-29.4 (-39.6 , -18)** | **364745 (289264 , 451362)** | **3288.8 (2579.2 , 4083.2)** | **-46.1 (-57.9 , -31.8)** | **12613 (9258 , 16703)** | **111.2 (81.5 , 147.4)** | **-62.7 (-73.2 , -48.5)** |
| **Turkey** | **8216569 (7237063 , 9187612)** | **10220.5 (8997.5 , 11436.4)** | **-31.1 (-40.3 , -21.3)** | **3715476 (3047718 , 4436269)** | **4873.7 (3935.9 , 5943.5)** | **-54.3 (-63.9 , -43)** | **127696 (95603 , 165998)** | **160.5 (121.1 , 207.4)** | **-74.6 (-81.7 , -65.2)** |
| **United Arab Emirates** | **878300 (706342 , 1114446)** | **10545.6 (9117.1 , 12191.6)** | **-21.6 (-33.4 , -7.5)** | **341573 (275891 , 415588)** | **4720.3 (3813.5 , 5793.6)** | **-35.6 (-48.9 , -18.8)** | **14425 (10123 , 19871)** | **181.4 (137.4 , 237.6)** | **-53.3 (-65.4 , -36.7)** |
| **Yemen** | **4443380 (4131480 , 4794259)** | **14772.5 (13705.7 , 15850.9)** | **-3 (-11.2 , 6)** | **6978705 (6464188 , 7484974)** | **19855.6 (18638.5 , 21137)** | **13.2 (3.3 , 24.2)** | **924057 (794664 , 1069972)** | **2609.1 (2266.7 , 2983)** | **5.9 (-15.1 , 32.2)** |
| **South Asia** | **351205972 (346260524 , 356081870)** | **19927.1 (19669.3 , 20184.5)** | **-2.4 (-4.2 , -0.8)** | **347120676 (339860696 , 353977035)** | **19790 (19396.2 , 20176.7)** | **-17.2 (-19.1 , -15.2)** | **32816471 (31684532 , 34025864)** | **1929.1 (1866.7 , 1995.2)** | **-40.6 (-43 , -38.1)** |
| **Bangladesh** | **29358283 (27144435 , 31810243)** | **18998.6 (17666.2 , 20449.5)** | **-5.4 (-13.6 , 2.8)** | **18843353 (16732034 , 21183861)** | **12426.8 (11104.2 , 13888.4)** | **-37.4 (-45.6 , -27.6)** | **1003303 (829847 , 1195983)** | **684.4 (571.6 , 813.9)** | **-63.7 (-70.9 , -54.9)** |
| **Bhutan** | **144256 (134201 , 154604)** | **19669.2 (18398.6 , 20918)** | **2.6 (-4.9 , 12.3)** | **168510 (155141 , 183222)** | **24082.6 (22160.4 , 26087)** | **-18 (-26.2 , -9.6)** | **10639 (8441 , 13164)** | **1548 (1238.8 , 1913.8)** | **-56.8 (-66.7 , -44.4)** |
| **India** | **275219060 (272132499 , 278278881)** | **20140.5 (19939.2 , 20350.3)** | **-2.1 (-3.7 , -0.7)** | **280284306 (275942854 , 284445548)** | **20861 (20552.5 , 21161.8)** | **-16.2 (-17.7 , -14.6)** | **27884076 (26981319 , 28845926)** | **2124.5 (2058.4 , 2195)** | **-40.1 (-42.6 , -37.5)** |
| **Nepal** | **5702246 (5312939 , 6158591)** | **19461.4 (18255.4 , 20827.3)** | **-5 (-12.4 , 3.9)** | **4303640 (3853798 , 4832388)** | **14777.7 (13326.9 , 16423.2)** | **-25.4 (-34.2 , -14.9)** | **244130 (204486 , 286520)** | **900.1 (756.3 , 1052.6)** | **-53.1 (-61.7 , -42.3)** |
| **Pakistan** | **40782127 (38053714 , 43601618)** | **19413.4 (18255.7 , 20648.8)** | **0.6 (-6.5 , 8.7)** | **43520869 (38748807 , 48501061)** | **18878.5 (17178 , 20625.7)** | **-11.3 (-20 , -0.7)** | **3674323 (3033672 , 4434420)** | **1739.6 (1478 , 2036.8)** | **-34.3 (-46 , -21.1)** |
| **Southern Sub-Saharan Africa** | **9055237 (8664556 , 9456937)** | **11833.3 (11349.5 , 12353.3)** | **-11.8 (-17.9 , -5.2)** | **6627141 (6147505 , 7120433)** | **8479.2 (7897.4 , 9095)** | **-18 (-26.2 , -9.1)** | **502985 (449095 , 562641)** | **655.2 (587.5 , 729.8)** | **-25 (-38.2 , -10.1)** |
| **Botswana** | **300669 (273092 , 329620)** | **13173.3 (12096.3 , 14369.2)** | **-14.2 (-22.1 , -5.7)** | **226259 (190894 , 264257)** | **9538.2 (8139.3 , 11042)** | **-33.2 (-43.4 , -21.6)** | **11315 (8849 , 14457)** | **489.9 (395.7 , 616)** | **-52 (-62.9 , -37.7)** |
| **Lesotho** | **261618 (246584 , 276804)** | **13041.3 (12343.2 , 13784.3)** | **-2.2 (-10.9 , 6.4)** | **222004 (198438 , 247786)** | **10697.6 (9655.3 , 11842.5)** | **-7.5 (-19.7 , 6.4)** | **16063 (13568 , 19135)** | **801.6 (680.8 , 934.6)** | **-17.6 (-34 , 3.8)** |
| **Namibia** | **298155 (278845 , 317614)** | **12604.5 (11866.8 , 13375.6)** | **-16.6 (-23.6 , -8.8)** | **236393 (204701 , 267828)** | **9311.5 (8174.2 , 10474.7)** | **-36.5 (-45.1 , -26.6)** | **13210 (10834 , 16062)** | **557.1 (472.5 , 660.4)** | **-58.7 (-67.5 , -47.5)** |
| **South Africa** | **5823206 (5452981 , 6221888)** | **10663.9 (10017.8 , 11354.5)** | **-15 (-23.2 , -5.8)** | **4042104 (3648443 , 4461593)** | **7317.4 (6629.6 , 8077.8)** | **-23.6 (-34.4 , -11.7)** | **363522 (314924 , 421116)** | **650.9 (568.8 , 754.4)** | **-28.8 (-44.5 , -8.9)** |
| **Eswatini** | **139989 (128360 , 154218)** | **12759.4 (11769.6 , 13909.9)** | **-7.2 (-16.3 , 3.1)** | **100509 (85011 , 116825)** | **8762.2 (7628.9 , 9990.3)** | **-17.2 (-29.9 , -1.4)** | **5594 (4474 , 6815)** | **543.2 (447.2 , 652.7)** | **-27.2 (-43.9 , -6)** |
| **Zimbabwe** | **2231600 (2088312 , 2375540)** | **15190.7 (14317 , 16078)** | **-5.5 (-13.1 , 2.9)** | **1799871 (1576882 , 2032644)** | **11515.1 (10293.7 , 12760.5)** | **0.3 (-14.6 , 18.1)** | **93279 (78458 , 110779)** | **694.8 (587.5 , 810.8)** | **19.6 (-6 , 52.8)** |
| **Western Sub-Saharan Africa** | **86203909 (83484092 , 88825027)** | **19479 (18906.3 , 20102.6)** | **4.4 (0.5 , 8.3)** | **110218651 (105654109 , 114888946)** | **19900.3 (19155.7 , 20661.7)** | **-1.2 (-5.6 , 3.4)** | **9184640 (8238052 , 10324254)** | **1597.7 (1451.8 , 1772.4)** | **-25.8 (-34.1 , -16.1)** |
| **Benin** | **2205657 (2062898 , 2359023)** | **17899.8 (16726.7 , 19123.3)** | **-0.6 (-8 , 7.5)** | **2704858 (2437324 , 2996655)** | **17364.1 (15794 , 18992.8)** | **-2.2 (-12.8 , 9.3)** | **165660 (128901 , 209416)** | **1054 (859.3 , 1301)** | **-14 (-32.1 , 10.8)** |
| **Burkina Faso** | **4275550 (4041801 , 4507066)** | **19736.1 (18668.3 , 20793)** | **-1.9 (-9.1 , 6)** | **7055384 (6551669 , 7540383)** | **24202.8 (22555.2 , 25739.5)** | **-0.8 (-9.3 , 8.7)** | **680423 (559365 , 809116)** | **2178.4 (1833.7 , 2541.2)** | **-9.3 (-30 , 16)** |
| **Cameroon** | **4605392 (4306343 , 4934960)** | **16486.3 (15491.5 , 17646.1)** | **6 (-2.3 , 15.4)** | **4597612 (4079656 , 5120337)** | **13731 (12408.5 , 15153.1)** | **-1.9 (-13.8 , 11.8)** | **269121 (217628 , 339418)** | **832.2 (691.8 , 1010.5)** | **-28.5 (-44.4 , -8.2)** |
| **Cabo Verde** | **85129 (76899 , 93184)** | **15293.8 (13909.8 , 16707.6)** | **-8.2 (-17.7 , 2.4)** | **58582 (49038 , 68308)** | **10426.5 (8711.1 , 12132.4)** | **-27.3 (-40.2 , -13.8)** | **2104 (1605 , 2750)** | **373.9 (287.2 , 491.7)** | **-53 (-66.5 , -34.8)** |
| **Chad** | **3020921 (2821860 , 3226349)** | **18781.3 (17532.5 , 20077.5)** | **3 (-4.9 , 11.1)** | **3996288 (3601038 , 4425339)** | **18612 (16944.2 , 20385.6)** | **-3.4 (-14 , 8.4)** | **311899 (236694 , 406410)** | **1457.4 (1171 , 1790.2)** | **-16.5 (-36.6 , 9.1)** |
| **CÃ´te d'Ivoire** | **5315774 (5073967 , 5560519)** | **20939.4 (20014.3 , 21920)** | **-4.3 (-10.4 , 1.8)** | **6300310 (5782386 , 6803407)** | **20702.7 (19245 , 22257.4)** | **-12.8 (-20.6 , -4.1)** | **431538 (354540 , 520593)** | **1440.2 (1209.1 , 1693.3)** | **-31.3 (-45.5 , -14)** |
| **Gambia** | **438590 (413013 , 466012)** | **19682.6 (18451.5 , 20937.1)** | **4 (-3.7 , 12.6)** | **592512 (546434 , 638977)** | **22400.7 (20783.4 , 23924.8)** | **-6.7 (-15.6 , 2)** | **43688 (35239 , 53018)** | **1704.1 (1414.3 , 2021.2)** | **-32.3 (-47.6 , -13.9)** |
| **Ghana** | **6082770 (5634742 , 6587594)** | **19740.4 (18374.9 , 21173.4)** | **2.7 (-6.7 , 13.4)** | **6018410 (5381627 , 6656249)** | **17665.1 (15973.1 , 19354.9)** | **-10.7 (-21.3 , 1.4)** | **365790 (294244 , 448184)** | **1085.8 (882.2 , 1312.1)** | **-41 (-54.3 , -21.7)** |
| **Guinea** | **2327401 (2205251 , 2456862)** | **19228.8 (18189.9 , 20257.2)** | **3.5 (-3.4 , 11.3)** | **2957647 (2715050 , 3220665)** | **19356.1 (17835.5 , 20905.1)** | **-1.4 (-11.5 , 9.8)** | **239987 (196300 , 287125)** | **1527.6 (1291.5 , 1783.1)** | **-21.1 (-36.9 , -1.4)** |
| **Guinea-Bissau** | **348444 (321774 , 374498)** | **18759.6 (17424.8 , 19995.6)** | **1.8 (-6.1 , 10.7)** | **410614 (366451 , 456166)** | **18406 (16613.5 , 20239.9)** | **-7.6 (-17.8 , 3.4)** | **30010 (22797 , 38128)** | **1356.6 (1061 , 1657.6)** | **-28.4 (-45.4 , -7.3)** |
| **Liberia** | **896605 (829829 , 964969)** | **19229.9 (17888.3 , 20607.4)** | **-1.2 (-8.6 , 7.2)** | **913642 (814436 , 1016001)** | **17008.1 (15345.6 , 18678.2)** | **-22.6 (-31.1 , -13.1)** | **53658 (42268 , 68668)** | **1031 (827.7 , 1270.5)** | **-48.5 (-60 , -33.1)** |
| **Mali** | **4222341 (3971522 , 4479412)** | **20189 (18969.8 , 21382.6)** | **3.8 (-4.1 , 12.4)** | **6657479 (6187060 , 7113830)** | **24075.3 (22405.3 , 25600.8)** | **-1.8 (-9.9 , 6.8)** | **791128 (642439 , 958593)** | **2625.8 (2177.4 , 3115.1)** | **-24.4 (-40 , -4.9)** |
| **Mauritania** | **728154 (670564 , 785997)** | **18352.7 (16954.8 , 19788.8)** | **-0.8 (-9.2 , 8.1)** | **748400 (654898 , 845505)** | **15867.4 (14055.8 , 17783.1)** | **-14.3 (-25.2 , -2.2)** | **37658 (27996 , 49686)** | **819 (630.1 , 1045.4)** | **-43.1 (-58.2 , -23.5)** |
| **Niger** | **4459879 (4185745 , 4720659)** | **19233 (18115.8 , 20353.7)** | **-1.4 (-9 , 6.7)** | **6512124 (5934819 , 7136535)** | **20590.2 (19010.7 , 22360.4)** | **-12.7 (-21 , -3.4)** | **469396 (361622 , 592660)** | **1489.1 (1205.7 , 1823)** | **-37.8 (-51.7 , -18.9)** |
| **Nigeria** | **40835671 (38446713 , 43310265)** | **19654.2 (18541.9 , 20861.7)** | **9.4 (1 , 18.4)** | **53083674 (48797877 , 57541715)** | **20203.5 (18749.6 , 21749.8)** | **6.1 (-3 , 15.3)** | **4774742 (3899914 , 5840117)** | **1719.6 (1427.9 , 2066.1)** | **-25.3 (-40.2 , -5.7)** |
| **Sao Tome and Principe** | **39310 (36184 , 42485)** | **19213.8 (17809.9 , 20582.9)** | **-5 (-14.1 , 4.8)** | **27673 (22813 , 33062)** | **12347.9 (10453.2 , 14466)** | **-25.8 (-39.4 , -9.1)** | **847 (674 , 1038)** | **413.7 (337.9 , 502.6)** | **-50.1 (-61.9 , -34.7)** |
| **Senegal** | **3128146 (2982865 , 3264118)** | **20938.6 (19939.3 , 21871.1)** | **3.3 (-3.5 , 10.7)** | **3553235 (3268633 , 3836800)** | **20488.5 (18995.2 , 22040.3)** | **-15.1 (-22.9 , -6.2)** | **237020 (197620 , 282709)** | **1399.3 (1188.4 , 1636.2)** | **-48 (-58.8 , -33.9)** |
| **Sierra Leone** | **1675361 (1598227 , 1755768)** | **20831.9 (19902.5 , 21825.2)** | **-1.1 (-7.8 , 5.8)** | **2123646 (1959874 , 2280351)** | **21572.6 (20081.7 , 23084.5)** | **-4 (-13.2 , 5.7)** | **166771 (136272 , 202351)** | **1664.2 (1391.7 , 1979.8)** | **-22.7 (-39.8 , -0.4)** |
| **Togo** | **1511608 (1442984 , 1582013)** | **19281.1 (18435.2 , 20147.6)** | **-3.8 (-10.5 , 3.1)** | **1905020 (1745977 , 2066035)** | **20677.6 (19124.4 , 22234.9)** | **-9.5 (-17.8 , 0)** | **113073 (92043 , 138054)** | **1257.6 (1054.4 , 1489.4)** | **-6.9 (-26.5 , 17.9)** |
| **Eastern Sub-Saharan Africa** | **67178382 (65916896 , 68381315)** | **17006.2 (16703.6 , 17293.5)** | **-4.9 (-6.8 , -3)** | **66827382 (64755699 , 69191145)** | **14549.9 (14178.3 , 14968.7)** | **-23.2 (-25.6 , -20.6)** | **4920268 (4640509 , 5205106)** | **1143.4 (1089 , 1196.8)** | **-45.4 (-48.6 , -42.2)** |
| **Burundi** | **1786621 (1678982 , 1899150)** | **15308.3 (14424.4 , 16210.2)** | **-7.7 (-15.6 , 0.2)** | **1925879 (1709454 , 2157613)** | **13522.5 (12241.1 , 14824.1)** | **-16.3 (-26.6 , -4.3)** | **112347 (90281 , 142953)** | **867.3 (731.6 , 1037.8)** | **-36.7 (-50.9 , -18)** |
| **Comoros** | **128710 (118740 , 139369)** | **18551.4 (17202.5 , 19997.9)** | **-5.9 (-14 , 2.5)** | **103130 (89897 , 117599)** | **14333.2 (12697.5 , 16080.4)** | **-26 (-34.8 , -16)** | **6420 (5119 , 7952)** | **915.4 (741 , 1117.6)** | **-48.8 (-59.7 , -34)** |
| **Djibouti** | **205767 (187271 , 224990)** | **17755.5 (16298.5 , 19326.8)** | **-7.4 (-15 , 2.1)** | **156354 (132957 , 178996)** | **12564.4 (10916.9 , 14146.8)** | **-26.4 (-36.3 , -15.3)** | **8884 (7053 , 11180)** | **741.9 (606.9 , 911.9)** | **-46.6 (-58.9 , -32.5)** |
| **Eritrea** | **1182651 (1087762 , 1282910)** | **18461.5 (17167.7 , 19821.3)** | **-5 (-12.7 , 3.5)** | **1084681 (950617 , 1219776)** | **15402.6 (13788 , 17064.6)** | **-26.3 (-34.3 , -17.6)** | **74201 (58759 , 93904)** | **1110.2 (901.4 , 1350)** | **-50.8 (-61.9 , -38.1)** |
| **Ethiopia** | **14128463 (13689157 , 14601633)** | **13430.2 (13046.8 , 13868.6)** | **-9.5 (-13 , -5.6)** | **14410664 (13525331 , 15403611)** | **11207.2 (10614.1 , 11909.8)** | **-25.4 (-29.7 , -20.3)** | **1022905 (922243 , 1139350)** | **827.9 (760.9 , 905)** | **-49.7 (-55.2 , -43.9)** |
| **Kenya** | **6423961 (6287349 , 6553918)** | **13582.3 (13344.4 , 13817.2)** | **-2.2 (-4.4 , -0.1)** | **5033374 (4922925 , 5142203)** | **10217.2 (10035.4 , 10403.7)** | **-16.4 (-18.4 , -14.2)** | **444746 (426385 , 462537)** | **1028.9 (990.2 , 1067.8)** | **-26.8 (-29.9 , -23.4)** |
| **Madagascar** | **5016786 (4640458 , 5410398)** | **19852.8 (18483.5 , 21256.1)** | **-2.4 (-10.2 , 6.7)** | **3924835 (3439528 , 4429916)** | **14139.9 (12607.8 , 15605.1)** | **-23.7 (-32.8 , -13)** | **203695 (166415 , 248211)** | **826.4 (689.4 , 978.6)** | **-46.1 (-56.9 , -32.4)** |
| **Malawi** | **3501762 (3246272 , 3740567)** | **19930.1 (18587.4 , 21193)** | **-2.6 (-10.6 , 5.6)** | **3811011 (3422221 , 4199682)** | **18655 (17109.2 , 20193.2)** | **-21.7 (-29.2 , -13.5)** | **251425 (204291 , 307045)** | **1317.3 (1105.3 , 1543.2)** | **-50.8 (-60.7 , -39)** |
| **Mozambique** | **5567457 (5234374 , 5912478)** | **19802.5 (18579 , 21082.9)** | **-0.8 (-8.3 , 6.9)** | **6496548 (5939428 , 7084088)** | **18940.2 (17551.6 , 20387.5)** | **-17.4 (-25.2 , -9)** | **478816 (399484 , 578331)** | **1447 (1228.9 , 1696.6)** | **-46.4 (-56.5 , -33.5)** |
| **Rwanda** | **1764417 (1590280 , 1960523)** | **15049.8 (13756.1 , 16510.5)** | **-11.8 (-21.1 , -2)** | **1168544 (1003024 , 1341884)** | **9238.3 (8075.3 , 10330.1)** | **-36.8 (-44.9 , -27.1)** | **67810 (53625 , 87337)** | **567.4 (461.9 , 696.7)** | **-61.7 (-69.8 , -52.1)** |
| **Somalia** | **3699477 (3445914 , 3967186)** | **19281.8 (18013.6 , 20551.6)** | **-3.5 (-11 , 4)** | **4307145 (3886754 , 4715973)** | **19419.1 (17957.1 , 20847)** | **-18.3 (-25.9 , -10.6)** | **401088 (329652 , 486246)** | **1917.8 (1606.3 , 2251.4)** | **-37.6 (-49.8 , -23.4)** |
| **South Sudan** | **1695469 (1568924 , 1814402)** | **19292 (18027.4 , 20541.4)** | **-3.9 (-11.3 , 3.8)** | **1794667 (1593343 , 2009178)** | **17301.3 (15703.5 , 18971.8)** | **-11.1 (-20.7 , -0.1)** | **139849 (111022 , 173388)** | **1423.8 (1187.3 , 1702)** | **-23.6 (-39.1 , -3)** |
| **United Republic of Tanzania** | **12015942 (11246717 , 12783884)** | **21605.1 (20277.3 , 22901.1)** | **-6.3 (-13 , 0.4)** | **11631970 (10319529 , 13011713)** | **18173 (16381.5 , 19978.5)** | **-35.1 (-42.5 , -27.3)** | **675217 (542612 , 836535)** | **1125.2 (929.7 , 1348.7)** | **-60.7 (-68.9 , -51.4)** |
| **Uganda** | **6177748 (5733922 , 6631148)** | **15928.3 (14937.2 , 16986.1)** | **-7.9 (-15.3 , 0.6)** | **6216813 (5498807 , 7017344)** | **13325.5 (12030.3 , 14664)** | **-27.8 (-36 , -18.7)** | **400467 (320213 , 491244)** | **900.9 (754.4 , 1063.9)** | **-57 (-66.2 , -46.1)** |
| **Zambia** | **3829444 (3621774 , 4032244)** | **22489.4 (21353 , 23580)** | **12.5 (5.5 , 19.7)** | **4708343 (4357104 , 5038879)** | **23627.5 (22049.3 , 25106.6)** | **-2.1 (-9.9 , 6.1)** | **628464 (520621 , 754219)** | **3210.2 (2728.2 , 3722.2)** | **-19.4 (-34 , -2.2)** |
| **Central Sub-Saharan Africa** | **24207433 (23298001 , 25096856)** | **19003 (18347.8 , 19679.7)** | **-5.3 (-9.9 , -0.6)** | **24932103 (23134150 , 26764270)** | **16720 (15685.3 , 17755.3)** | **-23.3 (-29.3 , -17.2)** | **1618251 (1368628 , 1900837)** | **1138.4 (995.1 , 1291)** | **-51 (-58.8 , -41.7)** |
| **Angola** | **5260813 (4838739 , 5686433)** | **18171.1 (16717.5 , 19544.7)** | **1.2 (-7.8 , 11)** | **4314027 (3730628 , 4995559)** | **12319.6 (10889.2 , 13944.7)** | **-13 (-24.7 , 0.9)** | **207864 (167698 , 254758)** | **636.6 (531.4 , 759.7)** | **-49.3 (-59.7 , -36.2)** |
| **Central African Republic** | **1032761 (971624 , 1096069)** | **19694.6 (18614.4 , 20792.4)** | **-1.2 (-8.3 , 6.1)** | **1169044 (1048958 , 1295023)** | **19119.2 (17433.7 , 20932.9)** | **-5.4 (-16.1 , 6.5)** | **66856 (53103 , 84481)** | **1250.1 (1038.1 , 1498.9)** | **1 (-18.6 , 27)** |
| **Congo** | **1090765 (1021310 , 1166672)** | **21244.3 (19917.7 , 22711.9)** | **2.3 (-5.6 , 11.1)** | **988246 (887007 , 1097103)** | **17496.9 (15964.1 , 19200.1)** | **-13.5 (-23.1 , -1.9)** | **44938 (36523 , 54686)** | **848.9 (715.4 , 1001.9)** | **-41.6 (-53.7 , -25.8)** |
| **Democratic Republic of the Congo** | **16177160 (15382880 , 16956279)** | **19022.5 (18138.4 , 19886.7)** | **-7.5 (-13.6 , -1.4)** | **17859961 (16281733 , 19498215)** | **17999 (16560.2 , 19389.3)** | **-25 (-32.5 , -17.2)** | **1267647 (1027079 , 1540450)** | **1326.5 (1119.1 , 1559.9)** | **-51.7 (-61 , -39.8)** |
| **Equatorial Guinea** | **269661 (244634 , 295272)** | **19677.8 (18293.2 , 21272.2)** | **-3.7 (-11.8 , 4.2)** | **227601 (194922 , 262045)** | **14695.2 (12912.9 , 16622)** | **-36.7 (-45 , -28.4)** | **10234 (7851 , 13291)** | **694 (555.1 , 868)** | **-71.3 (-77.9 , -62.6)** |
| **Gabon** | **376273 (357618 , 396100)** | **21786.9 (20743 , 22830.2)** | **1.2 (-5.9 , 8.7)** | **373224 (340686 , 408048)** | **20642.5 (18950.4 , 22367.6)** | **-12.9 (-21.8 , -3.3)** | **20712 (17544 , 24483)** | **1196.8 (1022.2 , 1416.7)** | **-41.4 (-53.7 , -26.3)** |
