# Supplementary material for: Burden of anemia and its underlying causes in 204 countries and territories, 1990–2019: results from the Global Burden of Disease Study 2019
Source: J Hematol Oncol. 2021 Nov 4;14:185. doi: 10.1186/s13045-021-01202-2 (PMC8567696; doi:10.1186/s13045-021-01202-2)
Supplement: Supplementary file 3 — Additional file 3: Table S3. Years lived with disability (YLDs) due to anemia in 2019 and the percentage change in the age-standardized rates (ASRs) from 1990 to 2019, by severity and location (Generated from data available from http://ghdx.healthdata.org/gbd-results-tool). [file 13045_2021_1202_MOESM3_ESM.doc]

| **Additional file 3: Table S3. YLDs due to anemia in 1990 and 2019 and the percentage change in the age-standardised rates (ASRs) per 100,000 by severity and location**  **(Generated from data available from http://ghdx.healthdata.org/gbd-results-tool)** | | | | | | | | | |
| --- | --- | --- | --- | --- | --- | --- | --- | --- | --- |
|  | **Mild** | | | **Moderate** | | | **Severe** | | |
|  | **No (95% UI)**  **2019** | **ASRs per 100,000 (95% UI)**  **2019** | **Percentage change in ASRs per 100,000**  **(1990 - 2019)** | **No (95% UI)**  **2019** | **ASRs per 100,000 (95% UI)**  **2019** | **Percentage change in ASRs per 100,000**  **(1990 - 2019)** | **No (95% UI)**  **2019** | **ASRs per 100,000 (95% UI)**  **2019** | **Percentage change in ASRs per 100,000**  **(1990 - 2019)** |
| **Global** | **3511875 (1256549 , 7744270)** | **45.5 (16.3 , 100.2)** | **-10 (-11 , -8.8)** | **38130541 (25010516 , 55924692)** | **511.8 (335.6 , 751.2)** | **-15.1 (-16.6 , -13.5)** | **8654069 (5931918 , 12141035)** | **115.2 (78.9 , 161.8)** | **-34.1 (-36.6 , -31.3)** |
| **High-income North America** | **77745 (27073 , 169562)** | **19.1 (6.6 , 41.6)** | **-10.1 (-20.9 , 4.4)** | **329585 (212308 , 494192)** | **85.4 (54.6 , 130)** | **-10.5 (-26.3 , 10.7)** | **36667 (23723 , 54048)** | **8.4 (5.3 , 12.6)** | **6.6 (-20.3 , 41.6)** |
| **Canada** | **4991 (1750 , 11287)** | **12 (4.1 , 26.8)** | **-25.8 (-42.7 , -5.6)** | **18354 (11093 , 27774)** | **47.8 (28.7 , 73.3)** | **-33.5 (-52.4 , -8.8)** | **1682 (976 , 2654)** | **3.9 (2.2 , 6.2)** | **-42.4 (-64 , -6.2)** |
| **Greenland** | **16 (6 , 35)** | **27.1 (9.7 , 61.1)** | **-20.8 (-34.6 , -5.8)** | **81 (50 , 125)** | **148.5 (89.4 , 224.9)** | **-32.2 (-47.3 , -11.7)** | **10 (6 , 16)** | **17.9 (10.9 , 27.6)** | **-39.8 (-58 , -12)** |
| **United States of America** | **72737 (25226 , 159478)** | **19.9 (6.9 , 43.5)** | **-8.7 (-20.4 , 6.3)** | **311144 (200185 , 466705)** | **89.6 (57.3 , 136.3)** | **-8.5 (-26.1 , 14)** | **34974 (22389 , 52054)** | **8.9 (5.6 , 13.4)** | **11.6 (-18.3 , 49.8)** |
| **Australasia** | **5542 (1966 , 12767)** | **19.2 (6.8 , 45.1)** | **-24.8 (-39.8 , -6.9)** | **21810 (13299 , 32161)** | **78.4 (47 , 118)** | **-36.9 (-54.4 , -12.4)** | **1791 (1113 , 2750)** | **5.2 (3.2 , 8.2)** | **-47.5 (-63.9 , -23)** |
| **Australia** | **4642 (1651 , 10969)** | **18.9 (6.8 , 45.7)** | **-25.9 (-42.7 , -5)** | **17810 (10687 , 26828)** | **74.7 (43.5 , 116.5)** | **-38.2 (-57.4 , -10.4)** | **1458 (863 , 2332)** | **5 (2.8 , 8.1)** | **-48.4 (-67 , -19.1)** |
| **New Zealand** | **900 (321 , 2084)** | **20.7 (7.1 , 48.1)** | **-19.7 (-35.7 , -0.3)** | **4000 (2407 , 6232)** | **97.3 (56.9 , 156.5)** | **-30.5 (-54.2 , 3.8)** | **333 (192 , 520)** | **6.5 (3.7 , 10.3)** | **-42.7 (-62.2 , -15)** |
| **High-income Asia Pacific** | **68020 (24345 , 152143)** | **29.7 (10.5 , 66.6)** | **-30 (-38 , -20.4)** | **310341 (199900 , 467725)** | **148.5 (94.9 , 222.8)** | **-50.3 (-57.8 , -41.2)** | **35380 (23013 , 51767)** | **16.5 (10.6 , 24.4)** | **-67.8 (-74.2 , -59.8)** |
| **Brunei Darussalam** | **152 (56 , 341)** | **36.5 (13.4 , 83.2)** | **-18.9 (-29.6 , -7.2)** | **919 (578 , 1415)** | **226.1 (141.8 , 347.4)** | **-35.1 (-47.2 , -20)** | **130 (81 , 197)** | **31.1 (19.7 , 45.7)** | **-50.9 (-63.1 , -34.9)** |
| **Japan** | **49739 (18003 , 112851)** | **29.8 (10.7 , 66.6)** | **-21.8 (-33.9 , -6.3)** | **224532 (142923 , 340809)** | **144.4 (89.3 , 220.2)** | **-37.6 (-51.1 , -20.4)** | **22014 (13922 , 33003)** | **12.4 (7.7 , 18.8)** | **-50.8 (-64.3 , -30.5)** |
| **Singapore** | **1667 (604 , 3810)** | **26.9 (9.5 , 60.7)** | **-36 (-46.1 , -23.9)** | **7055 (4288 , 10780)** | **120.9 (72.4 , 184.4)** | **-54.9 (-65.1 , -42.4)** | **783 (464 , 1210)** | **12.4 (7.4 , 19)** | **-68.8 (-77.7 , -57.4)** |
| **Republic of Korea** | **16462 (5683 , 37999)** | **28.8 (10 , 65.9)** | **-46.4 (-54.3 , -37.3)** | **77836 (48514 , 118981)** | **155 (95.7 , 243.3)** | **-67.9 (-73.9 , -60.6)** | **12453 (7737 , 18657)** | **24.7 (15.5 , 38.2)** | **-79.4 (-84.6 , -72.5)** |
| **Western Europe** | **65039 (23190 , 143465)** | **14.1 (5.1 , 31.5)** | **-33.3 (-39.1 , -26.4)** | **221605 (142650 , 325383)** | **52.3 (33.5 , 77.8)** | **-42.8 (-50.7 , -33.9)** | **19943 (12962 , 28415)** | **4.4 (2.8 , 6.3)** | **-47.7 (-58.3 , -34.8)** |
| **Andorra** | **11 (4 , 25)** | **12.3 (4.5 , 28.7)** | **-29 (-47.5 , -3.7)** | **32 (19 , 52)** | **39.7 (22.3 , 67.2)** | **-36.8 (-59.2 , -2.4)** | **2 (1 , 4)** | **2 (1.1 , 3.5)** | **-47.1 (-68.2 , -11.6)** |
| **Austria** | **1247 (427 , 2831)** | **12.9 (4.4 , 29.1)** | **-31.6 (-48.9 , -9)** | **3929 (2352 , 6118)** | **43 (24.4 , 71)** | **-42.2 (-62.8 , -12.4)** | **294 (165 , 490)** | **2.3 (1.3 , 3.9)** | **-52.4 (-70.6 , -24.8)** |
| **Belgium** | **1331 (449 , 3024)** | **11.3 (3.9 , 24.9)** | **-35.5 (-52.7 , -13.5)** | **4144 (2451 , 6685)** | **36.8 (20.9 , 62.1)** | **-45.5 (-65 , -13.4)** | **295 (165 , 490)** | **1.9 (1.1 , 3.2)** | **-56.8 (-74.4 , -27)** |
| **Cyprus** | **195 (70 , 467)** | **14.2 (5.2 , 33.2)** | **-47.9 (-60.6 , -33.2)** | **612 (367 , 1026)** | **47.8 (28 , 80.2)** | **-60.6 (-73.5 , -40.3)** | **40 (22 , 67)** | **2.7 (1.5 , 4.4)** | **-72.4 (-82.7 , -57.3)** |
| **Denmark** | **922 (325 , 2156)** | **15.4 (5.5 , 36.6)** | **-35.1 (-52 , -12.5)** | **2341 (1385 , 3786)** | **42.4 (23.9 , 71.5)** | **-46.3 (-65.1 , -12.5)** | **121 (67 , 198)** | **1.6 (0.9 , 2.7)** | **-54.6 (-74.5 , -22.8)** |
| **Finland** | **776 (273 , 1776)** | **13.8 (4.8 , 31.5)** | **-39.3 (-56.5 , -15.5)** | **2498 (1480 , 3862)** | **45.2 (25.1 , 71.9)** | **-51.1 (-67.3 , -26.5)** | **200 (112 , 317)** | **2.9 (1.5 , 4.9)** | **-61.9 (-78.4 , -35.2)** |
| **France** | **6121 (2074 , 14447)** | **9.3 (3.2 , 21.7)** | **-38 (-55.6 , -14.7)** | **18183 (10602 , 29683)** | **29.9 (16.3 , 52.4)** | **-47.9 (-68.2 , -13.2)** | **968 (528 , 1647)** | **1.3 (0.7 , 2.3)** | **-60.8 (-78.7 , -32.5)** |
| **Germany** | **12505 (4408 , 29155)** | **13.5 (4.6 , 31.3)** | **-35.5 (-51.6 , -14.9)** | **39608 (23566 , 61544)** | **45.9 (26.4 , 73.8)** | **-46.1 (-64 , -21)** | **3110 (1769 , 5024)** | **2.5 (1.5 , 3.9)** | **-57.5 (-73.8 , -32.2)** |
| **Greece** | **1955 (679 , 4359)** | **16.6 (5.7 , 36.2)** | **-24.2 (-41.5 , 0.7)** | **6986 (4184 , 10606)** | **59.7 (33.9 , 95.7)** | **-32.7 (-54.7 , -2.4)** | **735 (426 , 1146)** | **4.2 (2.4 , 6.8)** | **-42.2 (-62.1 , -12.4)** |
| **Iceland** | **42 (15 , 95)** | **11 (3.8 , 24.4)** | **-26.3 (-46.3 , -1.6)** | **117 (70 , 182)** | **33.1 (18.7 , 54.5)** | **-36.5 (-59.9 , 0.9)** | **8 (4 , 12)** | **1.7 (0.9 , 2.7)** | **-47.1 (-68.7 , -10.3)** |
| **Ireland** | **625 (219 , 1430)** | **12.3 (4.3 , 29.1)** | **-43.5 (-58.4 , -26.2)** | **1885 (1130 , 2998)** | **37.8 (21.6 , 61)** | **-54.8 (-69.8 , -32.7)** | **118 (66 , 195)** | **2 (1.1 , 3.3)** | **-66.6 (-79.5 , -44.9)** |
| **Israel** | **1723 (601 , 3878)** | **18 (6.3 , 39.9)** | **-33.8 (-49.1 , -15.5)** | **6628 (3788 , 10630)** | **69.6 (39.2 , 112.4)** | **-45.6 (-63.7 , -20.4)** | **432 (259 , 684)** | **4.3 (2.6 , 6.8)** | **-56.3 (-72.8 , -30.9)** |
| **Italy** | **9227 (3346 , 20329)** | **14.1 (5.1 , 32.5)** | **-33.5 (-48.6 , -12.4)** | **31095 (18703 , 48707)** | **49.2 (28.6 , 80.1)** | **-43.7 (-61.4 , -16)** | **2837 (1668 , 4594)** | **3 (1.7 , 4.8)** | **-55.4 (-70.3 , -31.7)** |
| **Luxembourg** | **82 (28 , 185)** | **12.5 (4.3 , 28.2)** | **-40.2 (-55.1 , -20.1)** | **256 (147 , 412)** | **40.9 (23.5 , 67.6)** | **-51.6 (-67.4 , -24.4)** | **18 (10 , 29)** | **2.2 (1.2 , 3.7)** | **-63.2 (-78 , -40.8)** |
| **Malta** | **75 (26 , 174)** | **15.8 (5.4 , 36.7)** | **-35.1 (-50.4 , -16.1)** | **242 (145 , 375)** | **54.5 (31.4 , 90.6)** | **-46.4 (-63.4 , -19.6)** | **19 (11 , 30)** | **3.1 (1.8 , 4.9)** | **-57.5 (-72.7 , -29.7)** |
| **Monaco** | **5 (2 , 11)** | **11.8 (3.9 , 27.6)** | **-24.3 (-45.9 , 0.9)** | **15 (9 , 24)** | **38.1 (21.3 , 63.1)** | **-31.9 (-56.2 , 4.9)** | **1 (1 , 2)** | **2 (1.1 , 3.3)** | **-40.4 (-64.2 , 2.1)** |
| **Netherlands** | **2062 (702 , 4879)** | **11.7 (4 , 27.6)** | **-30.6 (-51 , -2.8)** | **6055 (3553 , 9418)** | **34.1 (19.5 , 57.8)** | **-40.1 (-60.1 , -11)** | **419 (231 , 678)** | **1.8 (1 , 3.1)** | **-50.4 (-71 , -13.5)** |
| **Norway** | **806 (280 , 1843)** | **14.9 (5.1 , 35.2)** | **-32.9 (-49.1 , -12.2)** | **2279 (1333 , 3808)** | **45.2 (25.3 , 78.5)** | **-44.1 (-64.4 , -13.9)** | **132 (76 , 215)** | **2.1 (1.2 , 3.4)** | **-53.1 (-70.5 , -25.2)** |
| **Portugal** | **1639 (599 , 3603)** | **15.1 (5.4 , 34.1)** | **-44.9 (-58.1 , -27.5)** | **5496 (3121 , 8824)** | **53.8 (29.8 , 91.9)** | **-58.1 (-72.2 , -34.2)** | **419 (230 , 685)** | **2.9 (1.6 , 4.9)** | **-70.5 (-81.7 , -50.7)** |
| **San Marino** | **4 (2 , 10)** | **12.5 (4.3 , 27.8)** | **-26.7 (-45.5 , 0.3)** | **14 (8 , 22)** | **40.8 (23.3 , 66.7)** | **-34.2 (-56.4 , 2.6)** | **1 (1 , 2)** | **2.2 (1.2 , 3.6)** | **-44.4 (-67.4 , -8)** |
| **Spain** | **7941 (2807 , 17757)** | **17.7 (6.1 , 39.7)** | **-37.6 (-53.3 , -19.1)** | **26987 (15486 , 44210)** | **66.7 (36.3 , 113.1)** | **-50.7 (-68.4 , -22.2)** | **1580 (867 , 2619)** | **3.1 (1.7 , 5.2)** | **-64.9 (-80.1 , -40)** |
| **Sweden** | **1483 (538 , 3444)** | **13.5 (4.9 , 30.3)** | **-25 (-44.8 , -0.4)** | **4433 (2667 , 6902)** | **42.7 (24.7 , 70.7)** | **-32.7 (-56.1 , 1.8)** | **313 (185 , 497)** | **2.3 (1.3 , 3.8)** | **-41.6 (-63.1 , -7.1)** |
| **Switzerland** | **1073 (365 , 2466)** | **11.7 (4 , 27.3)** | **-28 (-47.1 , -2)** | **3245 (1865 , 5124)** | **37.7 (21 , 62.2)** | **-36.5 (-60.6 , 0.5)** | **203 (109 , 343)** | **1.8 (1 , 3.2)** | **-45.7 (-69.6 , -4.2)** |
| **United Kingdom** | **13131 (4725 , 30377)** | **18 (6.5 , 41.2)** | **-25.1 (-38.9 , -8.4)** | **54333 (34376 , 82048)** | **84.6 (52.3 , 129.3)** | **-32.1 (-48.6 , -8.4)** | **7662 (4759 , 11374)** | **13.7 (8.1 , 21.4)** | **-36.3 (-58.3 , -7)** |
| **Southern Latin America** | **20804 (7524 , 45539)** | **31.2 (11.2 , 68.4)** | **-24.7 (-33.9 , -14.8)** | **99701 (63326 , 149365)** | **158.3 (98.5 , 240.9)** | **-42.2 (-53.5 , -29)** | **11291 (7490 , 16207)** | **16.3 (10.7 , 23.6)** | **-55.1 (-64.8 , -43.3)** |
| **Argentina** | **16578 (5966 , 37367)** | **37.1 (13.4 , 84.3)** | **-21.1 (-32.5 , -9)** | **84613 (52535 , 128479)** | **198 (123.1 , 303.4)** | **-41.3 (-54.1 , -26.1)** | **9299 (6023 , 13589)** | **20.2 (13 , 29.6)** | **-53.5 (-65.5 , -39)** |
| **Chile** | **3122 (1099 , 6911)** | **15.4 (5.5 , 34.5)** | **-45.1 (-55.7 , -32.2)** | **10048 (6089 , 15294)** | **50.3 (30.4 , 77)** | **-59.5 (-69.4 , -47.5)** | **1439 (854 , 2221)** | **6.6 (3.9 , 10.1)** | **-67.9 (-77.4 , -53.6)** |
| **Uruguay** | **1103 (386 , 2435)** | **32.4 (11.1 , 71)** | **-23.7 (-35.2 , -9.4)** | **5035 (3106 , 7719)** | **157.6 (96.2 , 249.4)** | **-38.7 (-54 , -19)** | **553 (346 , 838)** | **14.5 (8.9 , 22)** | **-50.7 (-64.7 , -31.2)** |
| **Eastern Europe** | **73544 (25979 , 164622)** | **29.6 (10.5 , 65.5)** | **-16.1 (-24.9 , -5.8)** | **362332 (230915 , 541520)** | **146.4 (93.7 , 218.7)** | **-31.5 (-42.2 , -19.4)** | **55695 (34625 , 81538)** | **20.6 (13.1 , 29.9)** | **-43.8 (-55.1 , -29.3)** |
| **Belarus** | **3303 (1157 , 7106)** | **30.6 (10.6 , 67.4)** | **-22.9 (-35.1 , -8.6)** | **14825 (9349 , 22604)** | **138.6 (85.9 , 209.8)** | **-41.1 (-54.5 , -24.4)** | **1921 (1182 , 2915)** | **16 (9.9 , 24.7)** | **-55.3 (-68.1 , -36.1)** |
| **Estonia** | **442 (159 , 998)** | **28.9 (10.5 , 65)** | **-26.1 (-38.4 , -11.5)** | **1874 (1191 , 2847)** | **122.3 (77.5 , 184.2)** | **-46.1 (-58.8 , -29.4)** | **241 (152 , 364)** | **13.8 (8.6 , 20.7)** | **-59.6 (-71.1 , -44.2)** |
| **Latvia** | **731 (265 , 1659)** | **32.9 (11.7 , 73.8)** | **-16.4 (-29.7 , -1.1)** | **3457 (2135 , 5107)** | **155 (95.4 , 229.3)** | **-33.1 (-48.3 , -13.2)** | **481 (291 , 710)** | **18.9 (11.3 , 28.4)** | **-46.2 (-61 , -25.3)** |
| **Lithuania** | **1075 (380 , 2375)** | **33.1 (11.6 , 73.4)** | **-12.6 (-25.5 , 1.7)** | **5124 (3185 , 7715)** | **157.9 (94.4 , 238.6)** | **-25.8 (-41.1 , -6.2)** | **716 (448 , 1088)** | **19.3 (11.8 , 29.4)** | **-38.6 (-54.7 , -16.6)** |
| **Republic of Moldova** | **1746 (620 , 3877)** | **42.9 (15.4 , 94.3)** | **-17.4 (-27.1 , -6.4)** | **10551 (6774 , 15887)** | **266.2 (168.9 , 407.4)** | **-36.2 (-47.5 , -22.9)** | **1525 (966 , 2268)** | **33.9 (22 , 49.7)** | **-47.5 (-60.1 , -30.6)** |
| **Russian Federation** | **53561 (18628 , 120477)** | **30.7 (10.7 , 67.4)** | **-15.6 (-27.5 , -1.1)** | **275398 (174291 , 413363)** | **157.3 (99.6 , 236.1)** | **-31.6 (-45.3 , -15.7)** | **42636 (25495 , 64117)** | **22.6 (13.6 , 33.9)** | **-44.6 (-59.1 , -25.9)** |
| **Ukraine** | **12686 (4461 , 29129)** | **24.7 (8.6 , 55.6)** | **-16.3 (-30.2 , 1.3)** | **51103 (32164 , 77043)** | **103.6 (65.4 , 161)** | **-29.2 (-45.3 , -6.3)** | **8175 (5084 , 12217)** | **14.5 (9.2 , 21.5)** | **-39.1 (-55.4 , -18.2)** |
| **Central Europe** | **37085 (12982 , 80343)** | **33.7 (11.9 , 72.7)** | **-20.8 (-26.4 , -14.7)** | **173612 (112779 , 257409)** | **173.1 (111.1 , 256.4)** | **-38 (-44.1 , -30.7)** | **22231 (14821 , 31739)** | **19.9 (13.2 , 28.5)** | **-53.6 (-59.5 , -46.5)** |
| **Albania** | **1180 (413 , 2577)** | **43.4 (15.3 , 95.9)** | **-24.4 (-32.8 , -14.5)** | **6491 (4237 , 9522)** | **260 (169.4 , 384.9)** | **-38.9 (-50 , -25.8)** | **772 (497 , 1122)** | **28 (18 , 41)** | **-57.7 (-68.2 , -43.7)** |
| **Bosnia and Herzegovina** | **1248 (436 , 2811)** | **38.3 (13.4 , 85.6)** | **-17.6 (-30.6 , -3.2)** | **6212 (3968 , 9445)** | **209.2 (131.6 , 318.8)** | **-35.6 (-49.4 , -17.9)** | **859 (536 , 1306)** | **25.7 (16.4 , 39.3)** | **-51.5 (-64.1 , -33.4)** |
| **Bulgaria** | **2552 (917 , 5631)** | **38.6 (13.5 , 85.3)** | **-8.3 (-23.4 , 9.3)** | **12684 (8029 , 19404)** | **210.3 (131.1 , 325)** | **-18.6 (-37.5 , 3.5)** | **1762 (1082 , 2676)** | **25.8 (16 , 38.7)** | **-30.4 (-50.2 , -2.8)** |
| **Croatia** | **1129 (395 , 2504)** | **27.1 (9.4 , 60.8)** | **-18.3 (-32.9 , 1.8)** | **4336 (2668 , 6683)** | **110.3 (67.1 , 165.6)** | **-32 (-49.2 , -7.1)** | **491 (286 , 775)** | **10.8 (6.4 , 16.6)** | **-43.5 (-63 , -16.6)** |
| **Czechia** | **2939 (1020 , 6450)** | **28.8 (10.1 , 64)** | **-22.5 (-36.7 , -4.8)** | **12067 (7254 , 17852)** | **127.7 (78.5 , 191.4)** | **-38.5 (-53.4 , -17.7)** | **1364 (824 , 2129)** | **12.9 (7.7 , 19.8)** | **-52.8 (-67.8 , -33.6)** |
| **Hungary** | **2504 (886 , 5642)** | **28.3 (10.2 , 65.2)** | **-19.1 (-34.8 , -2.3)** | **11299 (6813 , 17679)** | **142.4 (85.8 , 223.3)** | **-35.9 (-52.6 , -12.3)** | **1362 (809 , 2112)** | **15.2 (8.8 , 23.5)** | **-50 (-65.6 , -28.4)** |
| **Montenegro** | **208 (73 , 472)** | **33.9 (11.8 , 76)** | **-9.6 (-24.9 , 8.6)** | **951 (590 , 1442)** | **166.3 (101.2 , 255.6)** | **-19.6 (-37.9 , 5.7)** | **116 (70 , 175)** | **18.5 (11.6 , 27.9)** | **-31.1 (-52.2 , -2.4)** |
| **North Macedonia** | **742 (258 , 1657)** | **34.1 (11.9 , 74.1)** | **-17.3 (-29.9 , -2.3)** | **3512 (2187 , 5222)** | **192.3 (120.7 , 291.2)** | **-34 (-46.3 , -15.7)** | **540 (336 , 782)** | **29.2 (17.8 , 42.4)** | **-49.6 (-63.8 , -29.8)** |
| **Poland** | **12787 (4554 , 28231)** | **34.1 (11.9 , 75.7)** | **-23.5 (-35.7 , -9.6)** | **60074 (36969 , 93648)** | **175.7 (105.7 , 276.4)** | **-42.2 (-54.2 , -25.8)** | **7910 (4745 , 12385)** | **20.9 (12.7 , 32)** | **-58.5 (-69.8 , -43.8)** |
| **Romania** | **6567 (2345 , 14358)** | **36.2 (13.1 , 78.5)** | **-19.5 (-32.4 , -5.7)** | **32334 (20307 , 48352)** | **197.6 (122.8 , 298.7)** | **-36 (-49.9 , -17.6)** | **4134 (2582 , 6065)** | **22.4 (14 , 33.2)** | **-52.2 (-65 , -34.1)** |
| **Serbia** | **3012 (1043 , 6581)** | **35.8 (12.5 , 79)** | **-20.4 (-33.6 , -5.3)** | **14238 (8398 , 21872)** | **182.8 (108.1 , 285.9)** | **-37.8 (-52.7 , -21.5)** | **1827 (1100 , 2845)** | **21.2 (13.2 , 33.2)** | **-53.3 (-66.9 , -35.9)** |
| **Slovakia** | **1694 (604 , 3922)** | **31.5 (11.1 , 71.4)** | **-19.4 (-33.7 , -3.3)** | **7396 (4529 , 11420)** | **149.7 (92.2 , 231.4)** | **-34.6 (-50.1 , -13.7)** | **879 (540 , 1337)** | **16 (9.9 , 24.1)** | **-49 (-64.8 , -29.1)** |
| **Slovenia** | **524 (190 , 1211)** | **26.1 (9.3 , 61.2)** | **-24.6 (-38.1 , -7.1)** | **2018 (1250 , 3168)** | **109.6 (66.2 , 174.5)** | **-39.6 (-54.8 , -17.5)** | **216 (131 , 334)** | **10.3 (6.1 , 16.4)** | **-53.6 (-68.2 , -31.1)** |
| **Central Asia** | **51560 (18483 , 113555)** | **55.4 (19.9 , 122.2)** | **-5 (-8.9 , -1.1)** | **505211 (325590 , 741898)** | **537 (346.1 , 791.9)** | **-21.4 (-27.3 , -15.3)** | **83745 (55876 , 116480)** | **89.6 (60 , 124.4)** | **-37.2 (-44.1 , -29.5)** |
| **Armenia** | **1247 (442 , 2783)** | **39.3 (14.1 , 87)** | **-9 (-18.9 , 2.4)** | **8447 (5381 , 12534)** | **291.1 (182.6 , 433.1)** | **-20.5 (-33.9 , -4.6)** | **1655 (1058 , 2454)** | **54.7 (35.1 , 80.6)** | **-36.6 (-51.7 , -18.9)** |
| **Azerbaijan** | **5357 (1893 , 11953)** | **51.4 (18.2 , 114.2)** | **-7.6 (-15.7 , 1.4)** | **42385 (26851 , 63996)** | **410.2 (259.6 , 614.8)** | **-28.9 (-39 , -17.1)** | **6910 (4346 , 10123)** | **64.7 (40.9 , 93.8)** | **-43.4 (-55.6 , -28.7)** |
| **Georgia** | **1798 (638 , 3936)** | **45.7 (16.3 , 100)** | **-5.7 (-15.3 , 4.7)** | **14796 (9338 , 21909)** | **391.5 (248.7 , 576.1)** | **-14.4 (-28 , 1.4)** | **3167 (2086 , 4673)** | **77.4 (50.6 , 113.9)** | **-15.6 (-33.7 , 7.9)** |
| **Kazakhstan** | **9508 (3342 , 20851)** | **51.2 (18 , 111.5)** | **-11.8 (-19.9 , -4.2)** | **88590 (56067 , 130783)** | **474.8 (299.3 , 703.8)** | **-29.7 (-39.4 , -18.7)** | **15400 (9856 , 22422)** | **81.8 (52.3 , 118.8)** | **-43.7 (-55.8 , -29.1)** |
| **Kyrgyzstan** | **3419 (1242 , 7534)** | **52.9 (19.1 , 116)** | **-4.3 (-12.9 , 5.5)** | **33768 (21595 , 51226)** | **509.1 (329.4 , 776.4)** | **-22 (-33.1 , -10)** | **5753 (3724 , 8342)** | **90.5 (59.2 , 129.7)** | **-42.6 (-53.6 , -27.9)** |
| **Mongolia** | **1475 (522 , 3250)** | **44.1 (15.7 , 96.8)** | **-14 (-21 , -6.7)** | **13737 (8766 , 20582)** | **409.6 (259.4 , 610.7)** | **-38.8 (-47.2 , -28.8)** | **3972 (2581 , 5648)** | **118.9 (78.4 , 170)** | **-54.2 (-62.3 , -44.2)** |
| **Tajikistan** | **4684 (1657 , 10441)** | **51.6 (18.2 , 114.1)** | **-3.9 (-12.4 , 5.3)** | **43578 (27894 , 63270)** | **465.1 (300.4 , 670.3)** | **-18 (-29 , -4.6)** | **8517 (5558 , 12066)** | **96.8 (64 , 136.5)** | **-29.3 (-43.4 , -12.3)** |
| **Turkmenistan** | **2393 (862 , 5269)** | **47.6 (17.1 , 105.4)** | **-10.2 (-18.7 , -0.6)** | **19789 (12604 , 29362)** | **388.5 (246.9 , 578.8)** | **-30.1 (-40.7 , -16.9)** | **3718 (2374 , 5339)** | **73.8 (47.1 , 105.2)** | **-43.3 (-55 , -28.1)** |
| **Uzbekistan** | **21679 (7847 , 48153)** | **65 (23.6 , 144.2)** | **-4.6 (-12.1 , 3)** | **240121 (151812 , 353806)** | **699.1 (442.5 , 1025.4)** | **-21.6 (-32.8 , -9.5)** | **34653 (22507 , 50156)** | **103.4 (67.7 , 149.6)** | **-36.3 (-50.3 , -18.8)** |
| **Central Latin America** | **62015 (21888 , 134238)** | **25.4 (9 , 54.9)** | **-25.9 (-29.1 , -22.3)** | **393841 (259513 , 576830)** | **164.2 (108.2 , 240.2)** | **-40.2 (-43.8 , -36.3)** | **62759 (42312 , 88174)** | **26.1 (17.6 , 36.5)** | **-53 (-56.4 , -49.6)** |
| **Colombia** | **11095 (4008 , 24455)** | **23 (8.2 , 50.8)** | **-43.4 (-51.9 , -33.5)** | **53200 (32832 , 78677)** | **112.9 (69.6 , 167.3)** | **-61.2 (-69.6 , -50.5)** | **7750 (4886 , 11441)** | **15.5 (9.7 , 22.9)** | **-68.4 (-76.8 , -57.6)** |
| **Costa Rica** | **1258 (453 , 2865)** | **26.7 (9.5 , 61.5)** | **-27.7 (-39 , -14.9)** | **6507 (3984 , 10088)** | **143 (86.7 , 220.3)** | **-39.3 (-53.5 , -19.5)** | **827 (509 , 1269)** | **17.2 (10.6 , 26.4)** | **-48.2 (-61.8 , -31)** |
| **El Salvador** | **1823 (648 , 4115)** | **29.8 (10.7 , 67.3)** | **-26.4 (-37.8 , -13.4)** | **11520 (6952 , 17773)** | **189 (114.2 , 289.8)** | **-38.1 (-53 , -20.6)** | **1502 (938 , 2243)** | **24.3 (15.2 , 36.3)** | **-58.9 (-68.6 , -46.4)** |
| **Guatemala** | **6777 (2361 , 15682)** | **39.3 (13.8 , 89.7)** | **-20.8 (-29.6 , -11)** | **54092 (34246 , 81366)** | **303.5 (192.9 , 454.1)** | **-36.9 (-47.7 , -25.2)** | **7591 (4836 , 11213)** | **47.6 (30.8 , 69.6)** | **-57.7 (-66.3 , -47)** |
| **Honduras** | **3734 (1316 , 8057)** | **39.7 (14 , 86)** | **-22.4 (-31.1 , -11.8)** | **26194 (16336 , 39482)** | **272.2 (171.9 , 407)** | **-36.8 (-48 , -22.3)** | **3378 (2163 , 4998)** | **41.2 (27 , 60.4)** | **-48.3 (-58.8 , -34.8)** |
| **Mexico** | **26302 (9389 , 57458)** | **21.8 (7.8 , 47.7)** | **-19.6 (-22.2 , -17)** | **183339 (120325 , 269264)** | **153.7 (100.9 , 225.8)** | **-35 (-38.4 , -31.6)** | **33106 (22615 , 46100)** | **27.7 (19 , 38.6)** | **-47.6 (-50.9 , -43.8)** |
| **Nicaragua** | **1571 (540 , 3577)** | **25.8 (8.9 , 57.7)** | **-39.7 (-49.2 , -29.1)** | **7354 (4386 , 11269)** | **123.3 (74.3 , 186.9)** | **-58.8 (-67.4 , -48)** | **1006 (624 , 1499)** | **19.3 (12.1 , 28.1)** | **-66.8 (-74.6 , -57.2)** |
| **Panama** | **1592 (567 , 3544)** | **38.3 (13.7 , 85.2)** | **-18.9 (-28.6 , -6.9)** | **10619 (6516 , 15811)** | **255.1 (156.2 , 377.9)** | **-34.6 (-47.6 , -19.7)** | **1359 (865 , 1976)** | **32.5 (20.8 , 47.3)** | **-51.4 (-64.1 , -34.3)** |
| **Venezuela (Bolivarian Republic of)** | **7863 (2769 , 17667)** | **28.1 (9.9 , 63.2)** | **-27.9 (-38 , -15.2)** | **41016 (25558 , 62885)** | **150.6 (93.5 , 233.7)** | **-42.7 (-54.9 , -27)** | **6240 (3884 , 9177)** | **22.5 (14 , 33)** | **-55.2 (-65.5 , -40.1)** |
| **Andean Latin America** | **23895 (8476 , 53147)** | **37.6 (13.4 , 83.7)** | **-23.9 (-29.5 , -17.5)** | **170690 (108456 , 253252)** | **269.5 (171.4 , 401.3)** | **-52.6 (-58 , -46.2)** | **25181 (17018 , 35492)** | **40.5 (27.3 , 57.1)** | **-71.8 (-75.8 , -67.1)** |
| **Bolivia (Plurinational State of)** | **7105 (2555 , 15982)** | **57.6 (20.6 , 129)** | **-10.5 (-19.1 , -1.6)** | **58956 (37499 , 87422)** | **463.6 (296.8 , 685.8)** | **-34.9 (-44.5 , -23.7)** | **10827 (7010 , 15812)** | **89.3 (58.2 , 130)** | **-59.1 (-67.5 , -49.4)** |
| **Ecuador** | **3725 (1321 , 8383)** | **21.8 (7.7 , 49.1)** | **-44.1 (-51 , -35.6)** | **22741 (14760 , 34441)** | **133.1 (86.6 , 201.2)** | **-65.5 (-72.3 , -57.6)** | **3713 (2377 , 5242)** | **22.6 (14.5 , 31.8)** | **-69 (-76.2 , -59.4)** |
| **Peru** | **13065 (4662 , 29009)** | **38.9 (13.9 , 86)** | **-22.3 (-31.1 , -11.6)** | **88993 (55529 , 133270)** | **267.6 (167.2 , 400.5)** | **-56.4 (-64.2 , -46.6)** | **10642 (6920 , 15386)** | **31.8 (20.8 , 46)** | **-79.5 (-83.7 , -73.6)** |
| **Caribbean** | **26532 (9332 , 58967)** | **56 (19.7 , 124.6)** | **-6.5 (-11.5 , -1.1)** | **227561 (147759 , 336949)** | **500.1 (324.7 , 739.2)** | **-6.6 (-14.3 , 1)** | **34157 (22973 , 48746)** | **73.4 (49.3 , 105)** | **-23.2 (-34.3 , -10.6)** |
| **Antigua and Barbuda** | **49 (18 , 109)** | **55.3 (19.7 , 120.6)** | **-10.6 (-21.7 , 1.5)** | **309 (194 , 464)** | **374.6 (233 , 559.1)** | **-25.2 (-39.5 , -8.2)** | **34 (21 , 51)** | **37.4 (22.9 , 56.3)** | **-38.6 (-55.5 , -16.6)** |
| **Barbados** | **133 (49 , 297)** | **42.7 (15.6 , 95.6)** | **-14.8 (-26.2 , -1.9)** | **668 (407 , 1043)** | **262 (157.9 , 410.6)** | **-25.2 (-41 , -6.4)** | **73 (42 , 117)** | **31.1 (16.9 , 53.3)** | **-52.1 (-71.2 , -18.4)** |
| **Belize** | **250 (88 , 545)** | **63.2 (22.3 , 137.7)** | **-5 (-14.7 , 6.5)** | **2048 (1265 , 3017)** | **503.6 (310.8 , 751.6)** | **-14.9 (-28.6 , 2.3)** | **253 (154 , 378)** | **63.1 (38.2 , 92.8)** | **-28.9 (-47.4 , -5.6)** |
| **Bermuda** | **24 (8 , 55)** | **35.8 (12.7 , 81.1)** | **-27.6 (-38.5 , -14.6)** | **101 (62 , 151)** | **172.2 (104.7 , 261.7)** | **-45.7 (-59.2 , -28.1)** | **9 (5 , 13)** | **12.7 (7.6 , 19.5)** | **-61.6 (-74.1 , -44.4)** |
| **Bahamas** | **210 (75 , 457)** | **55.9 (20.1 , 122)** | **-7.3 (-18.2 , 4.5)** | **1439 (917 , 2148)** | **398.7 (251.6 , 595.2)** | **-16.2 (-31.4 , 2.1)** | **172 (104 , 258)** | **44.4 (27.3 , 66.6)** | **-27.5 (-46.9 , -1)** |
| **Cuba** | **5435 (1963 , 12291)** | **45.9 (16.1 , 102)** | **-15.4 (-27.2 , -2.9)** | **27775 (16487 , 41917)** | **261.9 (153.2 , 404.3)** | **-28.2 (-45.2 , -9.4)** | **2646 (1592 , 4005)** | **22.2 (13.3 , 34)** | **-42.6 (-59.6 , -20.8)** |
| **Dominica** | **39 (14 , 87)** | **54.3 (19 , 122.2)** | **-5.3 (-16.5 , 7.8)** | **251 (159 , 377)** | **365.1 (231.5 , 547.1)** | **-13.6 (-28.3 , 4.6)** | **36 (23 , 53)** | **49.5 (31.7 , 73.4)** | **-16.7 (-38.1 , 9.9)** |
| **Dominican Republic** | **5569 (2013 , 12530)** | **51.6 (18.6 , 116.5)** | **-16.9 (-25.9 , -6.9)** | **36466 (22758 , 54413)** | **336.5 (209.7 , 502.8)** | **-37.3 (-47.6 , -25.4)** | **4811 (3060 , 7244)** | **44.4 (28.4 , 66.1)** | **-52.2 (-64.5 , -37.8)** |
| **Grenada** | **54 (19 , 120)** | **52.9 (18.8 , 117.4)** | **-8.9 (-17.8 , 1.7)** | **385 (243 , 575)** | **408.8 (257.6 , 617)** | **-25.8 (-38.3 , -11.1)** | **44 (28 , 66)** | **45.4 (28.6 , 67.4)** | **-42.5 (-56.2 , -23.6)** |
| **Guyana** | **518 (184 , 1137)** | **68.1 (24.3 , 148.4)** | **-13.7 (-20.4 , -6.6)** | **4939 (3059 , 7218)** | **656.1 (404.6 , 952.4)** | **-27.8 (-37.5 , -17)** | **834 (531 , 1241)** | **110.5 (70.3 , 164.9)** | **-46.4 (-57.3 , -33)** |
| **Haiti** | **8839 (3114 , 19565)** | **73 (25.8 , 160.8)** | **1.1 (-7.2 , 10.3)** | **118150 (76188 , 176393)** | **915.6 (594.7 , 1369.9)** | **-4.1 (-14.6 , 7.6)** | **21007 (13770 , 30836)** | **171.9 (113.5 , 249.6)** | **-34.8 (-48.2 , -17.1)** |
| **Jamaica** | **1601 (567 , 3517)** | **57.3 (20.5 , 126.4)** | **-6.4 (-18.3 , 4.8)** | **10602 (6667 , 15999)** | **397.1 (248.6 , 602.2)** | **-16.4 (-33.1 , 1.6)** | **1212 (753 , 1783)** | **43.1 (26.8 , 63.5)** | **-25.4 (-46.2 , 1.8)** |
| **Puerto Rico** | **1482 (520 , 3351)** | **40 (14.2 , 89)** | **-20.3 (-33.2 , -6.4)** | **6692 (4088 , 10118)** | **205.2 (121.7 , 319.2)** | **-36.4 (-51.6 , -15.6)** | **610 (378 , 927)** | **16.6 (10.2 , 25)** | **-50.6 (-65.6 , -28.9)** |
| **Saint Kitts and Nevis** | **31 (11 , 68)** | **51 (18 , 112.5)** | **-13.1 (-24.8 , -0.2)** | **181 (114 , 273)** | **319.8 (198.3 , 481.4)** | **-28.5 (-43.6 , -9.8)** | **19 (12 , 29)** | **30.9 (18.6 , 46.1)** | **-43.8 (-58.6 , -20.2)** |
| **Saint Lucia** | **104 (36 , 232)** | **58.3 (20.2 , 130.3)** | **-10.8 (-21 , 1)** | **690 (431 , 1046)** | **418 (260.7 , 640.5)** | **-27 (-40.5 , -11.5)** | **83 (52 , 126)** | **46.2 (28.9 , 70.1)** | **-43.9 (-58.6 , -24.2)** |
| **Saint Vincent and the Grenadines** | **71 (25 , 154)** | **61.4 (21.8 , 133.8)** | **-4.4 (-14.4 , 8.4)** | **517 (319 , 782)** | **474.1 (292.4 , 712.7)** | **-13 (-28.2 , 4.6)** | **69 (44 , 103)** | **59.7 (37.9 , 88.8)** | **-22.3 (-41.8 , 2.8)** |
| **Suriname** | **360 (128 , 791)** | **62.4 (22.3 , 136.6)** | **-5.9 (-15.7 , 4.8)** | **2909 (1824 , 4342)** | **512.8 (319.1 , 762.1)** | **-16.8 (-30.2 , -1.5)** | **398 (256 , 596)** | **68.1 (43.8 , 100.7)** | **-31.3 (-48.5 , -10.9)** |
| **Trinidad and Tobago** | **812 (286 , 1832)** | **57.3 (20.2 , 128.4)** | **-9.8 (-19.7 , 3.2)** | **5408 (3297 , 8211)** | **410.2 (248.7 , 627)** | **-22.6 (-37.3 , -5.1)** | **654 (419 , 995)** | **46 (29 , 70.2)** | **-37.4 (-55 , -15.5)** |
| **United States Virgin Islands** | **55 (20 , 122)** | **50.2 (17.8 , 112.9)** | **-11.3 (-22.6 , 1.5)** | **320 (201 , 481)** | **324.3 (203.1 , 489.7)** | **-23.1 (-38 , -2.4)** | **35 (22 , 53)** | **33 (20.3 , 50)** | **-36 (-54.5 , -12.5)** |
| **Tropical Latin America** | **95047 (33915 , 208564)** | **41.6 (14.9 , 91.2)** | **-19.8 (-28.3 , -10.3)** | **822311 (522294 , 1240843)** | **373.1 (238 , 564.4)** | **-32.7 (-42.6 , -21.1)** | **151857 (94413 , 230065)** | **68 (42.6 , 102.2)** | **-50.7 (-62.4 , -35.8)** |
| **Brazil** | **92287 (32765 , 203244)** | **41.7 (14.8 , 91.2)** | **-19.8 (-28.6 , -10.1)** | **801171 (508514 , 1212274)** | **375.1 (238.6 , 568.9)** | **-32.6 (-42.7 , -20.5)** | **148491 (92113 , 226153)** | **68.6 (42.6 , 103.7)** | **-50.7 (-62.7 , -35.4)** |
| **Paraguay** | **2760 (979 , 6254)** | **40.5 (14.5 , 91.6)** | **-20.4 (-29.1 , -9.4)** | **21139 (12908 , 31891)** | **308.5 (188.5 , 464.7)** | **-32.5 (-43.9 , -18.6)** | **3366 (2074 , 5192)** | **49.9 (31.4 , 76)** | **-46.1 (-59.4 , -29.3)** |
| **East Asia** | **361018 (130227 , 797828)** | **22 (8 , 48.4)** | **-51 (-53.5 , -48.3)** | **1762936 (1124847 , 2608646)** | **116.1 (73.4 , 172.9)** | **-70.3 (-73.8 , -66.6)** | **298013 (191716 , 431295)** | **18.7 (11.9 , 27)** | **-81.3 (-85 , -76.6)** |
| **China** | **342355 (123620 , 757579)** | **21.5 (7.8 , 47.4)** | **-52.1 (-54.7 , -49.3)** | **1639006 (1046179 , 2425045)** | **111 (70.8 , 165.1)** | **-71.7 (-75.3 , -67.8)** | **276861 (177207 , 402919)** | **18 (11.5 , 26)** | **-82.3 (-86 , -77.7)** |
| **Democratic People's Republic of Korea** | **12207 (4238 , 27020)** | **45.4 (16 , 101.4)** | **-9.9 (-19 , 0.5)** | **94648 (59030 , 141454)** | **369.2 (229 , 551.9)** | **-20.9 (-34 , -5.1)** | **17232 (10499 , 26065)** | **61.5 (37.6 , 92.4)** | **-21 (-41 , 2.6)** |
| **Taiwan (Province of China)** | **6456 (2324 , 14742)** | **24 (8.6 , 52.7)** | **-33.2 (-43.4 , -20.9)** | **29282 (17902 , 45777)** | **122.3 (74.8 , 191)** | **-49.7 (-61.7 , -34.9)** | **3921 (2467 , 5988)** | **15 (9.2 , 23.1)** | **-62.9 (-73.1 , -48.1)** |
| **Southeast Asia** | **316539 (111591 , 697335)** | **47.8 (16.9 , 105.2)** | **-21.9 (-25.4 , -18.4)** | **2292062 (1498764 , 3375266)** | **356.2 (233.4 , 523.4)** | **-43.7 (-47.7 , -39.6)** | **324616 (214211 , 458078)** | **50 (33.1 , 70.4)** | **-60 (-64.1 , -55.3)** |
| **Cambodia** | **10813 (3882 , 24137)** | **66.3 (23.6 , 147.3)** | **-6.5 (-14.4 , 2.5)** | **109440 (71035 , 159221)** | **664.4 (432.9 , 966.7)** | **-33.2 (-42.3 , -22.3)** | **11581 (7585 , 17034)** | **74.5 (49.1 , 108.3)** | **-65.6 (-72.8 , -56.3)** |
| **Indonesia** | **128869 (45122 , 287449)** | **50.6 (17.7 , 112.9)** | **-22.2 (-29.1 , -15.2)** | **948467 (612666 , 1401478)** | **383.3 (246.3 , 568.2)** | **-45.6 (-52.9 , -37.7)** | **150355 (97840 , 215129)** | **62.2 (40.8 , 88.7)** | **-56.4 (-64.1 , -47.3)** |
| **Lao People's Democratic Republic** | **4203 (1465 , 9440)** | **61.1 (21.3 , 136.5)** | **-8.1 (-15.5 , 0.6)** | **37697 (24185 , 55689)** | **545.2 (350.2 , 801.8)** | **-33.3 (-41.7 , -22.8)** | **5352 (3473 , 7696)** | **83.9 (54.5 , 119.5)** | **-58.2 (-66.1 , -48.1)** |
| **Malaysia** | **14132 (5001 , 32500)** | **45.9 (16.2 , 105.8)** | **-6.4 (-16.1 , 3.7)** | **101292 (64649 , 152688)** | **336.1 (213.4 , 505.8)** | **-39.5 (-49 , -28.9)** | **19814 (12592 , 29498)** | **63.9 (40.2 , 94.9)** | **-61.6 (-69.2 , -52.2)** |
| **Maldives** | **249 (87 , 548)** | **53 (18.7 , 117.3)** | **-25.1 (-34 , -15.7)** | **1774 (1111 , 2707)** | **385.7 (239 , 581.9)** | **-62.8 (-69.6 , -55.5)** | **144 (88 , 222)** | **31 (19.3 , 47.9)** | **-85.7 (-89.5 , -81.1)** |
| **Mauritius** | **651 (239 , 1475)** | **49.8 (18.3 , 114.2)** | **-12.7 (-22.8 , -1.8)** | **4077 (2638 , 6082)** | **365.7 (229.5 , 551.6)** | **-33.4 (-45.6 , -19.2)** | **492 (314 , 724)** | **41.8 (26.3 , 62.1)** | **-50.5 (-63.7 , -35)** |
| **Myanmar** | **35795 (12806 , 79734)** | **66.6 (23.9 , 147.4)** | **-3 (-10.8 , 5.4)** | **387960 (247189 , 582901)** | **730.2 (464.3 , 1097.9)** | **-20.1 (-29.9 , -9.3)** | **50563 (31878 , 74365)** | **95.8 (61.8 , 140.3)** | **-53 (-62.5 , -41.3)** |
| **Philippines** | **44477 (15896 , 97946)** | **41.3 (14.7 , 91.1)** | **-27 (-34.5 , -19.3)** | **312424 (197330 , 477771)** | **287.4 (182.9 , 438.3)** | **-44.1 (-53.3 , -33.7)** | **44320 (27653 , 65348)** | **43.2 (27.1 , 62.7)** | **-55.8 (-65.3 , -43.4)** |
| **Sri Lanka** | **10671 (3698 , 23870)** | **47.4 (16.5 , 105.5)** | **-25.5 (-34.4 , -16.8)** | **62852 (38822 , 97602)** | **285.7 (175.4 , 438)** | **-56.8 (-65.1 , -47.2)** | **6153 (3769 , 9532)** | **27.2 (16.9 , 42)** | **-76.1 (-82.3 , -68.2)** |
| **Seychelles** | **48 (17 , 106)** | **46.6 (16.8 , 102.8)** | **-18.9 (-29 , -7.9)** | **280 (175 , 428)** | **289.3 (177.5 , 441.6)** | **-41.2 (-52.7 , -27.1)** | **31 (19 , 46)** | **30.1 (18.9 , 44.4)** | **-59.6 (-69.4 , -46.8)** |
| **Thailand** | **31384 (11165 , 69963)** | **41 (14.6 , 91.9)** | **-28.4 (-38.2 , -17.9)** | **129871 (80051 , 192643)** | **187.1 (116.5 , 283.7)** | **-51.5 (-61.7 , -38.7)** | **11725 (7363 , 17652)** | **15 (9.2 , 22.7)** | **-68.5 (-77.4 , -58)** |
| **Timor-Leste** | **760 (271 , 1655)** | **57.3 (20.6 , 127.7)** | **-12.8 (-21.2 , -2.7)** | **6783 (4277 , 10055)** | **475.8 (301.6 , 706)** | **-34.3 (-44.1 , -22.3)** | **643 (410 , 923)** | **53.1 (34.5 , 74.9)** | **-60.7 (-68.8 , -50)** |
| **Viet Nam** | **34071 (12102 , 78101)** | **35.6 (12.7 , 82)** | **-34.9 (-42.8 , -25.4)** | **186143 (115553 , 284774)** | **207.5 (130.6 , 317.6)** | **-60.3 (-67.3 , -51.5)** | **23018 (14772 , 34672)** | **25.6 (16.5 , 38.3)** | **-73.3 (-80.1 , -63.8)** |
| **Oceania** | **7858 (2722 , 17059)** | **61.9 (21.4 , 133.9)** | **-1.6 (-8.1 , 5.2)** | **93908 (60031 , 138394)** | **681 (436.6 , 996.9)** | **-10.7 (-18.7 , -2.3)** | **21643 (14236 , 31434)** | **164.3 (108.5 , 235.9)** | **-24.6 (-37.8 , -8.7)** |
| **American Samoa** | **29 (10 , 64)** | **54.6 (19.3 , 122.5)** | **-3.5 (-13.5 , 8.4)** | **205 (134 , 303)** | **390.9 (253.1 , 578.3)** | **-13.8 (-27.6 , 1.7)** | **31 (20 , 47)** | **59.5 (39.5 , 89.9)** | **-24.8 (-42.3 , -2.1)** |
| **Cook Islands** | **9 (3 , 21)** | **50.3 (18.1 , 112.3)** | **-9.5 (-20.8 , 4.5)** | **55 (34 , 85)** | **308.2 (191.6 , 480.1)** | **-25.7 (-40.4 , -7.2)** | **6 (4 , 9)** | **32.3 (20.1 , 49.5)** | **-40.8 (-56.9 , -18.3)** |
| **Micronesia (Federated States of)** | **60 (21 , 131)** | **62.4 (22.2 , 138.7)** | **-5.2 (-13.4 , 4.9)** | **541 (346 , 801)** | **559.2 (359.9 , 822.7)** | **-24.5 (-34.9 , -13.5)** | **91 (59 , 133)** | **97 (64 , 141.8)** | **-42.8 (-54.4 , -27.4)** |
| **Fiji** | **682 (242 , 1512)** | **76.4 (27.1 , 168.9)** | **12.4 (3.1 , 22.6)** | **5306 (3368 , 7895)** | **594.9 (377.8 , 884.2)** | **2.1 (-11.2 , 18.4)** | **744 (479 , 1053)** | **86.4 (56.2 , 121.5)** | **-9.3 (-28.2 , 14.6)** |
| **Guam** | **93 (33 , 208)** | **53.5 (19.3 , 120.7)** | **-0.3 (-13.1 , 13.9)** | **612 (377 , 910)** | **359.7 (219.8 , 536.3)** | **-8.4 (-25.5 , 11.7)** | **75 (46 , 111)** | **43.4 (26.8 , 65.4)** | **-14.1 (-37 , 13.5)** |
| **Kiribati** | **75 (26 , 162)** | **67.1 (23.7 , 146.1)** | **0.5 (-7.7 , 10.1)** | **856 (551 , 1285)** | **731.7 (474.7 , 1093.8)** | **-9.2 (-19.7 , 3.5)** | **185 (115 , 277)** | **168.2 (106.5 , 251.2)** | **-22.8 (-38.5 , -1.2)** |
| **Marshall Islands** | **34 (12 , 75)** | **63.8 (22.6 , 144.7)** | **-0.7 (-10.2 , 9.7)** | **323 (208 , 477)** | **579.3 (371.7 , 849.1)** | **-16.3 (-27.1 , -4.1)** | **57 (37 , 84)** | **106.6 (69.1 , 155.1)** | **-29 (-43.9 , -10.1)** |
| **Nauru** | **6 (2 , 13)** | **60.1 (21.5 , 138.4)** | **-2.7 (-12.6 , 8.5)** | **49 (31 , 75)** | **474.6 (300.5 , 715.3)** | **-14.3 (-27.6 , 1)** | **7 (4 , 10)** | **68.6 (43.5 , 102.2)** | **-27.7 (-45 , -5.6)** |
| **Niue** | **1 (0 , 2)** | **53.1 (18.7 , 114.7)** | **-9.4 (-19.6 , 2.6)** | **6 (4 , 9)** | **352.9 (220.6 , 531.9)** | **-29.9 (-42.1 , -15.4)** | **1 (0 , 1)** | **41.5 (25.4 , 61.7)** | **-46.8 (-59.3 , -29)** |
| **Northern Mariana Islands** | **22 (8 , 49)** | **50.6 (17.8 , 109.2)** | **-6.5 (-18.6 , 7.3)** | **122 (75 , 185)** | **308.4 (186.4 , 462.2)** | **-18.2 (-34.6 , 2.3)** | **14 (9 , 22)** | **33.6 (20.7 , 50)** | **-29.1 (-48.2 , -3.5)** |
| **Palau** | **10 (3 , 21)** | **52.5 (18.5 , 114.6)** | **-8.2 (-18.8 , 4.4)** | **57 (36 , 84)** | **335.1 (211.1 , 504.3)** | **-26 (-39.7 , -9.1)** | **7 (4 , 11)** | **39.3 (25 , 60)** | **-38.1 (-53.5 , -18)** |
| **Papua New Guinea** | **5729 (1998 , 12407)** | **60 (20.8 , 129.5)** | **-3.8 (-12.1 , 5.3)** | **73790 (46916 , 109246)** | **710.9 (454.6 , 1044.9)** | **-15.6 (-25.1 , -5.7)** | **18077 (11873 , 26429)** | **184.9 (120.5 , 265.9)** | **-31.2 (-45.1 , -14.1)** |
| **Samoa** | **92 (33 , 205)** | **47.5 (16.7 , 108.3)** | **-5.4 (-16.7 , 6.6)** | **741 (462 , 1125)** | **356.6 (225.7 , 536.4)** | **-16.7 (-31.9 , 1.8)** | **103 (66 , 156)** | **52.1 (33.4 , 78.1)** | **-28.7 (-45.8 , -4.5)** |
| **Solomon Islands** | **406 (144 , 878)** | **66.8 (23.8 , 145.6)** | **-1.3 (-9.8 , 8.6)** | **4388 (2758 , 6504)** | **643.8 (412.9 , 946.5)** | **-14.1 (-24.9 , 0)** | **750 (467 , 1124)** | **117.2 (74.2 , 173.5)** | **-32.1 (-46.7 , -11.1)** |
| **Tokelau** | **1 (0 , 2)** | **59 (20.7 , 129.1)** | **-7.7 (-17.7 , 3.1)** | **6 (4 , 9)** | **440 (285.1 , 653)** | **-32.2 (-43.2 , -18.9)** | **1 (1 , 1)** | **60.8 (39.8 , 88)** | **-52.3 (-63.7 , -37.5)** |
| **Tonga** | **56 (20 , 123)** | **58.1 (21 , 128.6)** | **-3.4 (-13.1 , 8)** | **451 (282 , 687)** | **455.1 (287.3 , 687.1)** | **-16.1 (-29.7 , 0)** | **70 (46 , 104)** | **73.7 (48.1 , 108.8)** | **-28.6 (-45.7 , -6.1)** |
| **Tuvalu** | **7 (3 , 16)** | **63 (22.5 , 137.5)** | **-3.6 (-13.7 , 6.3)** | **61 (39 , 92)** | **533.3 (345.2 , 802.1)** | **-26.5 (-36.9 , -14.9)** | **10 (6 , 14)** | **87 (55.7 , 127.3)** | **-44.9 (-57.6 , -29.3)** |
| **Vanuatu** | **177 (62 , 383)** | **66.3 (23.3 , 143.1)** | **1.1 (-8.3 , 10.9)** | **1903 (1230 , 2879)** | **659.9 (428 , 992.4)** | **-6.9 (-18 , 5.5)** | **394 (257 , 568)** | **143.4 (93 , 206.7)** | **-19.1 (-35.9 , 3.8)** |
| **North Africa and Middle East** | **241762 (86245 , 531562)** | **40.7 (14.6 , 89.3)** | **-23.7 (-26.9 , -20.5)** | **2030272 (1321451 , 3009169)** | **337 (219.3 , 499.9)** | **-35.9 (-39.7 , -32)** | **341534 (227820 , 481251)** | **57.1 (38.1 , 80)** | **-44.9 (-49.9 , -39.4)** |
| **Afghanistan** | **13175 (4707 , 28924)** | **36.1 (12.9 , 79.5)** | **-16.5 (-25 , -7.7)** | **169071 (105545 , 259360)** | **412.1 (263.5 , 610.5)** | **-25.6 (-36.1 , -13)** | **34731 (22091 , 51374)** | **95.8 (61.3 , 136.2)** | **-36.8 (-50.1 , -19.9)** |
| **Algeria** | **16992 (6119 , 37187)** | **40.8 (14.7 , 89.5)** | **-22.2 (-31.6 , -11.5)** | **120843 (75608 , 186536)** | **289.3 (181.9 , 446.7)** | **-40.2 (-52.4 , -24.5)** | **12710 (7768 , 19098)** | **31 (19.1 , 45.8)** | **-58.9 (-70.3 , -44.8)** |
| **Bahrain** | **628 (223 , 1391)** | **44.3 (15.7 , 98.2)** | **-25.7 (-35.7 , -14.7)** | **3500 (2158 , 5373)** | **273.3 (169.6 , 423.1)** | **-46.4 (-58.3 , -31.1)** | **250 (152 , 388)** | **19.6 (12.1 , 30.7)** | **-66.6 (-76.7 , -53.2)** |
| **Egypt** | **47055 (16361 , 105881)** | **48.4 (17 , 107.8)** | **-19 (-28.6 , -8.2)** | **322540 (200355 , 493975)** | **317.9 (199.8 , 488)** | **-46 (-56.5 , -34.6)** | **29805 (18383 , 46190)** | **31.9 (20.2 , 47.8)** | **-68.7 (-77.1 , -58.1)** |
| **Iran (Islamic Republic of)** | **25589 (9208 , 56720)** | **31.1 (11.2 , 67.6)** | **-37.6 (-46.5 , -28.4)** | **133523 (83327 , 204894)** | **169.6 (107.7 , 257.9)** | **-56.1 (-65 , -45.4)** | **15516 (9484 , 23699)** | **19.8 (12.3 , 29.9)** | **-68.2 (-76.3 , -58.4)** |
| **Iraq** | **16696 (5971 , 38012)** | **41.7 (14.7 , 94.2)** | **-20.1 (-29.6 , -9.7)** | **123409 (73946 , 186990)** | **300.1 (184.4 , 453.4)** | **-37.4 (-48.7 , -23.7)** | **13388 (8142 , 20038)** | **35.2 (22.2 , 51.8)** | **-57.4 (-68 , -43)** |
| **Jordan** | **4388 (1578 , 9614)** | **39.3 (14.2 , 86.8)** | **-21.4 (-30.7 , -11.3)** | **36044 (22822 , 53289)** | **311.3 (198 , 461)** | **-38.1 (-47.8 , -26.4)** | **4680 (2932 , 6962)** | **41.7 (26.1 , 61.2)** | **-59.7 (-69.1 , -47.3)** |
| **Kuwait** | **1439 (498 , 3188)** | **32.9 (11.5 , 73.9)** | **-19.5 (-30.1 , -7)** | **9629 (5922 , 14377)** | **224.3 (138.8 , 336.5)** | **-33.2 (-46.1 , -16.7)** | **1141 (687 , 1713)** | **25.2 (15.8 , 37.3)** | **-52.2 (-64.6 , -36.2)** |
| **Lebanon** | **1531 (522 , 3578)** | **29.4 (10.1 , 68.5)** | **-43.1 (-51.9 , -32.4)** | **7347 (4402 , 11704)** | **142.7 (84.4 , 228.4)** | **-63 (-72 , -50)** | **516 (314 , 789)** | **9.9 (6 , 14.9)** | **-77.7 (-84.1 , -69.3)** |
| **Libya** | **2868 (988 , 6261)** | **44 (15.5 , 96.3)** | **-14.6 (-25.5 , -2.7)** | **20503 (12698 , 30916)** | **327.1 (202.9 , 501.3)** | **-27.5 (-41.4 , -9.9)** | **2367 (1445 , 3568)** | **37.3 (23.1 , 56.4)** | **-44.9 (-59.8 , -24.8)** |
| **Morocco** | **16772 (5930 , 37303)** | **47.3 (16.8 , 105.6)** | **-17.1 (-25.9 , -7.1)** | **138372 (86071 , 206726)** | **397.3 (247.7 , 595.3)** | **-35.7 (-46.7 , -22.7)** | **18043 (11193 , 26916)** | **52.3 (32.5 , 77.5)** | **-57 (-67.5 , -43.2)** |
| **Palestine** | **2014 (731 , 4531)** | **42.4 (15.3 , 96.5)** | **-18.4 (-27.8 , -7)** | **13745 (8459 , 20908)** | **277.1 (171.5 , 417.4)** | **-40.4 (-51.6 , -26.7)** | **1186 (740 , 1741)** | **27.7 (17.4 , 40.1)** | **-60.1 (-69.9 , -47.4)** |
| **Oman** | **2301 (772 , 5158)** | **56.6 (19.6 , 126.3)** | **-24 (-34.5 , -13.7)** | **12240 (7332 , 18602)** | **346 (210.3 , 527.9)** | **-53.3 (-62.7 , -41.5)** | **812 (496 , 1216)** | **28.6 (17.9 , 42.3)** | **-69.1 (-76.9 , -59.3)** |
| **Qatar** | **713 (250 , 1681)** | **28.4 (10 , 66.4)** | **-42.9 (-53.4 , -31.3)** | **2831 (1636 , 4392)** | **119.4 (68.7 , 191.3)** | **-62.8 (-71.9 , -51.4)** | **238 (136 , 389)** | **9.3 (5.6 , 14.4)** | **-79.3 (-85 , -71.8)** |
| **Saudi Arabia** | **8228 (2821 , 18621)** | **25.7 (9.1 , 58.1)** | **-35.2 (-45.9 , -23.6)** | **36877 (22132 , 56794)** | **127.4 (77.4 , 198.2)** | **-51.2 (-62.2 , -38)** | **3633 (2169 , 5660)** | **12.8 (7.9 , 19.7)** | **-70.6 (-78.4 , -61)** |
| **Sudan** | **21431 (7805 , 47186)** | **54.2 (19.9 , 119.7)** | **-10.2 (-18.3 , -0.9)** | **250549 (158953 , 375265)** | **563.2 (358.3 , 854.1)** | **-27.1 (-36.9 , -15.7)** | **40519 (25629 , 61059)** | **94.3 (60.7 , 137)** | **-52.4 (-62.7 , -38.9)** |
| **Syrian Arab Republic** | **6211 (2238 , 13823)** | **44.8 (16 , 99.7)** | **-18.7 (-28.5 , -7.2)** | **44971 (27983 , 67973)** | **327.8 (203.4 , 495.2)** | **-37.9 (-50.9 , -23.5)** | **4723 (2979 , 7084)** | **34.7 (21.7 , 52.8)** | **-59.9 (-70.8 , -46.1)** |
| **Tunisia** | **3552 (1248 , 8221)** | **30.8 (10.7 , 71.5)** | **-29.4 (-39.6 , -18)** | **18686 (11307 , 28897)** | **168.4 (101.9 , 261.4)** | **-46 (-57.8 , -31.6)** | **1862 (1138 , 2925)** | **16.4 (10 , 25.9)** | **-62.6 (-72.9 , -47.8)** |
| **Turkey** | **30342 (10940 , 67779)** | **37.7 (13.7 , 85)** | **-31 (-40.1 , -21.1)** | **190396 (118439 , 295326)** | **249.9 (156 , 392.9)** | **-54.1 (-63.6 , -43.1)** | **18799 (11542 , 28605)** | **23.6 (14.4 , 36.5)** | **-74.4 (-81.9 , -65.1)** |
| **United Arab Emirates** | **3240 (1141 , 7575)** | **38.9 (13.3 , 82.6)** | **-21.4 (-33.5 , -7)** | **17504 (10504 , 26190)** | **241.6 (144.8 , 359.7)** | **-35.5 (-48.9 , -18.6)** | **2128 (1226 , 3357)** | **26.6 (16.4 , 39.6)** | **-53.1 (-65.8 , -36.5)** |
| **Yemen** | **16352 (5818 , 35983)** | **54.3 (19.6 , 119.2)** | **-3 (-11.2 , 6.4)** | **355629 (231312 , 522843)** | **1011 (657.2 , 1488.7)** | **13.4 (3.8 , 24.2)** | **134140 (87774 , 192149)** | **378.8 (250.6 , 535.7)** | **5.8 (-15.2 , 32.6)** |
| **South Asia** | **1290558 (460252 , 2834388)** | **73.2 (26.1 , 160.7)** | **-2.3 (-4 , -0.6)** | **17667653 (11643495 , 25849278)** | **1005.9 (664.2 , 1471.5)** | **-17 (-19 , -15.1)** | **4761358 (3264447 , 6675632)** | **279.1 (191.2 , 391)** | **-40.4 (-42.8 , -37.8)** |
| **Bangladesh** | **107952 (38953 , 232949)** | **69.8 (25.3 , 150.7)** | **-5.4 (-13.7 , 3.3)** | **961505 (606160 , 1443693)** | **633.5 (396.9 , 957.5)** | **-37.3 (-45.4 , -27.6)** | **146333 (95347 , 210509)** | **99.6 (65.3 , 142.7)** | **-63.6 (-70.8 , -54.6)** |
| **Bhutan** | **531 (186 , 1180)** | **72.3 (25.4 , 161.1)** | **2.8 (-5 , 12.2)** | **8592 (5559 , 12703)** | **1227.1 (794.1 , 1821.7)** | **-17.7 (-26.1 , -9.2)** | **1549 (977 , 2243)** | **225.1 (143.7 , 325.7)** | **-56.6 (-66.6 , -44.4)** |
| **India** | **1011106 (360072 , 2213134)** | **73.9 (26.4 , 162)** | **-2 (-3.6 , -0.6)** | **14255727 (9362122 , 20908785)** | **1059.9 (696.4 , 1555)** | **-16 (-17.6 , -14.4)** | **4041501 (2734663 , 5651851)** | **307.2 (207.1 , 428.6)** | **-39.9 (-42.4 , -37.3)** |
| **Nepal** | **20970 (7600 , 46597)** | **71.5 (25.7 , 159.6)** | **-4.9 (-12.3 , 4)** | **219493 (141677 , 326830)** | **752.4 (485.8 , 1125.5)** | **-25.2 (-34.1 , -14.5)** | **35544 (23102 , 51477)** | **130.7 (85.6 , 189)** | **-52.8 (-61.6 , -42.1)** |
| **Pakistan** | **150000 (53379 , 338918)** | **71.3 (25.4 , 160.4)** | **0.5 (-6.4 , 9)** | **2222336 (1414425 , 3348523)** | **961.5 (617.5 , 1434.6)** | **-11.2 (-19.9 , -0.7)** | **536430 (341998 , 793438)** | **252.9 (163.3 , 367.1)** | **-34.1 (-45.8 , -20.6)** |
| **Southern Sub-Saharan Africa** | **33345 (11884 , 74302)** | **43.5 (15.5 , 97.1)** | **-11.8 (-17.9 , -5.1)** | **338594 (223391 , 497829)** | **432.7 (285.9 , 633.3)** | **-18.1 (-26.4 , -9.1)** | **73722 (49810 , 103216)** | **95.9 (64.5 , 134)** | **-25 (-38.2 , -9.9)** |
| **Botswana** | **1107 (392 , 2444)** | **48.5 (17.1 , 106.8)** | **-14.2 (-22 , -5.5)** | **11573 (7257 , 17144)** | **487.3 (306.9 , 716.8)** | **-33.2 (-43.5 , -21.7)** | **1660 (1054 , 2442)** | **71.7 (45.8 , 104.4)** | **-51.9 (-63 , -37.1)** |
| **Lesotho** | **963 (345 , 2147)** | **48 (17.1 , 106.2)** | **-2.3 (-10.8 , 6.3)** | **11356 (7177 , 16680)** | **546.3 (347.9 , 796.8)** | **-7.5 (-19.8 , 6.8)** | **2358 (1523 , 3402)** | **117.4 (76.4 , 168)** | **-17.5 (-34 , 4.8)** |
| **Namibia** | **1099 (390 , 2403)** | **46.4 (16.5 , 101.5)** | **-16.5 (-23.6 , -8.6)** | **12110 (7627 , 17950)** | **476.3 (301.9 , 705.6)** | **-36.3 (-45.1 , -26.4)** | **1942 (1222 , 2749)** | **81.7 (52.8 , 115.3)** | **-58.6 (-67.8 , -47.2)** |
| **South Africa** | **21430 (7624 , 47718)** | **39.2 (13.9 , 87.1)** | **-15.1 (-23.3 , -5.7)** | **206368 (135048 , 302819)** | **373.3 (245 , 546.5)** | **-23.6 (-34.3 , -11.6)** | **53248 (35663 , 74805)** | **95.2 (63.7 , 133.6)** | **-28.8 (-44.9 , -8.4)** |
| **Eswatini** | **516 (183 , 1164)** | **47 (16.5 , 106.9)** | **-7.1 (-16.1 , 3.3)** | **5151 (3224 , 7559)** | **447.9 (285.1 , 657.5)** | **-17.1 (-30 , -1.1)** | **822 (529 , 1209)** | **79.5 (51.4 , 115)** | **-27.1 (-43.4 , -5.4)** |
| **Zimbabwe** | **8228 (2916 , 17989)** | **55.9 (19.8 , 122.8)** | **-5.5 (-13.1 , 3)** | **92036 (58185 , 136488)** | **587.5 (375.7 , 869)** | **0.3 (-14.7 , 18.2)** | **13692 (8664 , 19512)** | **101.6 (66.2 , 145.3)** | **19.4 (-6.4 , 54.1)** |
| **Western Sub-Saharan Africa** | **317288 (113179 , 698870)** | **71.6 (25.4 , 156.7)** | **4.5 (0.5 , 8.4)** | **5619034 (3687439 , 8234679)** | **1013.7 (667 , 1489.8)** | **-1.1 (-5.4 , 3.5)** | **1336299 (906004 , 1885816)** | **232.5 (157.6 , 327.2)** | **-25.6 (-33.8 , -16)** |
| **Benin** | **8126 (2912 , 17900)** | **65.9 (24 , 145.6)** | **-0.5 (-8.1 , 7.4)** | **137903 (89628 , 202533)** | **884.5 (583.8 , 1301.1)** | **-2.2 (-12.9 , 9.4)** | **24215 (15315 , 36352)** | **154 (100.5 , 228.7)** | **-13.8 (-32 , 11.3)** |
| **Burkina Faso** | **15742 (5540 , 34598)** | **72.6 (25.6 , 160.4)** | **-1.7 (-9 , 6)** | **359064 (233033 , 531982)** | **1231.4 (799.5 , 1816.6)** | **-0.6 (-8.9 , 8.8)** | **98572 (64731 , 141077)** | **316 (208 , 450.1)** | **-9.4 (-29.9 , 16.3)** |
| **Cameroon** | **16960 (6113 , 36996)** | **60.6 (21.9 , 132.8)** | **6.1 (-2.2 , 15.8)** | **234859 (150162 , 344613)** | **700.5 (448.8 , 1038.2)** | **-1.8 (-13.9 , 11.7)** | **39432 (25288 , 57333)** | **121.8 (78.7 , 174.4)** | **-28.4 (-44.3 , -8)** |
| **Cabo Verde** | **314 (111 , 702)** | **56.4 (19.8 , 124.9)** | **-8 (-17.8 , 2.4)** | **2996 (1880 , 4356)** | **533.2 (332 , 779.3)** | **-27.2 (-40.1 , -13.7)** | **309 (189 , 457)** | **54.9 (33.9 , 81.6)** | **-52.9 (-66.1 , -34.5)** |
| **Chad** | **11126 (3994 , 24305)** | **69.1 (24.7 , 153.5)** | **3 (-4.9 , 11.4)** | **204005 (132529 , 303286)** | **949.2 (610.5 , 1394.8)** | **-3.3 (-13.9 , 8.4)** | **45463 (28693 , 69525)** | **212.5 (138.5 , 313.4)** | **-16.6 (-36.6 , 9.2)** |
| **CÃ´te d'Ivoire** | **19577 (6972 , 43100)** | **77 (27.2 , 170.1)** | **-4.3 (-10.3 , 1.9)** | **321238 (208652 , 469013)** | **1054.8 (681.7 , 1538.6)** | **-12.7 (-20.4 , -4.1)** | **62971 (40034 , 92018)** | **210.1 (135.3 , 303.6)** | **-31.1 (-45.6 , -13.6)** |
| **Gambia** | **1616 (567 , 3488)** | **72.4 (25.5 , 157.3)** | **4.2 (-3.5 , 12.7)** | **30221 (19495 , 44259)** | **1141.6 (735.6 , 1673.5)** | **-6.6 (-15.4 , 2.1)** | **6357 (4044 , 9163)** | **248 (158.5 , 351.5)** | **-32.1 (-47.3 , -13.6)** |
| **Ghana** | **22388 (7969 , 50640)** | **72.6 (26 , 163.2)** | **2.9 (-6.6 , 13.5)** | **307035 (197498 , 444798)** | **900.5 (582.8 , 1309.8)** | **-10.5 (-21.3 , 1.5)** | **53429 (35240 , 77061)** | **158.5 (103.6 , 225.4)** | **-40.8 (-54.1 , -21.6)** |
| **Guinea** | **8565 (2972 , 18856)** | **70.7 (24.7 , 157.4)** | **3.6 (-3.4 , 11.4)** | **150782 (98028 , 221615)** | **986 (646.1 , 1449)** | **-1.3 (-11.4 , 9.7)** | **34996 (22616 , 50873)** | **222.7 (146.7 , 322)** | **-21 (-36.8 , -1.7)** |
| **Guinea-Bissau** | **1283 (458 , 2859)** | **69 (24.4 , 153.3)** | **1.9 (-6.3 , 10.7)** | **20972 (13517 , 30979)** | **939.1 (606.1 , 1394.2)** | **-7.4 (-17.7 , 3.7)** | **4383 (2719 , 6344)** | **198 (126.7 , 282.2)** | **-28.2 (-45.5 , -7)** |
| **Liberia** | **3301 (1154 , 7291)** | **70.7 (24.8 , 157.6)** | **-1 (-8.6 , 7.4)** | **46624 (29317 , 70136)** | **867.2 (560.3 , 1291.9)** | **-22.4 (-30.9 , -13.2)** | **7856 (4932 , 11711)** | **150.8 (96.6 , 222.7)** | **-48.3 (-60.2 , -32.9)** |
| **Mali** | **15542 (5585 , 34655)** | **74.2 (26.7 , 162.6)** | **3.9 (-3.9 , 12.6)** | **339366 (219241 , 504683)** | **1226.2 (779 , 1806.8)** | **-1.6 (-9.7 , 7)** | **114810 (75166 , 172058)** | **381.4 (250.9 , 558.6)** | **-24.4 (-40.1 , -4.6)** |
| **Mauritania** | **2682 (950 , 6062)** | **67.5 (23.9 , 149.6)** | **-0.9 (-9 , 7.7)** | **38214 (24452 , 57782)** | **809.7 (518.6 , 1216.6)** | **-14.2 (-25.3 , -2.3)** | **5498 (3406 , 8463)** | **119.6 (75.6 , 177.9)** | **-43.1 (-57.8 , -23.2)** |
| **Niger** | **16412 (5818 , 36507)** | **70.7 (25 , 158.1)** | **-1.3 (-8.9 , 6.6)** | **331547 (215939 , 491401)** | **1047.5 (683.7 , 1548)** | **-12.7 (-21.1 , -3.3)** | **68194 (43120 , 100168)** | **216.4 (139.4 , 315.8)** | **-37.9 (-51.7 , -18.5)** |
| **Nigeria** | **150259 (53612 , 333511)** | **72.2 (25.6 , 159.7)** | **9.5 (1 , 18.5)** | **2705889 (1762740 , 4001529)** | **1028.8 (674 , 1510)** | **6.2 (-2.8 , 15.4)** | **694301 (449294 , 1030411)** | **250 (165.5 , 367.8)** | **-25 (-40 , -5.4)** |
| **Sao Tome and Principe** | **145 (51 , 319)** | **70.7 (24.9 , 156.5)** | **-4.9 (-14 , 4.6)** | **1416 (873 , 2132)** | **631.2 (397 , 938.8)** | **-25.7 (-39.2 , -9)** | **124 (79 , 186)** | **60.7 (39.3 , 89.4)** | **-50 (-62.3 , -34.1)** |
| **Senegal** | **11517 (4070 , 24680)** | **77 (27.2 , 165.6)** | **3.4 (-3.4 , 11)** | **181541 (118738 , 268407)** | **1045.9 (679.8 , 1544.1)** | **-15 (-22.8 , -5.9)** | **34621 (22068 , 50226)** | **204.3 (129.9 , 292.9)** | **-47.9 (-58.8 , -33.8)** |
| **Sierra Leone** | **6165 (2195 , 13748)** | **76.6 (27.1 , 170.8)** | **-1.1 (-8 , 5.8)** | **108186 (70499 , 160175)** | **1098.4 (715.1 , 1624.3)** | **-3.9 (-12.9 , 5.6)** | **24277 (15390 , 35508)** | **242.3 (155.2 , 350)** | **-22.7 (-40.2 , -0.3)** |
| **Togo** | **5563 (1961 , 12193)** | **70.9 (25 , 156.1)** | **-3.8 (-10.6 , 3)** | **97098 (63944 , 142916)** | **1053.2 (693.3 , 1552.7)** | **-9.3 (-17.7 , 0.1)** | **16474 (10718 , 24178)** | **183.2 (120.6 , 263.8)** | **-6.9 (-26.3 , 18.2)** |
| **Eastern Sub-Saharan Africa** | **247495 (88014 , 543307)** | **62.6 (22.2 , 137.4)** | **-4.9 (-6.9 , -2.7)** | **3413793 (2240064 , 5067326)** | **741.6 (487.6 , 1091.4)** | **-23.1 (-25.4 , -20.5)** | **719409 (482741 , 1010909)** | **166.7 (112.5 , 232.8)** | **-45.2 (-48.5 , -42)** |
| **Burundi** | **6580 (2365 , 14691)** | **56.3 (20.3 , 124.8)** | **-7.7 (-15.4 , 0.2)** | **98368 (64092 , 145640)** | **689.4 (450.6 , 1008.1)** | **-16.2 (-26.4 , -4.3)** | **16453 (10042 , 24704)** | **126.7 (81.7 , 184.1)** | **-36.6 (-51.5 , -16.5)** |
| **Comoros** | **474 (170 , 1024)** | **68.2 (24.6 , 148.2)** | **-5.8 (-14.1 , 2.6)** | **5272 (3361 , 7929)** | **731.7 (471.7 , 1104)** | **-26 (-34.5 , -15.8)** | **941 (605 , 1399)** | **133.9 (87.1 , 197.9)** | **-48.6 (-59.7 , -33.6)** |
| **Djibouti** | **758 (273 , 1676)** | **65.3 (23.6 , 143.1)** | **-7.3 (-15.1 , 2.4)** | **8004 (5121 , 12000)** | **641.9 (415.4 , 954.7)** | **-26.3 (-36.2 , -14.8)** | **1304 (834 , 1914)** | **108.6 (70.2 , 158)** | **-46.5 (-58.6 , -31.8)** |
| **Eritrea** | **4360 (1590 , 9701)** | **68 (24.6 , 151.5)** | **-4.9 (-12.7 , 3.5)** | **55454 (35413 , 82329)** | **785.7 (506 , 1164.4)** | **-26.2 (-34.4 , -17.3)** | **10867 (6816 , 16056)** | **162.1 (103.8 , 236.1)** | **-50.6 (-61.7 , -37.6)** |
| **Ethiopia** | **52060 (18560 , 112044)** | **49.4 (17.7 , 107.1)** | **-9.4 (-13 , -5.5)** | **736592 (484500 , 1088165)** | **571.6 (377.4 , 845.4)** | **-25.2 (-29.6 , -20.3)** | **149687 (100342 , 211086)** | **120.9 (82 , 170.3)** | **-49.6 (-55 , -43.5)** |
| **Kenya** | **23662 (8490 , 51797)** | **49.9 (17.9 , 109.6)** | **-2.2 (-4.3 , -0.1)** | **256828 (168887 , 374185)** | **519.7 (341.9 , 757.6)** | **-16.3 (-18.4 , -14.1)** | **64728 (43980 , 90503)** | **149.1 (101.3 , 209.1)** | **-26.8 (-29.9 , -23.5)** |
| **Madagascar** | **18468 (6422 , 41513)** | **73 (25.3 , 164.9)** | **-2.3 (-10 , 7)** | **200580 (128215 , 290470)** | **720.9 (466.1 , 1060.9)** | **-23.6 (-32.6 , -12.8)** | **29892 (18655 , 44044)** | **120.7 (77.3 , 174.5)** | **-46 (-56.7 , -32.5)** |
| **Malawi** | **12902 (4557 , 28556)** | **73.3 (26 , 159.6)** | **-2.6 (-10.7 , 6)** | **194630 (125215 , 286275)** | **950.8 (616.3 , 1398.3)** | **-21.5 (-29.1 , -13.3)** | **36769 (23062 , 53602)** | **192.2 (123.7 , 276.3)** | **-50.7 (-60.8 , -38.6)** |
| **Mozambique** | **20512 (7163 , 46250)** | **72.9 (25.4 , 163.5)** | **-0.7 (-8.2 , 6.9)** | **331651 (211865 , 488021)** | **964.9 (624.8 , 1440.6)** | **-17.2 (-25.2 , -8.7)** | **69960 (46289 , 103183)** | **211 (141.3 , 303.6)** | **-46.2 (-56.2 , -33.3)** |
| **Rwanda** | **6500 (2349 , 14383)** | **55.4 (20 , 122.8)** | **-11.8 (-21.3 , -2)** | **59777 (37448 , 87925)** | **471.4 (299.1 , 688)** | **-36.7 (-45 , -26.9)** | **9962 (6456 , 14850)** | **83.1 (54.7 , 121.6)** | **-61.7 (-70 , -52.1)** |
| **Somalia** | **13636 (4840 , 29967)** | **70.9 (25.4 , 155)** | **-3.3 (-10.9 , 3.9)** | **220013 (139950 , 333653)** | **989.1 (633.4 , 1483.1)** | **-18.2 (-25.8 , -10.4)** | **58655 (37678 , 85561)** | **279.5 (182.2 , 393.2)** | **-37.5 (-49.9 , -22.9)** |
| **South Sudan** | **6241 (2177 , 13509)** | **70.9 (24.7 , 155.4)** | **-3.8 (-11.1 , 3.6)** | **91665 (58396 , 135524)** | **881.2 (566.9 , 1304.1)** | **-11 (-20.9 , 0)** | **20443 (12828 , 30234)** | **207.4 (134.5 , 301.1)** | **-23.5 (-39 , -2.1)** |
| **United Republic of Tanzania** | **44243 (15446 , 97229)** | **79.4 (27.7 , 175.5)** | **-6.1 (-12.9 , 0.8)** | **593992 (384458 , 874214)** | **926.2 (603.3 , 1373)** | **-34.9 (-42.2 , -27.2)** | **98724 (62592 , 146021)** | **164.2 (106.8 , 239.1)** | **-60.5 (-68.8 , -50.9)** |
| **Uganda** | **22794 (8016 , 50682)** | **58.7 (20.8 , 129)** | **-7.7 (-15.2 , 0.7)** | **317991 (205193 , 478911)** | **680 (441.2 , 1014.9)** | **-27.7 (-35.8 , -18.5)** | **58708 (38476 , 85404)** | **131.7 (87.2 , 189.9)** | **-56.9 (-66.1 , -45.9)** |
| **Zambia** | **14107 (4957 , 31069)** | **82.7 (29.5 , 181.7)** | **12.6 (5.4 , 19.7)** | **240247 (157252 , 352435)** | **1203.7 (782.8 , 1758.1)** | **-2 (-9.7 , 6.1)** | **91739 (58270 , 134328)** | **467.9 (303 , 679.3)** | **-19.2 (-33.6 , -2)** |
| **Central Sub-Saharan Africa** | **89186 (31968 , 196690)** | **69.9 (25.1 , 153.7)** | **-5.3 (-9.9 , -0.5)** | **1273690 (829241 , 1873665)** | **852.9 (554.5 , 1261.2)** | **-23.1 (-29.1 , -17.1)** | **236779 (160191 , 336566)** | **166.3 (113.4 , 235.8)** | **-50.8 (-58.7 , -41.5)** |
| **Angola** | **19405 (7024 , 42467)** | **66.9 (24.2 , 148.8)** | **1.3 (-7.7 , 10.8)** | **220887 (138385 , 331790)** | **629.6 (394.1 , 940.7)** | **-12.8 (-24.5 , 0.9)** | **30508 (19488 , 44513)** | **93.2 (60.7 , 136.9)** | **-49.3 (-60 , -36.3)** |
| **Central African Republic** | **3801 (1348 , 8337)** | **72.4 (25.5 , 158)** | **-1.3 (-8.2 , 6.1)** | **59668 (38496 , 90914)** | **974.5 (635.4 , 1468.6)** | **-5.2 (-16.2 , 6.5)** | **9760 (6240 , 14476)** | **182.1 (118.3 , 268.4)** | **1 (-19 , 26)** |
| **Congo** | **4020 (1426 , 8886)** | **78.2 (27.8 , 172.6)** | **2.4 (-5.4 , 11.2)** | **50485 (32628 , 74921)** | **892.7 (579.2 , 1319.3)** | **-13.3 (-23.3 , -1.6)** | **6581 (4193 , 9667)** | **124.1 (79.7 , 179.9)** | **-41.5 (-54.2 , -26.1)** |
| **Democratic Republic of the Congo** | **59582 (21296 , 129762)** | **70 (25.1 , 155.5)** | **-7.5 (-13.8 , -1.3)** | **911981 (591952 , 1347303)** | **917.8 (597.4 , 1347.7)** | **-24.8 (-32.2 , -17.2)** | **185403 (122263 , 270389)** | **193.7 (130.7 , 275.5)** | **-51.6 (-60.8 , -39.5)** |
| **Equatorial Guinea** | **993 (347 , 2176)** | **72.4 (25.6 , 157.7)** | **-3.7 (-11.9 , 4.3)** | **11638 (7374 , 17314)** | **750.2 (471.9 , 1116.7)** | **-36.5 (-44.7 , -27.7)** | **1501 (933 , 2308)** | **101.6 (64.4 , 150.8)** | **-71.1 (-77.9 , -62.2)** |
| **Gabon** | **1386 (484 , 3037)** | **80.2 (28.2 , 177.1)** | **1.2 (-5.8 , 8.6)** | **19031 (12128 , 27647)** | **1051.7 (670 , 1520.1)** | **-12.7 (-21.8 , -3.2)** | **3025 (1971 , 4333)** | **174.6 (113.8 , 249.7)** | **-41.3 (-53.8 , -26.4)** |
