# Supplementary figures and images for: Burden of anemia and its underlying causes in 204 countries and territories, 1990–2019: results from the Global Burden of Disease Study 2019
Source: J Hematol Oncol. 2021 Nov 4;14:185. doi: 10.1186/s13045-021-01202-2 (PMC8567696; doi:10.1186/s13045-021-01202-2)

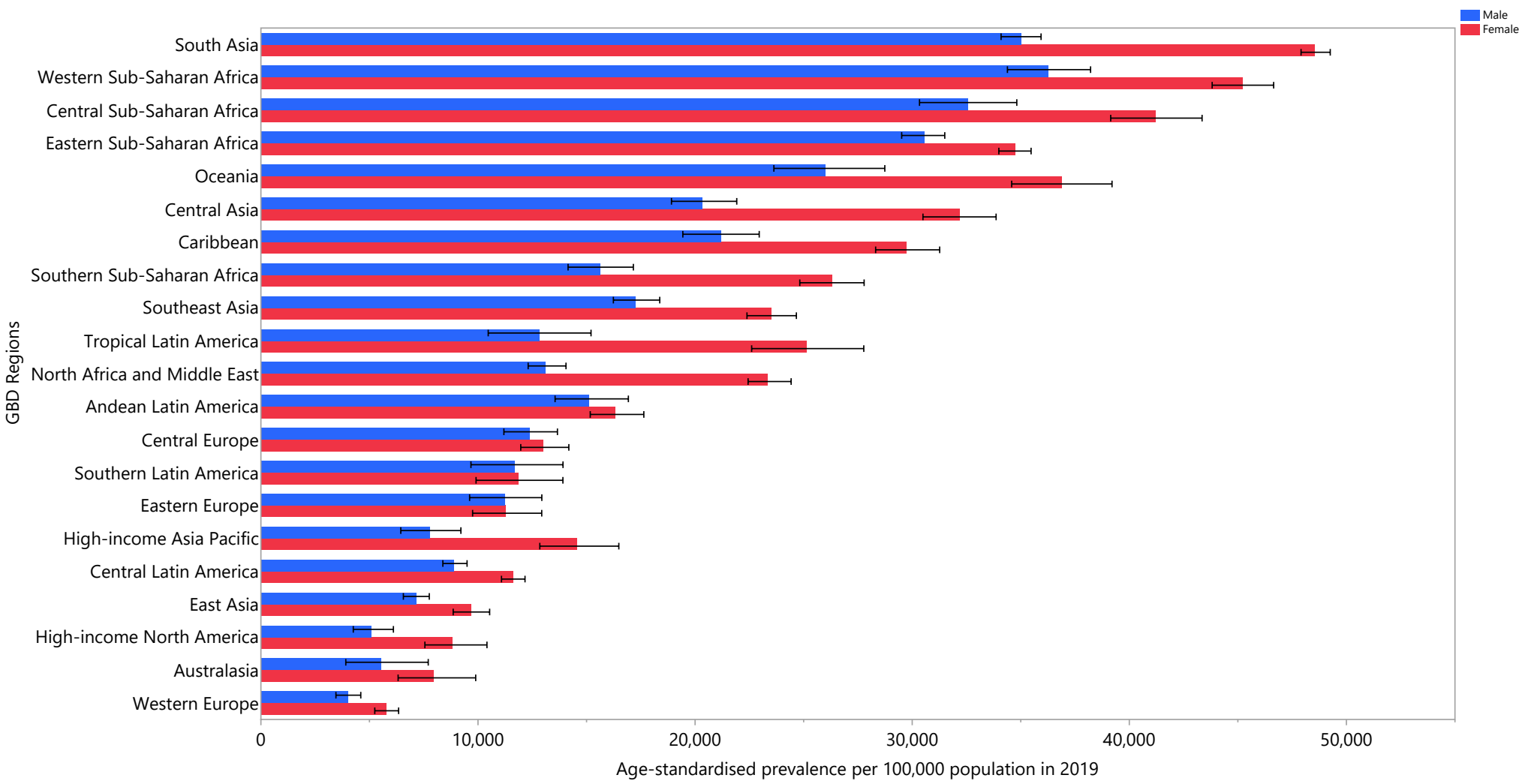

Supplement: Supplementary file 4 — Additional file 4: Figure S1. The age-standardized point prevalence of anemia in 2019 for the 21 Global Burden of Disease regions, by sex. (Generated from data available from http://ghdx.healthdata.org/gbd-results-tool). [file 13045_2021_1202_MOESM4_ESM.pdf]

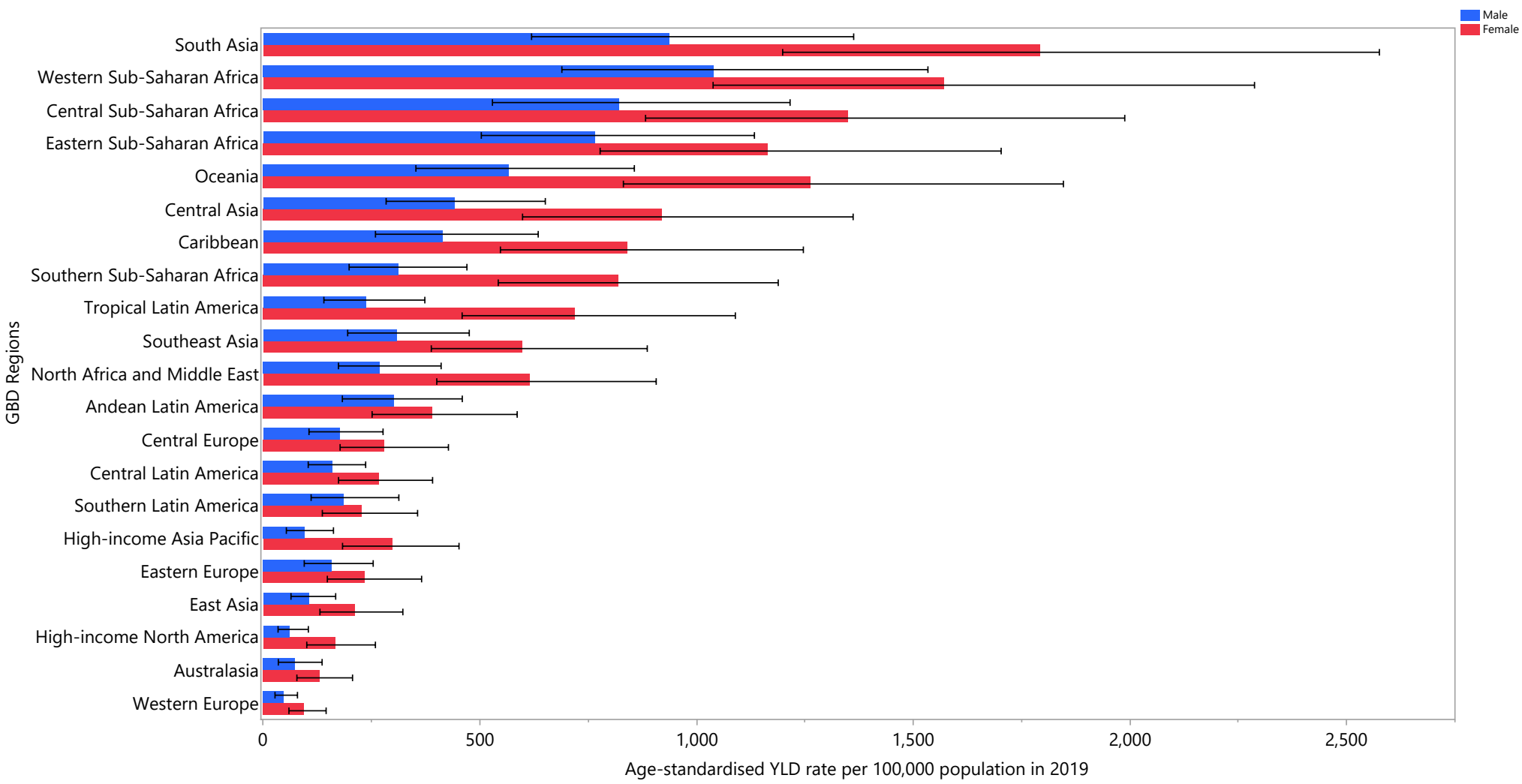

Supplement: Supplementary file 5 — Additional file 5: Figure S2. The age-standardized years lived with disability (YLDs) rates of anemia in 2019 for the 21 Global Burden of Disease regions, by sex. (Generated from data available from http://ghdx.healthdata.org/gbd-results-tool). [file 13045_2021_1202_MOESM5_ESM.pdf]

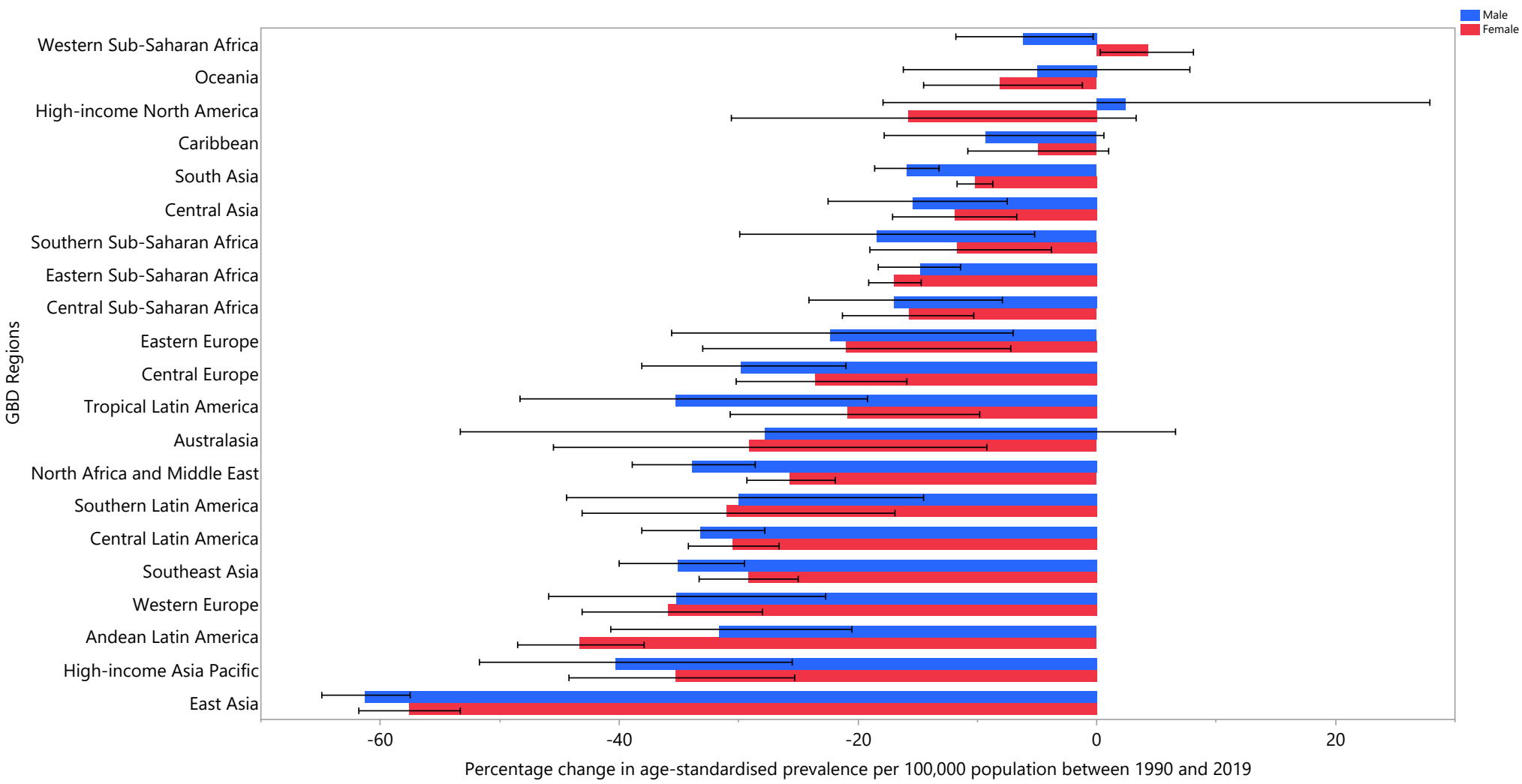

Supplement: Supplementary file 6 — Additional file 6: Figure S3. The percentage change in the age-standardized point prevalence of anemia from 1990 to 2019 for the 21 Global Burden of Disease regions, by sex. (Generated from data available from http://ghdx.healthdata.org/gbd-results-tool). [file 13045_2021_1202_MOESM6_ESM.pdf]

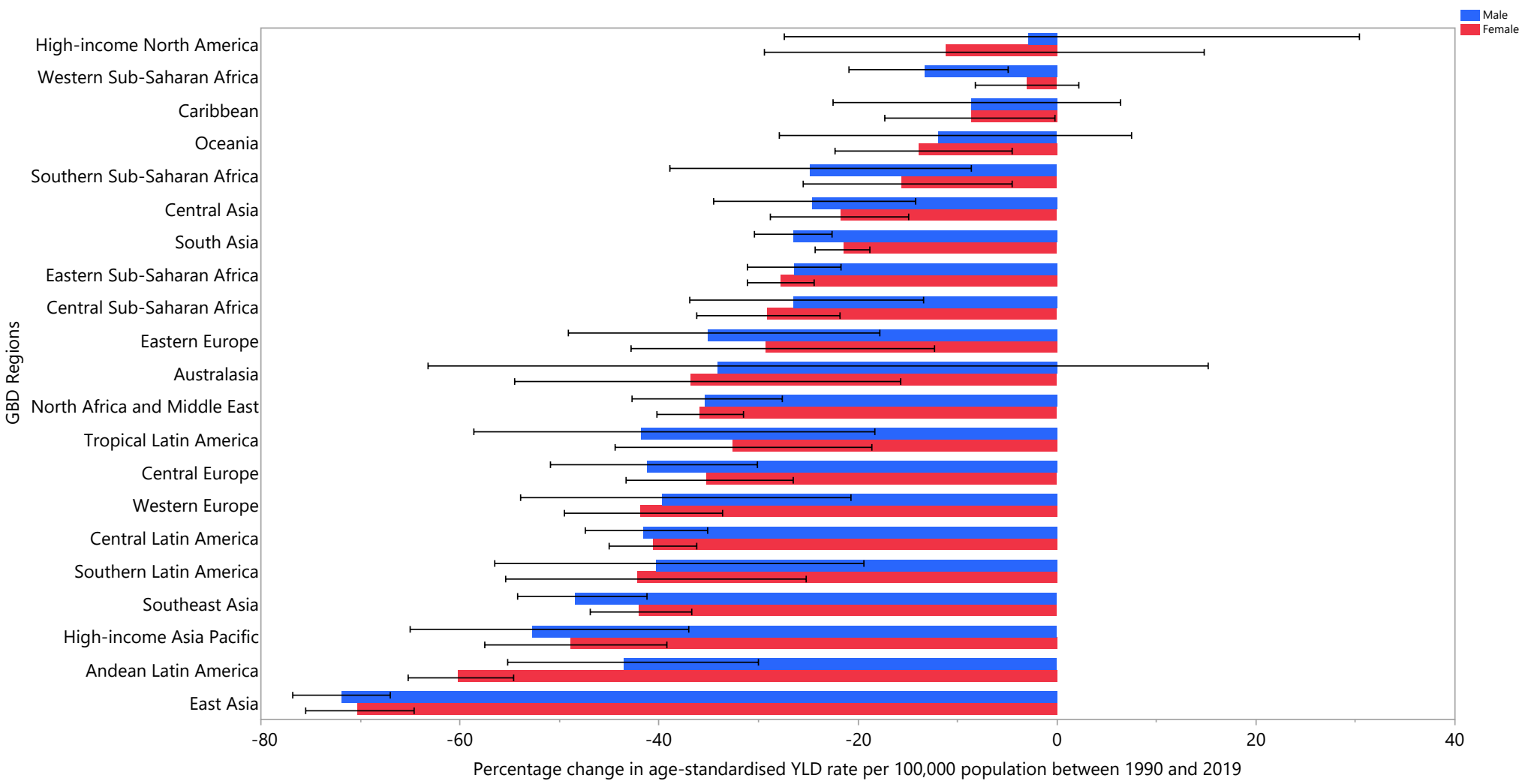

Supplement: Supplementary file 7 — Additional file 7: Figure S4. The percentage change in the age-standardized years lived with disability (YLDs) rates of anemia from 1990 to 2019 for the 21 Global Burden of Disease regions, by sex. (Generated from data available from http://ghdx.healthdata.org/gbd-results-tool). [file 13045_2021_1202_MOESM7_ESM.pdf]

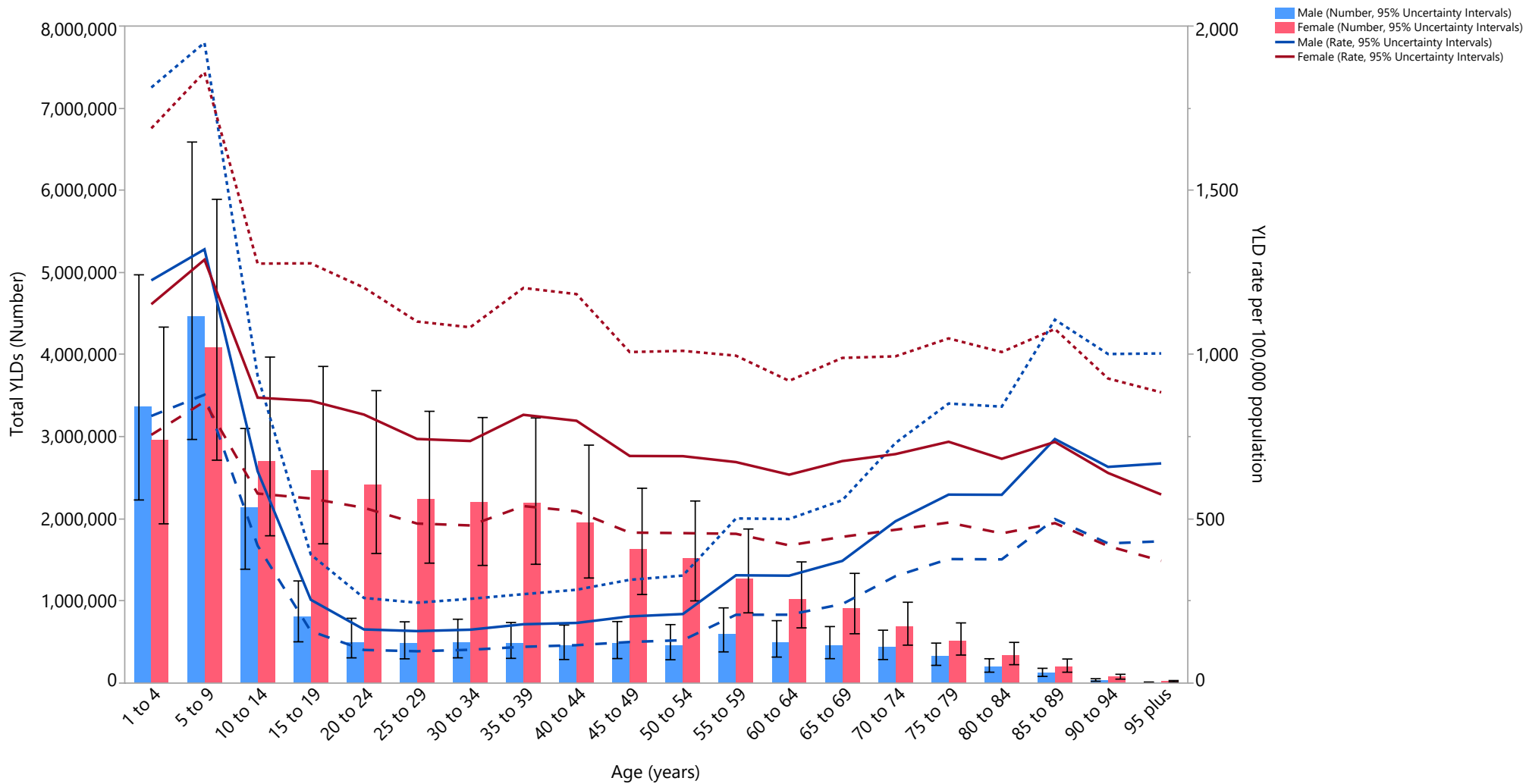

Supplement: Supplementary file 10 — Additional file 10: Figure S5. Global number of years lived with disability (YLDs) cases and years lived with disability (YLDs) of anemia per 100,000 population, by age and sex in 2019; Dotted and dashed lines indicate 95% upper and lower uncertainty intervals, respectively. (Generated from data available from http://ghdx.healthdata.org/gbd-results-tool). [file 13045_2021_1202_MOESM10_ESM.pdf]

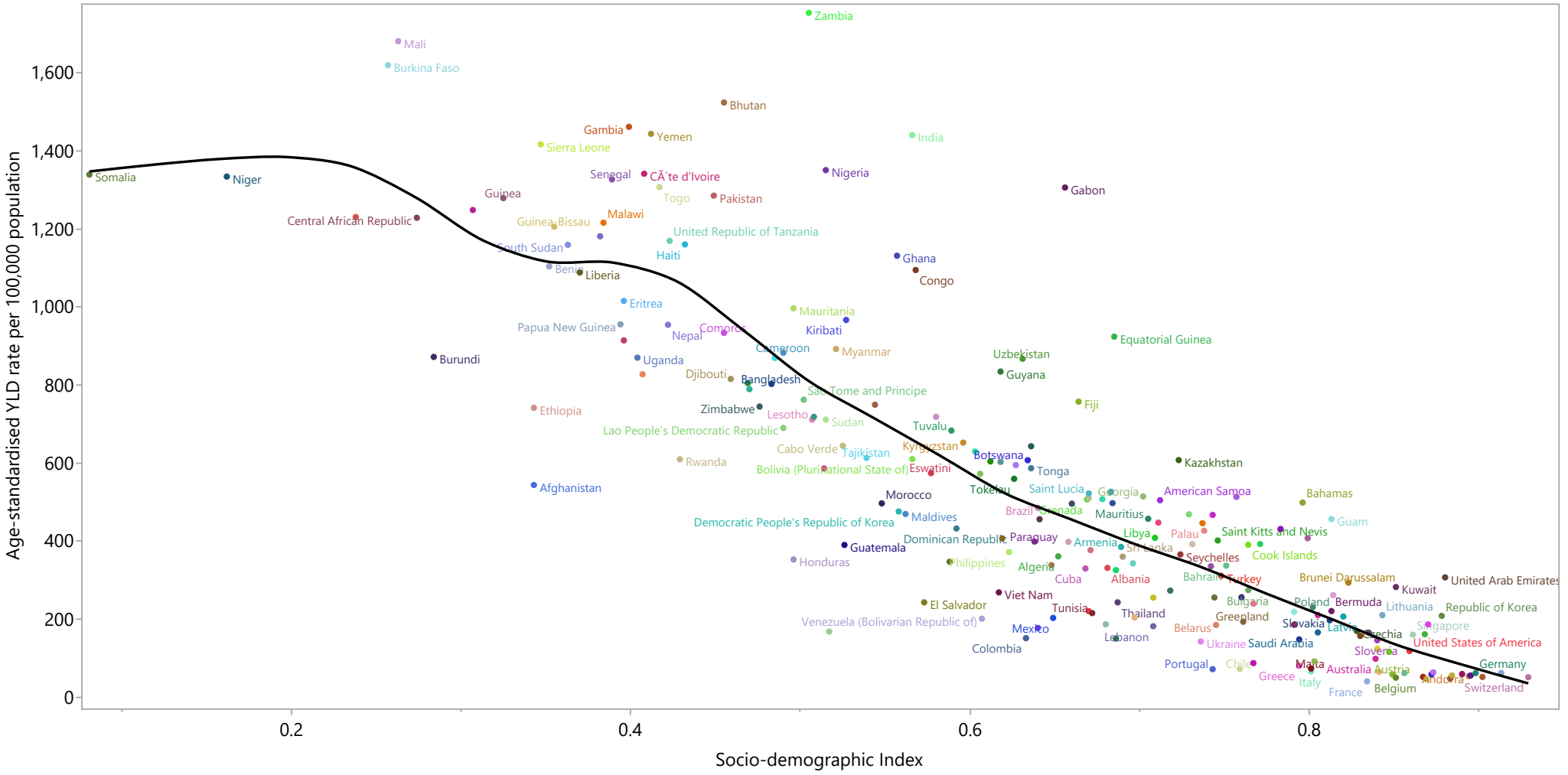

Supplement: Supplementary file 11 — Additional file 11: Figure S6. Age-standardized YLD rates of anemia for 204 countries and territories by Socio-demographic Index, in 2019; Expected values based on the Socio-demographic Index and disease rates in all locations are shown as the black line. Each point shows the observed age-standardized YLD rate for each country in 2019. YLD = years lived with disability. (Generated from data available from http://ghdx.healthdata.org/gbd-results-tool). [file 13045_2021_1202_MOESM11_ESM.pdf]

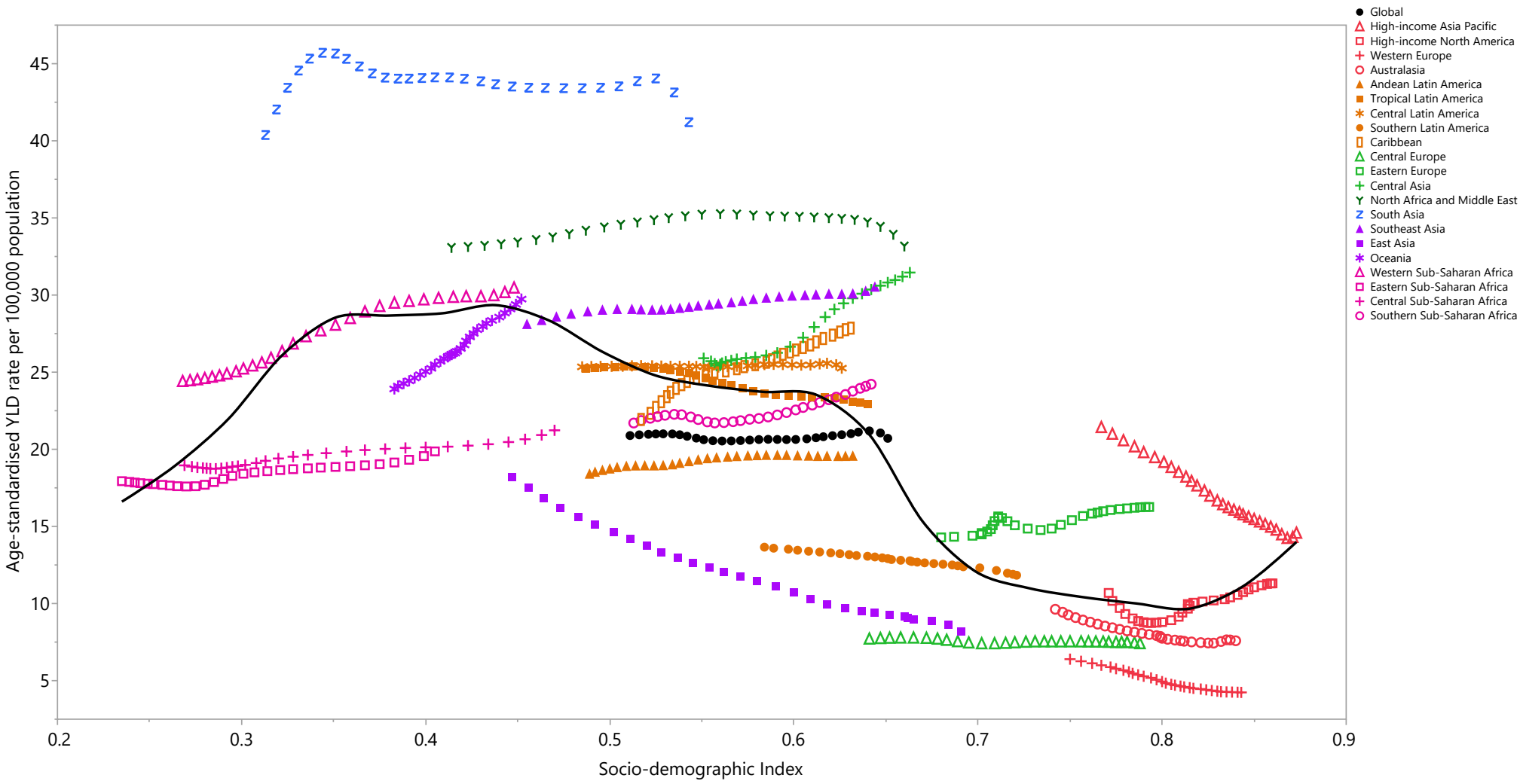

Supplement: Supplementary file 12 — Additional file 12: Figure S7. Age-standardized YLD rates of anemia attributable to chronic kidney disease for the 21 Global Burden of Disease regions by Socio-demographic Index, 1990–2019; Expected values based on Socio-demographic Index and disease rates in all locations are shown as the black line. Thirty points are plotted for each GBD region and show the observed age-standardized YLD rates from 1990 to 2019 for that region. YLD = years lived with disability. (Generated from data available from http://ghdx.healthdata.org/gbd-results-tool). [file 13045_2021_1202_MOESM12_ESM.pdf]

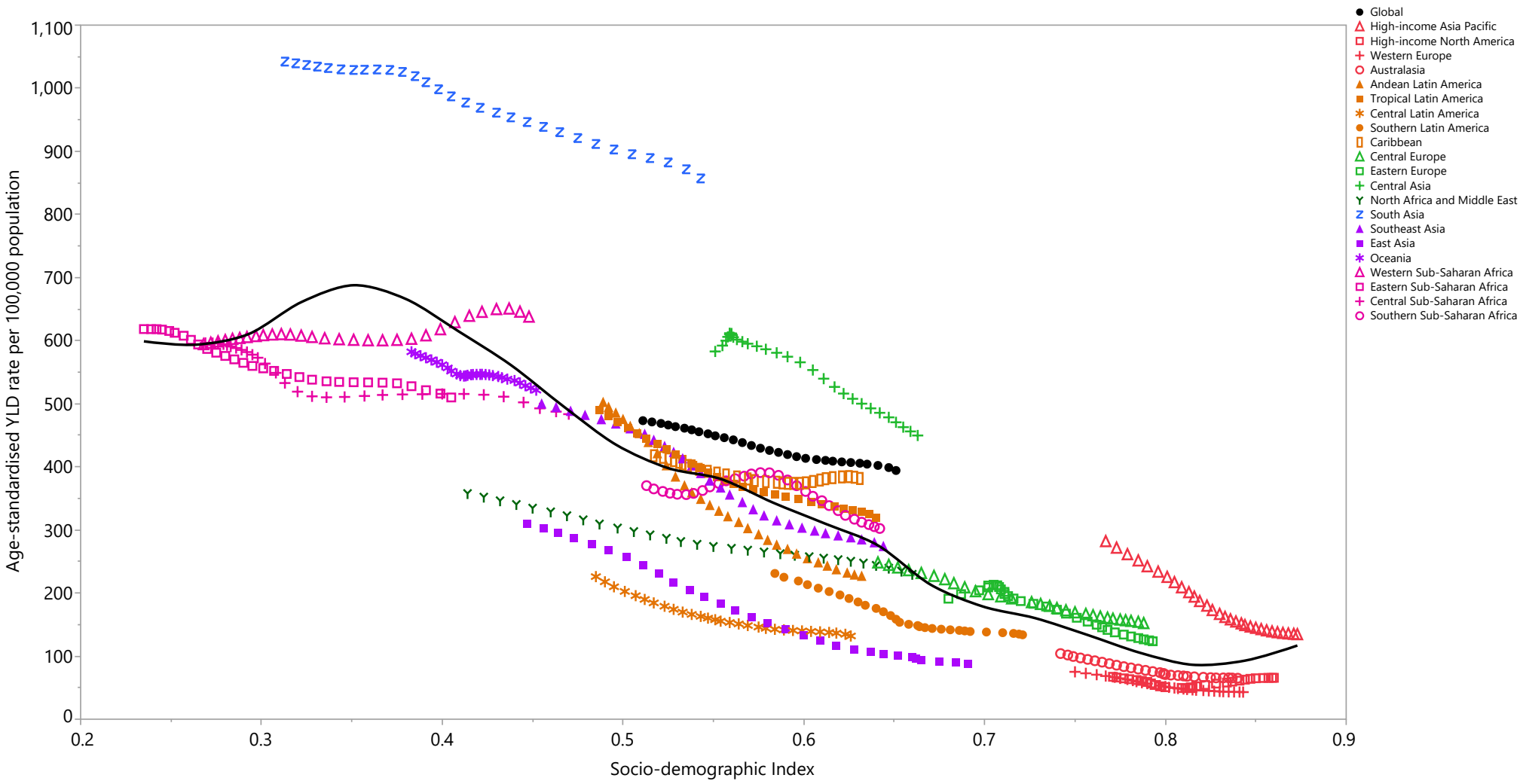

Supplement: Supplementary file 13 — Additional file 13: Figure S8. Age-standardized YLD rates of anemia attributable to dietary iron deficiency for the 21 Global Burden of Disease regions by Socio-demographic Index, 1990–2019; Expected values based on Socio-demographic Index and disease rates in all locations are shown as the black line. Thirty points are plotted for each GBD region and show the observed age-standardized YLD rates from 1990 to 2019 for that region. YLD = years lived with disability. (Generated from data available from http://ghdx.healthdata.org/gbd-results-tool). [file 13045_2021_1202_MOESM13_ESM.pdf]

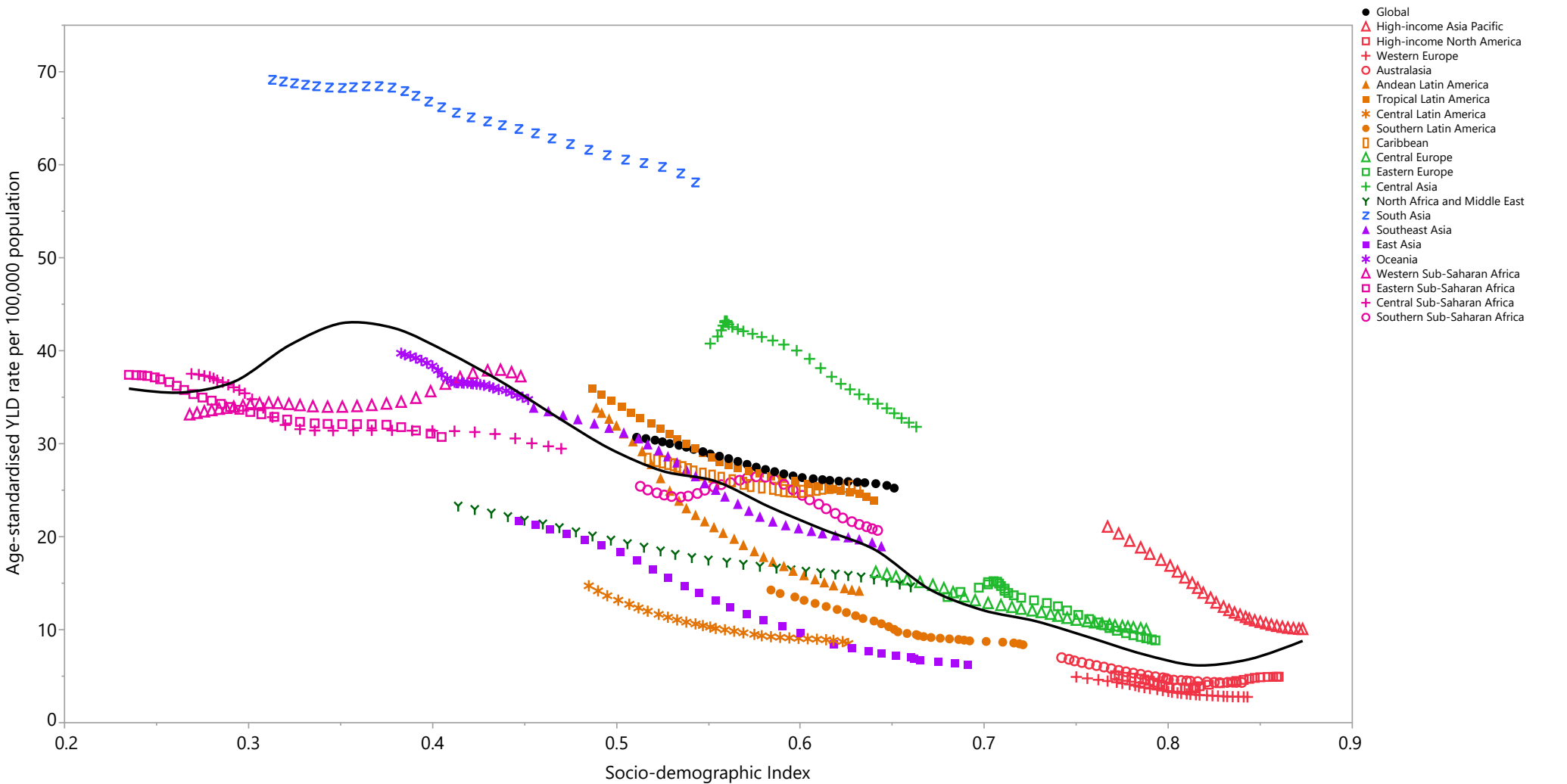

Supplement: Supplementary file 14 — Additional file 14: Figure S9. Age-standardized YLD rates of anemia attributable to endocrine, metabolic, blood, and immune disorders for the 21 Global Burden of Disease regions by Socio-demographic Index, 1990–2019; Expected values based on Socio-demographic Index and disease rates in all locations are shown as the black line. Thirty points are plotted for each GBD region and show the observed age-standardized YLD rates from 1990 to 2019 for that region. YLD = years lived with disability. (Generated from data available from http://ghdx.healthdata.org/gbd-results-tool). [file 13045_2021_1202_MOESM14_ESM.pdf]

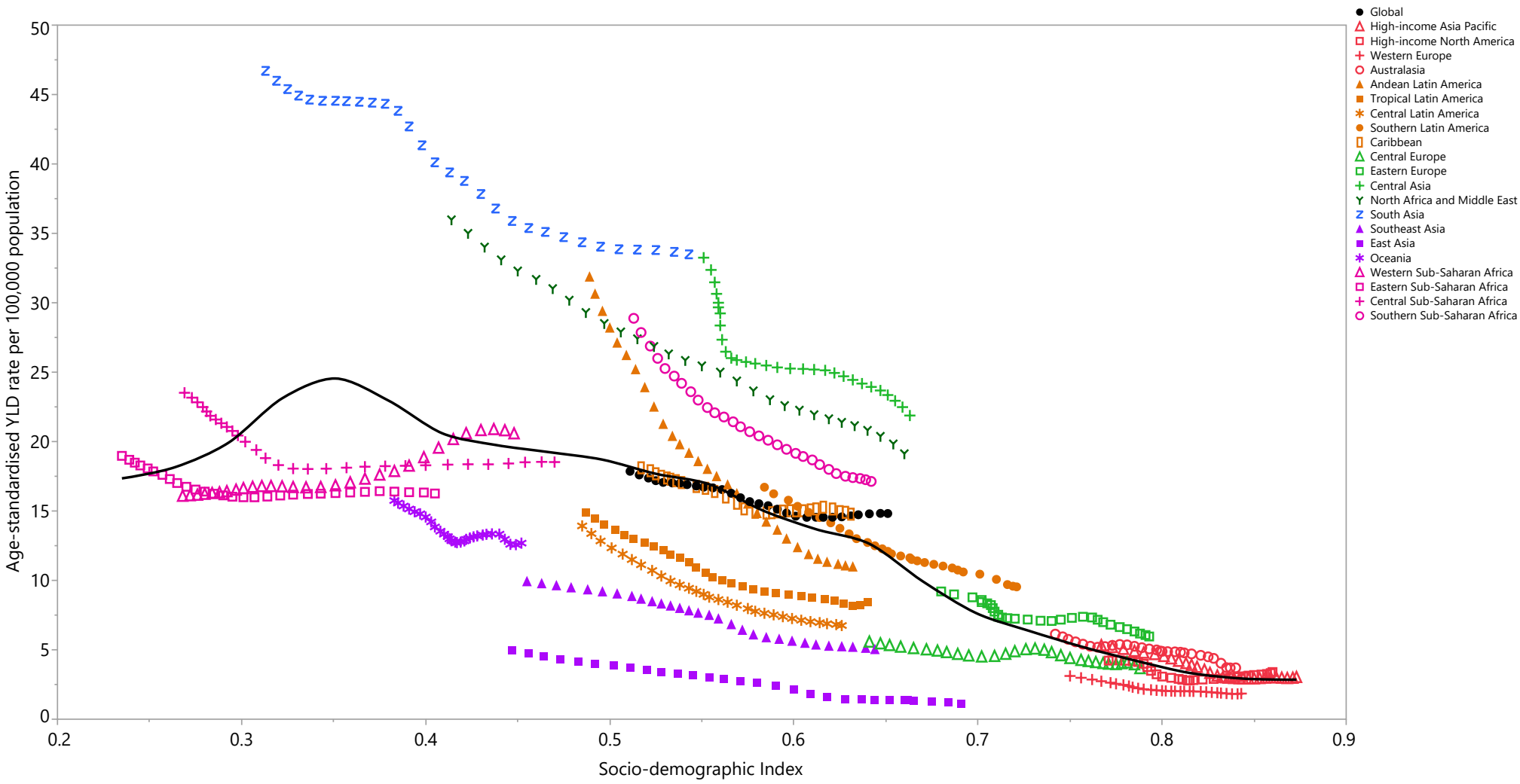

Supplement: Supplementary file 15 — Additional file 15: Figure S10. Age-standardized YLD rates of anemia attributable to gynecological diseases for the 21 Global Burden of Disease regions by Socio-demographic Index, 1990–2019; Expected values based on Socio-demographic Index and disease rates in all locations are shown as the black line. Thirty points are plotted for each GBD region and show the observed age-standardized YLD rates from 1990 to 2019 for that region. YLD = years lived with disability. (Generated from data available from http://ghdx.healthdata.org/gbd-results-tool). [file 13045_2021_1202_MOESM15_ESM.pdf]

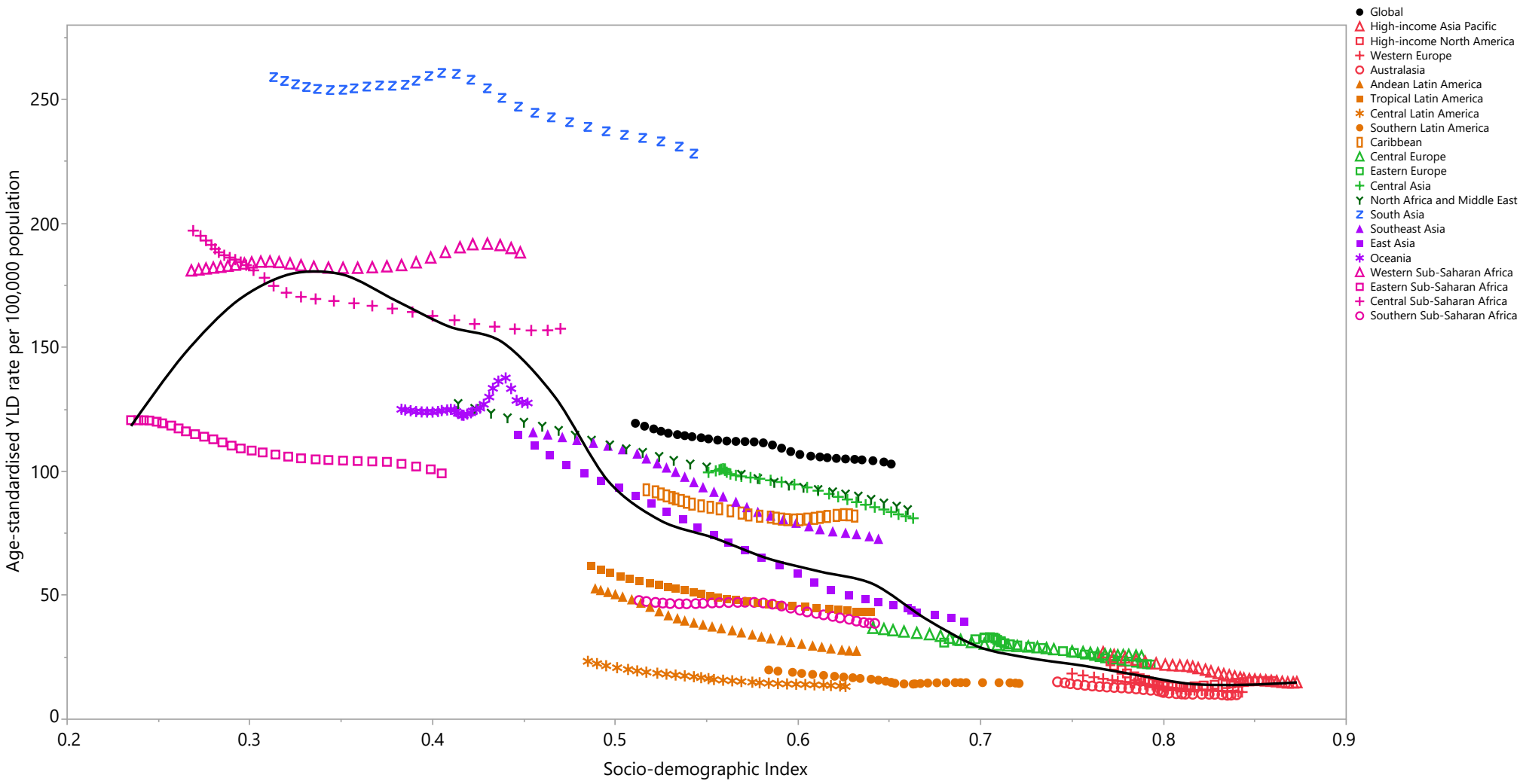

Supplement: Supplementary file 16 — Additional file 16: Figure S11. Age-standardized YLD rates of anemia attributable to hemoglobinopathies and hemolytic anemias for the 21 Global Burden of Disease regions by Socio-demographic Index, 1990–2019; Expected values based on Socio-demographic Index and disease rates in all locations are shown as the black line. Thirty points are plotted for each GBD region and show the observed age-standardized YLD rates from 1990 to 2019 for that region. YLD = years lived with disability. (Generated from data available from http://ghdx.healthdata.org/gbd-results-tool). [file 13045_2021_1202_MOESM16_ESM.pdf]

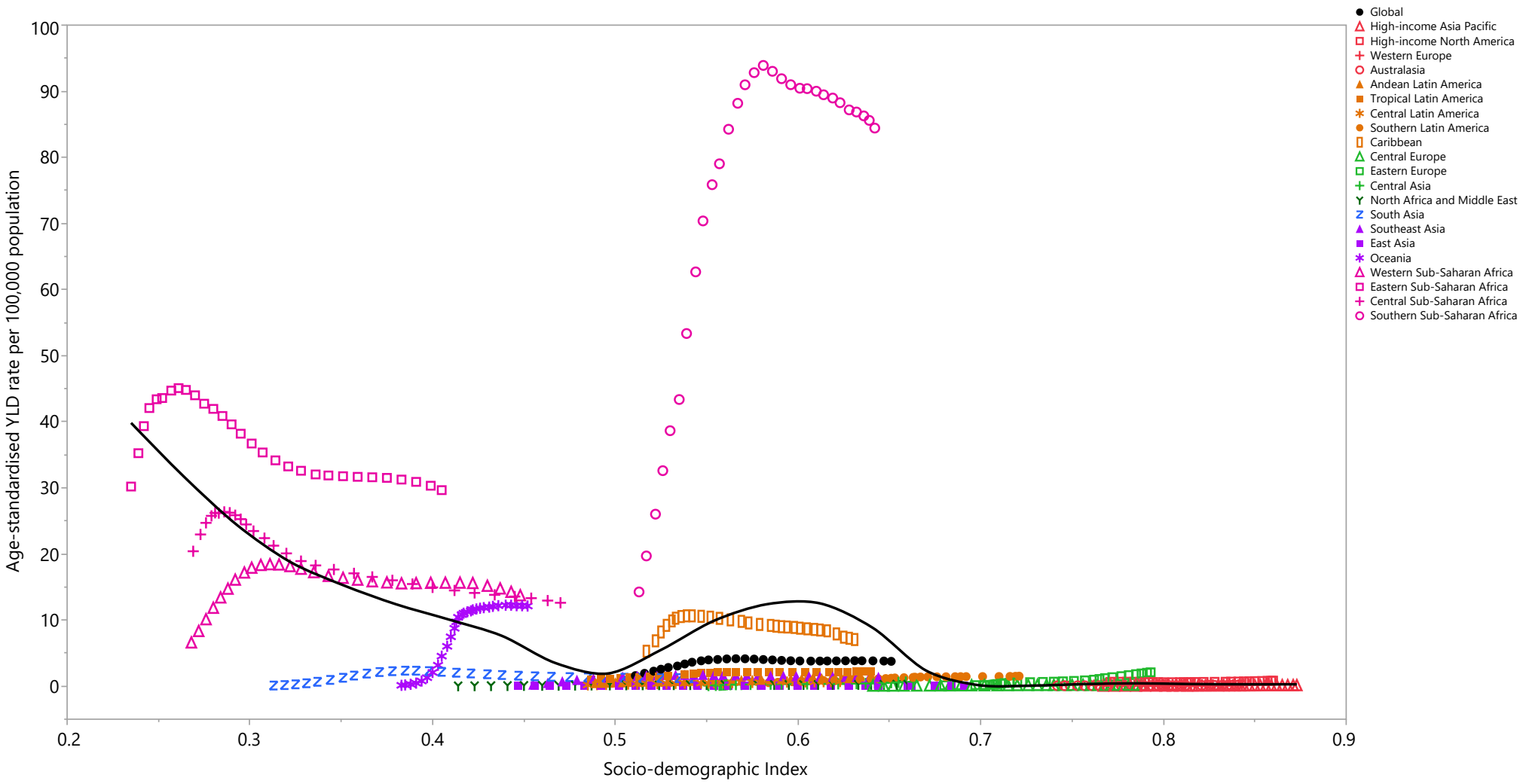

Supplement: Supplementary file 17 — Additional file 17: Figure S12. Age-standardized YLD rates of anemia attributable to HIV/AIDS for the 21 Global Burden of Disease regions by Socio-demographic Index, 1990–2019; Expected values based on Socio-demographic Index and disease rates in all locations are shown as the black line. Thirty points are plotted for each GBD region and show the observed age-standardized YLD rates from 1990 to 2019 for that region. YLD = years lived with disability, HIV= human immunodeficiency virus, AIDS: acquired immunodeficiency syndrome (Generated from data available from http://ghdx.healthdata.org/gbd-results-tool). [file 13045_2021_1202_MOESM17_ESM.pdf]

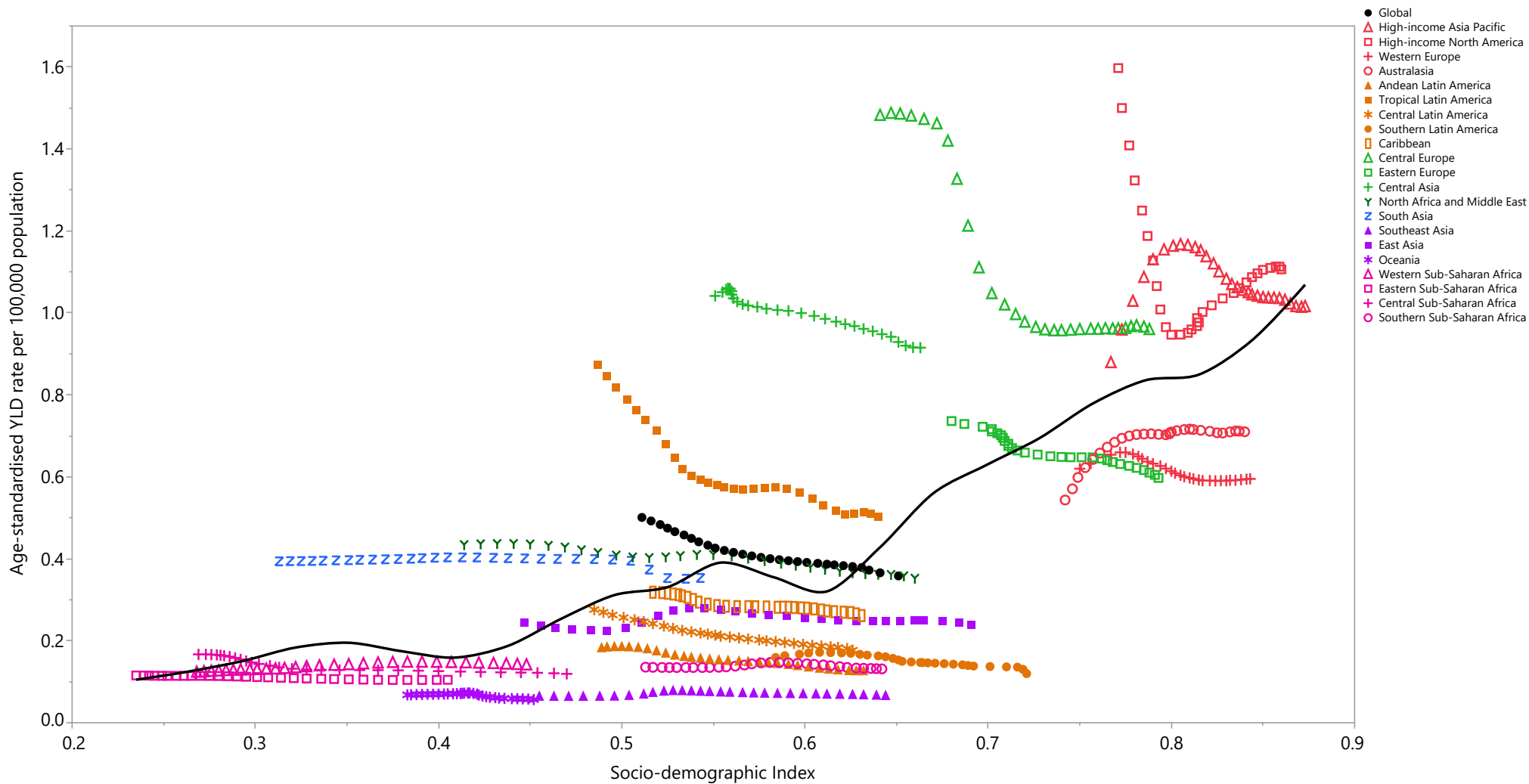

Supplement: Supplementary file 18 — Additional file 18: Figure S13. Age-standardized YLD rates of anemia attributable to inflammatory bowel disease for the 21 Global Burden of Disease regions by Socio-demographic Index, 1990–2019; Expected values based on Socio-demographic Index and disease rates in all locations are shown as the black line. Thirty points are plotted for each GBD region and show the observed age-standardized YLD rates from 1990 to 2019 for that region. YLD = years lived with disability. (Generated from data available from http://ghdx.healthdata.org/gbd-results-tool). [file 13045_2021_1202_MOESM18_ESM.pdf]

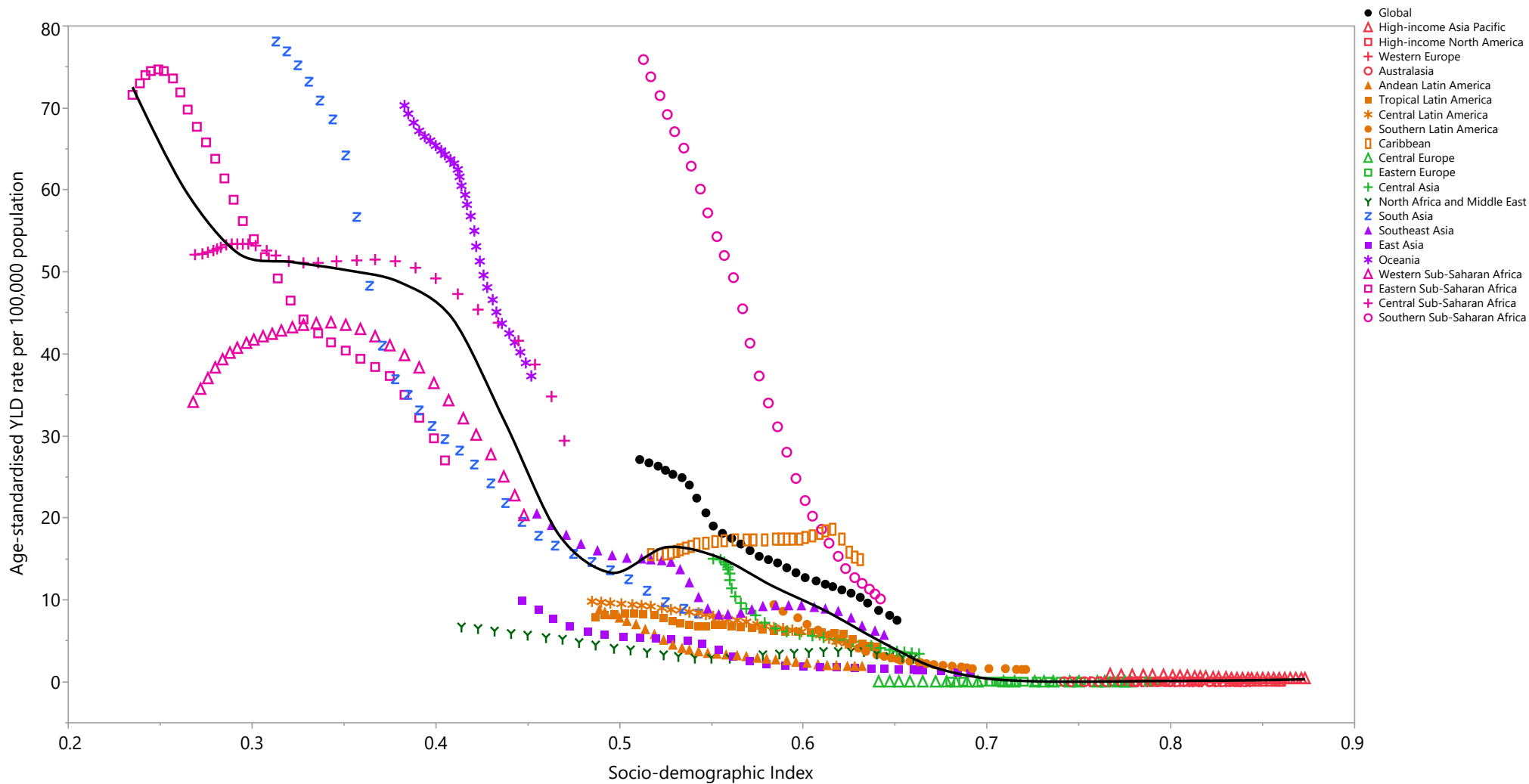

Supplement: Supplementary file 19 — Additional file 19: Figure S14. Age-standardized YLD rates of anemia attributable to intestinal nematode infections for the 21 Global Burden of Disease regions by Socio-demographic Index, 1990–2019; Expected values based on Socio-demographic Index and disease rates in all locations are shown as the black line. Thirty points are plotted for each GBD region and show the observed age-standardized YLD rates from 1990 to 2019 for that region. YLD = years lived with disability. (Generated from data available from http://ghdx.healthdata.org/gbd-results-tool). [file 13045_2021_1202_MOESM19_ESM.pdf]

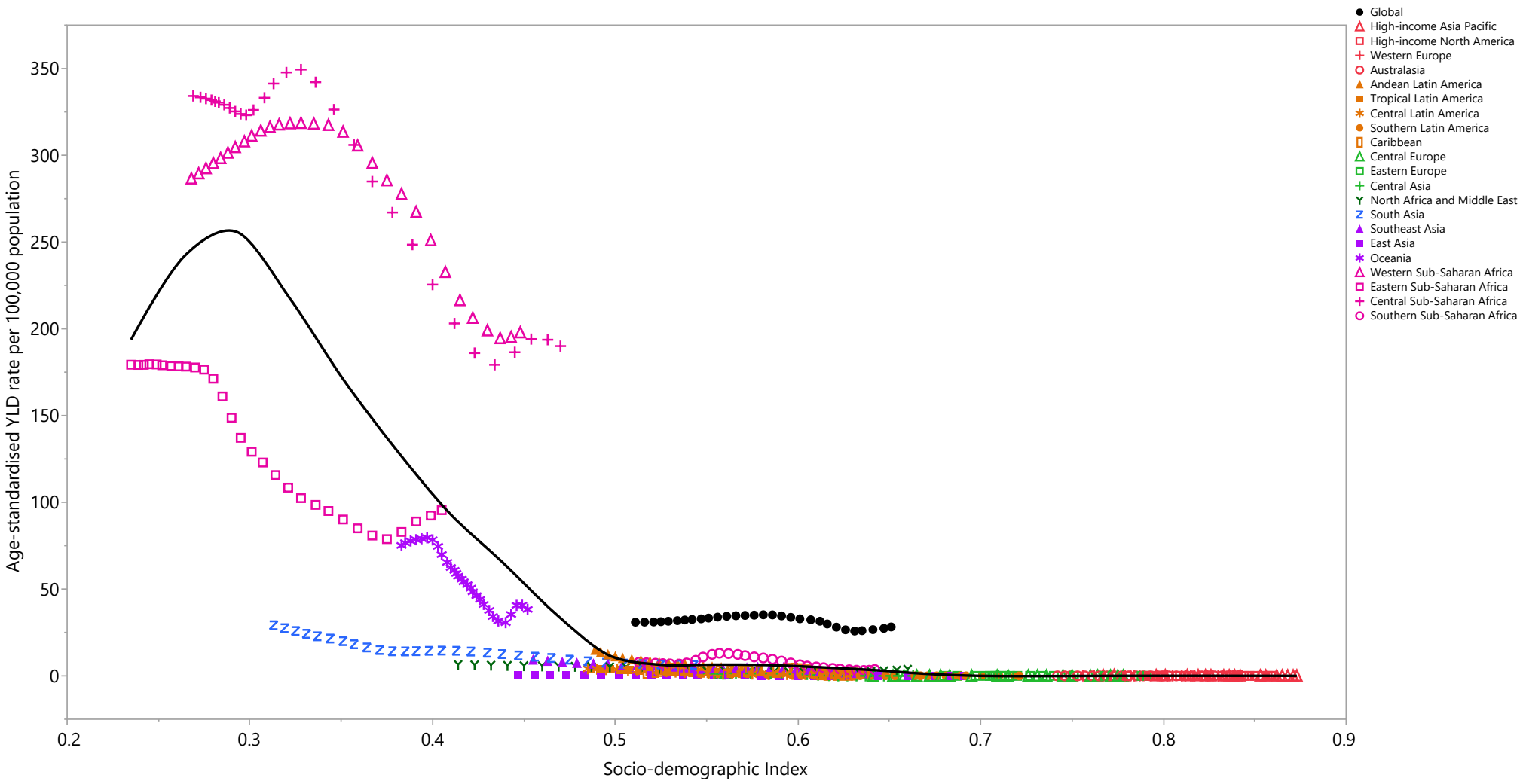

Supplement: Supplementary file 20 — Additional file 20: Figure S15. Age-standardized YLD rates of anemia attributable to malaria for the 21 Global Burden of Disease regions by Socio-demographic Index, 1990–2019; Expected values based on Socio-demographic Index and disease rates in all locations are shown as the black line. Thirty points are plotted for each GBD region and show the observed age-standardized YLD rates from 1990 to 2019 for that region. YLD = years lived with disability. (Generated from data available from http://ghdx.healthdata.org/gbd-results-tool). [file 13045_2021_1202_MOESM20_ESM.pdf]

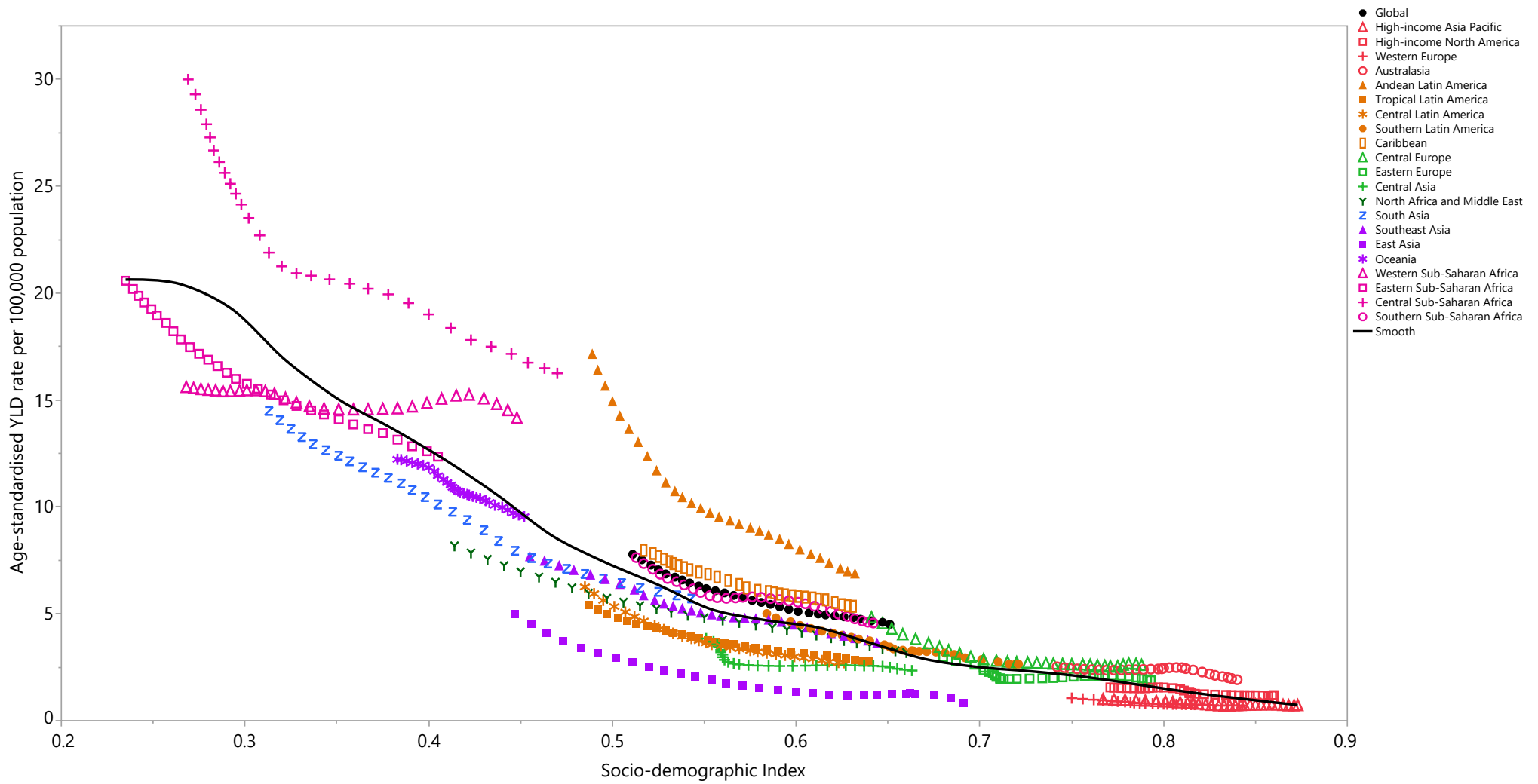

Supplement: Supplementary file 21 — Additional file 21: Figure S16. Age-standardized YLD rates of anemia attributable to maternal disorders for the 21 Global Burden of Disease regions by Socio-demographic Index, 1990–2019; Expected values based on Socio-demographic Index and disease rates in all locations are shown as the black line. Thirty points are plotted for each GBD region and show the observed age-standardized YLD rates from 1990 to 2019 for that region. YLD = years lived with disability. (Generated from data available from http://ghdx.healthdata.org/gbd-results-tool). [file 13045_2021_1202_MOESM21_ESM.pdf]

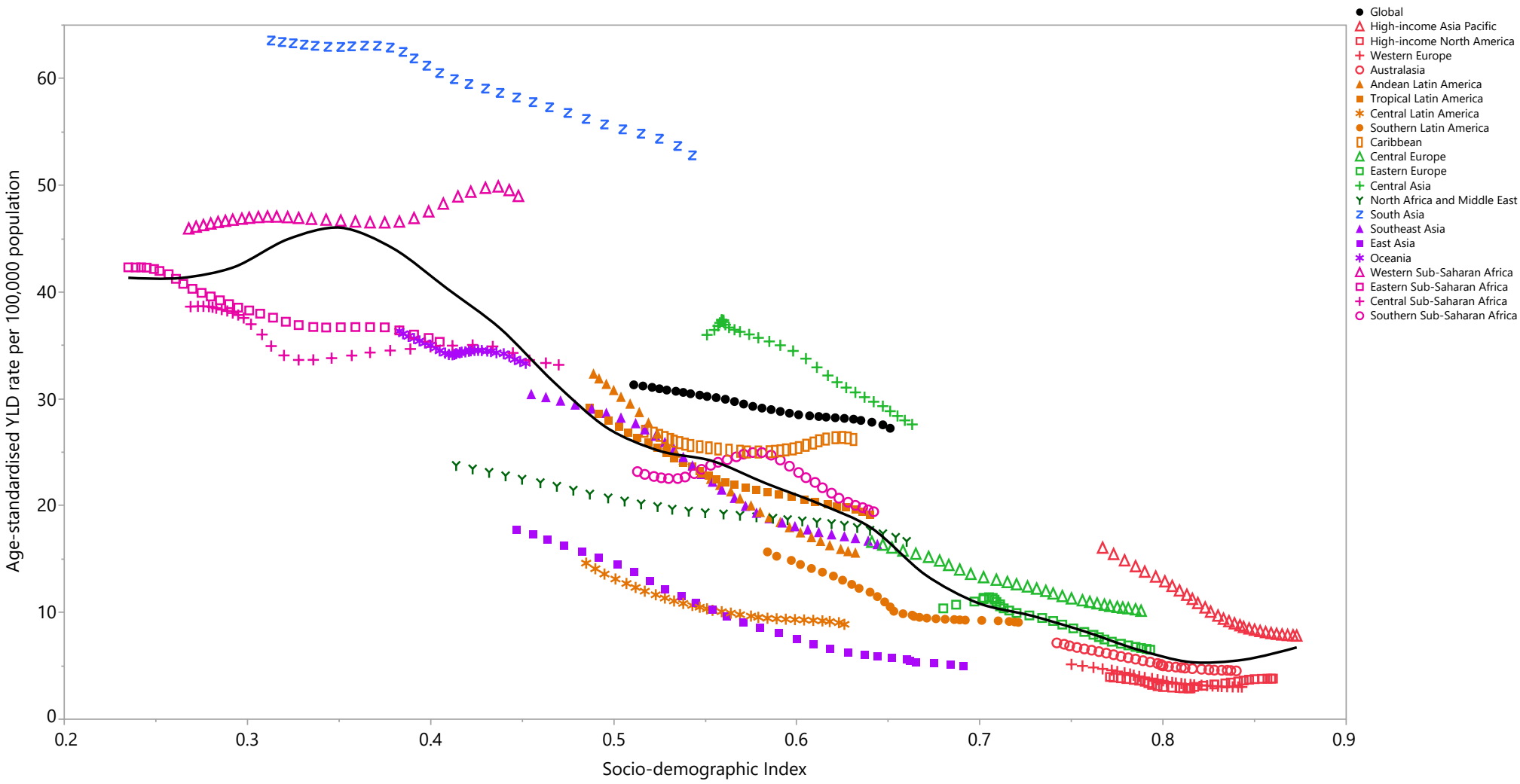

Supplement: Supplementary file 22 — Additional file 22: Figure S17. Age-standardized YLD rates of anemia attributable to “other neglected tropical diseases” for the 21 Global Burden of Disease regions by Socio-demographic Index, 1990–2019; Expected values based on Socio-demographic Index and disease rates in all locations are shown as the black line. Thirty points are plotted for each GBD region and show the observed age-standardized YLD rates from 1990 to 2019 for that region. YLD = years lived with disability. (Generated from data available from http://ghdx.healthdata.org/gbd-results-tool). [file 13045_2021_1202_MOESM22_ESM.pdf]

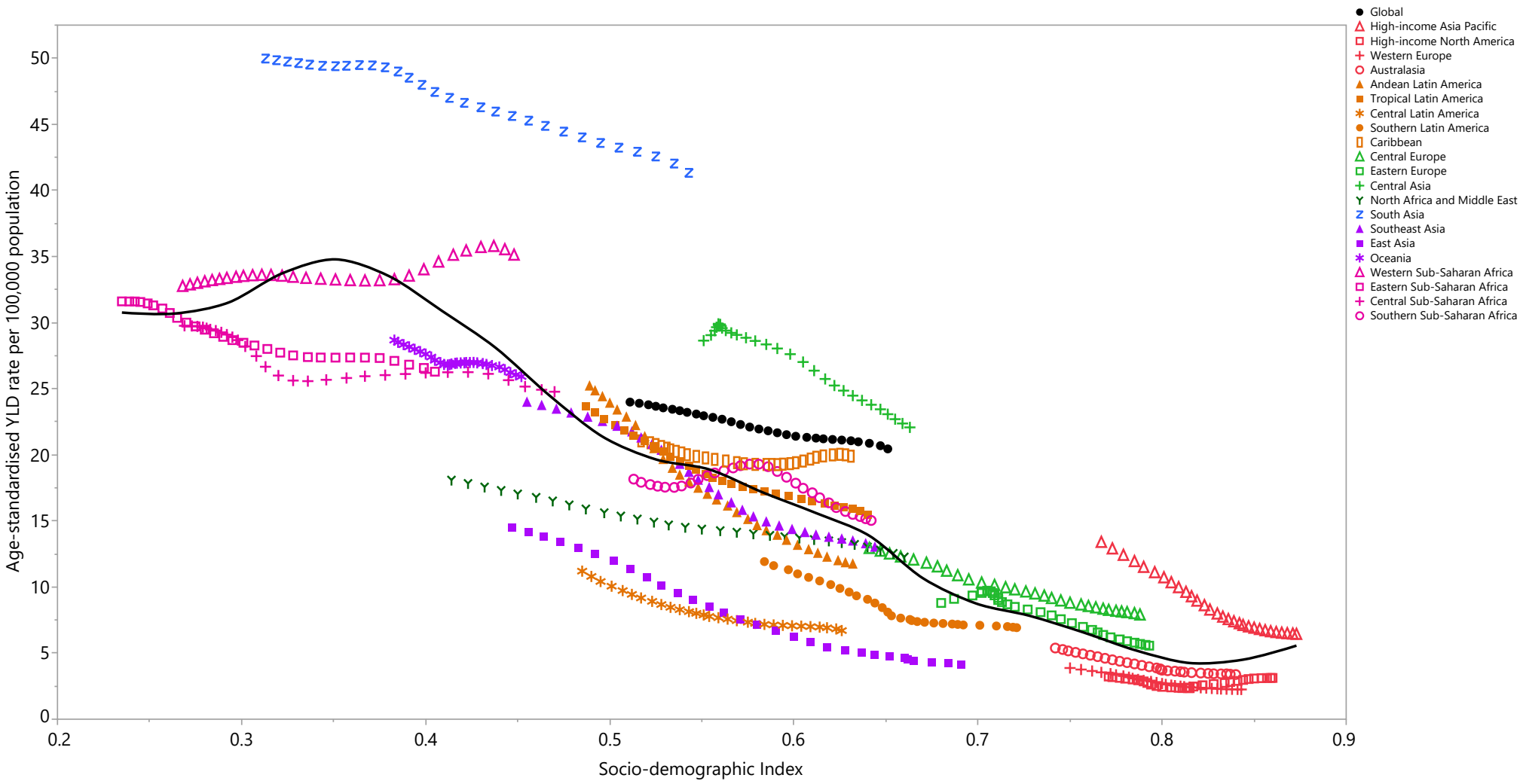

Supplement: Supplementary file 23 — Additional file 23: Figure S18. Age-standardized YLD rates of anemia attributable to “other unspecified infectious diseases” for the 21 Global Burden of Disease regions by Socio-demographic Index, 1990–2019; Expected values based on Socio-demographic Index and disease rates in all locations are shown as the black line. Thirty points are plotted for each GBD region and show the observed age-standardized YLD rates from 1990 to 2019 for that region. YLD = years lived with disability. (Generated from data available from http://ghdx.healthdata.org/gbd-results-tool). [file 13045_2021_1202_MOESM23_ESM.pdf]

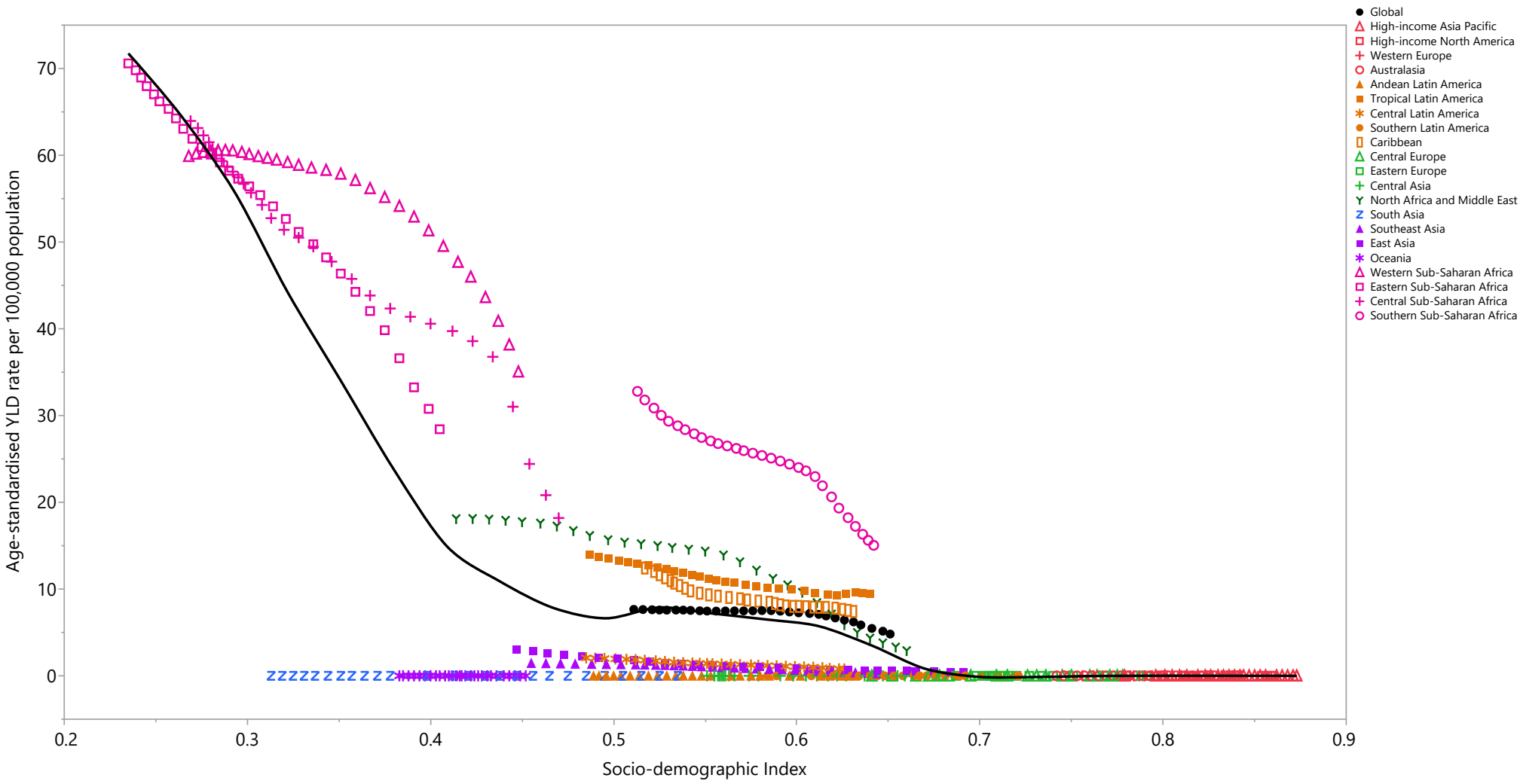

Supplement: Supplementary file 24 — Additional file 24: Figure S19. Age-standardized YLD rates of anemia attributable to schistosomiasis for the 21 Global Burden of Disease regions by Socio-demographic Index, 1990–2019; Expected values based on Socio-demographic Index and disease rates in all locations are shown as the black line. Thirty points are plotted for each GBD region and show the observed age-standardized YLD rates from 1990 to 2019 for that region. YLD = years lived with disability. (Generated from data available from http://ghdx.healthdata.org/gbd-results-tool). [file 13045_2021_1202_MOESM24_ESM.pdf]

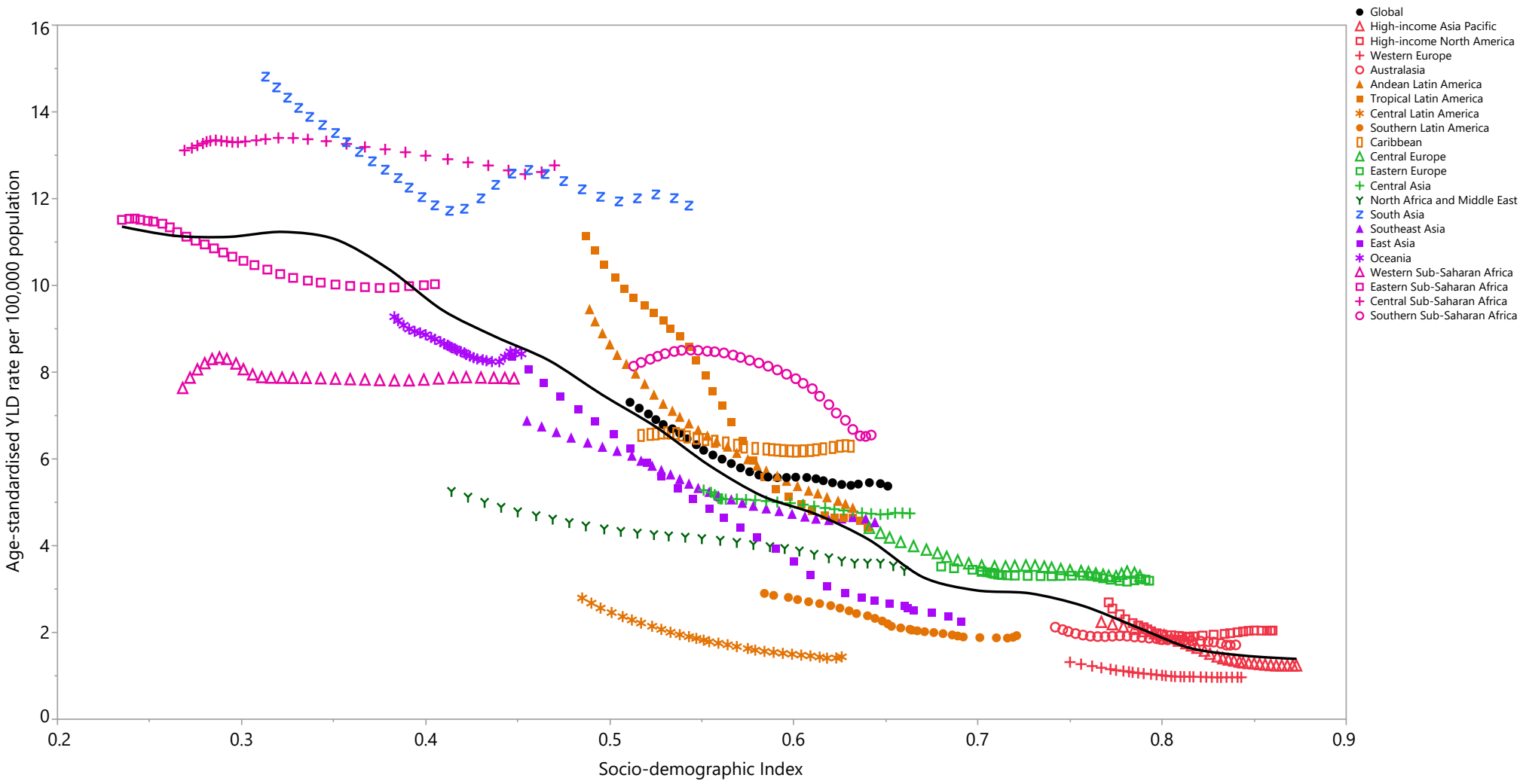

Supplement: Supplementary file 25 — Additional file 25: Figure S20. Age-standardized YLD rates of anemia attributable to upper digestive system diseases for the 21 Global Burden of Disease regions by Socio-demographic Index, 1990–2019; Expected values based on Socio-demographic Index and disease rates in all locations are shown as the black line. Thirty points are plotted for each GBD region and show the observed age-standardized YLD rates from 1990 to 2019 for that region. YLD = years lived with disability. (Generated from data available from http://ghdx.healthdata.org/gbd-results-tool). [file 13045_2021_1202_MOESM25_ESM.pdf]

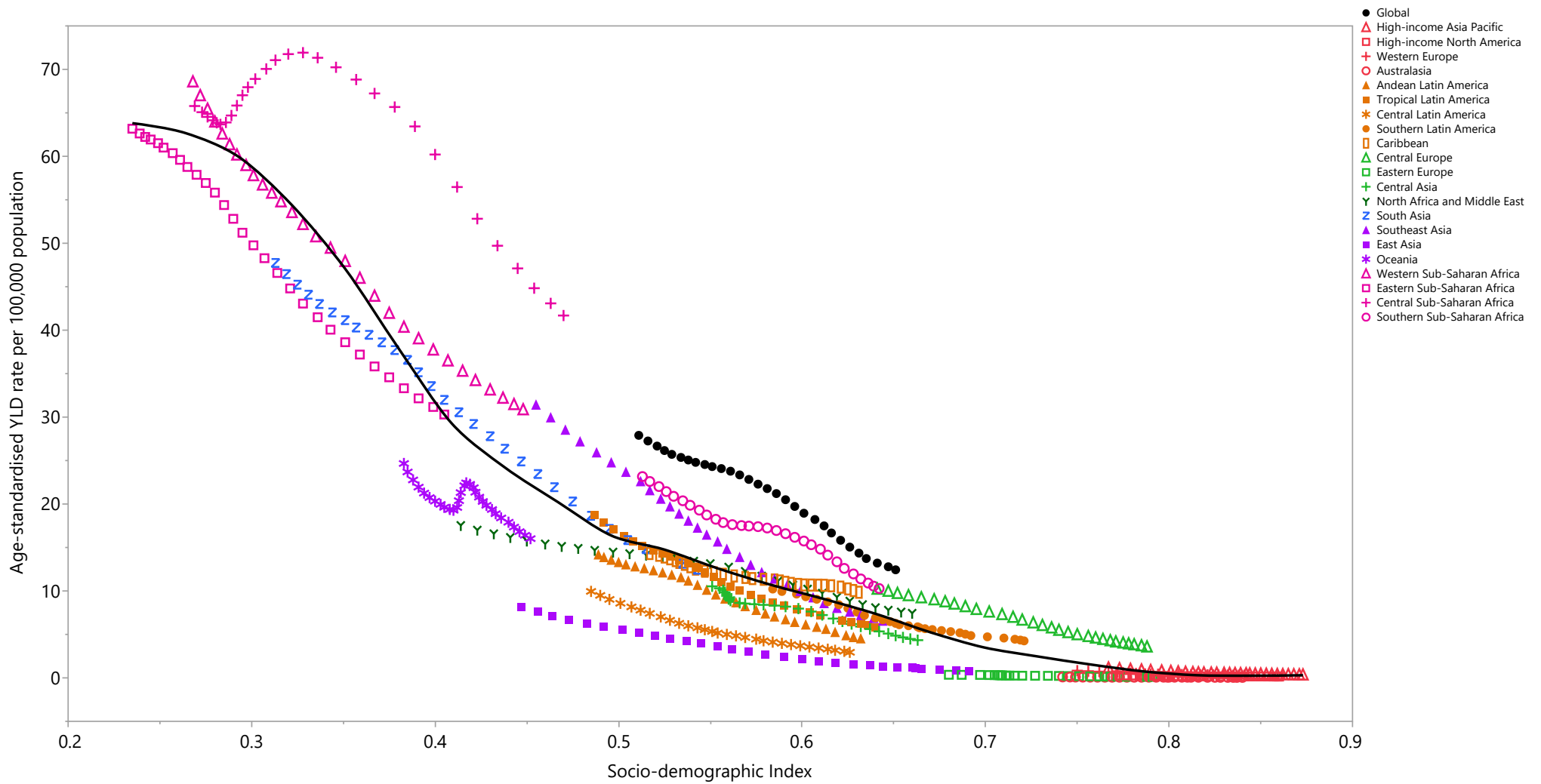

Supplement: Supplementary file 26 — Additional file 26: Figure S21. Age-standardized YLD rates of anemia attributable to vitamin A deficiency for the 21 Global Burden of Disease regions by Socio-demographic Index, 1990–2019; Expected values based on Socio-demographic Index and disease rates in all locations are shown as the black line. Thirty points are plotted for each GBD region and show the observed age-standardized YLD rates from 1990 to 2019 for that region. YLD = years lived with disability. (Generated from data available from http://ghdx.healthdata.org/gbd-results-tool). [file 13045_2021_1202_MOESM26_ESM.pdf]
