# Supplementary material for: Burden of anemia and its underlying causes in 204 countries and territories, 1990–2019: results from the Global Burden of Disease Study 2019
Source: J Hematol Oncol. 2021 Nov 4;14:185. doi: 10.1186/s13045-021-01202-2 (PMC8567696; doi:10.1186/s13045-021-01202-2)
Supplement: Supplementary file 8 — Additional file 8: Table S4. Prevalent cases of anemia in 1990 and 2019 and the percentage change in the age-standardized rates (ASRs) per 100,000, by location (Generated from data available from http://ghdx.healthdata.org/gbd-results-tool). [file 13045_2021_1202_MOESM8_ESM.doc]

| **Additional file 8: Table S4. Prevalent cases of anemia in 1990 and 2019 and the percentage change in the age-standardised rates (ASRs) per 100,000 by location**  **(Generated from data available from http://ghdx.healthdata.org/gbd-results-tool)** | | | | | |
| --- | --- | --- | --- | --- | --- |
|  | **1990** | | **2019** | | **Percentage change in ASRs per 100,000** |
|  | **No (95% UI)** | **ASRs per 100,000 (95% UI)** | **No (95% UI)** | **ASRs per 100,000 (95% UI)** |
| **Global** | **1441197733 (1427406375 , 1454392253)** | **26752 (26508.1 , 26978.6)** | **1761561953 (1744013909 , 1779850189)** | **23176.2 (22943.5 , 23418.6)** | **-13.4 (-14.5 , -12.1)** |
| **High-income North America** | **22403849 (20595793 , 24319566)** | **7684.7 (7039.3 , 8370.9)** | **27820834 (24886580 , 31222390)** | **6920 (6162.3 , 7806.5)** | **-10 (-22 , 4.8)** |
| **Canada** | **1668250 (1412064 , 1956934)** | **5844.2 (4932.3 , 6869.3)** | **1723217 (1400485 , 2093166)** | **4216.6 (3358.2 , 5124.7)** | **-27.9 (-44.7 , -7.2)** |
| **Greenland** | **7474 (6324 , 8676)** | **13768 (11840 , 15872.3)** | **5897 (5028 , 6793)** | **10376.1 (8788.7 , 12048.8)** | **-24.6 (-37.9 , -9.1)** |
| **United States of America** | **20727612 (18933961 , 22641688)** | **7879.2 (7186.8 , 8641.7)** | **26091278 (23126134 , 29382447)** | **7219.6 (6378.6 , 8196.9)** | **-8.4 (-21.4 , 7.9)** |
| **Australasia** | **1845288 (1591653 , 2114956)** | **9418.5 (8055.6 , 10884.9)** | **1942195 (1649693 , 2271715)** | **6765.7 (5571.5 , 8157.6)** | **-28.2 (-43.2 , -8.9)** |
| **Australia** | **1525167 (1270403 , 1790694)** | **9332.6 (7654.2 , 11069.9)** | **1617367 (1339379 , 1938093)** | **6605.3 (5328.7 , 8215.3)** | **-29.2 (-46.1 , -7.1)** |
| **New Zealand** | **320121 (281927 , 364847)** | **9787.7 (8481.2 , 11283.8)** | **324828 (267370 , 385348)** | **7557.4 (6043.7 , 9334.2)** | **-22.8 (-41.4 , -0.5)** |
| **High-income Asia Pacific** | **31294014 (29228325 , 33382953)** | **17659.1 (16428.4 , 18908.4)** | **24772126 (22617793 , 27016500)** | **11037.4 (9947 , 12361.4)** | **-37.5 (-45.1 , -28.6)** |
| **Brunei Darussalam** | **47403 (42639 , 52856)** | **19450.8 (17937.5 , 21233.1)** | **59871 (52018 , 68935)** | **14515.5 (12765.3 , 16640.4)** | **-25.4 (-36 , -13.5)** |
| **Japan** | **19882356 (17834594 , 21864675)** | **15016.2 (13252.7 , 16723.6)** | **18063967 (16047143 , 20240757)** | **10965.6 (9484.1 , 12690.9)** | **-27 (-39.2 , -12)** |
| **Singapore** | **498293 (445105 , 557070)** | **16880.9 (15012.7 , 18857.8)** | **595431 (505920 , 696124)** | **9729.9 (8248.8 , 11343.3)** | **-42.4 (-51.6 , -31.3)** |
| **Republic of Korea** | **10865962 (10220986 , 11537125)** | **24792.1 (23446.3 , 26212.6)** | **6052857 (5196287 , 6966848)** | **10967.6 (9482.9 , 12618.5)** | **-55.8 (-62.4 , -48.1)** |
| **Western Europe** | **28812625 (27043936 , 30737146)** | **7575.6 (7082 , 8110.3)** | **22120972 (20605893 , 23792370)** | **4880.1 (4491.3 , 5308.1)** | **-35.6 (-41.6 , -28.7)** |
| **Andorra** | **3003 (2352 , 3703)** | **5964.5 (4645.4 , 7430.9)** | **3511 (2750 , 4433)** | **4120.5 (3187.7 , 5316.2)** | **-30.9 (-50.1 , -3.5)** |
| **Austria** | **510803 (415245 , 626742)** | **6606.9 (5305.5 , 8218.8)** | **418172 (328800 , 523152)** | **4364.5 (3341.8 , 5614.2)** | **-33.9 (-51.7 , -11.8)** |
| **Belgium** | **587140 (476411 , 714942)** | **6078.3 (4795.2 , 7604.6)** | **444191 (351273 , 550731)** | **3778.9 (2898.6 , 4873)** | **-37.8 (-54.5 , -15.7)** |
| **Cyprus** | **74960 (62078 , 89687)** | **9845.1 (8164.6 , 11737.8)** | **65197 (50680 , 81247)** | **4806.7 (3706.8 , 6096.1)** | **-51.2 (-63.5 , -36.4)** |
| **Denmark** | **400654 (344270 , 465106)** | **8010.3 (6707.8 , 9647)** | **296324 (227441 , 377688)** | **5022.4 (3792.9 , 6617.7)** | **-37.3 (-54 , -14.5)** |
| **Finland** | **396458 (333846 , 468344)** | **7996.3 (6470.2 , 9897.2)** | **260867 (207070 , 323492)** | **4634.4 (3471 , 6156.9)** | **-42 (-58.2 , -18.9)** |
| **France** | **2872774 (2353316 , 3495478)** | **5219.1 (4115 , 6635.8)** | **2023187 (1572056 , 2564677)** | **3118 (2341.9 , 4261.1)** | **-40.3 (-58 , -17)** |
| **Germany** | **5937844 (5011709 , 6998492)** | **7367.4 (6174.6 , 8827.3)** | **4182431 (3377320 , 5124041)** | **4574.2 (3621.9 , 5670)** | **-37.9 (-53.7 , -17.3)** |
| **Greece** | **814223 (678795 , 965188)** | **7714 (6298.8 , 9358)** | **672936 (552346 , 814464)** | **5683.6 (4452.7 , 7118.8)** | **-26.3 (-43.4 , -1.9)** |
| **Iceland** | **12785 (10452 , 15781)** | **5057.2 (4084.1 , 6348.1)** | **13628 (10632 , 17036)** | **3622.1 (2747.8 , 4656.3)** | **-28.4 (-48.5 , -0.5)** |
| **Ireland** | **265428 (215010 , 322627)** | **7595.6 (6120.8 , 9296.9)** | **207162 (160174 , 261462)** | **4094.4 (3104.3 , 5257.8)** | **-46.1 (-60.9 , -28.9)** |
| **Israel** | **489042 (399395 , 589004)** | **9919.7 (8205.5 , 11815.3)** | **599288 (470804 , 739873)** | **6260.3 (4844.8 , 7771.7)** | **-36.9 (-52.3 , -19)** |
| **Italy** | **4215978 (3529556 , 4972197)** | **7488.7 (6078.5 , 8965.4)** | **3144566 (2535101 , 3816770)** | **4809.6 (3678.4 , 6054.6)** | **-35.8 (-51.4 , -14.2)** |
| **Luxembourg** | **28390 (23162 , 33911)** | **7371.8 (5873.3 , 9011.7)** | **27348 (21666 , 34196)** | **4189.4 (3208.2 , 5364.5)** | **-43.2 (-57.3 , -22.6)** |
| **Malta** | **31769 (25618 , 38442)** | **8645.9 (6905.2 , 10556.2)** | **25348 (20505 , 30599)** | **5380.8 (4243.5 , 6805.9)** | **-37.8 (-52.8 , -19.4)** |
| **Monaco** | **1729 (1378 , 2112)** | **5326.4 (4141.9 , 6828.1)** | **1646 (1303 , 2035)** | **3943.9 (2969.4 , 5153.4)** | **-26 (-46.9 , 0.5)** |
| **Netherlands** | **841359 (669547 , 1036847)** | **5691.8 (4435.7 , 7201.1)** | **679601 (533503 , 860983)** | **3837.9 (2908.9 , 5189.5)** | **-32.6 (-52.7 , -6.2)** |
| **Norway** | **319151 (270445 , 373932)** | **7624.2 (6322.4 , 9287.7)** | **263281 (213007 , 331537)** | **4911 (3839.9 , 6415.3)** | **-35.6 (-52 , -13.8)** |
| **Portugal** | **963652 (790792 , 1138144)** | **9973.5 (8076.3 , 11973)** | **553502 (440340 , 690219)** | **5142.6 (3935.9 , 6563.7)** | **-48.4 (-61.5 , -30.3)** |
| **San Marino** | **1349 (1074 , 1674)** | **5844.8 (4590.1 , 7343.1)** | **1471 (1181 , 1843)** | **4183.4 (3220.1 , 5398.8)** | **-28.4 (-46.8 , -1.4)** |
| **Spain** | **3772538 (3179114 , 4498630)** | **10394 (8650.8 , 12475.8)** | **2692687 (2160688 , 3288679)** | **6125.5 (4748.4 , 7768.4)** | **-41.1 (-56.5 , -21.8)** |
| **Sweden** | **542512 (444154 , 653257)** | **6134.9 (4858.6 , 7523.7)** | **491331 (383630 , 609331)** | **4501.1 (3353.8 , 5702.7)** | **-26.6 (-46.4 , -1.1)** |
| **Switzerland** | **375350 (302459 , 450336)** | **5568.2 (4387.5 , 6918.8)** | **355548 (283196 , 440514)** | **3910.9 (2996.5 , 5085.9)** | **-29.8 (-49.7 , -2.2)** |
| **United Kingdom** | **5329811 (4658924 , 6033941)** | **9079.9 (7964.6 , 10349.2)** | **4678466 (4093006 , 5401405)** | **6623.7 (5653.7 , 7887.6)** | **-27.1 (-41 , -9.9)** |
| **Southern Latin America** | **8302071 (7686993 , 8952093)** | **16834.1 (15617.3 , 18146.1)** | **7667450 (6832474 , 8619405)** | **11667.6 (10346.7 , 13149)** | **-30.7 (-39.9 , -20.8)** |
| **Argentina** | **6488925 (5866302 , 7136322)** | **19620.3 (17785.7 , 21577.9)** | **6211342 (5396542 , 7133341)** | **14056 (12183.9 , 16120.4)** | **-28.4 (-39.7 , -16)** |
| **Chile** | **1297605 (1110010 , 1503444)** | **10212.4 (8846.9 , 11704.8)** | **1054292 (884765 , 1249526)** | **5215.3 (4354.8 , 6198.6)** | **-48.9 (-58.9 , -37.1)** |
| **Uruguay** | **515205 (446962 , 587050)** | **16706.1 (14422.9 , 19104.5)** | **401430 (340749 , 467978)** | **11957.3 (10047.5 , 14158.6)** | **-28.4 (-40.7 , -14.1)** |
| **Eastern Europe** | **33426887 (30713405 , 36352568)** | **14023.4 (12907.2 , 15251.6)** | **27467447 (24847685 , 30416012)** | **11042.4 (9962.7 , 12230.3)** | **-21.3 (-30 , -11)** |
| **Belarus** | **1699670 (1483673 , 1929274)** | **15633.6 (13582.5 , 17841.6)** | **1203164 (1035194 , 1378046)** | **11146 (9451.4 , 12899.3)** | **-28.7 (-40.8 , -14.9)** |
| **Estonia** | **251176 (222049 , 283641)** | **15265.7 (13429.3 , 17418.1)** | **158820 (137062 , 185381)** | **10352 (8718.4 , 12258.2)** | **-32.2 (-44.1 , -18.2)** |
| **Latvia** | **434742 (380527 , 488754)** | **15445.3 (13482.6 , 17453.3)** | **270143 (234347 , 307908)** | **12090.3 (10329.2 , 14011.6)** | **-21.7 (-34.8 , -5.9)** |
| **Lithuania** | **561496 (494214 , 632847)** | **14676.8 (12918.6 , 16529.6)** | **397714 (345639 , 452847)** | **12209.5 (10422.8 , 14154.2)** | **-16.8 (-29.7 , -2.2)** |
| **Republic of Moldova** | **1014055 (912138 , 1119677)** | **22709.6 (20519.4 , 25095.1)** | **691923 (617741 , 763955)** | **17089.6 (15370 , 18829.9)** | **-24.7 (-34.6 , -13.9)** |
| **Russian Federation** | **23408267 (20762384 , 26196253)** | **14663.2 (13085.1 , 16431.3)** | **20243324 (17750553 , 23023407)** | **11564.3 (10114.7 , 13181)** | **-21.1 (-32.6 , -6.4)** |
| **Ukraine** | **6057482 (5296791 , 6928386)** | **11024.3 (9619 , 12565.5)** | **4502359 (3877182 , 5265195)** | **8812.9 (7513.6 , 10366.7)** | **-20.1 (-33.7 , -1.6)** |
| **Central Europe** | **20716435 (19619911 , 21863324)** | **17256.4 (16308 , 18224.2)** | **13595798 (12724085 , 14551566)** | **12637.2 (11788 , 13577.9)** | **-26.8 (-32.3 , -21)** |
| **Albania** | **814574 (730477 , 905233)** | **24294.4 (22012.2 , 26750)** | **451925 (418061 , 486450)** | **17020.5 (15652 , 18581.4)** | **-29.9 (-38.5 , -19.9)** |
| **Bosnia and Herzegovina** | **888701 (777169 , 993675)** | **19316.9 (16932.9 , 21603)** | **465009 (397424 , 531753)** | **14618.9 (12374.4 , 16859.6)** | **-24.3 (-35.9 , -9.5)** |
| **Bulgaria** | **1391336 (1195128 , 1583799)** | **16688.2 (14406.6 , 19084.5)** | **952455 (819356 , 1094092)** | **14735.1 (12587.4 , 17133.9)** | **-11.7 (-26.7 , 5.4)** |
| **Croatia** | **597082 (508426 , 693607)** | **12282.5 (10390.3 , 14324.6)** | **393761 (326035 , 470191)** | **9568.9 (7832.2 , 11496.1)** | **-22.1 (-37 , -2.7)** |
| **Czechia** | **1408799 (1196470 , 1617982)** | **14329.6 (12196.7 , 16534)** | **1043811 (879449 , 1215060)** | **10417.2 (8711.2 , 12228.4)** | **-27.3 (-40.5 , -10.5)** |
| **Hungary** | **1342832 (1148263 , 1542798)** | **14002.7 (11911 , 16142.9)** | **907964 (749456 , 1080850)** | **10527 (8655.7 , 12398.6)** | **-24.8 (-39.8 , -7.6)** |
| **Montenegro** | **90110 (77582 , 103670)** | **14380 (12364.5 , 16616.5)** | **75794 (64787 , 88210)** | **12551.5 (10739.8 , 14766.4)** | **-12.7 (-27.4 , 5.7)** |
| **North Macedonia** | **347570 (305307 , 391473)** | **17250.5 (15195.8 , 19330.5)** | **273231 (236794 , 312431)** | **13195.6 (11546 , 14952.1)** | **-23.5 (-34.7 , -8.6)** |
| **Poland** | **6879766 (6038168 , 7761823)** | **18324.7 (16003.1 , 20710.2)** | **4689868 (4015445 , 5465281)** | **12789 (10799.6 , 14969)** | **-30.2 (-41.7 , -16.6)** |
| **Romania** | **4218413 (3726769 , 4753186)** | **18527.9 (16434.8 , 20813.9)** | **2442670 (2091674 , 2788662)** | **13829.8 (11869 , 15845)** | **-25.4 (-37.3 , -11.9)** |
| **Serbia** | **1698062 (1484237 , 1921833)** | **18243.9 (15895 , 20791.1)** | **1107625 (936057 , 1284301)** | **13432.8 (11361.9 , 15640.8)** | **-26.4 (-39.2 , -12.5)** |
| **Slovakia** | **788363 (677720 , 912761)** | **15285.3 (13160.7 , 17815.8)** | **608590 (515297 , 717930)** | **11549.1 (9699.7 , 13618.5)** | **-24.4 (-38 , -7.8)** |
| **Slovenia** | **250827 (214813 , 291859)** | **13050.5 (11104.1 , 15284.1)** | **183096 (153813 , 216408)** | **9290.2 (7689.2 , 11097.5)** | **-28.8 (-42 , -11.2)** |
| **Central Asia** | **20996220 (20320923 , 21720706)** | **30174.8 (29283.3 , 31152.6)** | **24438402 (23313742 , 25576821)** | **26151.5 (24981.8 , 27309.4)** | **-13.3 (-17.7 , -8.8)** |
| **Armenia** | **655243 (595399 , 722954)** | **19448.9 (17755.8 , 21359.9)** | **515424 (461566 , 571553)** | **16732.1 (14975.5 , 18562.8)** | **-14 (-24.7 , -1.8)** |
| **Azerbaijan** | **1967525 (1834205 , 2119412)** | **27166.8 (25434.3 , 29091.4)** | **2327230 (2122748 , 2538418)** | **22410.1 (20501.4 , 24469.3)** | **-17.5 (-25.6 , -8)** |
| **Georgia** | **1251612 (1133580 , 1366350)** | **22703.2 (20609.4 , 24789.1)** | **800094 (732950 , 877325)** | **20574.1 (18576.2 , 22705.6)** | **-9.4 (-19.8 , 2.1)** |
| **Kazakhstan** | **4861167 (4570279 , 5182610)** | **29946.5 (28177.4 , 31773.1)** | **4417047 (4042690 , 4840868)** | **23730 (21679.7 , 25968.2)** | **-20.8 (-28.6 , -12.6)** |
| **Kyrgyzstan** | **1286049 (1181006 , 1400923)** | **28865.6 (26807.3 , 31143.4)** | **1625717 (1469994 , 1778165)** | **24928.7 (22734.2 , 27093.1)** | **-13.6 (-22.4 , -4.1)** |
| **Mongolia** | **605851 (562495 , 651888)** | **28787.4 (27109.8 , 30558.3)** | **694891 (628471 , 762053)** | **20773.9 (18903.3 , 22660.5)** | **-27.8 (-34.9 , -19.8)** |
| **Tajikistan** | **1443818 (1314922 , 1576078)** | **26602.7 (24499.1 , 28778.8)** | **2179278 (1956196 , 2389326)** | **23763 (21655.2 , 25783.1)** | **-10.7 (-19.4 , -1)** |
| **Turkmenistan** | **958670 (864243 , 1055133)** | **26157.3 (23960.1 , 28385.6)** | **1062069 (959912 , 1176480)** | **21037.5 (19117.6 , 23235.3)** | **-19.6 (-28.1 , -10.2)** |
| **Uzbekistan** | **7966284 (7408103 , 8551357)** | **37098 (34931.4 , 39458.7)** | **10816651 (9888660 , 11782137)** | **32042.8 (29394.4 , 34744.5)** | **-13.6 (-22.1 , -5.6)** |
| **Central Latin America** | **24052521 (23217633 , 24971481)** | **15061 (14582.2 , 15567.2)** | **24973205 (24051149 , 26013867)** | **10286.9 (9918.3 , 10716.6)** | **-31.7 (-34.8 , -28.4)** |
| **Colombia** | **5366677 (4700096 , 6125540)** | **17073.7 (15271.1 , 19061.5)** | **4115598 (3502264 , 4788723)** | **8574.9 (7246.9 , 9999.2)** | **-49.8 (-58 , -40.3)** |
| **Costa Rica** | **445963 (389125 , 505021)** | **14830.8 (13093.2 , 16599.8)** | **474035 (400409 , 552293)** | **10145.9 (8552.1 , 11785.5)** | **-31.6 (-43.1 , -17.7)** |
| **El Salvador** | **899018 (782241 , 1012954)** | **17351.6 (15463.6 , 19239.5)** | **730225 (627213 , 835081)** | **11952 (10293.1 , 13642)** | **-31.1 (-42.3 , -18.1)** |
| **Guatemala** | **1900748 (1722738 , 2083152)** | **23674.2 (21733.1 , 25608.6)** | **2944647 (2596051 , 3303304)** | **16917 (15070.3 , 18830.4)** | **-28.5 (-37 , -19.1)** |
| **Honduras** | **1100933 (990159 , 1217156)** | **22851.5 (20892.9 , 24899.6)** | **1549056 (1358045 , 1753401)** | **16389.9 (14575.4 , 18305.9)** | **-28.3 (-37.3 , -17.8)** |
| **Mexico** | **10343418 (10053731 , 10671360)** | **12365.8 (12066.2 , 12693.4)** | **10952571 (10660037 , 11276329)** | **9117.9 (8875.1 , 9381.5)** | **-26.3 (-28.8 , -23.5)** |
| **Nicaragua** | **633168 (556269 , 720306)** | **17849.7 (16072.5 , 19724.8)** | **575293 (485625 , 674313)** | **9524.5 (8141 , 11031.3)** | **-46.6 (-55.2 , -36.4)** |
| **Panama** | **482142 (437283 , 531372)** | **20892.9 (19035.4 , 22775.9)** | **648740 (571040 , 732458)** | **15595.1 (13715.7 , 17638.2)** | **-25.4 (-35.6 , -13.5)** |
| **Venezuela (Bolivarian Republic of)** | **2880454 (2508294 , 3302137)** | **16072 (14275.7 , 18113.9)** | **2983041 (2557930 , 3440137)** | **10750.5 (9260 , 12445.1)** | **-33.1 (-42.9 , -21)** |
| **Andean Latin America** | **9993482 (9411505 , 10614309)** | **25521.7 (24229.8 , 26833.5)** | **9988961 (9351246 , 10696617)** | **15746.5 (14742.1 , 16875.9)** | **-38.3 (-43.3 , -32.5)** |
| **Bolivia (Plurinational State of)** | **2109430 (1961311 , 2269581)** | **32925.3 (30798.4 , 35126.7)** | **3152277 (2863223 , 3470754)** | **25314.1 (23055.7 , 27770.1)** | **-23.1 (-30.9 , -14.2)** |
| **Ecuador** | **1912510 (1683699 , 2147124)** | **18662.3 (16759.5 , 20664.2)** | **1482863 (1309999 , 1657966)** | **8691.6 (7701.3 , 9696.4)** | **-53.4 (-60.3 , -45.8)** |
| **Peru** | **5971542 (5458060 , 6520888)** | **26663.5 (24616.5 , 28718.9)** | **5353821 (4766620 , 5979595)** | **15992.6 (14210.7 , 17964.6)** | **-40 (-47.8 , -31)** |
| **Caribbean** | **9621664 (9189487 , 10054721)** | **27387.8 (26198.1 , 28535.7)** | **11886493 (11346858 , 12405308)** | **25484.8 (24316.8 , 26618.2)** | **-6.9 (-12.4 , -1.5)** |
| **Antigua and Barbuda** | **16338 (14605 , 18026)** | **26999.8 (24196 , 29688.2)** | **19562 (17258 , 21837)** | **22569.8 (19972 , 25267.7)** | **-16.4 (-27.7 , -4.1)** |
| **Barbados** | **52655 (47923 , 57537)** | **20874.5 (18874.8 , 22894.3)** | **49609 (43030 , 56511)** | **16889 (14582.8 , 19385.8)** | **-19.1 (-30.8 , -5.8)** |
| **Belize** | **55862 (49640 , 62335)** | **30225.5 (27487.9 , 33160.5)** | **109678 (97091 , 121998)** | **27464.1 (24563.5 , 30319.1)** | **-9.1 (-19.7 , 2.5)** |
| **Bermuda** | **11748 (10371 , 13322)** | **19839.9 (17502.5 , 22491.9)** | **8595 (7368 , 9887)** | **13166.1 (11216.9 , 15312.5)** | **-33.6 (-44.5 , -21.1)** |
| **Bahamas** | **65144 (58056 , 73059)** | **26085.4 (23350.4 , 28836.8)** | **86164 (76212 , 95764)** | **23258.6 (20611.1 , 25950.8)** | **-10.8 (-22.4 , 1.6)** |
| **Cuba** | **2365414 (2078823 , 2649267)** | **22109 (19580.3 , 24774.8)** | **2037266 (1757373 , 2310480)** | **17704.2 (15233 , 20125.8)** | **-19.9 (-32.5 , -6.4)** |
| **Dominica** | **17487 (15688 , 19284)** | **24230 (21927.6 , 26638.4)** | **15736 (14193 , 17418)** | **22214 (20004.1 , 24600.4)** | **-8.3 (-19.7 , 4.5)** |
| **Dominican Republic** | **1942967 (1752462 , 2150383)** | **27968.4 (25671.7 , 30592.6)** | **2257624 (2017415 , 2489744)** | **20890.6 (18647.8 , 22982.6)** | **-25.3 (-33.7 , -15.2)** |
| **Grenada** | **23690 (22189 , 25210)** | **27113.6 (25553.3 , 28746.3)** | **22497 (19996 , 25176)** | **22672.5 (20251.3 , 25333.8)** | **-16.4 (-25.8 , -6)** |
| **Guyana** | **307649 (287291 , 329460)** | **40630.3 (38369.1 , 43136.2)** | **243004 (224384 , 261935)** | **32097 (29610.3 , 34585.9)** | **-21 (-28.2 , -12.8)** |
| **Haiti** | **2554570 (2377357 , 2728318)** | **40173.4 (37762.5 , 42557.4)** | **4852656 (4516599 , 5204217)** | **38937.6 (36584.7 , 41567.6)** | **-3.1 (-10.9 , 5.7)** |
| **Jamaica** | **612066 (545271 , 678786)** | **26305.5 (23771.1 , 29010.9)** | **650117 (575543 , 724359)** | **23603.6 (20973.6 , 26415.9)** | **-10.3 (-22.1 , 2.1)** |
| **Puerto Rico** | **718992 (628313 , 812963)** | **20167.9 (17635.3 , 22806)** | **538149 (462751 , 615396)** | **14988.6 (12652.6 , 17273.1)** | **-25.7 (-38.3 , -11.2)** |
| **Saint Kitts and Nevis** | **10213 (9018 , 11482)** | **25084.1 (22326.3 , 27972.1)** | **11990 (10598 , 13609)** | **20313.8 (17785.2 , 23066.1)** | **-19 (-30.9 , -5.9)** |
| **Saint Lucia** | **39947 (35747 , 44484)** | **29513.9 (26651.6 , 32431.7)** | **42136 (37974 , 47032)** | **24268.4 (21725.3 , 27382.2)** | **-17.8 (-28.2 , -5.9)** |
| **Saint Vincent and the Grenadines** | **30785 (27623 , 34309)** | **28613.6 (26009.1 , 31339.2)** | **29796 (26836 , 32911)** | **26381.9 (23796.8 , 29234.6)** | **-7.8 (-19.2 , 5.1)** |
| **Suriname** | **117430 (106497 , 130110)** | **30781.9 (28101.4 , 33751.2)** | **157415 (143083 , 173019)** | **27448.5 (24877.4 , 30231.2)** | **-10.8 (-20.7 , 0.3)** |
| **Trinidad and Tobago** | **333050 (297951 , 373080)** | **28080.7 (25382.9 , 31136.6)** | **330541 (294709 , 366776)** | **23860.8 (21131.8 , 26831.3)** | **-15 (-25.5 , -2.8)** |
| **United States Virgin Islands** | **25137 (22359 , 28038)** | **23952.7 (21395.6 , 26573.2)** | **21304 (18750 , 23973)** | **20152.3 (17603.4 , 22902.9)** | **-15.9 (-27.5 , -1.2)** |
| **Tropical Latin America** | **38404299 (35162828 , 41567727)** | **25892 (23851.1 , 27868.6)** | **42933173 (38993225 , 46967298)** | **19063.1 (17259.7 , 20779.5)** | **-26.4 (-34.6 , -17)** |
| **Brazil** | **37471213 (34236293 , 40642281)** | **25953 (23845.8 , 27991.8)** | **41748297 (37773562 , 45832840)** | **19112.9 (17252.4 , 20885.3)** | **-26.4 (-35 , -16.8)** |
| **Paraguay** | **933086 (829154 , 1040885)** | **23426.4 (21149 , 25748)** | **1184877 (1049392 , 1340704)** | **17378.4 (15449.9 , 19556.2)** | **-25.8 (-34.4 , -14.6)** |
| **East Asia** | **242317203 (234616647 , 249609434)** | **20520.1 (19942.1 , 21077)** | **134607357 (126648483 , 143183464)** | **8358.4 (7840.8 , 8888.8)** | **-59.3 (-62.1 , -56.2)** |
| **China** | **234459589 (226833632 , 241620459)** | **20562.9 (19972.9 , 21148.1)** | **126982290 (118843710 , 135345439)** | **8120.2 (7579.4 , 8667.2)** | **-60.5 (-63.4 , -57.4)** |
| **Democratic People's Republic of Korea** | **4989322 (4543953 , 5453156)** | **23329.5 (21357.8 , 25464.7)** | **5274583 (4823221 , 5777255)** | **19930.3 (18265.4 , 21933.5)** | **-14.6 (-24 , -2.6)** |
| **Taiwan (Province of China)** | **2868292 (2489423 , 3280270)** | **14765.4 (12897.2 , 16716.9)** | **2350485 (1985069 , 2715010)** | **8984.7 (7555.4 , 10529.3)** | **-39.1 (-49.4 , -26.8)** |
| **Southeast Asia** | **134462388 (129750492 , 139282802)** | **29935.5 (29012.9 , 30855)** | **133070367 (127670127 , 138618485)** | **20313 (19510.6 , 21123.8)** | **-32.1 (-35.7 , -28.9)** |
| **Cambodia** | **4364151 (4023225 , 4660859)** | **40351.3 (37757.8 , 42651.1)** | **5159370 (4720410 , 5595520)** | **31569.8 (28982.7 , 34140.7)** | **-21.8 (-29.2 , -13.2)** |
| **Indonesia** | **57837558 (53758765 , 61991632)** | **32529.4 (30565.9 , 34545.1)** | **54606815 (50479886 , 58900335)** | **21706.1 (20103.4 , 23356.2)** | **-33.3 (-39.8 , -26.7)** |
| **Lao People's Democratic Republic** | **1463850 (1341281 , 1581507)** | **35493.7 (33015 , 37750.8)** | **1913551 (1745790 , 2095201)** | **27858.6 (25654.4 , 30293.4)** | **-21.5 (-28.4 , -13.3)** |
| **Malaysia** | **4341257 (4118971 , 4564802)** | **25327.3 (24248.5 , 26441.4)** | **5952078 (5275864 , 6591713)** | **19483.9 (17292.3 , 21574.6)** | **-23.1 (-32.3 , -13.9)** |
| **Maldives** | **100342 (93256 , 107592)** | **41128.1 (38503.7 , 43845.7)** | **103124 (91112 , 115909)** | **22160.6 (19495.7 , 24731.8)** | **-46.1 (-52.9 , -39.6)** |
| **Mauritius** | **280533 (253471 , 307949)** | **26833 (24383.8 , 29198.4)** | **260298 (230697 , 288776)** | **20965.9 (18483 , 23471.5)** | **-21.9 (-32.2 , -11.1)** |
| **Myanmar** | **15795272 (14623663 , 17032648)** | **38033.8 (35572.8 , 40610.7)** | **17672993 (16551950 , 18923453)** | **33076 (31017.7 , 35362.6)** | **-13 (-20.5 , -5.2)** |
| **Philippines** | **15716759 (14262350 , 17216275)** | **26163.9 (24176 , 28192.9)** | **18476922 (16602979 , 20426065)** | **17143.4 (15528.8 , 18803.5)** | **-34.5 (-42.1 , -26.6)** |
| **Sri Lanka** | **5201049 (4830982 , 5581787)** | **31068.5 (29013.4 , 33214.3)** | **4167009 (3682558 , 4650982)** | **18627.8 (16444.6 , 20848.3)** | **-40 (-47.9 , -31.8)** |
| **Seychelles** | **18317 (16473 , 20260)** | **25792.4 (23456.5 , 28393.9)** | **18749 (16585 , 21241)** | **18544.2 (16379.1 , 21031.3)** | **-28.1 (-37.9 , -17.3)** |
| **Thailand** | **12041394 (11361118 , 12742433)** | **23452.1 (22247.2 , 24691.4)** | **11177053 (9759224 , 12712593)** | **14914.3 (12983.6 , 17084.1)** | **-36.4 (-45 , -27)** |
| **Timor-Leste** | **270694 (245546 , 296252)** | **33024.1 (30533.3 , 35728.8)** | **343660 (309276 , 379712)** | **25282.3 (23104.2 , 27541.7)** | **-23.4 (-31.2 , -14.3)** |
| **Viet Nam** | **16852481 (15387949 , 18297650)** | **25762.4 (23833.5 , 27627)** | **13044409 (11544438 , 14663046)** | **13889.5 (12346.4 , 15599.3)** | **-46.1 (-53 , -37.8)** |
| **Oceania** | **2159756 (2057867 , 2269190)** | **33535.8 (32159.4 , 34922.9)** | **4117576 (3867196 , 4392826)** | **31291.1 (29606.7 , 33061)** | **-6.7 (-12.8 , -0.2)** |
| **American Samoa** | **11181 (10072 , 12509)** | **24766.3 (22621.8 , 27192.2)** | **11999 (10808 , 13262)** | **22906.9 (20619.2 , 25178.2)** | **-7.5 (-18.4 , 3.5)** |
| **Cook Islands** | **4362 (3857 , 4865)** | **23569.2 (21107.8 , 26044.8)** | **3681 (3251 , 4160)** | **19882.5 (17448.5 , 22644)** | **-15.6 (-26.7 , -2.3)** |
| **Micronesia (Federated States of)** | **34452 (32006 , 37068)** | **33535.9 (31305.6 , 35768.6)** | **27381 (24728 , 30038)** | **28600.5 (26050.5 , 31187.1)** | **-14.7 (-22.7 , -6.1)** |
| **Fiji** | **219493 (204721 , 233699)** | **30560.1 (28764.7 , 32262.8)** | **294180 (271412 , 317625)** | **33042 (30637 , 35561.9)** | **8.1 (-1.7 , 19)** |
| **Guam** | **29969 (26238 , 33639)** | **22574.8 (20037.5 , 25104.3)** | **37639 (33551 , 42049)** | **21852.6 (19470 , 24443)** | **-3.2 (-16 , 10.9)** |
| **Kiribati** | **25585 (23611 , 27666)** | **35449.4 (33032.8 , 38021.6)** | **38296 (35193 , 41515)** | **33717.6 (31281.8 , 36132.1)** | **-4.9 (-12.9 , 4.2)** |
| **Marshall Islands** | **14862 (13901 , 15862)** | **32047.4 (30192.6 , 34067.1)** | **15861 (14375 , 17364)** | **29450.5 (27001.3 , 32006.5)** | **-8.1 (-16.3 , 0.8)** |
| **Nauru** | **2868 (2579 , 3195)** | **28277.4 (25728.8 , 30935)** | **2571 (2300 , 2885)** | **26085.6 (23667 , 28728.3)** | **-7.8 (-17.6 , 3)** |
| **Niue** | **613 (546 , 680)** | **26306.4 (23614.3 , 28976.2)** | **368 (328 , 409)** | **21588.9 (19222.4 , 23978.9)** | **-17.9 (-27.7 , -7.2)** |
| **Northern Mariana Islands** | **9793 (8621 , 11041)** | **22417.5 (19917.8 , 25015.4)** | **8518 (7385 , 9738)** | **20030.3 (17586.8 , 22499.3)** | **-10.6 (-22.9 , 4.6)** |
| **Palau** | **3674 (3290 , 4097)** | **24791.4 (22400.5 , 27389.6)** | **3781 (3359 , 4246)** | **21079.3 (18623.8 , 23762.2)** | **-15 (-25.3 , -2.6)** |
| **Papua New Guinea** | **1456496 (1360718 , 1555162)** | **35301.6 (33337.5 , 37355.2)** | **3121635 (2887344 , 3379049)** | **31496 (29370.2 , 33787.1)** | **-10.8 (-18.6 , -2.5)** |
| **Samoa** | **35195 (31318 , 39101)** | **22480.1 (20394.5 , 24660.3)** | **40093 (35054 , 45420)** | **20251.5 (18050.1 , 22661.2)** | **-9.9 (-22.3 , 3.3)** |
| **Solomon Islands** | **116428 (106215 , 126390)** | **34263.5 (31637.8 , 36935.9)** | **201097 (182136 , 221641)** | **31582.8 (29050.6 , 34360.4)** | **-7.8 (-16.2 , 2.2)** |
| **Tokelau** | **532 (485 , 580)** | **30941.6 (28415.5 , 33341.9)** | **355 (318 , 393)** | **25051.1 (22564.9 , 27582.3)** | **-19 (-28.6 , -9.1)** |
| **Tonga** | **24545 (21996 , 26979)** | **27695.8 (25391.4 , 30072.3)** | **24393 (21917 , 27131)** | **25213.7 (22831.2 , 27839.5)** | **-9 (-18.9 , 2.8)** |
| **Tuvalu** | **3085 (2836 , 3341)** | **33062.4 (30603.7 , 35521.9)** | **3218 (2894 , 3536)** | **28145.8 (25511.2 , 30794)** | **-14.9 (-23.4 , -5.7)** |
| **Vanuatu** | **47107 (43512 , 51138)** | **32940.9 (30919.8 , 35147)** | **88032 (80805 , 95699)** | **31912.8 (29505.1 , 34406.7)** | **-3.1 (-12.2 , 6.4)** |
| **North Africa and Middle East** | **88326772 (85497085 , 91386150)** | **25504.3 (24785.9 , 26262.7)** | **107540095 (103637597 , 111903977)** | **18019.7 (17398.5 , 18738.9)** | **-29.3 (-32.4 , -26.3)** |
| **Afghanistan** | **2784618 (2517038 , 3056403)** | **23670.3 (21762.9 , 25448.9)** | **7107753 (6370295 , 7953585)** | **18531.7 (16982.8 , 20087.8)** | **-21.7 (-29.9 , -12.2)** |
| **Algeria** | **6115828 (5391712 , 6893793)** | **24211.8 (21730.4 , 26627.2)** | **7051161 (6103648 , 8006699)** | **16925.4 (14685.7 , 19218.5)** | **-30.1 (-39.7 , -18.8)** |
| **Bahrain** | **133643 (119813 , 147406)** | **26597 (24077 , 29086.6)** | **239827 (205215 , 274083)** | **17475.5 (15177 , 19923.7)** | **-34.3 (-44.1 , -22.8)** |
| **Egypt** | **16061329 (14595921 , 17749534)** | **28476.8 (26158.5 , 31070)** | **19237611 (16957884 , 21577324)** | **19546.2 (17411.3 , 21794.8)** | **-31.4 (-40.2 , -21.7)** |
| **Iran (Islamic Republic of)** | **12153203 (10767608 , 13488555)** | **21545.3 (19573.2 , 23487.9)** | **9668112 (8374103 , 11105046)** | **11902 (10326.9 , 13612.4)** | **-44.8 (-52.9 , -36.1)** |
| **Iraq** | **4184187 (3746038 , 4686136)** | **24144.1 (22047.9 , 26514.8)** | **7016802 (6117552 , 8012287)** | **17410.1 (15369.5 , 19600.5)** | **-27.9 (-36.6 , -17.1)** |
| **Jordan** | **876412 (785008 , 980833)** | **24106.8 (22037.8 , 26378.7)** | **1922828 (1727112 , 2140690)** | **17004.6 (15420.4 , 18824.2)** | **-29.5 (-38 , -19.7)** |
| **Kuwait** | **320611 (297106 , 347527)** | **17987.4 (16749.9 , 19296.3)** | **585123 (510813 , 662313)** | **13456.6 (11790.1 , 15118.6)** | **-25.2 (-36.2 , -13.3)** |
| **Lebanon** | **728506 (655517 , 804487)** | **21853.2 (19901.8 , 23891.2)** | **562777 (467683 , 662470)** | **10838.9 (9011 , 12726.8)** | **-50.4 (-59 , -39.7)** |
| **Libya** | **998609 (878114 , 1139564)** | **23285.2 (20931.6 , 25943.5)** | **1195147 (1041970 , 1358345)** | **18607.4 (16312.3 , 20970.9)** | **-20.1 (-30.7 , -8.4)** |
| **Morocco** | **7177343 (6463396 , 7992791)** | **28436.2 (26064.2 , 31133.3)** | **7378310 (6615236 , 8298854)** | **20971.3 (18780.6 , 23566.6)** | **-26.3 (-35.1 , -16.6)** |
| **Palestine** | **489432 (432964 , 551504)** | **23687.1 (21526.5 , 25990.9)** | **821510 (725787 , 926560)** | **17114.7 (15412.7 , 19046.4)** | **-27.7 (-37.1 , -17.4)** |
| **Oman** | **708197 (658621 , 761695)** | **35455.2 (33276.3 , 38002.7)** | **869228 (736534 , 1002749)** | **22360.5 (19532.9 , 25358.3)** | **-36.9 (-45.8 , -27.5)** |
| **Qatar** | **82572 (71975 , 94992)** | **20059 (17889.2 , 22590.3)** | **250063 (202556 , 309042)** | **10102.1 (8276.7 , 12112.8)** | **-49.6 (-58.9 , -39.7)** |
| **Saudi Arabia** | **2419102 (2170840 , 2685423)** | **16252.5 (14636.8 , 17847.2)** | **2975434 (2431374 , 3599959)** | **9572.7 (7990.9 , 11244.9)** | **-41.1 (-51 , -30.1)** |
| **Sudan** | **6976364 (6366011 , 7595340)** | **32921.5 (30455.3 , 35365.8)** | **10975219 (9915743 , 12086284)** | **26370.6 (24068 , 28766.1)** | **-19.9 (-27.7 , -10.5)** |
| **Syrian Arab Republic** | **3404295 (2995451 , 3825830)** | **25877.4 (23352.7 , 28383.4)** | **2592709 (2247528 , 2948910)** | **18767.2 (16435.5 , 21189)** | **-27.5 (-37.8 , -16.6)** |
| **Tunisia** | **1524270 (1393237 , 1674205)** | **18234.9 (16708.9 , 19844.8)** | **1341042 (1154205 , 1554822)** | **11748.8 (10059.7 , 13560.1)** | **-35.6 (-45.9 , -24.5)** |
| **Turkey** | **15560682 (14012412 , 17165580)** | **26128.7 (23761.7 , 28412.2)** | **12059740 (10541030 , 13568725)** | **15254.7 (13303.8 , 17243.3)** | **-41.6 (-50 , -32.5)** |
| **United Arab Emirates** | **377940 (326752 , 434123)** | **21166.6 (18721.4 , 23481.1)** | **1234299 (1015995 , 1510422)** | **15447.2 (13210.5 , 17762.2)** | **-27 (-38.2 , -14.7)** |
| **Yemen** | **5190222 (4849286 , 5542135)** | **35226.5 (33279.6 , 37462.5)** | **12346142 (11600265 , 13077498)** | **37237.3 (35166.4 , 39232.1)** | **5.7 (-2.1 , 14)** |
| **South Asia** | **517489379 (510460894 , 524063402)** | **47570.2 (47039.5 , 48077.6)** | **731143119 (720084143 , 741777108)** | **41646.1 (41034.3 , 42208.3)** | **-12.5 (-14 , -11)** |
| **Bangladesh** | **45235340 (42018066 , 48407211)** | **41819.1 (39344.6 , 44199.2)** | **49204939 (45139600 , 53684311)** | **32109.9 (29643.2 , 34875.3)** | **-23.2 (-30.4 , -16)** |
| **Bhutan** | **336997 (322363 , 352643)** | **52129.2 (50074.4 , 54217.8)** | **323405 (305859 , 342839)** | **45299.8 (42925.3 , 47776.3)** | **-13.1 (-19 , -7.2)** |
| **India** | **414526684 (409684522 , 419352625)** | **49016.1 (48521.6 , 49500.7)** | **583387442 (576471775 , 590135701)** | **43126 (42648.2 , 43596.3)** | **-12 (-13.3 , -10.7)** |
| **Nepal** | **8079741 (7481886 , 8660377)** | **42209.9 (39726.9 , 44720.7)** | **10250015 (9477623 , 11097498)** | **35139.2 (32812.9 , 37860.8)** | **-16.8 (-24 , -8.6)** |
| **Pakistan** | **49310617 (45885761 , 52593222)** | **43228.9 (40825.4 , 45652)** | **87977318 (81235537 , 94971240)** | **40031.4 (37483 , 42635.1)** | **-7.4 (-14 , 0.3)** |
| **Southern Sub-Saharan Africa** | **12904624 (12018565 , 13785907)** | **24631.4 (23248.8 , 26109.9)** | **16185363 (15329927 , 17101430)** | **20967.7 (19936.3 , 22072.8)** | **-14.9 (-21.3 , -7.8)** |
| **Botswana** | **422902 (392556 , 458439)** | **30658.8 (28784 , 32746)** | **538243 (478063 , 597964)** | **23201.3 (20761.3 , 25664.8)** | **-24.3 (-32.2 , -15.8)** |
| **Lesotho** | **483699 (442844 , 524058)** | **25874.2 (23941.1 , 27785.8)** | **499686 (463620 , 539025)** | **24540.4 (22943.7 , 26260.9)** | **-5.2 (-13.6 , 5)** |
| **Namibia** | **462660 (424129 , 499502)** | **31125.2 (28841 , 33258.5)** | **547758 (499148 , 595925)** | **22473.1 (20667 , 24239.8)** | **-27.8 (-34.7 , -20.3)** |
| **South Africa** | **8346764 (7536011 , 9201690)** | **23026.3 (21044.1 , 25131.1)** | **10228832 (9479375 , 10992838)** | **18632.1 (17318.1 , 20029.8)** | **-19.1 (-27.6 , -9.2)** |
| **Eswatini** | **210282 (187636 , 233610)** | **25078.4 (22814.5 , 27441.8)** | **246093 (219956 , 274979)** | **22064.8 (20059.1 , 24339.8)** | **-12 (-21.7 , -0.4)** |
| **Zimbabwe** | **2978317 (2676867 , 3284632)** | **28132.9 (25823.6 , 30343.1)** | **4124751 (3778514 , 4483270)** | **27400.6 (25455 , 29366.7)** | **-2.6 (-12.3 , 8.3)** |
| **Western Sub-Saharan Africa** | **89221652 (86947344 , 91425721)** | **40960.7 (39903.4 , 42019.9)** | **205607201 (199087872 , 212195550)** | **40977 (39789.3 , 42154.8)** | **0 (-3.3 , 3.6)** |
| **Benin** | **2035170 (1928495 , 2155526)** | **36993.7 (35270.4 , 38798.5)** | **5076176 (4705822 , 5455483)** | **36318 (33944.7 , 38809.8)** | **-1.8 (-9.2 , 6.4)** |
| **Burkina Faso** | **5208482 (4931657 , 5480060)** | **46911.1 (44550.5 , 49265.8)** | **12011357 (11358348 , 12592886)** | **46117.2 (43640.7 , 48319.2)** | **-1.7 (-7.9 , 5.3)** |
| **Cameroon** | **3526381 (3279387 , 3774344)** | **30712.6 (28852.8 , 32669.6)** | **9472125 (8733777 , 10264576)** | **31049.4 (28951.8 , 33241.1)** | **1.1 (-7.8 , 10.5)** |
| **Cabo Verde** | **124962 (113554 , 136806)** | **31797.9 (29284.8 , 34480.8)** | **145815 (129453 , 160838)** | **26094.2 (23220.2 , 28657.7)** | **-17.9 (-27.5 , -8.3)** |
| **Chad** | **2692032 (2514092 , 2883453)** | **39235.2 (36847.5 , 41821.4)** | **7329108 (6805844 , 7889752)** | **38850.8 (36296.8 , 41612.1)** | **-1 (-8.9 , 7.8)** |
| **CÃ´te d'Ivoire** | **6398846 (6093321 , 6712727)** | **47728.9 (45549.9 , 49778.2)** | **12047622 (11379532 , 12730342)** | **43082.3 (40804.8 , 45330.2)** | **-9.7 (-15.4 , -3.4)** |
| **Gambia** | **527051 (498245 , 555692)** | **45468.3 (43123.4 , 47820.1)** | **1074790 (1009137 , 1140621)** | **43787.4 (41246.2 , 46186.2)** | **-3.7 (-10.5 , 3.1)** |
| **Ghana** | **6636201 (6183559 , 7095534)** | **40838.9 (38239.2 , 43336.4)** | **12466969 (11542551 , 13483467)** | **38491.4 (35827 , 41348.2)** | **-5.7 (-14.1 , 3.3)** |
| **Guinea** | **2720500 (2582923 , 2872611)** | **40150.4 (38140.9 , 42139.8)** | **5525035 (5187901 , 5878472)** | **40112.4 (37841.3 , 42392.3)** | **-0.1 (-7.3 , 7.6)** |
| **Guinea-Bissau** | **450903 (418472 , 482102)** | **40241.7 (37820.2 , 42788)** | **789068 (724090 , 851461)** | **38522.2 (35781.8 , 41175.3)** | **-4.3 (-11.9 , 3.7)** |
| **Liberia** | **928064 (871903 , 987926)** | **43426.9 (41066.8 , 45937.1)** | **1863905 (1718710 , 2015527)** | **37269 (34520.6 , 39859)** | **-14.2 (-21 , -6.5)** |
| **Mali** | **4556248 (4320631 , 4794377)** | **47441.5 (44984.8 , 49787.9)** | **11670948 (11040827 , 12259123)** | **46890.1 (44301.1 , 49389.8)** | **-1.2 (-8.3 , 5.6)** |
| **Mauritania** | **889877 (826352 , 955176)** | **38464.1 (35882.4 , 41101.6)** | **1514211 (1384631 , 1641352)** | **35039.1 (32319.7 , 37687)** | **-8.9 (-17.5 , -0.4)** |
| **Niger** | **4201234 (3931795 , 4444599)** | **45502.4 (43034.4 , 47978.7)** | **11441400 (10740469 , 12194641)** | **41312.3 (39007.9 , 43783.2)** | **-9.2 (-15.8 , -2.3)** |
| **Nigeria** | **40522157 (38570517 , 42545724)** | **39305.8 (37212.3 , 41504)** | **98694088 (92714741 , 104949684)** | **41577.3 (39265.3 , 43966.1)** | **5.8 (-1.5 , 13.3)** |
| **Sao Tome and Principe** | **50761 (45969 , 55536)** | **37697.4 (34851.8 , 40677.7)** | **67830 (60876 , 75210)** | **31975.4 (29216.3 , 35027)** | **-15.2 (-24.7 , -5)** |
| **Senegal** | **4019275 (3778862 , 4248857)** | **47092.4 (44690.8 , 49650.5)** | **6918401 (6521686 , 7304901)** | **42826.3 (40590.4 , 45036.6)** | **-9.1 (-15 , -2.5)** |
| **Sierra Leone** | **1830215 (1721183 , 1933745)** | **45695.3 (43079.2 , 48309)** | **3965778 (3762305 , 4178835)** | **44068.7 (41987.3 , 46203.2)** | **-3.6 (-10.2 , 3.3)** |
| **Togo** | **1900304 (1788015 , 2015733)** | **44241.5 (41552 , 46887.7)** | **3529700 (3336354 , 3725317)** | **41216.3 (39112.6 , 43340.9)** | **-6.8 (-13.3 , 0)** |
| **Eastern Sub-Saharan Africa** | **78781361 (77456951 , 80153343)** | **38935.4 (38313.5 , 39524.3)** | **138926032 (135954864 , 142247277)** | **32699.5 (32069.9 , 33345.9)** | **-16 (-17.9 , -14)** |
| **Burundi** | **2063312 (1895379 , 2230427)** | **34120.3 (31875.8 , 36459.3)** | **3824848 (3539748 , 4132320)** | **29698.1 (27802.9 , 31676.6)** | **-13 (-20.6 , -4.4)** |
| **Comoros** | **197800 (183114 , 212377)** | **40882.4 (38419 , 43488.6)** | **238261 (217707 , 260150)** | **33800.1 (31130.3 , 36647.8)** | **-17.3 (-24.3 , -9.9)** |
| **Djibouti** | **190500 (172886 , 206631)** | **37627.5 (34725.1 , 40281.4)** | **371005 (334619 , 409590)** | **31061.9 (28366.9 , 33873.1)** | **-17.4 (-25.5 , -8.6)** |
| **Eritrea** | **1328531 (1226077 , 1421787)** | **42584.1 (39862.7 , 45123.7)** | **2341533 (2128580 , 2545821)** | **34974.3 (32298.1 , 37681.6)** | **-17.9 (-24.6 , -10.2)** |
| **Ethiopia** | **17934743 (17245202 , 18639600)** | **31503.7 (30463.9 , 32547.1)** | **29562031 (28325127 , 30930280)** | **25465.3 (24533.9 , 26498)** | **-19.2 (-22.7 , -15.2)** |
| **Kenya** | **6565923 (6448563 , 6681774)** | **27517.1 (27122.6 , 27867.3)** | **11902081 (11681639 , 12117727)** | **24828.4 (24426.6 , 25216.8)** | **-9.8 (-11.5 , -8)** |
| **Madagascar** | **5052136 (4674454 , 5428553)** | **40401.3 (37817.8 , 42981.1)** | **9145316 (8384242 , 9963341)** | **34819 (32335.3 , 37494.6)** | **-13.8 (-21.4 , -5.1)** |
| **Malawi** | **4765730 (4496916 , 5038072)** | **46967.3 (44517 , 49398.4)** | **7564199 (7002602 , 8126377)** | **39902.4 (37271.6 , 42463.1)** | **-15 (-21.7 , -8.1)** |
| **Mozambique** | **6313137 (5937881 , 6671005)** | **45594.3 (43136.4 , 47851.1)** | **12542821 (11754837 , 13403199)** | **40189.6 (37872.4 , 42742.1)** | **-11.9 (-18.2 , -5.2)** |
| **Rwanda** | **2357549 (2180059 , 2558179)** | **33158.3 (30949.6 , 35635.2)** | **3000771 (2673921 , 3325083)** | **24855.5 (22591.3 , 27196.4)** | **-25 (-33.1 , -16.2)** |
| **Somalia** | **3413902 (3182637 , 3636894)** | **46818.2 (44374.8 , 49268.8)** | **8407710 (7796188 , 9024335)** | **40618.7 (38166.9 , 43041.5)** | **-13.2 (-19.8 , -6.5)** |
| **South Sudan** | **2520208 (2340642 , 2691911)** | **41395 (38821.3 , 43850.7)** | **3629985 (3328414 , 3940826)** | **38017 (35425.4 , 40774.2)** | **-8.2 (-15.6 , 0.5)** |
| **United Republic of Tanzania** | **14912822 (14218419 , 15622867)** | **53900.9 (51533.5 , 56149)** | **24323129 (22463624 , 26368674)** | **40903.3 (38174.7 , 43626.6)** | **-24.1 (-30.3 , -17.7)** |
| **Uganda** | **7077748 (6599252 , 7580944)** | **37854.3 (35629.4 , 40136.6)** | **12795028 (11761987 , 13952892)** | **30154.7 (28003.7 , 32353.8)** | **-20.3 (-27.2 , -12.9)** |
| **Zambia** | **4029508 (3820687 , 4232827)** | **48122.8 (45935.6 , 50055.8)** | **9166251 (8616556 , 9677760)** | **49327.1 (46838.5 , 51700.1)** | **2.5 (-3.7 , 9.3)** |
| **Central Sub-Saharan Africa** | **25665245 (24472259 , 26801865)** | **44195.3 (42505.8 , 45846.3)** | **50757787 (48119655 , 53363924)** | **36861.4 (35218.3 , 38434.2)** | **-16.6 (-21.1 , -11.8)** |
| **Angola** | **3344849 (3067006 , 3630443)** | **33370.2 (30933 , 35727.4)** | **9782704 (8823788 , 10790556)** | **31127.3 (28416.6 , 33852.2)** | **-6.7 (-16.5 , 3.5)** |
| **Central African Republic** | **1268439 (1173726 , 1356908)** | **41396.7 (38776.9 , 43846.3)** | **2268660 (2115031 , 2429161)** | **40063.9 (37785.8 , 42567.4)** | **-3.2 (-10.3 , 4.9)** |
| **Congo** | **1065861 (981827 , 1144176)** | **42443.1 (39856.4 , 44957.7)** | **2123948 (1966038 , 2287220)** | **39590.1 (36985.3 , 42318.7)** | **-6.7 (-14.2 , 2.1)** |
| **Democratic Republic of the Congo** | **19289027 (18091992 , 20380110)** | **47330.2 (45012.1 , 49632.2)** | **35304768 (32961725 , 37541275)** | **38348 (36132.3 , 40445.7)** | **-19 (-24.9 , -12.8)** |
| **Equatorial Guinea** | **209215 (195462 , 222993)** | **46068.2 (43479.6 , 48674.3)** | **507496 (455846 , 560428)** | **35067.1 (32175.1 , 38081)** | **-23.9 (-30.7 , -16.9)** |
| **Gabon** | **487854 (456190 , 522589)** | **47256.4 (44563 , 50116.8)** | **770210 (725671 , 817445)** | **43626.2 (41258.5 , 46107.6)** | **-7.7 (-14.8 , -0.5)** |
