# Supplementary material for: Burden of anemia and its underlying causes in 204 countries and territories, 1990–2019: results from the Global Burden of Disease Study 2019
Source: J Hematol Oncol. 2021 Nov 4;14:185. doi: 10.1186/s13045-021-01202-2 (PMC8567696; doi:10.1186/s13045-021-01202-2)
Supplement: Supplementary file 9 — Additional file 9: Table S5. Years lived with disability (YLDs) due to anemia in 1990 and 2019 and the percentage change in the age-standardized rates (ASRs) per 100,000, by location (Generated from data available from http://ghdx.healthdata.org/gbd-results-tool). [file 13045_2021_1202_MOESM9_ESM.doc]

| **Additional file 9: Table S5. YLDs due to anemia in 1990 and 2019 and the percentage change in the age-standardised rates (ASRs) per 100,000 by location**  **(Generated from data available from http://ghdx.healthdata.org/gbd-results-tool)** | | | | | |
| --- | --- | --- | --- | --- | --- |
|  | **1990** | | **2019** | | **Percentage change in ASRs per 100,000** |
|  | **No (95% UI)** | **ASRs per 100,000 (95% UI)** | **No (95% UI)** | **ASRs per 100,000 (95% UI)** |
| **Global** | **45434224 (30161499 , 65937847)** | **828 (550.5 , 1200)** | **50296485 (33399077 , 73432754)** | **672.4 (447.2 , 981.5)** | **-18.8 (-20.8 , -16.9)** |
| **High-income North America** | **359204 (222789 , 554376)** | **124.5 (77.3 , 191.1)** | **443997 (273887 , 695503)** | **112.9 (68.4 , 177.5)** | **-9.3 (-24.4 , 10.6)** |
| **Canada** | **26593 (15978 , 43437)** | **94.9 (57.4 , 153)** | **25027 (14595 , 40598)** | **63.7 (37 , 101.9)** | **-32.8 (-51.7 , -9.2)** |
| **Greenland** | **153 (93 , 242)** | **282.9 (173.7 , 441.5)** | **107 (65 , 167)** | **193.5 (115.9 , 295.7)** | **-31.6 (-46.2 , -11.8)** |
| **United States of America** | **332450 (204344 , 510889)** | **127.7 (78.2 , 196)** | **418855 (257649 , 657344)** | **118.4 (72 , 186.1)** | **-7.3 (-24.1 , 14.5)** |
| **Australasia** | **30897 (19046 , 49770)** | **159.7 (98.7 , 256.2)** | **29143 (17242 , 47395)** | **102.8 (59.1 , 166.9)** | **-35.6 (-52.5 , -13.1)** |
| **Australia** | **25216 (15377 , 41353)** | **155.9 (94.5 , 254.9)** | **23910 (13845 , 39511)** | **98.5 (55.2 , 163.3)** | **-36.8 (-55.6 , -11.1)** |
| **New Zealand** | **5681 (3520 , 8939)** | **177.1 (109.1 , 277)** | **5233 (3032 , 8433)** | **124.5 (70.3 , 201.5)** | **-29.7 (-52 , 0.6)** |
| **High-income Asia Pacific** | **682181 (436230 , 1029532)** | **392.2 (252 , 590.5)** | **413741 (259883 , 642035)** | **194.6 (120.4 , 301)** | **-50.4 (-58.1 , -41.9)** |
| **Brunei Darussalam** | **1136 (720 , 1720)** | **456.6 (294 , 681.7)** | **1201 (746 , 1897)** | **293.7 (184.3 , 462.7)** | **-35.7 (-48.1 , -21.9)** |
| **Japan** | **382624 (238275 , 585957)** | **294.5 (183 , 455)** | **296285 (181435 , 461923)** | **186.6 (113.4 , 291.1)** | **-36.7 (-50.1 , -20.2)** |
| **Singapore** | **10175 (6306 , 15229)** | **349.6 (216.5 , 525.2)** | **9505 (5722 , 15320)** | **160.2 (94.9 , 255.6)** | **-54.2 (-64 , -42.4)** |
| **Republic of Korea** | **288247 (185630 , 431358)** | **656.5 (423.1 , 982.2)** | **106751 (66186 , 165975)** | **208.5 (127.4 , 333)** | **-68.2 (-74.4 , -61.1)** |
| **Western Europe** | **443251 (274294 , 697421)** | **120.9 (74.7 , 189.9)** | **306588 (191681 , 481932)** | **70.8 (44.3 , 112.5)** | **-41.5 (-49.1 , -33.7)** |
| **Andorra** | **40 (22 , 67)** | **83.9 (47.5 , 139.2)** | **45 (26 , 79)** | **54 (29.8 , 95.8)** | **-35.6 (-57.1 , -4.6)** |
| **Austria** | **7396 (4209 , 11931)** | **98.2 (55.8 , 165.2)** | **5470 (3109 , 8721)** | **58.3 (31.7 , 98)** | **-40.6 (-60.7 , -14.4)** |
| **Belgium** | **8373 (4915 , 13848)** | **89.4 (51.5 , 152.2)** | **5769 (3272 , 9651)** | **50 (27.5 , 85.3)** | **-44.1 (-62.4 , -15.4)** |
| **Cyprus** | **1192 (694 , 1885)** | **158.2 (91.4 , 248.4)** | **848 (484 , 1489)** | **64.7 (37.3 , 115.9)** | **-59.1 (-71.8 , -41.1)** |
| **Denmark** | **5043 (2951 , 8085)** | **106.3 (59.6 , 177.2)** | **3384 (1897 , 5693)** | **59.4 (31.8 , 103.5)** | **-44.1 (-61.7 , -14.9)** |
| **Finland** | **6141 (3723 , 9721)** | **122.7 (71.9 , 193.8)** | **3474 (1955 , 5630)** | **61.9 (33 , 104.2)** | **-49.6 (-65.2 , -26.6)** |
| **France** | **40096 (23022 , 64460)** | **75.7 (42 , 126.1)** | **25273 (14372 , 42393)** | **40.5 (21.9 , 70.3)** | **-46.5 (-65.8 , -15.7)** |
| **Germany** | **87959 (50392 , 147207)** | **111.9 (64.9 , 189.8)** | **55223 (31543 , 91662)** | **61.9 (34.6 , 103.8)** | **-44.7 (-61.9 , -22)** |
| **Greece** | **12443 (7318 , 19876)** | **117.9 (66.2 , 189.6)** | **9676 (5818 , 15302)** | **80.5 (46 , 131.6)** | **-31.7 (-51.3 , -3.9)** |
| **Iceland** | **175 (97 , 289)** | **70.1 (38.8 , 117.7)** | **166 (92 , 278)** | **45.7 (24.6 , 77.2)** | **-34.8 (-57 , -2.3)** |
| **Ireland** | **3881 (2249 , 6360)** | **111.4 (64.5 , 181.9)** | **2628 (1490 , 4295)** | **52.1 (28.8 , 88.3)** | **-53.2 (-67.9 , -32.9)** |
| **Israel** | **8215 (4631 , 13724)** | **165 (93.5 , 274.7)** | **8782 (4921 , 14149)** | **91.9 (51.3 , 148.3)** | **-44.3 (-61.4 , -20.8)** |
| **Italy** | **63010 (38115 , 101047)** | **115.1 (67.2 , 181)** | **43159 (24767 , 69804)** | **66.2 (37.3 , 108.7)** | **-42.5 (-59.4 , -17.2)** |
| **Luxembourg** | **417 (249 , 674)** | **111.4 (64.7 , 184.3)** | **355 (195 , 589)** | **55.6 (30.9 , 95.7)** | **-50.1 (-64.9 , -25.4)** |
| **Malta** | **484 (274 , 781)** | **133.5 (74.6 , 216.6)** | **336 (199 , 563)** | **73.5 (41.2 , 122.3)** | **-44.9 (-61.4 , -21.6)** |
| **Monaco** | **24 (13 , 39)** | **74.9 (42.2 , 129.1)** | **21 (12 , 36)** | **51.9 (28 , 90)** | **-30.7 (-53.8 , 1.9)** |
| **Netherlands** | **11386 (6321 , 18764)** | **77.5 (41.5 , 129.8)** | **8536 (4730 , 14221)** | **47.7 (25.6 , 82.2)** | **-38.5 (-58.4 , -12)** |
| **Norway** | **4338 (2554 , 7252)** | **107.5 (61.7 , 181.8)** | **3217 (1826 , 5551)** | **62.2 (33.9 , 109.8)** | **-42.2 (-61.2 , -14.9)** |
| **Portugal** | **15596 (9316 , 25310)** | **165.6 (97.4 , 271.8)** | **7554 (4180 , 12632)** | **71.8 (38.4 , 126.1)** | **-56.6 (-70.3 , -34.8)** |
| **San Marino** | **19 (10 , 30)** | **82.9 (45.3 , 135.3)** | **19 (11 , 32)** | **55.4 (30.1 , 92.2)** | **-33.1 (-53.7 , 0.2)** |
| **Spain** | **60469 (35294 , 101540)** | **172.4 (99.2 , 284.9)** | **36508 (20249 , 62011)** | **87.5 (46.8 , 154.2)** | **-49.2 (-66.3 , -22.2)** |
| **Sweden** | **7350 (4378 , 12091)** | **85.5 (49.4 , 142.4)** | **6229 (3603 , 10341)** | **58.5 (33.2 , 100.3)** | **-31.5 (-53.5 , -0.7)** |
| **Switzerland** | **5143 (2948 , 8327)** | **78.9 (44.1 , 130.6)** | **4521 (2518 , 7591)** | **51.2 (28.2 , 88.1)** | **-35.1 (-58.5 , -2)** |
| **United Kingdom** | **93694 (58971 , 141742)** | **170.1 (106.8 , 257.5)** | **75125 (46285 , 117001)** | **116.3 (70.8 , 182.3)** | **-31.6 (-46.7 , -10.8)** |
| **Southern Latin America** | **174290 (110593 , 261928)** | **351.6 (224.7 , 526.2)** | **131795 (81751 , 202574)** | **205.9 (126.8 , 316.7)** | **-41.5 (-52.1 , -29.7)** |
| **Argentina** | **142528 (90160 , 218995)** | **427.8 (271.6 , 656.1)** | **110489 (69103 , 172014)** | **255.3 (157.1 , 399.4)** | **-40.3 (-52.7 , -26.6)** |
| **Chile** | **21737 (13332 , 33369)** | **173.2 (108.1 , 264.7)** | **14609 (8638 , 23771)** | **72.4 (42.1 , 116.7)** | **-58.2 (-68 , -46.6)** |
| **Uruguay** | **10018 (6175 , 15607)** | **329.1 (200.6 , 511.1)** | **6690 (4072 , 10468)** | **204.5 (122.1 , 321.8)** | **-37.9 (-52.3 , -19.6)** |
| **Eastern Europe** | **678143 (439672 , 1016172)** | **285.8 (184.9 , 427.1)** | **491571 (311459 , 755427)** | **196.6 (122.7 , 301.5)** | **-31.2 (-41.9 , -19.8)** |
| **Belarus** | **33713 (20654 , 51446)** | **310.9 (190.8 , 476.1)** | **20048 (12408 , 31169)** | **185.2 (112.8 , 293)** | **-40.4 (-53 , -24.8)** |
| **Estonia** | **4925 (3043 , 7594)** | **300.1 (185.7 , 463.7)** | **2557 (1587 , 4022)** | **165 (101 , 270.8)** | **-45 (-57.6 , -30)** |
| **Latvia** | **8597 (5251 , 13459)** | **306.1 (183.9 , 483.9)** | **4669 (2865 , 7196)** | **206.8 (124.9 , 321.1)** | **-32.5 (-47.1 , -13.7)** |
| **Lithuania** | **10752 (6689 , 16763)** | **282.2 (175.1 , 442.1)** | **6915 (4278 , 10942)** | **210.3 (125.7 , 332.6)** | **-25.5 (-40.3 , -6.9)** |
| **Republic of Moldova** | **23805 (15284 , 35920)** | **533.7 (340.7 , 804.3)** | **13822 (8722 , 21053)** | **343 (217.1 , 518.7)** | **-35.7 (-47.1 , -22.9)** |
| **Russian Federation** | **488591 (309563 , 743648)** | **307.1 (194.7 , 466.3)** | **371595 (230604 , 578484)** | **210.6 (129.9 , 322.9)** | **-31.4 (-44.9 , -16.4)** |
| **Ukraine** | **107759 (66142 , 166992)** | **199.6 (122.9 , 311.2)** | **71964 (44413 , 113818)** | **142.8 (87.9 , 226.5)** | **-28.4 (-44.3 , -8.7)** |
| **Central Europe** | **428624 (273625 , 636875)** | **364.5 (232.6 , 538.7)** | **232928 (148386 , 358829)** | **226.6 (141.9 , 347.2)** | **-37.8 (-43.9 , -30.8)** |
| **Albania** | **18592 (11592 , 28191)** | **549.1 (346.6 , 830)** | **8442 (5382 , 12761)** | **331.4 (211.9 , 503.2)** | **-39.6 (-50.3 , -26.8)** |
| **Bosnia and Herzegovina** | **19088 (11812 , 28748)** | **424.1 (260.4 , 645.3)** | **8318 (5230 , 13122)** | **273.2 (169.6 , 430)** | **-35.6 (-48.4 , -19)** |
| **Bulgaria** | **27132 (16507 , 41436)** | **337.3 (200.6 , 513.9)** | **16998 (10609 , 27277)** | **274.6 (171.4 , 430.1)** | **-18.6 (-36.2 , 2.3)** |
| **Croatia** | **10167 (6116 , 16200)** | **214.6 (128.2 , 344.1)** | **5955 (3524 , 9590)** | **148.2 (86.5 , 237.1)** | **-30.9 (-47.6 , -7.8)** |
| **Czechia** | **25873 (15786 , 40290)** | **272.3 (167.5 , 422.4)** | **16370 (9681 , 26273)** | **169.4 (101.6 , 272.5)** | **-37.8 (-51.9 , -18.9)** |
| **Hungary** | **26435 (16504 , 39858)** | **287.3 (178.5 , 436.4)** | **15165 (9045 , 24241)** | **185.8 (110.1 , 299.2)** | **-35.3 (-51.6 , -13.5)** |
| **Montenegro** | **1678 (1018 , 2635)** | **271.4 (165.6 , 423.8)** | **1275 (775 , 1975)** | **218.8 (131.2 , 336.9)** | **-19.4 (-36.9 , 3.4)** |
| **North Macedonia** | **7737 (4833 , 11792)** | **390.5 (244.9 , 593.7)** | **4794 (2946 , 7534)** | **255.6 (155.9 , 400.9)** | **-34.6 (-46.6 , -16.7)** |
| **Poland** | **148100 (90539 , 226662)** | **398.8 (247 , 610.9)** | **80771 (49599 , 128845)** | **230.6 (137.8 , 365.7)** | **-42.2 (-53.9 , -26.6)** |
| **Romania** | **89238 (54595 , 131688)** | **400.4 (245.4 , 594.4)** | **43035 (26746 , 66629)** | **256.2 (155.2 , 400)** | **-36 (-49.5 , -18.7)** |
| **Serbia** | **34961 (21656 , 53850)** | **384.3 (235.2 , 596.6)** | **19077 (11071 , 29986)** | **239.9 (141.4 , 385.9)** | **-37.6 (-52.1 , -22.3)** |
| **Slovakia** | **15206 (9297 , 23707)** | **299.3 (185 , 464.6)** | **9969 (6111 , 15955)** | **197.2 (119.6 , 310.7)** | **-34.1 (-49.3 , -14.3)** |
| **Slovenia** | **4418 (2718 , 6756)** | **238.2 (143 , 368.4)** | **2759 (1638 , 4517)** | **146 (86.9 , 236.7)** | **-38.7 (-53.4 , -18.3)** |
| **Central Asia** | **623987 (412777 , 915967)** | **884.1 (584.9 , 1297.1)** | **640516 (415160 , 948555)** | **681.9 (441.4 , 1011.3)** | **-22.9 (-28.6 , -16.8)** |
| **Armenia** | **16818 (10595 , 25279)** | **495.7 (312.7 , 746.2)** | **11349 (7114 , 17096)** | **385.1 (240.6 , 576.4)** | **-22.3 (-35.9 , -6.5)** |
| **Azerbaijan** | **54342 (34779 , 80254)** | **746.6 (480.3 , 1101.6)** | **54652 (34683 , 82260)** | **526.3 (334.3 , 790.3)** | **-29.5 (-40 , -18)** |
| **Georgia** | **32646 (21209 , 48878)** | **597.8 (387.9 , 895.7)** | **19761 (12575 , 29256)** | **514.6 (326.9 , 748.4)** | **-13.9 (-27.6 , 1.6)** |
| **Kazakhstan** | **143048 (94192 , 211544)** | **878.5 (579.9 , 1307.3)** | **113497 (71594 , 169776)** | **607.8 (384.6 , 909.7)** | **-30.8 (-40.6 , -20.1)** |
| **Kyrgyzstan** | **39114 (25470 , 57035)** | **865.8 (564.5 , 1267.3)** | **42941 (27749 , 65755)** | **652.5 (423.9 , 991.5)** | **-24.6 (-35.3 , -12.7)** |
| **Mongolia** | **20943 (13575 , 30677)** | **980.2 (636.7 , 1417.3)** | **19184 (12345 , 28626)** | **572.5 (370.7 , 843.9)** | **-41.6 (-50.1 , -32.1)** |
| **Tajikistan** | **42443 (27947 , 61861)** | **757.6 (500.3 , 1092.8)** | **56779 (36289 , 82809)** | **613.4 (399 , 896.9)** | **-19 (-30.3 , -6.1)** |
| **Turkmenistan** | **27759 (18215 , 41637)** | **738.9 (486.6 , 1102.4)** | **25900 (16376 , 38835)** | **509.9 (321.9 , 765.6)** | **-31 (-41.3 , -18.7)** |
| **Uzbekistan** | **246874 (160249 , 359069)** | **1122.1 (737.1 , 1617.5)** | **296453 (188517 , 435485)** | **867.5 (549.3 , 1277.5)** | **-22.7 (-33.4 , -10.7)** |
| **Central Latin America** | **598408 (390003 , 894761)** | **364.5 (238.7 , 543.8)** | **518615 (338670 , 765747)** | **215.7 (141 , 318.5)** | **-40.8 (-44.5 , -37.1)** |
| **Colombia** | **119935 (72519 , 181997)** | **380.5 (234.9 , 570)** | **72046 (43999 , 110096)** | **151.3 (92.8 , 232.1)** | **-60.2 (-68.6 , -49.7)** |
| **Costa Rica** | **9429 (5854 , 14751)** | **305.9 (189.7 , 471.1)** | **8591 (5205 , 13615)** | **186.9 (113.1 , 295.1)** | **-38.9 (-52.3 , -21.2)** |
| **El Salvador** | **21803 (13615 , 33905)** | **405 (257.5 , 616)** | **14845 (8955 , 22891)** | **243.1 (146.8 , 375.5)** | **-40 (-53.2 , -24.6)** |
| **Guatemala** | **53873 (35019 , 80272)** | **643.3 (414.7 , 952.6)** | **68460 (42832 , 103575)** | **390.3 (249 , 589.1)** | **-39.3 (-49.5 , -28.1)** |
| **Honduras** | **28389 (17912 , 42427)** | **561.4 (362.4 , 833.6)** | **33305 (20729 , 50296)** | **353 (223.2 , 528.7)** | **-37.1 (-47.6 , -24.3)** |
| **Mexico** | **274961 (180641 , 399839)** | **316.6 (207.9 , 460.2)** | **242746 (160818 , 359054)** | **203.2 (134.5 , 300.4)** | **-35.8 (-39.3 , -32.2)** |
| **Nicaragua** | **14452 (8856 , 22180)** | **399.8 (252.8 , 599.5)** | **9932 (5849 , 15625)** | **168.3 (100.7 , 259.2)** | **-57.9 (-66.3 , -47.9)** |
| **Panama** | **11754 (7352 , 17497)** | **503.8 (316.2 , 747)** | **13570 (8268 , 20440)** | **325.8 (198.6 , 490.5)** | **-35.3 (-47.6 , -20.8)** |
| **Venezuela (Bolivarian Republic of)** | **63812 (39181 , 98551)** | **351.9 (221.1 , 537.7)** | **55119 (33884 , 86794)** | **201.2 (124 , 315.2)** | **-42.8 (-53.7 , -28.1)** |
| **Andean Latin America** | **305793 (197376 , 455780)** | **761.7 (497.5 , 1123.7)** | **219766 (139238 , 329651)** | **347.6 (220.7 , 520.1)** | **-54.4 (-59.8 , -48.1)** |
| **Bolivia (Plurinational State of)** | **66010 (43648 , 96431)** | **994.8 (650.9 , 1457.4)** | **76888 (48791 , 114761)** | **610.5 (386 , 901.6)** | **-38.6 (-48 , -27.9)** |
| **Ecuador** | **51750 (32804 , 79516)** | **497.8 (318.6 , 746.2)** | **30178 (19400 , 46707)** | **177.5 (113.6 , 272.9)** | **-64.3 (-71.1 , -56.1)** |
| **Peru** | **188033 (119713 , 280410)** | **819.1 (526.2 , 1220)** | **112699 (69836 , 173187)** | **338.3 (209.9 , 520.2)** | **-58.7 (-66.6 , -49.7)** |
| **Caribbean** | **247026 (161827 , 361514)** | **690.7 (454 , 1014.3)** | **288251 (187090 , 427217)** | **629.5 (409.4 , 931.5)** | **-8.9 (-16.3 , -1.8)** |
| **Antigua and Barbuda** | **381 (242 , 588)** | **623.6 (394.4 , 959.1)** | **392 (243 , 587)** | **467.3 (288.7 , 703.3)** | **-25.1 (-38.5 , -9.3)** |
| **Barbados** | **1144 (728 , 1735)** | **465.5 (297.6 , 701.3)** | **874 (530 , 1376)** | **335.8 (203.8 , 530.2)** | **-27.9 (-42.6 , -10.2)** |
| **Belize** | **1466 (911 , 2206)** | **746.9 (469.7 , 1107.3)** | **2551 (1564 , 3826)** | **629.9 (389.7 , 942.6)** | **-15.7 (-29.2 , 0.6)** |
| **Bermuda** | **229 (141 , 354)** | **399.5 (248.5 , 626.1)** | **134 (81 , 208)** | **220.7 (131.5 , 350.6)** | **-44.7 (-58 , -28.5)** |
| **Bahamas** | **1522 (940 , 2327)** | **597.2 (369.7 , 908.3)** | **1820 (1128 , 2744)** | **499 (311.4 , 752.9)** | **-16.4 (-30.6 , 1.1)** |
| **Cuba** | **47972 (29914 , 72692)** | **457.8 (284.9 , 700.4)** | **35856 (20846 , 56843)** | **329.9 (189.7 , 524.7)** | **-27.9 (-44.2 , -10.9)** |
| **Dominica** | **392 (248 , 609)** | **539.4 (340.4 , 838.6)** | **326 (205 , 494)** | **468.9 (293.9 , 712.5)** | **-13.1 (-27.7 , 4.6)** |
| **Dominican Republic** | **49448 (31743 , 73493)** | **691.3 (445.5 , 1028.5)** | **46847 (29011 , 71019)** | **432.4 (267.7 , 654.1)** | **-37.4 (-47.3 , -26.1)** |
| **Grenada** | **631 (410 , 937)** | **688.2 (448 , 1024.5)** | **483 (298 , 736)** | **507.1 (311.8 , 763.7)** | **-26.3 (-38.5 , -12)** |
| **Guyana** | **9131 (5983 , 13611)** | **1193.6 (781 , 1777.2)** | **6292 (3967 , 9268)** | **834.7 (524.6 , 1224.5)** | **-30.1 (-39.1 , -19.7)** |
| **Haiti** | **84116 (54027 , 124080)** | **1290.9 (845.1 , 1892.5)** | **147995 (96787 , 220441)** | **1160.5 (765.1 , 1720.6)** | **-10.1 (-20.4 , 1)** |
| **Jamaica** | **14174 (8654 , 21377)** | **594 (368.8 , 896.7)** | **13416 (8293 , 20536)** | **497.5 (309.1 , 759.7)** | **-16.2 (-32.4 , 1)** |
| **Puerto Rico** | **14444 (8620 , 22424)** | **406.4 (243.4 , 635)** | **8784 (5249 , 13840)** | **261.7 (152.8 , 420.2)** | **-35.6 (-50.5 , -16.3)** |
| **Saint Kitts and Nevis** | **232 (142 , 353)** | **561.3 (347.8 , 864.2)** | **231 (144 , 353)** | **401.7 (248.7 , 613.6)** | **-28.4 (-42.6 , -10.6)** |
| **Saint Lucia** | **1007 (621 , 1541)** | **720.4 (454.7 , 1086.2)** | **877 (542 , 1356)** | **522.5 (324.6 , 817.4)** | **-27.5 (-40.4 , -12.8)** |
| **Saint Vincent and the Grenadines** | **766 (480 , 1159)** | **686 (430.2 , 1042.3)** | **657 (410 , 1002)** | **595.2 (368.9 , 901.2)** | **-13.2 (-27.6 , 3.5)** |
| **Suriname** | **3057 (1929 , 4584)** | **782 (495.3 , 1167.4)** | **3668 (2296 , 5464)** | **643.3 (400.8 , 959.9)** | **-17.7 (-30.7 , -3)** |
| **Trinidad and Tobago** | **8124 (5208 , 12223)** | **667 (430.2 , 995)** | **6874 (4172 , 10415)** | **513.5 (307.7 , 785.3)** | **-23 (-37.6 , -6.7)** |
| **United States Virgin Islands** | **562 (344 , 872)** | **529.9 (326.4 , 819.3)** | **410 (253 , 640)** | **407.5 (249.5 , 630.9)** | **-23.1 (-37.6 , -4)** |
| **Tropical Latin America** | **1135088 (723760 , 1694993)** | **744.1 (475.3 , 1122)** | **1069215 (681813 , 1668724)** | **482.7 (308.4 , 738.4)** | **-35.1 (-45 , -23.8)** |
| **Brazil** | **1109975 (707374 , 1656735)** | **747.8 (476.4 , 1128.8)** | **1041949 (663207 , 1623391)** | **485.4 (309.8 , 742.8)** | **-35.1 (-45.2 , -23.4)** |
| **Paraguay** | **25113 (15947 , 37803)** | **600.9 (386.1 , 896.1)** | **27265 (16748 , 41245)** | **399 (245.7 , 603.3)** | **-33.6 (-44.8 , -20.6)** |
| **East Asia** | **6291117 (4163036 , 9124713)** | **536.3 (355.5 , 778.6)** | **2421967 (1515435 , 3828239)** | **156.8 (97.3 , 242.3)** | **-70.8 (-74.8 , -66.6)** |
| **China** | **6102739 (4033955 , 8852339)** | **539.1 (357.5 , 782.3)** | **2258222 (1411692 , 3558089)** | **150.4 (93.3 , 233.5)** | **-72.1 (-76.1 , -67.8)** |
| **Democratic People's Republic of Korea** | **125974 (81425 , 189127)** | **594.9 (383 , 894.6)** | **124087 (76898 , 188465)** | **476.1 (289.6 , 725.8)** | **-20 (-33.2 , -4.9)** |
| **Taiwan (Province of China)** | **62405 (38232 , 95655)** | **319.5 (196.6 , 485.6)** | **39658 (23808 , 62235)** | **161.3 (96.5 , 258.9)** | **-49.5 (-61 , -35.5)** |
| **Southeast Asia** | **3768376 (2454042 , 5553513)** | **819.2 (533.8 , 1208.5)** | **2933216 (1906247 , 4391293)** | **454 (295.7 , 679.7)** | **-44.6 (-48.8 , -40.1)** |
| **Cambodia** | **143864 (92758 , 210824)** | **1282.5 (847.8 , 1868)** | **131833 (84152 , 194194)** | **805.2 (515.7 , 1189.9)** | **-37.2 (-45.8 , -27.5)** |
| **Indonesia** | **1649451 (1092539 , 2466519)** | **913 (601.7 , 1352.9)** | **1227692 (789774 , 1841859)** | **496.1 (320.1 , 743)** | **-45.7 (-53.1 , -37.9)** |
| **Lao People's Democratic Republic** | **45589 (29168 , 67237)** | **1084.1 (712.7 , 1587.3)** | **47251 (29929 , 71408)** | **690.2 (440.8 , 1035.2)** | **-36.3 (-44.6 , -26.3)** |
| **Malaysia** | **136248 (88731 , 201097)** | **770.7 (501.2 , 1138.2)** | **135239 (86703 , 204728)** | **445.9 (282 , 675)** | **-42.1 (-51.6 , -32.2)** |
| **Maldives** | **3513 (2259 , 5056)** | **1325.4 (855.5 , 1903.8)** | **2166 (1340 , 3329)** | **469.7 (290 , 716.4)** | **-64.6 (-71.1 , -57.6)** |
| **Mauritius** | **7263 (4781 , 10769)** | **690.6 (450.3 , 1023.7)** | **5219 (3316 , 7988)** | **457.3 (293.5 , 695.5)** | **-33.8 (-45.8 , -20.4)** |
| **Myanmar** | **505438 (322411 , 745370)** | **1186.4 (758.8 , 1766.3)** | **474318 (300942 , 712036)** | **892.6 (567.7 , 1346.1)** | **-24.8 (-34.2 , -14.6)** |
| **Philippines** | **412136 (262194 , 614634)** | **668.5 (436.9 , 995.9)** | **401222 (249219 , 617778)** | **371.9 (232.7 , 565.8)** | **-44.4 (-53 , -34.7)** |
| **Sri Lanka** | **143127 (93254 , 212460)** | **839.3 (543.7 , 1240.4)** | **79677 (47551 , 123349)** | **360.2 (217.8 , 561.5)** | **-57.1 (-65.2 , -48.5)** |
| **Seychelles** | **447 (285 , 683)** | **623.7 (396.8 , 953.2)** | **359 (220 , 547)** | **365.9 (223.6 , 564.2)** | **-41.3 (-52.4 , -28.2)** |
| **Thailand** | **249219 (157978 , 374988)** | **490.4 (311.4 , 731.5)** | **172980 (107649 , 273008)** | **243.1 (150.1 , 386.9)** | **-50.4 (-60.5 , -39)** |
| **Timor-Leste** | **7901 (5101 , 11666)** | **924.7 (601.2 , 1358.1)** | **8185 (5171 , 12241)** | **586.2 (369.2 , 870.1)** | **-36.6 (-46 , -25.7)** |
| **Viet Nam** | **459170 (293814 , 688044)** | **673.3 (435 , 1007.5)** | **243232 (149842 , 381608)** | **268.7 (166.9 , 417.7)** | **-60.1 (-67 , -51.1)** |
| **Oceania** | **70323 (46259 , 101768)** | **1043.5 (682.1 , 1506)** | **123409 (80514 , 181276)** | **907.2 (594.6 , 1321.3)** | **-13.1 (-21.7 , -4.1)** |
| **American Samoa** | **276 (175 , 418)** | **589.2 (369.5 , 875.1)** | **265 (170 , 397)** | **505 (322.9 , 761.1)** | **-14.3 (-28 , 0.3)** |
| **Cook Islands** | **100 (61 , 155)** | **524.7 (325.3 , 812.3)** | **70 (44 , 111)** | **390.8 (240.7 , 619.2)** | **-25.5 (-39.6 , -8.4)** |
| **Micronesia (Federated States of)** | **1072 (713 , 1609)** | **976.2 (646.6 , 1469.9)** | **691 (446 , 1028)** | **718.6 (465.4 , 1060.4)** | **-26.4 (-36.1 , -15.7)** |
| **Fiji** | **5600 (3613 , 8267)** | **746 (479.7 , 1094.4)** | **6731 (4300 , 10186)** | **757.7 (482.6 , 1137.8)** | **1.6 (-11.7 , 17)** |
| **Guam** | **673 (415 , 1009)** | **496.8 (309.7 , 753.6)** | **779 (474 , 1178)** | **456.6 (276.6 , 692.5)** | **-8.1 (-23.8 , 11.1)** |
| **Kiribati** | **812 (540 , 1198)** | **1090.8 (725.8 , 1596.6)** | **1116 (729 , 1651)** | **967 (641 , 1419.5)** | **-11.4 (-21.9 , 1.2)** |
| **Marshall Islands** | **465 (299 , 692)** | **906.5 (587.6 , 1340.2)** | **414 (268 , 617)** | **749.7 (490.9 , 1098.2)** | **-17.3 (-27.6 , -5.6)** |
| **Nauru** | **77 (49 , 116)** | **710.8 (453.8 , 1059.1)** | **62 (39 , 96)** | **603.3 (385.2 , 915.2)** | **-15.1 (-27.8 , -0.6)** |
| **Niue** | **15 (10 , 23)** | **639.9 (407.6 , 965.7)** | **7 (5 , 11)** | **447.5 (277.7 , 681.7)** | **-30.1 (-42.2 , -16.6)** |
| **Northern Mariana Islands** | **215 (130 , 329)** | **478.8 (291.8 , 720.5)** | **159 (96 , 246)** | **392.7 (237 , 606)** | **-18 (-34.2 , 1.5)** |
| **Palau** | **86 (53 , 130)** | **573.1 (357.4 , 862.1)** | **73 (46 , 111)** | **426.8 (262.4 , 643.9)** | **-25.5 (-38.6 , -10.1)** |
| **Papua New Guinea** | **50302 (32939 , 73048)** | **1173.1 (763.7 , 1687.4)** | **97597 (62976 , 142880)** | **955.8 (621.4 , 1391.3)** | **-18.5 (-28.7 , -7.8)** |
| **Samoa** | **917 (573 , 1372)** | **551.3 (344.1 , 824.9)** | **935 (578 , 1421)** | **456.2 (286.2 , 693.3)** | **-17.2 (-31.7 , 0.1)** |
| **Solomon Islands** | **3637 (2375 , 5361)** | **989.7 (649.7 , 1437.5)** | **5544 (3521 , 8151)** | **827.8 (533.2 , 1212.2)** | **-16.4 (-27.2 , -2.6)** |
| **Tokelau** | **15 (10 , 22)** | **840.3 (543.8 , 1242.6)** | **8 (5 , 12)** | **559.9 (359 , 841.8)** | **-33.4 (-44.3 , -21.2)** |
| **Tonga** | **645 (415 , 954)** | **705.8 (452.9 , 1037.8)** | **576 (363 , 881)** | **586.9 (372.7 , 894.9)** | **-16.8 (-30.4 , -1.3)** |
| **Tuvalu** | **90 (57 , 135)** | **948.7 (606.9 , 1408.3)** | **78 (50 , 118)** | **683.4 (439.2 , 1028.6)** | **-28 (-38.4 , -16.3)** |
| **Vanuatu** | **1433 (943 , 2122)** | **951.8 (630 , 1400.4)** | **2474 (1608 , 3682)** | **869.6 (569.8 , 1294.5)** | **-8.6 (-20 , 4)** |
| **North Africa and Middle East** | **2477794 (1642022 , 3658563)** | **682.4 (455.1 , 1005.7)** | **2613567 (1704030 , 3884951)** | **434.8 (283.2 , 648.5)** | **-36.3 (-40.2 , -32.5)** |
| **Afghanistan** | **89449 (57504 , 132613)** | **748.3 (485.8 , 1115.8)** | **216977 (137441 , 328395)** | **544 (347.9 , 808.7)** | **-27.3 (-38 , -14.8)** |
| **Algeria** | **161832 (99776 , 244236)** | **611.3 (385.7 , 906.1)** | **150544 (93365 , 237491)** | **361 (223.5 , 567.5)** | **-40.9 (-52.6 , -26.5)** |
| **Bahrain** | **3258 (2070 , 5021)** | **628.8 (402.2 , 962.2)** | **4377 (2681 , 6981)** | **337.3 (210.3 , 527.6)** | **-46.4 (-58 , -32.5)** |
| **Egypt** | **438305 (275033 , 663400)** | **750.7 (479 , 1125.5)** | **399400 (246074 , 619171)** | **398.2 (247 , 614.4)** | **-47 (-56.9 , -36.4)** |
| **Iran (Islamic Republic of)** | **300718 (188735 , 441140)** | **498.1 (320.8 , 736.8)** | **174628 (106834 , 273129)** | **220.5 (136.1 , 339.6)** | **-55.7 (-64 , -45.8)** |
| **Iraq** | **110924 (69243 , 167985)** | **614.5 (390.5 , 925)** | **153493 (91152 , 241211)** | **377 (227.9 , 583.4)** | **-38.6 (-49.4 , -26.1)** |
| **Jordan** | **24619 (15223 , 36998)** | **656.1 (421.7 , 975.5)** | **45112 (28295 , 67586)** | **392.2 (248.2 , 587.3)** | **-40.2 (-49.9 , -29.3)** |
| **Kuwait** | **7815 (5021 , 11530)** | **429.2 (276 , 632.3)** | **12209 (7431 , 18596)** | **282.4 (172.6 , 432.8)** | **-34.2 (-46.6 , -19.2)** |
| **Lebanon** | **16206 (10154 , 24237)** | **481.4 (304.9 , 719.2)** | **9394 (5472 , 15641)** | **181.9 (105.3 , 302.1)** | **-62.2 (-70.9 , -50.6)** |
| **Libya** | **25795 (16061 , 39573)** | **570.5 (361.5 , 865.4)** | **25737 (15787 , 39531)** | **408.5 (249.3 , 622.7)** | **-28.4 (-41.3 , -11.8)** |
| **Morocco** | **206646 (132728 , 306536)** | **796.4 (516.9 , 1172)** | **173188 (106578 , 263267)** | **496.9 (307.9 , 755.7)** | **-37.6 (-48.4 , -25.4)** |
| **Palestine** | **12566 (7890 , 19228)** | **586.1 (367.6 , 890.1)** | **16946 (10400 , 25874)** | **347.3 (214.1 , 531.4)** | **-40.8 (-51.6 , -28.5)** |
| **Oman** | **18974 (12303 , 28367)** | **907.5 (595.3 , 1340.1)** | **15354 (8982 , 23894)** | **431.2 (259 , 664.8)** | **-52.5 (-62.2 , -42)** |
| **Qatar** | **1665 (1030 , 2590)** | **415.5 (258.9 , 640.7)** | **3783 (2130 , 6042)** | **157.1 (90.3 , 254.9)** | **-62.2 (-70.9 , -52.2)** |
| **Saudi Arabia** | **53668 (34012 , 82342)** | **344.4 (220.2 , 525.8)** | **48738 (29201 , 77900)** | **166 (99.4 , 258.4)** | **-51.8 (-62.3 , -40.2)** |
| **Sudan** | **234994 (149502 , 343085)** | **1030.7 (666.3 , 1510.1)** | **312499 (198216 , 473243)** | **711.8 (457.4 , 1066.9)** | **-30.9 (-40.5 , -19.8)** |
| **Syrian Arab Republic** | **93362 (58806 , 141158)** | **669.5 (423.9 , 1005)** | **55905 (34379 , 86183)** | **407.2 (251.2 , 626.5)** | **-39.2 (-51.2 , -25.9)** |
| **Tunisia** | **34655 (22094 , 51767)** | **399.6 (256.9 , 595.8)** | **24099 (14311 , 37717)** | **215.6 (127.9 , 342.4)** | **-46 (-57.3 , -32.5)** |
| **Turkey** | **421341 (273738 , 640810)** | **691.6 (452.1 , 1042.9)** | **239536 (147976 , 372868)** | **311.2 (190.8 , 492.1)** | **-55 (-64.6 , -44.5)** |
| **United Arab Emirates** | **8700 (5218 , 13296)** | **481.1 (296 , 728.3)** | **22872 (13169 , 35615)** | **307.1 (179.1 , 467.9)** | **-36.2 (-48.7 , -20.6)** |
| **Yemen** | **210635 (141174 , 305237)** | **1305.7 (882.2 , 1898.3)** | **506121 (338800 , 749911)** | **1444.1 (970.4 , 2133.1)** | **10.6 (-0.2 , 23.1)** |
| **South Asia** | **19440166 (13075762 , 27962135)** | **1755.7 (1179 , 2513.3)** | **23719569 (15826969 , 34432493)** | **1358.2 (907.3 , 1972.1)** | **-22.6 (-25.3 , -20.1)** |
| **Bangladesh** | **1498380 (978188 , 2199128)** | **1358.3 (890.8 , 1992.2)** | **1215790 (771624 , 1852532)** | **802.9 (509.5 , 1221.6)** | **-40.9 (-48.5 , -31.8)** |
| **Bhutan** | **14138 (9397 , 20419)** | **2080.5 (1396.4 , 2994.9)** | **10672 (6983 , 15821)** | **1524.5 (1002.9 , 2248.2)** | **-26.7 (-34.3 , -19)** |
| **India** | **15849029 (10685397 , 22744517)** | **1847.6 (1248.9 , 2636)** | **19308334 (12924471 , 27847930)** | **1441 (965.6 , 2077.1)** | **-22 (-24.5 , -19.7)** |
| **Nepal** | **253140 (169582 , 378299)** | **1358.4 (914.8 , 2001.8)** | **276007 (178462 , 417402)** | **954.6 (617.7 , 1431.7)** | **-29.7 (-38.1 , -19.9)** |
| **Pakistan** | **1825480 (1216599 , 2647391)** | **1537.2 (1029 , 2201)** | **2908766 (1866086 , 4347930)** | **1285.7 (841.6 , 1891.8)** | **-16.4 (-25.1 , -6.5)** |
| **Southern Sub-Saharan Africa** | **376815 (247060 , 561368)** | **705.3 (460.1 , 1044.1)** | **445660 (293732 , 652913)** | **572.1 (379.6 , 834.9)** | **-18.9 (-27.1 , -10)** |
| **Botswana** | **13577 (8906 , 19866)** | **934.6 (620.8 , 1361.9)** | **14340 (9156 , 21545)** | **607.5 (385.5 , 912)** | **-35 (-45 , -24.3)** |
| **Lesotho** | **15342 (9747 , 22828)** | **781.6 (504.3 , 1155.1)** | **14678 (9418 , 21689)** | **711.6 (461 , 1045.5)** | **-9 (-21 , 5.8)** |
| **Namibia** | **15553 (9828 , 23058)** | **1001.3 (644.1 , 1482.1)** | **15152 (9689 , 22488)** | **604.4 (387.8 , 896.8)** | **-39.6 (-48 , -29.9)** |
| **South Africa** | **246015 (159110 , 362217)** | **668.7 (438.5 , 987.5)** | **281046 (186028 , 409693)** | **507.7 (337.1 , 739.8)** | **-24.1 (-34.9 , -11.6)** |
| **Eswatini** | **6129 (3828 , 9387)** | **700.2 (445.7 , 1055.2)** | **6489 (4090 , 9687)** | **574.4 (365.3 , 845.8)** | **-18 (-30.6 , -2.9)** |
| **Zimbabwe** | **80200 (49785 , 122882)** | **730.2 (462.9 , 1091.3)** | **113955 (71806 , 170666)** | **745 (479 , 1108.6)** | **2 (-12.7 , 19.9)** |
| **Western Sub-Saharan Africa** | **3436819 (2293131 , 4937123)** | **1405.9 (936.9 , 2045.7)** | **7272620 (4861319 , 10527853)** | **1317.8 (879.9 , 1912.7)** | **-6.3 (-11 , -1.6)** |
| **Benin** | **70764 (46372 , 104263)** | **1149.1 (758.9 , 1706.8)** | **170243 (111317 , 248903)** | **1104.4 (727.5 , 1632.6)** | **-3.9 (-14.3 , 7.9)** |
| **Burkina Faso** | **210928 (141469 , 301921)** | **1661.4 (1108 , 2403.1)** | **473379 (315481 , 691323)** | **1620 (1077.6 , 2362.7)** | **-2.5 (-11.5 , 8.3)** |
| **Cameroon** | **120502 (77184 , 174969)** | **940.7 (601.9 , 1365.6)** | **291252 (187088 , 427666)** | **882.9 (565.9 , 1296.9)** | **-6.1 (-18.1 , 7.7)** |
| **Cabo Verde** | **3896 (2506 , 5844)** | **910.3 (592.3 , 1346.4)** | **3619 (2227 , 5461)** | **644.4 (396.6 , 974.3)** | **-29.2 (-41.4 , -16.1)** |
| **Chad** | **99648 (63761 , 145345)** | **1303.9 (844.1 , 1890.8)** | **260593 (171652 , 386294)** | **1230.8 (802.5 , 1815.9)** | **-5.6 (-16.7 , 7.2)** |
| **CÃ´te d'Ivoire** | **236028 (156804 , 339988)** | **1593.3 (1064.6 , 2305.7)** | **403786 (266009 , 586492)** | **1341.9 (872 , 1941.4)** | **-15.8 (-24.2 , -7)** |
| **Gambia** | **21345 (14244 , 30899)** | **1657.1 (1090.7 , 2376.3)** | **38194 (24819 , 55526)** | **1462.1 (955.4 , 2145)** | **-11.8 (-20.9 , -2.3)** |
| **Ghana** | **240559 (157659 , 345869)** | **1344.8 (888.2 , 1932.7)** | **382853 (247862 , 559508)** | **1131.6 (737.4 , 1658.5)** | **-15.9 (-26.4 , -4.5)** |
| **Guinea** | **100531 (66293 , 145156)** | **1349.6 (893.5 , 1961.6)** | **194343 (127634 , 284944)** | **1279.4 (845.9 , 1877.6)** | **-5.2 (-16 , 6.2)** |
| **Guinea-Bissau** | **16818 (11059 , 24173)** | **1357.3 (899.9 , 1963.7)** | **26638 (17384 , 39083)** | **1206.1 (778.8 , 1774.7)** | **-11.1 (-22.1 , 0.2)** |
| **Liberia** | **34002 (22706 , 49420)** | **1480.1 (990.3 , 2141.7)** | **57781 (37125 , 86366)** | **1088.7 (703.8 , 1620.7)** | **-26.4 (-35.7 , -17.2)** |
| **Mali** | **195200 (129326 , 281051)** | **1821.7 (1213.7 , 2601.2)** | **469718 (310822 , 675519)** | **1681.7 (1110.1 , 2438.7)** | **-7.7 (-17.1 , 1.5)** |
| **Mauritania** | **31129 (20543 , 46274)** | **1222.6 (811.8 , 1804)** | **46393 (29932 , 69631)** | **996.8 (647.9 , 1501.2)** | **-18.5 (-30 , -6.1)** |
| **Niger** | **166452 (109086 , 245821)** | **1619.2 (1063.4 , 2390.7)** | **416153 (271412 , 611290)** | **1334.6 (871.6 , 1940.2)** | **-17.6 (-26.8 , -8.1)** |
| **Nigeria** | **1594443 (1069069 , 2300521)** | **1367.8 (899.3 , 1986.3)** | **3550449 (2320283 , 5175436)** | **1351.1 (897.4 , 1959.9)** | **-1.2 (-10.4 , 8.6)** |
| **Sao Tome and Principe** | **1502 (951 , 2281)** | **1045.8 (667.7 , 1563.7)** | **1685 (1064 , 2532)** | **762.6 (480.3 , 1147)** | **-27.1 (-39.8 , -12.7)** |
| **Senegal** | **158949 (103901 , 227584)** | **1696.4 (1112.2 , 2439)** | **227678 (149860 , 332922)** | **1327.2 (874.2 , 1931.2)** | **-21.8 (-30.1 , -12.6)** |
| **Sierra Leone** | **67011 (44226 , 98138)** | **1534.3 (1013.3 , 2252.3)** | **138628 (90862 , 205494)** | **1417.3 (930.6 , 2096)** | **-7.6 (-17.5 , 2.6)** |
| **Togo** | **66998 (43851 , 99111)** | **1431.8 (931.7 , 2120.5)** | **119134 (78812 , 173737)** | **1307.4 (861.1 , 1905.1)** | **-8.7 (-17.5 , 1.7)** |
| **Eastern Sub-Saharan Africa** | **2923146 (1940080 , 4253163)** | **1334.3 (885.1 , 1933)** | **4380697 (2907670 , 6427045)** | **970.8 (646.2 , 1419.7)** | **-27.2 (-30.4 , -24)** |
| **Burundi** | **70789 (46251 , 102591)** | **1083.3 (708.3 , 1573)** | **121401 (80282 , 178600)** | **872.3 (576.3 , 1258.1)** | **-19.5 (-29.6 , -7.5)** |
| **Comoros** | **6825 (4511 , 10218)** | **1321.4 (879 , 1975.9)** | **6687 (4293 , 10086)** | **933.9 (607.4 , 1404.5)** | **-29.3 (-38.3 , -18.7)** |
| **Djibouti** | **6221 (3995 , 9184)** | **1143.8 (742.4 , 1705.4)** | **10066 (6425 , 15014)** | **815.8 (523.8 , 1229.6)** | **-28.7 (-38.9 , -17.8)** |
| **Eritrea** | **48761 (31754 , 71879)** | **1464.1 (963.3 , 2122.5)** | **70680 (45278 , 104244)** | **1015.7 (657.2 , 1500.2)** | **-30.6 (-39.5 , -21.8)** |
| **Ethiopia** | **664262 (438480 , 956148)** | **1058.6 (698.6 , 1531.3)** | **938339 (620818 , 1391334)** | **741.8 (492.6 , 1094.3)** | **-29.9 (-34.9 , -24.8)** |
| **Kenya** | **218468 (145953 , 313754)** | **875.9 (586.9 , 1262.7)** | **345219 (229631 , 504708)** | **718.7 (478.5 , 1045.6)** | **-17.9 (-20.2 , -15.7)** |
| **Madagascar** | **167869 (108920 , 245267)** | **1242 (823.7 , 1785.9)** | **248940 (160275 , 363611)** | **914.6 (593.1 , 1345.3)** | **-26.4 (-35.1 , -16.1)** |
| **Malawi** | **182856 (119789 , 264393)** | **1676.7 (1108.3 , 2422.9)** | **244301 (159274 , 357033)** | **1216.2 (795.2 , 1762.4)** | **-27.5 (-35.2 , -18.9)** |
| **Mozambique** | **244906 (159306 , 358700)** | **1631.4 (1072.1 , 2382.6)** | **422123 (279421 , 624516)** | **1248.8 (823 , 1847.3)** | **-23.5 (-31.7 , -14.1)** |
| **Rwanda** | **78543 (50248 , 115806)** | **1024.2 (656.9 , 1498.1)** | **76240 (48370 , 113683)** | **609.9 (386.7 , 896.9)** | **-40.5 (-48.8 , -31.4)** |
| **Somalia** | **132894 (88298 , 192008)** | **1729 (1147.7 , 2511.2)** | **292303 (191016 , 438645)** | **1339.5 (891.8 , 1980.5)** | **-22.5 (-30.7 , -14.3)** |
| **South Sudan** | **87637 (56862 , 127204)** | **1335.2 (877.9 , 1933.9)** | **118349 (76213 , 174398)** | **1159.5 (753.2 , 1694.7)** | **-13.2 (-23.2 , -1.4)** |
| **United Republic of Tanzania** | **568592 (374467 , 820652)** | **1921.9 (1260.3 , 2760.9)** | **736959 (476598 , 1083788)** | **1169.8 (761.3 , 1723.8)** | **-39.1 (-46.5 , -31.2)** |
| **Uganda** | **272435 (178198 , 396826)** | **1309 (861.7 , 1894.6)** | **399494 (257358 , 597407)** | **870.4 (569.5 , 1307.3)** | **-33.5 (-41.9 , -24.6)** |
| **Zambia** | **169943 (111344 , 245363)** | **1880.3 (1238.4 , 2722.3)** | **346093 (231670 , 499063)** | **1754.3 (1176.7 , 2517.7)** | **-6.7 (-16.1 , 2.9)** |
| **Central Sub-Saharan Africa** | **942777 (628865 , 1376172)** | **1521.5 (1013.6 , 2211.5)** | **1599655 (1049715 , 2328750)** | **1089.1 (721.3 , 1588.2)** | **-28.4 (-35 , -22)** |
| **Angola** | **106650 (68493 , 154105)** | **972 (628.6 , 1413.8)** | **270800 (170550 , 408074)** | **789.7 (497.4 , 1188.9)** | **-18.8 (-30.2 , -5.6)** |
| **Central African Republic** | **41638 (27025 , 61137)** | **1281.9 (838.1 , 1876.6)** | **73229 (46929 , 110320)** | **1229 (801.2 , 1825.3)** | **-4.1 (-15.2 , 8.4)** |
| **Congo** | **34873 (22421 , 51292)** | **1318.6 (854.7 , 1945.3)** | **61086 (39994 , 90685)** | **1095 (713 , 1611.6)** | **-17 (-26.7 , -5.9)** |
| **Democratic Republic of the Congo** | **734944 (492748 , 1065010)** | **1696.8 (1126.6 , 2451.3)** | **1156966 (754562 , 1695420)** | **1181.5 (776.1 , 1744.3)** | **-30.4 (-38.2 , -22.4)** |
| **Equatorial Guinea** | **7794 (5087 , 11457)** | **1607.7 (1052.9 , 2340.9)** | **14133 (9019 , 21008)** | **924.2 (586.3 , 1367.9)** | **-42.5 (-50.4 , -33.8)** |
| **Gabon** | **16877 (11159 , 24318)** | **1581.8 (1046.8 , 2289)** | **23441 (15123 , 34049)** | **1306.5 (850.1 , 1892.1)** | **-17.4 (-26.4 , -8.1)** |
